# Characterization of a Fungal Modular Polyketide Synthase System Reveals an Unusual Strategy for Polyketide Chain Initiation

**DOI:** 10.1002/anie.7344670

**Published:** 2026-09-30

**Authors:** Qiaolin Ji, Chao Peng, Shuhan He, Bin‐Tao Peng, Wei‐Guang Wang, Yudai Matsuda

**Affiliations:** ^1^ Department of Chemistry City University of Hong Kong Kowloon Hong Kong SAR China; ^2^ Key Laboratory of Natural Products Synthetic Biology of Ethnic Medicinal Endophytes, State Ethnic Affairs Commission; Key Laboratory of Chemistry in Ethnic Medicinal Resources Ministry of Education Yunnan Minzu University Kunming Yunnan China

**Keywords:** biosynthesis, enzymes, macrolides, natural products, polyketides

## Abstract

Polyketides constitute a major class of natural products with remarkable structural diversity and biological activities. While bacteria predominantly produce polyketides via modular type I polyketide synthases (PKSs), eukaryotic modular PKSs remain largely unexplored. Here, we report the functional characterization of a fungal modular PKS system from *Lasiodiplodia theobromae* through heterologous expression in *Aspergillus oryzae*, leading to the production of the antimalarial macrolide strasseriolide C (**1**). Intriguingly, biosynthesis of **1** involves *C*‐methylation of the starter malonyl unit despite the absence of a methyltransferase domain in the loading module. Furthermore, module 2 of the PKS system contains an unusual arrangement of four consecutive acyl carrier protein (ACP) domains. Although the catalytic basis of this methylation remains unresolved, our isotope‐labeling experiments, mutational analyses, and in vitro biochemical assays support a model in which the malonyl unit loaded onto the module 2 ACPs is transferred to the loading ACP, where starter‐unit methylation is proposed to occur prior to chain initiation. Our study reveals unexpected biosynthetic logic and establishes a foundation for mechanistic studies and engineering of eukaryotic modular PKSs.

## Graphical abstract

Fungal modular polyketide synthases (PKSs) remain largely unexplored. Functional characterization of the LasC/LasD assembly line established its role in strasseriolide C biosynthesis and suggested an unexpected strategy for chain initiation, in which a malonyl unit is transferred from the module 2 acyl carrier protein (ACP) domains to the loading ACP and methylated by an unidentified methyltransferase before polyketide assembly.

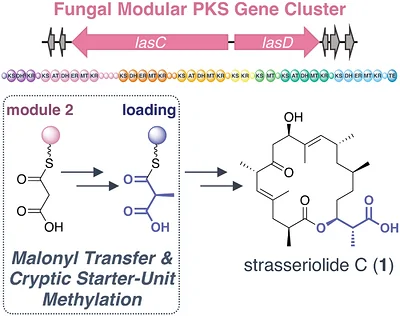

## 1 Introduction

Polyketides, generated through the repeated condensation of C_2_ acetate units, constitute a major class of natural products and have provided numerous pharmaceutical agents and other functional molecules [1, 2]. They are biosynthesized by a wide range of organisms, including bacteria, fungi, plants, protists, and even animals. Nature employs several distinct types of polyketide synthases (PKSs) to construct the diverse scaffolds of polyketides. Many bacterial polyketides are assembled by modular type I PKSs, which consist of multiple modules composed of discrete catalytic domains [3, 4]. Decades of studies on these systems have elucidated their assembly logic and enabled their rational engineering [5]. In contrast, eukaryotic modular PKSs remain largely unexplored. Although genomic and transcriptomic analyses have revealed their widespread presence, and numerous eukaryotic natural products are presumed to arise from modular PKSs, the enzymatic functions and products of these enzymes are mostly unknown [6]. A representative example is TgPKS2 from the apicomplexan parasite *Toxoplasma gondii*, which has been subjected to in vitro biochemical characterization, yet its product has not been identified [7, 8, 9]. Furthermore, modular PKSs have been identified in algae [10, 11, 12, 13]; however, most have not been explicitly linked to corresponding metabolites. Notably, giant modular PKSs designated PKZILLAs from the harmful haptophyte algae *Prymnesium parvum* have been implicated in prymnesin biosynthesis, and their domain architecture is consistent with the structural features of prymnesins [14]. Nevertheless, direct experimental characterization of these enzymes, either in vivo or in vitro, has not yet been achieved.

Fungi are a major eukaryotic lineage and prolific producers of structurally diverse and biologically active polyketides, exemplified by the cholesterol‐lowering agent, lovastatin, and the mycotoxin, aflatoxin B_1_ [15]. Although several type III PKSs have been characterized from fungi [16], the majority of fungal polyketides are biosynthesized by iterative type I PKSs, in which a single multidomain module operates repeatedly to assemble the polyketide backbone [17, 18, 19]. In 2019, modular type I PKS genes were identified in phytopathogenic fungi of the genus *Eutiarosporella*, representing the first report of modular PKSs in fungi [20]. However, the products of these modular PKS gene clusters remain unknown, and no fungal metabolites have been experimentally linked to modular PKSs. Given the remarkable rarity of modular PKSs in fungi, elucidating their functions would substantially advance our understanding of polyketide biosynthesis, reveal whether biosynthetic principles established for bacterial modular PKSs extend to fungal systems, and facilitate future engineering efforts to access structurally diverse analogues.

Herein, we report the functional characterization of a modular PKS system comprising LasC and LasD from the fungus *Lasiodiplodia theobromae* through heterologous expression, isotope‐incorporation studies, systematic mutational analyses, and in vitro biochemical assays. We reveal that LasC and LasD together produce the macrolide strasseriolide C (**1**) and that biosynthesis of **1** involves a noncanonical starter‐unit methylation event. Our study provides the first experimental characterization of a fungal modular PKS system and offers new insights into the catalytic logic and stereochemical programming of fungal modular PKSs, thereby paving the way for future engineering of these assembly lines to afford diverse polyketides.

## 2 Results and Discussion

### 2.1 Identification and Heterologous Reconstitution of a Fungal Modular PKS Gene Cluster

To experimentally characterize a fungal modular PKS system, we focused on the fungus *Lasiodiplodia theobromae* (*L. hormozganensis*) CBS 339.90, which harbors a modular PKS gene cluster designated as the *las* cluster (Figure 1A and Tables S1 and S2). The two PKSs encoded by this cluster are highly homologous to those reported in the earlier study [20] (Figure S1), and comparison with the genome of *Macrophomina phaseolina* MS6 enabled us to predict the cluster boundaries. In addition to the two modular PKSs, LasC and LasD, the *las* cluster also encodes the transcription factor (TF) LasA, the α/β hydrolase (ABH) LasB, the mitochondrial phosphate carrier (mPiC) protein LasE, the cytochrome P450 monooxygenase LasF, and the major facilitator superfamily (MFS) transporter LasG.

**FIGURE 1:**
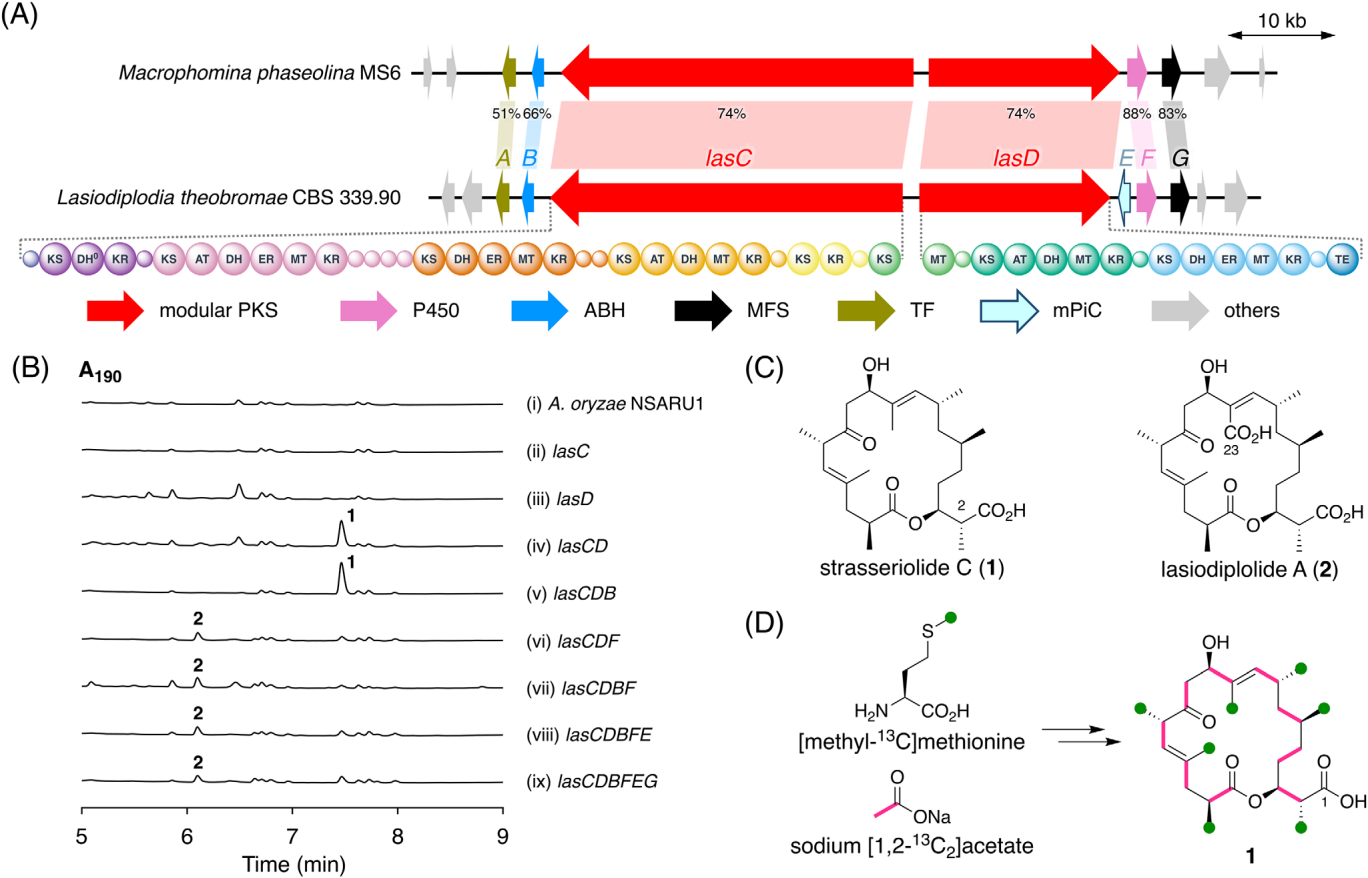
Identification and characterization of a fungal modular PKS gene cluster. (A) Schematic representation of the *las* cluster and comparison with a homologous gene cluster from *Macrophomina phaseolina*. Sequence identities of the gene products are indicated. KS, ketosynthase; AT, acyltransferase; KR, ketoreductase; DH, dehydratase; ER, enoylreductase; MT, methyltransferase; TE, thioesterase. Acyl carrier protein (ACP) domains are depicted as small unlabeled circles. (B) HPLC profiles of metabolites produced by *A. oryzae* transformants. (C) Structures of compounds **1** and **2**. (D) Summary of the isotope incorporation experiments.

As only LasD possesses a chain‐releasing thioesterase (TE) domain, we hypothesized that the two modular PKSs, LasC and LasD, work together to yield a polyketide product. To obtain this product, we performed heterologous expression in *Aspergillus oryzae* NSARU1 [21]. Because *lasC* is considerably long (ca. 32.6 kb), the gene was split into two fragments and introduced by CRISPR–Cas9‐mediated in vivo recombination as established in our earlier study [22] (Figure S2). Expression of either *lasC* or *lasD* alone in *A. oryzae* did not yield any additional metabolites compared with the host strain (Figure 1B, traces i–iii). As expected, co‐expression of the two PKS genes produced a major metabolite **1** (Figure 1B, trace iv, and Figure S3), whose molecular formula was determined to be C_26_H_42_O_6_. Comparison of the NMR spectra of **1** with literature data [23, 24] revealed that **1** is identical to strasseriolide C (Figure 1C and Tables S3 and S4), an antimalarial macrolide originally isolated from the fungus *Strasseria geniculata* [23].

Next, we sought to identify downstream metabolites of **1** by characterizing the putative tailoring enzymes encoded in the *las* cluster. Introduction of the α/β‐hydrolase gene *lasB* into the *A. oryzae* transformant producing **1** did not lead to the production of any additional metabolites (Figure 1B, trace v). In contrast, expression of the P450 gene *lasF* resulted in the production of a new metabolite, **2** (Figure 1B, trace vi), which was identified as an oxidized derivative of **1** bearing a carboxyl group at C‐23 and was named lasiodiplolide A (Figure 1C). Co‐expression of *lasB* and *lasF*, however, did not yield any additional metabolites (Figure 1B, trace vii). Further introduction of the mPiC gene *lasE*, either alone or together with the MFS transporter gene *lasG*, also did not alter the metabolic profile (Figure 1B, traces viii and ix). Thus, the functional roles of *lasB* and *lasE* currently remain unclear.

### 2.2 Investigation of Unusual Starter‐Unit Methylation Event

The module and domain organization of LasC and LasD largely corresponds to the structure of **1** (Figure 1A). However, **1** exhibits two unusual features: it possesses a carboxyl group at the starting point of the polyketide chain, and contains a methyl group at C‐2 despite the absence of a *C*‐methyltransferase (MT) domain in the loading module. To gain insight into the biosynthetic mechanisms governed by the two modular PKSs, we conducted isotope‐feeding experiments. Feeding with sodium [1,2‐^13^C_2_]acetate resulted in ^13^C enrichment across all carbon atoms of the main polyketide chain except for C‐1 (i.e., the carboxyl group), revealing nine intact C_2_ units (Figure 1D and Table S5). Meanwhile, feeding with [methyl‐^13^C]methionine demonstrated that all seven methyl groups were labeled (Figure 1D). Thus, although the loading module lacks an MT domain, the methyl group at C‐2 must be derived from *S*‐adenosylmethionine (SAM), indicating the presence of a cryptic methyltransferase.

Given that heterologous expression of the two modular PKSs was sufficient to produce **1**, we reasoned that the enzymatic domain responsible for C‐2 methylation must reside within these proteins, rather than being supplied by a *trans*‐acting methyltransferase. We therefore hypothesized that one of the MT domains in downstream modules catalyzes the initial methylation of the starter unit in addition to its canonical role during chain elongation. To test this hypothesis, the catalytic histidine residues in all MT domains (MT2, MT3, MT4, MT6, MT7, and MT8) were individually mutated to alanine, and the resulting variants were evaluated by heterologous expression. Among the six MT variants, only the MT2 mutant (LasC‐H3660A) abolished production of **1** without yielding any detectable demethylated analogues (Figure 2A, trace i). This observation may be explained by the greater impact of early‐stage methylation on substrate recognition by downstream domains/modules. In contrast, the other five variants each produced one or two demethylated analogues (Figure 2A, traces ii–vi; Figure 2C). The MT3 variant (LasC‐H6171A) yielded **3** as the major product. Although **3** was converted into its dehydrated derivative **3′** during purification, structural analysis confirmed that it lacks the C‐8 methyl group, consistent with the predicted function of module 3. Similarly, mutation of MT4 (LasC‐H8444A) resulted in the production of **4**, which lacks the C‐10 methyl group. Mutation of the catalytic residue in MT6 (LasD‐H765A) led to the formation of two products, **5** and **6**, with **6** also observed as the predominant product of the MT7 variant (LasD‐H2898A). Compounds **5** and **6** were identified as demethylated analogues of **1** lacking the C‐14 and C‐16 methyl groups, respectively. The unexpected formation of **6** by LasD‐H765A may reflect incomplete inactivation of the MT domain or altered processing within the PKS assembly line. To address this, we also mutated Glu791, which forms the catalytic dyad [25] with His765, and constructed the double variant LasD‐H765A/E791Q, selectively producing **5** (Figure 2A, trace vii). The MT8 variant (LasD‐H4989A) afforded **7**, whose dehydrated form **7′** was isolated and characterized as lacking the C‐18 methyl group. Collectively, none of the demethylated analogues obtained herein lack the C‐2 methyl group, indicating that MT3, MT4, MT6, MT7, and MT8 are not responsible for C‐2 methylation.

**FIGURE 2:**
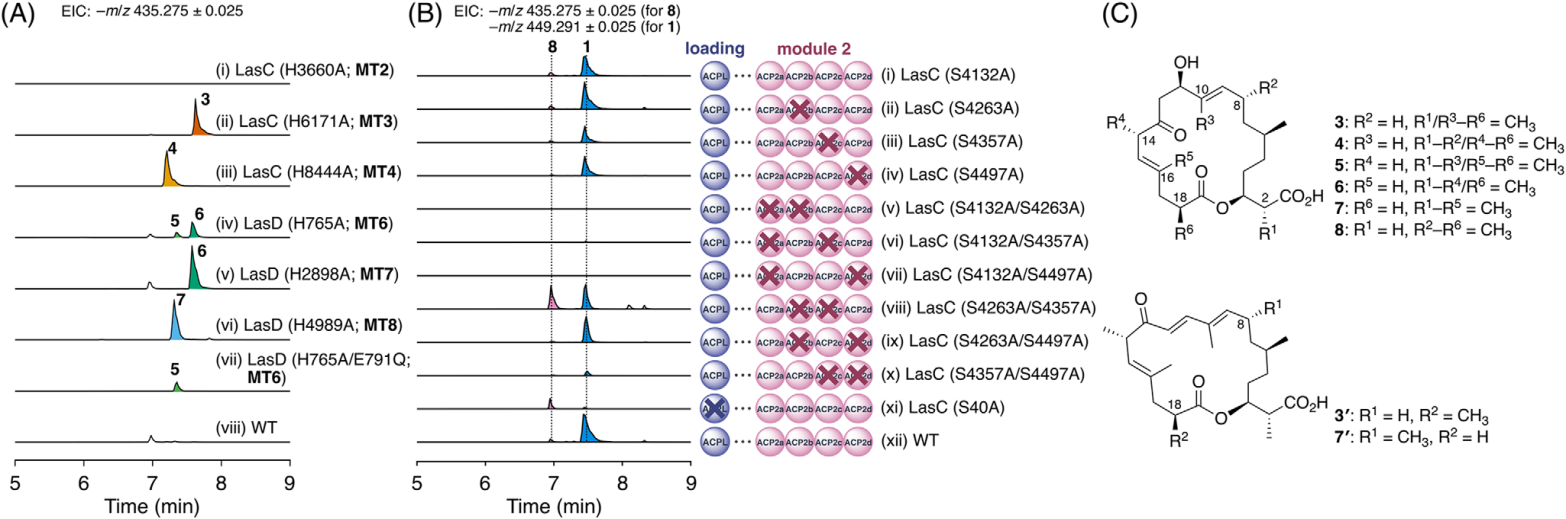
Mechanistic investigation of the noncanonical methylation. (A, B) LC–MS profiles of metabolites produced by *A. oryzae* transformants expressing (A) MT variants and (B) ACP variants of LasC or LasD, together with the corresponding wild‐type partner enzyme. (C) Structures of compounds **3–8**, **3′**, and **7′**. The structures of **3** and **7** were assigned based on their corresponding dehydrated derivatives, and the stereochemistry of **3–8**, **3′**, and **7′** was assigned by analogy to **1**, for which the absolute configuration was previously established [23].

We next focused on the possible involvement of module 2 in starter‐unit methylation. This module harbors a tandem array of four ACP domains (hereafter referred to as ACP2a–ACP2d), whose functional role cannot be readily inferred from the structure of **1**. We therefore hypothesized that these ACP domains may contribute to the unusual starter‐unit methylation process. To assess the importance of the four ACP domains, the serine residues serving as phosphopantetheine (Ppant) attachment sites in each ACP (Ser4132, Ser4263, Ser4357, and Ser4497) were individually mutated to alanine. The resulting four single variants exhibited product profiles similar to that of the wild‐type enzyme (Figure 2B, traces i–iv). We then constructed six double variants in which two of the four Ppant attachment sites were inactivated (Figure 2B, traces v–x). Intriguingly, one of these variants, in which ACP2b and ACP2c were inactivated, produced not only **1** but also an additional major product, **8**, which was identified as the C‐2 desmethyl analogue of **1** (Figure 2C). In contrast, the other five variants either almost completely lost product formation or displayed profiles similar to that of the wild type. ACP2b and ACP2c share 46% sequence identity, the highest among the ACP domains in LasC and LasD (Table S6), consistent with apparent redundancy. We also investigated the importance of the loading ACP domain (ACPL). Surprisingly, inactivation of the Ppant attachment site of this ACP (Ser40) almost completely abolished the production of **1**, with the C‐2 demethylated analogue **8** becoming the predominant product (Figure 2B, trace xi). Together, these results suggest that efficient C‐2 methylation requires both ACPL and the quartet of ACP domains in module 2, particularly ACP2b and ACP2c.

The involvement of both the module 2 ACPs and the ACPL prompted us to reconsider the mechanism of starter‐unit methylation. Given the absence of both an AT domain in the loading module and a *trans*‐AT, we hypothesized that the malonyl unit is initially loaded onto the ACP domains of module 2 and subsequently transferred to ACPL, where starter‐unit methylation would occur. To test this hypothesis, we sought to individually express and purify ACPL and the four ACP domains of module 2 from *Escherichia coli* for in vitro assays. However, ACP2b and ACP2c were largely insoluble and could not be obtained in sufficient quantities for biochemical characterization. ACPL was first converted to the holo form using CoA and the promiscuous phosphopantetheinyl transferase Sfp [26]. ACP2a and ACP2d were then incubated with malonyl‐CoA and Sfp to generate the corresponding malonyl‐ACP species. LC–MS analysis revealed malonyl transfer from ACP2a and ACP2d to ACPL (Figure 3). In contrast, no malonyl transfer was detected from ACPL to either ACP2a or ACP2d (Figure 3). Interestingly, no detectable methylmalonyl transfer was observed either from or to ACP2a or ACP2d (Figure S4). Although chain transfer involving ACP2b or ACP2c remains to be examined, the observed directional malonyl transfer from ACP2a and ACP2d supports our proposed model for substrate loading, in which ACP‐to‐ACP transfer occurs prior to starter‐unit methylation. Furthermore, neither malonyl‐ACP2a nor malonyl‐ACP2d underwent methylation when incubated with the purified MT2 and SAM (Figure S5), whereas both acetoacetyl‐ACP2a and acetoacetyl‐ACP2d, used as shortened mimics of the elongating polyketide chain, were methylated under the same reaction conditions (Figure S6). These observations further suggest that, although the isolated MT2 retains methyltransferase activity, it does not methylate malonyl‐ACP2 species.

**FIGURE 3:**
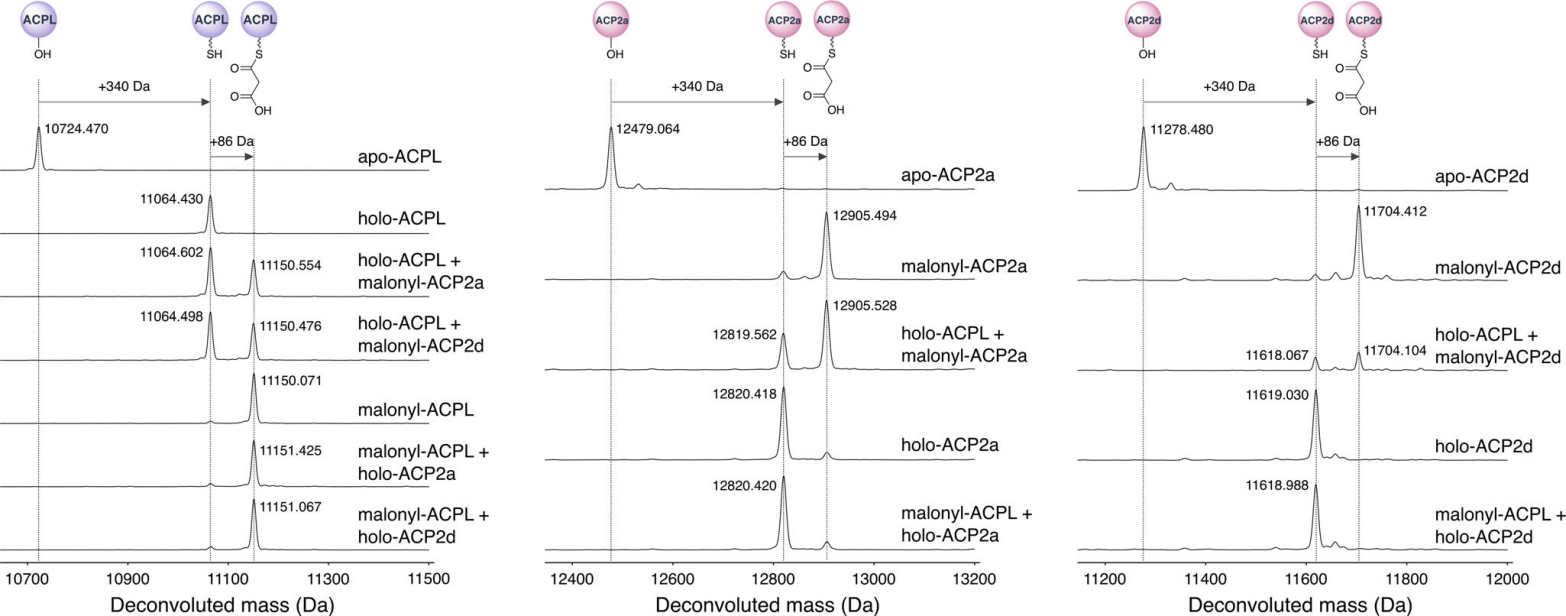
Investigation of malonyl transfer between ACP domains. Deconvoluted mass spectra of ACP species following incubation of holo‐loading ACP (ACPL) with malonyl‐ACP2a or malonyl‐ACP2d, or malonyl‐ACPL with holo‐ACP2a or holo‐ACP2d.

Given that the mutational experiments suggested that none of the MT domains except MT2 is involved in C‐2 methylation, MT2 remained the most likely candidate for this reaction. We thus incubated malonyl‐ACPL with MT2 and SAM; however, no methylation was detected (Figure S7). Notably, unlike the module 2 ACPs, even acetoacetyl‐ACPL did not undergo methylation by MT2 (Figure S6), suggesting that MT2 preferentially recognizes its cognate ACPs. Moreover, as expected, reactions with malonyl‐ACPL and each of the remaining MT domains in LasC/LasD also did not yield a methylated product (Figure S7). These observations indicate that starter‐unit methylation cannot be reconstituted using the isolated ACP and MT domains alone and may require additional molecular determinants, such as higher‐order protein–protein interactions or an as‐yet‐unidentified catalytic element.

### 2.3 Biosynthetic Programming and Stereochemical Logic of the LasC/LasD Assembly Line

Based on the available experimental data, we propose a working model for the biosynthesis of strasseriolide C (**1**) by LasC and LasD (Figure 4A). The acyltransferase (AT) domain in module 2 is proposed to accept malonyl‐CoA and load the malonyl unit onto one of the ACP domains within the module. Our mutational analyses together with the in vitro malonyl transfer assays suggest that this malonyl unit is subsequently transferred to ACPL. While methylation of malonyl‐ACP is rare in polyketide biosynthesis and is less chemically favorable than that of typical β‐ketoacyl substrates due to the higher pKa of the α‐protons, the MT domain in module 8 of the epothilone PKS system [27] and metal‐dependent MT domains, including AprA MT1 involved in apratoxin A biosynthesis [28], are known to perform malonyl‐ACP methylation. However, our present study suggests that a typical MT domain may not be involved in this methylation, and no AprA MT1‐like domain was identifiable in LasC or LasD. Thus, the mechanism for starter‐unit methylation remains to be elucidated. Nevertheless, selective production of the C‐2 demethylated analogue **8** upon inactivation of ACPL supports a model in which starter‐unit methylation occurs on the malonyl unit bound to ACPL prior to chain initiation. This model is also consistent with the established catalytic logic of PKS MT domains, which generally methylate the α‐carbon of β‐ketoacyl intermediates rather than more distal carbons of an elongated polyketide chain.

**FIGURE 4:**
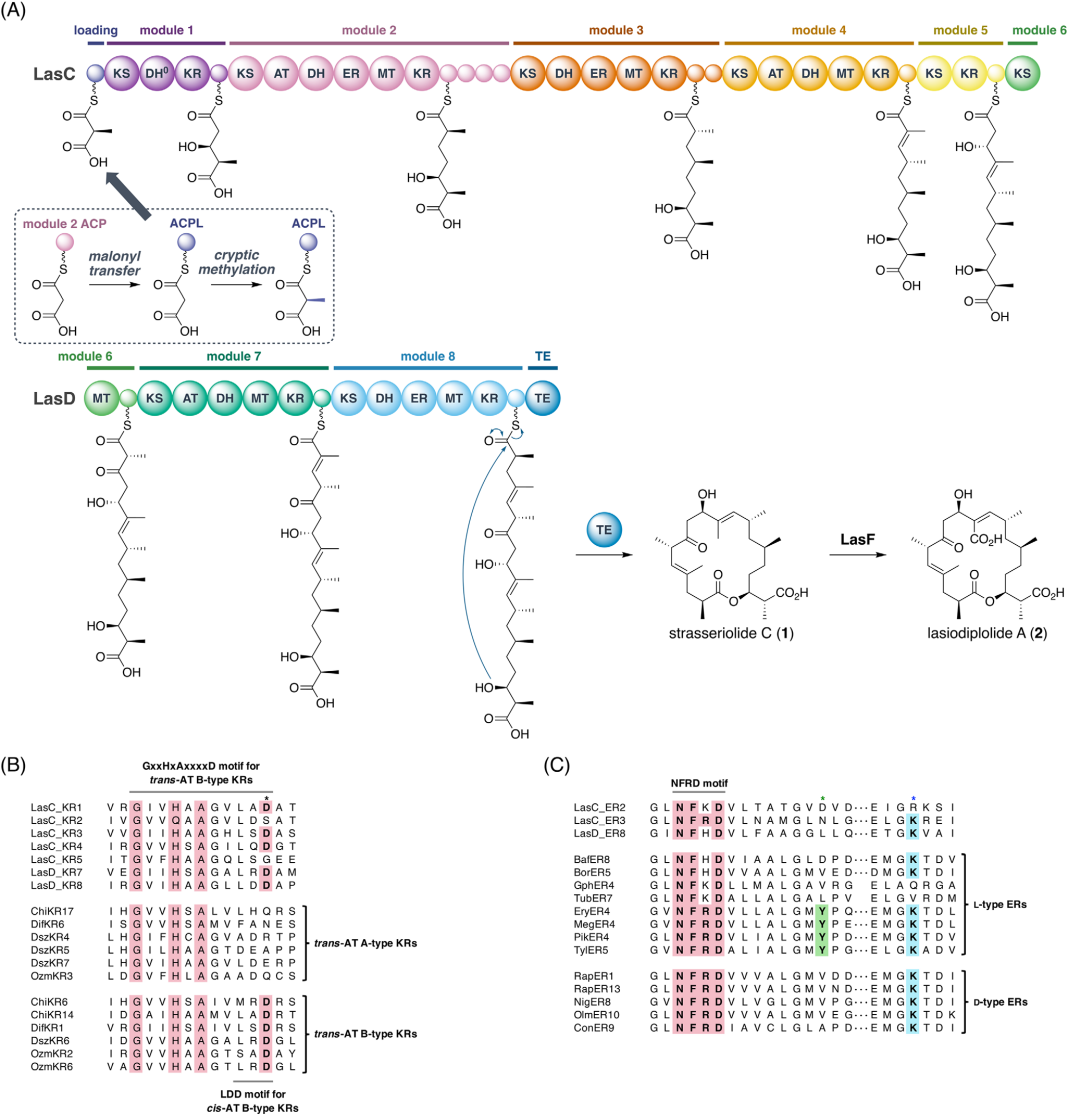
Biosynthetic model and sequence determinants of stereochemical control in the LasC/LasD system. (A) Proposed biosynthetic pathway for strasseriolide C (**1**) and its oxidation to lasiodiplolide A (**2**). The pathway features a noncanonical methylation of the starter malonyl unit. (B) Sequence alignment of the KR domains from LasC and LasD and representative bacterial *trans*‐AT PKSs. The asterisk indicates the conserved aspartate residue diagnostic of B‐type KRs. (C) Sequence alignment of the ER domains from LasC and LasD and representative bacterial PKSs. The green and blue asterisks indicate residues proposed to function as proton donors in l‐ and d‐type ERs, respectively.

The resulting methylated starter unit would then be transferred to the ketosynthase (KS) domain of module 1 (KS1) to initiate polyketide assembly. Notably, the starter‐unit carboxyl group is retained throughout chain initiation and ultimately incorporated into **1**. This feature contrasts with the more common processing of malonyl‐derived units in PKS systems, where the carboxyl group is frequently removed or otherwise modified prior to condensation [29, 30, 31]. Nevertheless, precedents exist in which carboxyacyl starter units are directly accepted by KS domains [32, 33]. These examples demonstrate that a starter unit bearing a free carboxyl group can be accommodated by a KS domain and undergo subsequent chain extension. Thus, retention of the C‐1 carboxyl group in the LasC/LasD system is mechanistically plausible, although the molecular basis by which KS1 accommodates and retains this carboxyl group remains to be elucidated. A separate notable feature of the LasC/LasD system is that only modules 2, 4, and 7 harbor embedded AT domains. AT‐less modules are common in modular PKSs and are typically complemented by *trans*‐acting ATs [34]; however, no such *trans*‐AT appears to participate in the biosynthesis of **1**. Our observation of malonyl transfer from module 2 to ACPL raises the possibility that analogous ACP‐to‐ACP transfer events may also supply extender units to the ACP domains of other AT‐less modules. The remaining chain elongation and tailoring reactions are largely consistent with the domain organization of LasC and LasD, with macrocyclization by the terminal thioesterase (TE) domain ultimately releasing **1**.

Another intriguing feature of the LasC/LasD system is the presence of four consecutive ACP domains. Tandem ACP domains have been proposed to function either “in parallel,” thereby enhancing catalytic throughput [35, 36], or “in series,” enabling sequential processing or facilitating interactions with distinct catalytic domains [37, 38, 39, 40]. In addition, our recent study on a fungal iterative PKS demonstrated that mutation of either ACP within a tandem pair altered the methylation pattern, leading to the formation of a hypermethylated product [41]. Collectively, these findings indicate that tandem ACP domains can influence not only catalytic efficiency but also biosynthetic outcomes. Our present study suggests an additional functional role for tandem ACP domains, in which the quartet of ACPs in module 2 contributes to efficient starter‐unit loading and methylation. The production of the C‐2 demethylated analogue **8** upon inactivation of specific ACP domains further highlights the functional importance of these tandem ACPs, although the precise mechanistic basis for their cooperation remains to be elucidated.

It is well established that the stereochemical outcomes of KR‐ and ER‐catalyzed reductions in bacterial modular PKSs can often be predicted from characteristic sequence motifs. To examine whether similar sequence–function relationships apply to fungal modular PKSs, we analyzed the KR and ER domains of LasC and LasD and compared them with those of characterized bacterial PKSs (Figure 4B,C). The KR domains of modules 1, 3, 4, 7, and 8 (KR1, KR3, KR4, KR7, and KR8) contain the conserved aspartate residue within the diagnostic GxxHxAxxxxD motif, which is characteristic of B‐type KRs and is associated with the formation of d‐β‐hydroxyl groups (Figure 4B) [42]. In contrast, the KR domains of modules 2 and 5 (KR2 and KR5) lack this motif and are therefore predicted to belong to the A‐type family, which generates l‐β‐hydroxyl groups (Figure 4B). These predictions are consistent with the stereochemical requirements inferred from the biosynthesis of strasseriolide C. In particular, the KR1 and KR5 are predicted to generate the stereochemistry observed in the final product, while KR4 and KR7 are expected to furnish d‐β‐hydroxyl intermediates that subsequently undergo dehydration to form *trans*‐double bonds, a transformation generally associated with B‐type KR products [43]. It is noteworthy that the GxxHxAxxxxD motif has primarily been established as a diagnostic feature of KR domains from bacterial *trans*‐AT PKSs [42], whereas B‐type KRs from *cis*‐AT systems typically contain the LDD motif in the corresponding region [44]. In the LasC/LasD system, the LDD motif is present only in KR8, suggesting that fungal modular PKSs share sequence features with KR domains from bacterial *trans*‐AT PKSs.

Meanwhile, the stereoselectivity of ER domains in modular PKSs has been proposed to depend on the presence of a signature tyrosine residue within the α1–β4 loop, which is associated with l‐type ERs [45]. Surprisingly, none of the ER domains in LasC or LasD contain this tyrosine residue, which would classify all of them as d‐type ERs according to the current sequence‐based model (Figure 4C). However, the ER domains of modules 2 and 8 (ER2 and ER8) are expected to generate l‐configured methyl branches, as indicated by the structure of **1**. Further sequence analysis revealed that the conserved NFRD motif is altered in ER2 and ER8, yielding NFKD and NFHD motifs, respectively. Notably, Arg‐to‐His substitutions have previously been observed in several l‐type ERs lacking the signature tyrosine residue, where histidine has been proposed to participate in protonation during enoyl reduction [45]. The Arg‐to‐Lys substitution observed in ER2 may likewise contribute to an alternative mechanism of stereochemical control. In addition, the conserved lysine residue proposed to act as the proton donor in d‐type ERs is replaced by arginine in ER2. Supporting this hypothesis, the NFKD motif is also found in the module 4 ER from the gephyronic acid PKS system (GphER4) and in the module 7 ER from the tubulysin PKS system (TubER7) [46, 47]. Based on the structures of the corresponding products, both ERs are expected to generate l‐configured methyl branches despite lacking the canonical tyrosine residue associated with l‐type ERs. Furthermore, both ERs also lack the otherwise highly conserved lysine residue, mirroring the unusual sequence features observed in ER2. The recurrence of these sequence features in multiple evolutionarily distinct PKSs further supports the assignment of ER2 as an l‐type ER and suggests that the NFKD motif may be associated with l‐type stereoselectivity, although this hypothesis remains to be experimentally validated.

BLASTp searches against the NCBI ClusteredNR database using LasC/LasD sequences as queries revealed that all currently identifiable fungal modular PKSs are closely related to LasC or LasD (Figures S8 and S9). The remaining hits were derived from apicomplexans, dinoflagellates, and bacteria, consistent with phylogenetic analysis showing that KS domains of fungal modular PKSs form a clade with those of bacterial and protist origin [20]. However, these non‐fungal modular PKSs exhibit only limited sequence and modular similarity to LasC or LasD, suggesting that fungal modular PKSs have diverged substantially following their emergence. Nevertheless, the agreement between the predicted stereochemical outcomes of the KR and ER domains and the structure of **1** indicates that key sequence determinants governing stereochemical control remain conserved. Thus, sequence‐based prediction of stereoselectivity can be extended to fungal modular PKSs, providing a framework for their future functional annotation and engineering to access stereochemically diversified analogues.

## 3 Conclusion

In summary, we report the first experimental characterization of a fungal modular PKS system and uncover a noncanonical starter‐unit methylation event. Our genetic and biochemical analyses support a model in which a malonyl unit is transferred from the ACP domains of module 2 to ACPL, where starter‐unit methylation is proposed to occur prior to chain elongation. Furthermore, our study provides insights into the programming principles of fungal modular PKSs, which may facilitate their mechanistic investigation and future engineering. Future studies will focus on elucidating the molecular basis of starter‐unit methylation and substrate loading in this unusual assembly line through biochemical and structural analyses. In addition, strasseriolides have attracted considerable attention as targets for total synthesis owing to their potent antiparasitic activities [24, 48, 49, 50, 51]. Our biosynthetic platform, which yields multiple strasseriolide analogues, provides a complementary approach to accessing this important class of natural products.

## Author Contributions

**Qiaolin Ji**: investigation, visualization, methodology, writing – original draft, formal analysis, data curation. **Chao Peng**: investigation, visualization, methodology, writing – original draft, formal analysis, data curation. **Shuhan He**: investigation. **Bin‐Tao Peng**: investigation. **Wei‐Guang Wang**: conceptualization, supervision, funding acquisition, visualization, methodology, writing – original draft, writing – review and editing, project administration, formal analysis. **Yudai Matsuda**: conceptualization, supervision, funding acquisition, visualization, methodology, writing – original draft, writing – review and editing, project administration, formal analysis.

## Conflicts of Interest

The authors declare no conflicts of interest.

## Supporting information




**Supporting File**: anie75136‐sup‐0001‐SuppMat.pdf.

## Data Availability

The data that support the findings of this study are available in the Supporting Information of this article.
