# Supplementary material for: Characterization of a Fungal Modular Polyketide Synthase System Reveals an Unusual Strategy for Polyketide Chain Initiation
Source: Angew Chem Int Ed Engl. 2026 Sep 30;65(46):e7344670. doi: 10.1002/anie.7344670 (PMC13749307; doi:10.1002/anie.7344670)
Supplement: Supplementary file 1 [file ANIE-65-e7344670-s001.pdf]

Table of Contents

Supplementary Materials and Methods **S2–S8**

Supplementary Tables S1–S9 **S9–S26**

Supplementary Figures S1–S64 **S27–S84**

Supplementary References **S85–S86**

Supplementary Materials and Methods

General experimental procedures

Organic solvents were purchased from Anaqua (Hong Kong) Co. Ltd., Anhui Tedia High Purity Solvents Co., Ltd., and Duksan Pure Chemicals Co. Ltd., and other chemicals were purchased from Wako Chemicals Ltd., Thermo Fisher Scientific, Sigma-Aldrich, J&K Scientific Ltd., or Merck KGaA Ltd., unless noted otherwise. Oligonucleotide primers (Table S7) were purchased from Beijing Genomics Institute or Tsingke. PCR was performed using a Bio-Gener Thermo Cycler (Bio-Gener Technology Co., Ltd.) or a T30 Thermal Cycler (LongGene) with Phanta Max Super-Fidelity DNA Polymerase (Vazyme Biotech Co., Ltd.). Preparative HPLC was performed on a Waters 1525 Binary HPLC pump with a 2998 photodiode array detector (Waters Corporation). Flash chromatography was performed using an Isolera Spektra One flash purification system (Biotage). NMR spectra were obtained at 600 MHz (^{1}H)/150 MHz (^{13}C) with a Bruker Ascend Avance III HD spectrometer, and chemical shifts were recorded with reference to solvent signals (^{1}H NMR: $CDCl_3$ 7.26 ppm, CD_3OD 3.31 ppm; ^{13}C NMR: $CDCl_3$ 77.0 ppm, CD_3OD 49.0 ppm). HR-ESI-MS spectra were obtained with a SCIEX X500R Q-TOF mass spectrometer. For the analysis of metabolites from *Aspergillus oryzae* transformants, samples for LC–MS analysis were injected into a SCIEX ExionLC AD System with a SCIEX X500R Q-TOF mass spectrometer, using a Kinetex C18 column (5 µm, 100 Å, 4.6 × 150 mm; Phenomenex). For protein analysis, samples were injected into an Acquity UPLC I-Class plus system equipped with a Xevo G2-XS QTof mass spectrometer, using a ZORBAX 300SB-C3 column (3.0 i.d. × 150 mm, 3.5 µm). Optical rotations were measured with a P-2000 Digital Polarimeter (JASCO Corporation).

Microbial strains

Lasiodiplodia theobromae CBS 339.90 was obtained from the Westerdijk Fungal Biodiversity Institute and used for cloning the *las* genes. *Aspergillus oryzae* NSARU1 (*niaD*$^-$, *sC*$^-$, *ΔargB, adeA*$^-$, *pyrG*$^-$)[1] was utilized as the fungal heterologous expression host. Standard DNA engineering was performed with *Escherichia coli* DH5α (Takara Bio Inc). *E. coli* Rosetta2 (DE3) (Novagen) was used for the protein expression experiments.

Construction of new fungal transformation vectors, pPenoA142h, pPgpdA1, pPubi, pPTRI-HR, and pPyrG-HS601

To facilitate the construction of fungal transformation plasmids harboring multiple genes under different promoters and terminators, several additional vectors were generated in this study.

The *Aspergillus oryzae* glyceraldehyde-3-phosphate dehydrogenase promoter (*PgpdA1*) and terminator (*TgpdA1*)[2] were amplified from genomic DNA of *A. oryzae* NSARU1 and ligated into *Sma*I-digested pUC19 to yield pPgpdA1 (Figure S10). Similarly, pPubi was constructed by inserting the ubiquitin promoter (*Pubi*) and terminator (*Tubi*)[2] into *Sma*I-digested pUC19 (Figure S11). The vector pPenoA142h was generated by inserting

PenoA142[3] fused to the 5′ untranslated region of the *Hsp12* gene,[4] together with the *A. oryzae pgkA* terminator (~300 bp of the 3′ flanking region of AO090038000395; GenBank: BAE64188.1), into the *Sma*I-digested pUC19 backbone (Figure S12). These fragments were amplified from the pTAex3A[5] vector and genomic DNA of *A. oryzae* NSARU1, respectively.

A homologous recombination-based integration vector, pPTRI-HR (Figure S13), was constructed as a derivative of pPTRI[6] targeting the HS601 locus of *A. oryzae*.[7] The upstream and downstream regions of the target site (~1 kb each) were amplified from genomic DNA of *A. oryzae* NSAR1[8] and inserted into a PCR-linearized pPTRI vector such that the *ptrA* marker was flanked by these regions. For *pyrG*-based selection, the flanking regions were subsequently transferred to a new construct and ligated with a *pyrG* marker amplified from pPyrG,[1] yielding pPyrG-HS601 (Figure S14).

Primer sequences and detailed construction procedures are provided in Tables S7 and S8. DNA fragment assembly was performed using the ClonExpress Ultra One Step Cloning Kit (Vazyme Biotech Co., Ltd).

Construction of fungal transformation plasmids

To construct fungal expression plasmids for *A. oryzae*, genes from the *las* cluster were amplified from genomic DNA of *L. theobromae* CBS 339.90 using primers listed in Tables S7 and S8. The first and latter halves of *lasC* or its variants were introduced into two different plasmids, each containing a different selection marker, using a ClonExpress Ultra One Step Cloning Kit (Vazyme Biotech Co., Ltd). The *lasC* gene (or its variants) was divided into two overlapping fragments (~1 kb overlap), each cloned into separate plasmids carrying different selection markers using a ClonExpress Ultra One Step Cloning Kit (Vazyme). These fragments were reassembled in vivo and integrated into the chromosome of *A. oryzae* NSARU1 via CRISPR–Cas9-mediated homologous recombination.[9] In parallel, *lasD* (or its variants) was cloned into the *Sma*I-digested pPyrG-HR[1] vector. Other genes were assembled into fungal expression vectors, including newly constructed vectors (pPenoA142h, pPgpdA1, and pPubi) as well as the previously reported pTAex3[10] vector, after which promoter–terminator cassettes were transferred into the pPyrG-HS601 vector. Details of vector construction, modular assembly, and plasmid design are provided in Table S8.

Fungal transformation

Transformation of *A. oryzae* NSARU1 was performed using a previously reported protoplast–polyethylene glycol (PEG) method[11] in combination with CRISPR–Cas9-mediated homologous recombination.[1,9,12] To introduce genes encoding tailoring enzymes into *A. oryzae* strains harboring *lasC* and *lasD*, transformants were first cultivated on minimum medium [0.2% NH_4Cl, 0.2% $(NH_4)_2SO_4$, 0.05% KCl, 0.05% NaCl, 0.05% $MgSO_4 \cdot 7H_2O$, 0.1% KH_2PO_4, 0.002% $FeSO_4 \cdot 7H_2O$, 2% glucose, and 1.5% agar] supplemented with 0.15% methionine, 0.2% uracil, and 0.5% uridine. Spores were harvested in 20% glycerol and plated onto minimum medium containing 2 mg mL^{-1} 5-fluoroorotic acid (5-FOA) for marker recycling. Colonies were subjected to a

second round of selection, and isolates that had lost the *pyrG* marker were used for subsequent transformations with pPyrG-HS601-based plasmids. The transformants generated in this study and the plasmids used are listed in Table S9.

LC–MS analysis of metabolites from *Aspergillus oryzae* transformants

To analyze metabolites produced by *A. oryzae* transformants, strains were cultivated on DPY agar plates [2% dextrin, 1% hipolypepton (Nihon Pharmaceutical Co., Ltd.), 0.5% yeast extract, 0.5% KH_2PO_4, 0.05% $MgSO_4 \cdot 7H_2O$, and 1.5% agar] at 30 °C for 7 days. A small piece of fungal mycelium together with agar was excised from the plate, soaked in ethyl acetate, and extracted using an ultrasonic bath. The ethyl acetate layer was transferred to a fresh tube, and the solvent was removed under a stream of nitrogen. The resulting residue was dissolved in methanol and subjected to LC–MS analysis. LC–MS analysis was performed using a solvent system consisting of 20 mM formic acid in water (solvent A) and acetonitrile containing 20 mM formic acid (solvent B), at a flow rate of 1 mL/min and a column temperature of 40 °C. Separation was achieved using a linear gradient from 10:90 (B/A) to 100:0 over 10 min, followed by 100:0 for 3 min, a linear gradient from 100:0 to 10:90 over 2 min, and equilibration at 10:90 for 2.5 min.

Isolation of each metabolite

To isolate each metabolite, *A. oryzae* transformants were cultivated on DPY agar plates for seven days at 30 °C. The fungal cultures, together with the agar medium, were cut into small pieces, soaked in ethyl acetate, and extracted three times using an ultrasonic bath. After filtration, the ethyl acetate was removed in vacuo. The resulting crude extract was fractionated by flash chromatography using an Sfär Silica column (60 µm, 50 g; Biotage) and further purified by preparative HPLC using an XBridge BEH C18 OBD Prep column (100 Å, 5 µm, 19 mm i.d. × 250 mm; Waters Corporation). Detailed purification procedures for each compound are provided below.

Purification conditions for strasseriolide C (**1**):

The extract of *A. oryzae* strain harboring *lasC* and *lasD* (0.79 g) from 100 DPY plates (*ca.* 2 L) was subjected to flash chromatography and eluted stepwise using a dichloromethane:ethyl acetate gradient (100:0 to 0:100) containing 0.05% trifluoroacetic acid (TFA). Fractions that contained **1** were purified by reverse-phase preparative HPLC (45% aqueous acetonitrile with 0.05% TFA, 10 mL/min) to yield 28.9 mg of **1**.

Purification conditions for lasdiodiplolide A (**2**):

The extract of *A. oryzae* harboring *lasC*, *lasD*, *lasB*, *lasF*, and *lasE* (2.62 g) from 200 DPY plates (*ca.* 4 L) was subjected to flash chromatography and eluted stepwise using a dichloromethane:ethyl acetate gradient (100:0

to 0:100) containing 0.05% TFA. Fractions that contained **2** were purified by reverse-phase preparative HPLC (45% aqueous acetonitrile with 0.05% TFA, 10 mL/min) to yield 8.8 mg of **2**.

Purification conditions for compound **3′**:

The extract of *A. oryzae* harboring *lasC* (H6171A) and *lasD* (1.64 g) from 100 DPY plates (*ca.* 2 L) was subjected to flash chromatography and eluted stepwise using a dichloromethane:ethyl acetate gradient (100:0 to 0:100) containing 0.05% TFA. Fractions that contained **3** were purified by reverse-phase preparative HPLC (68% aqueous methanol with 0.05% TFA, 10 mL/min) to yield 7.8 mg of **3′**.

Purification conditions for compound **4**:

The extract of *A. oryzae* harboring *lasC* (H8444A) and *lasD* (1.23 g) from 100 DPY plates (*ca.* 2 L) was subjected to flash chromatography and eluted stepwise using a dichloromethane:ethyl acetate gradient (100:0 to 0:100) containing 0.05% TFA. Fractions that contained **4** were purified by reverse-phase preparative HPLC (45% aqueous acetonitrile with 0.05% TFA, 10 mL/min) to yield 81.5 mg of **4**.

Purification conditions for compounds **5** and **6**:

The extract of *A. oryzae* harboring *lasC* and *lasD* (H765A) (5.35 g) from 300 DPY plates (*ca.* 6 L) was subjected to flash chromatography and eluted stepwise using a dichloromethane:ethyl acetate gradient (100:0 to 0:100) containing 0.05% TFA. Fractions that contained **5** and **6** were purified by reverse-phase preparative HPLC (44% aqueous acetonitrile with 0.05% TFA, 10 mL/min) to yield 2.0 mg of **5** and 8.0 mg of **6**.

Purification conditions for compound **7′**:

The extract of *A. oryzae* harboring *lasC* and *lasD* (H4989A) (1.0 g) from 100 DPY plates (*ca.* 2 L) was subjected to flash chromatography and eluted stepwise using a dichloromethane:ethyl acetate gradient (100:0 to 0:100) containing 0.05% TFA. Fractions that contained **7** were purified by reverse-phase preparative HPLC (45% aqueous acetonitrile with 0.05% TFA, 10 mL/min) to yield 32.5 mg of a mixture containing **7′** as the major product. The mixture was further purified by reverse-phase preparative HPLC (78% aqueous acetonitrile with 0.05% TFA, 10 mL/min) to yield 13.0 mg of **7′**.

Purification conditions for compound **8**:

The extract of *A. oryzae* harboring *lasC* (S4263A/S4357A) and *lasD* (2.5 g) from 150 DPY plates (*ca.* 3 L) was subjected to flash chromatography and eluted stepwise using a dichloromethane:ethyl acetate gradient (100:0 to 0:100) containing 0.05% TFA. Fractions that contained **8** were purified by reverse-phase preparative HPLC (45% aqueous acetonitrile with 0.05% TFA, 10 mL/min) to yield 56.2 mg of **8**.

In vivo labeling experiments

For isotope incorporation experiments with [methyl-^{13}C]methionine and sodium [1,2-$^{13}C_2$]acetate, the *A. oryzae* transformant expressing *lasC* and *lasD* was cultivated at 30 °C for seven days on DPY agar plates (30 plates each) supplemented with 400 mg L^{-1} [methyl-^{13}C]methionine or 500 mg L^{-1} sodium [1,2-$^{13}C_2$]acetate, respectively. Labeled strasseriolide C was extracted and purified as described for unlabeled **1**, yielding 9.0 mg and 14.0 mg of yellowish amorphous solid from the [methyl-^{13}C]methionine- and sodium [1,2-$^{13}C_2$]acetate-fed cultures, respectively. NMR data of the labeled compounds are provided in Table S5 and Figures S20–S22.

Protein expression and purification

The coding sequences for the loading ACP (ACPL), ACP2a, ACP2d, and MT2 were amplified and ligated into the *Nco*I and *Xho*I sites of the pET-28a(+) vector, whereas those for the other MT domains, namely MT3, MT4, MT6, MT7, and MT8, were introduced into the *Nde*I- and *Eco*RI-digested pET-28a(+) vector. The pET-28a(+)-sfp plasmid was constructed in our previous study.[13]

To express the ACP and MT domains, *E. coli* Rosetta2(DE3) was transformed with the corresponding pET-28a(+)-based plasmids. Each *E. coli* transformant was incubated with shaking at 37 °C and 200 rpm in LB medium supplemented with appropriate antibiotics (50 mg/L kanamycin sulfate and 34 mg/L chloramphenicol for individual expression strains; 100 mg/L ampicillin sodium, 50 mg/L kanamycin sulfate and 34 mg/L chloramphenicol for the co-expression strain) until the OD_{600} reached 0.5–0.8, after which 0.5 mM IPTG was added to induce gene expression. After further cultivation at 16 °C and 200 rpm for 16 h, the cells were collected by centrifugation (4 °C, 8,000 rpm, 5 min), resuspended in lysis buffer (50 mM Tris-HCl, pH 7.5, 200 mM NaCl, 5 mM imidazole), and lysed on ice by sonication. Cell debris was removed by centrifugation (4 °C, 8,000 rpm, 30 min) to obtain the cell-free supernatant.

For protein purification, the cell-free supernatants were loaded onto a Ni-NTA affinity column. The resin was washed with wash buffer (50 mM Tris-HCl, pH 7.5, 200 mM NaCl, 15 mM imidazole), and the His-tagged proteins were eluted with 4 column volumes of elution buffer (50 mM Tris-HCl, pH 7.5, 200 mM NaCl, 300 mM imidazole). The protein solutions were ultrafiltrated and concentrated using a 10 kDa or 30 kDa Amicon Ultra-15 Device (Merck) with imidazole-free buffer (50 mM Tris-HCl, pH 7.5, 200 mM NaCl) three times. The purity of the enzymes was analyzed by SDS-PAGE (Figure S15), and the protein concentration was determined using a NanoDrop OneC spectrophotometer (Thermo Scientific).

LC–MS analysis of proteins

LC–MS assays of proteins were conducted using a binary solvent system comprising water containing 0.1% (v/v) formic acid (solvent A) and acetonitrile containing 0.1% (v/v) formic acid (solvent B). The operational parameters included a flow rate of 0.4 mL/min and a column thermostat set at 45 °C. Chromatographic separation was achieved via a linear gradient commencing at 10:90 (solvent B/solvent A), transitioning to 100:0

over 15 min, maintained at 100:0 for an additional 3 min, and subsequently reverting from 100:0 back to 10:90 over 2 min.

In vitro protein assays

To prepare the holo-, malonyl-, methylmalonyl-, or acetoacetyl-ACP species, 50 μM of the respective apo-ACP was incubated with 10 μM Sfp, 2 mM CoA, malonyl-CoA, methylmalonyl-CoA, or acetoacetyl-CoA, and 10 mM $MgCl_2$ in 100 μL of Tris–HCl buffer (50 mM, pH 7.5) for 30 min. The reaction mixture was then subjected to ultrafiltration using an Amicon Ultra device and washed twice with 1 mL of Tris–HCl buffer (50 mM, pH 7.5) to remove excess CoA, malonyl-CoA, or methylmalonyl-CoA.

To investigate malonyl or methylmalonyl transfer between ACP domains, 100 μL of the holo-ACP solution was mixed with 100 μL of the malonyl- or methylmalonyl-ACP solution and incubated for 30 min (final concentration of each ACP was 25 μM). After centrifugation at 12,000 rpm for 5 min to remove precipitates, the supernatant was analyzed by LC–MS.

For the methylation assays, the loading ACP was first converted to the malonyl- or acetoacetyl-ACP form as described above. The reaction mixture was then supplemented with 50 μM of an individual MT domain and 1 mM *S*-adenosyl-L-methionine (SAM), followed by incubation for 30 min. After centrifugation at 12,000 rpm for 5 min, the supernatant was analyzed by LC–MS.

Deconvolution analysis of intact proteins

The raw LC–MS data of the intact proteins were deconvoluted using UniDec software.[14] The *m*/*z* range used for data processing was restricted to 500–1500. For the deconvolution parameters, the charge state range was set between 5 and 20, and the expected mass range was defined from 10,000.0 to 14,000.0 Da with a mass sampling interval of 1.0 Da. The analysis was performed with the "Smooth Charge States Distributions" and "Use Automatic *m*/*z* Peak Width" functions enabled. The "Smooth Nearby Points" option was set to "Some," and artifact suppression was disabled. For peak selection, the peak detection range was set to 500.0 Da with a detection threshold of 0.3. Peak thresholds were normalized using a marker threshold of 0.1 and a species separation value of 0.025.

Analytical data

Strasseriolide C (1). Yellowish amorphous solid; $[\alpha]^{20}_{D}$ +155.9 (*c* 1.0, CH_3OH); for NMR data, see Tables S3 and S4 and Figures S16–S19; HRMS (ESI) *m/z*: $[M - H]^-$ Calcd for $C_{26}H_{41}O_6$ 449.2909; Found 449.2909. The NMR data are in good agreement with the reported data.[15]

Lasiodiplolide A (2). Yellowish amorphous solid; $[\alpha]^{20}_{D}$ +203.9 (*c* 0.7, CH_3OH); for NMR data, see Figures S23–S28; HRMS (ESI) *m/z*: $[M - H]^-$ Calcd for $C_{26}H_{39}O_8$ 479.2650; Found 479.2628.

Compound 3′. Yellowish amorphous solid; for NMR data, see Figures S29–S34; HRMS (ESI) *m/z*: $[M - H]^-$ Calcd for $C_{25}H_{37}O_5$ 417.2647; Found 417.2646.

Compound 4. Yellowish amorphous solid; for NMR data, see Figures S35–S40; HRMS (ESI) *m/z*: $[M - H]^-$ Calcd for $C_{25}H_{39}O_6$ 435.2752; Found 435.2740.

Compound 5. Yellowish amorphous solid; for NMR data, see Figures S41–S46; HRMS (ESI) *m/z*: $[M - H]^-$ Calcd for $C_{25}H_{39}O_6$ 435.2752; Found 435.2742.

Compound 6. Colorless amorphous solid; for NMR data, see Figures S47–S52; HRMS (ESI) *m/z*: $[M - H]^-$ Calcd for $C_{25}H_{39}O_6$ 435.2752; Found 435.2738.

Compound 7′. Yellowish amorphous solid; for NMR data, see Figures S53–S58; HRMS (ESI) *m/z*: $[M - H]^-$ Calcd for $C_{25}H_{37}O_5$ 417.2647; Found 417.2635.

Compound 8. Colorless amorphous solid; for NMR data, see Figures S59–S64; HRMS (ESI) *m/z*: $[M - H]^-$ Calcd for $C_{25}H_{39}O_6$ 435.2752; Found 435.2752.

Table S1. Annotation of each gene in the *las* cluster from *Lasiodiplodia theobromae* CBS 339.90 (GenBank accession: JAUJDW010000003.1; nucleotide positions 192,728–256,413).

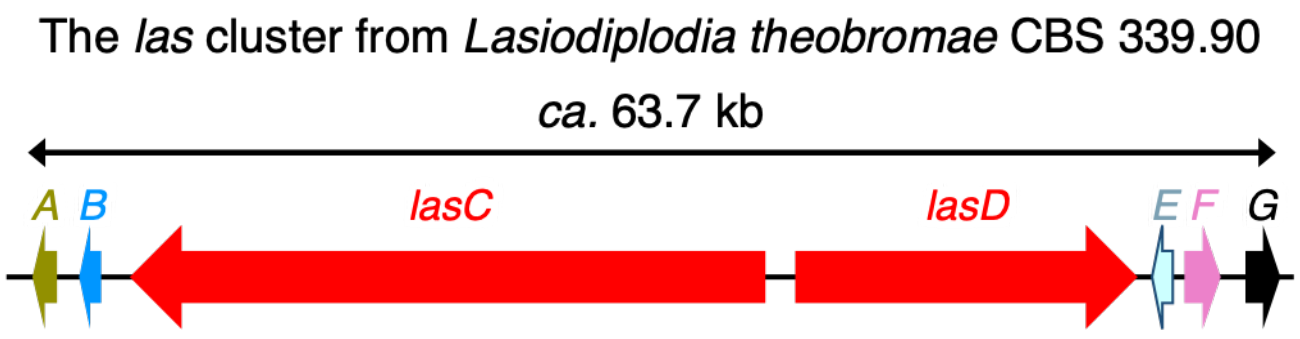

Gene	Amino acids (base pairs)	Proposed function
lasA	419 (1309)	transcription factor
lasB	402 (1209)	α/β hydrolase
lasC	10876 (32631)	modular polyketide synthase
lasD	5815 (17448)	modular polyketide synthase
lasE	313 (1204)	mitochondrial phosphate carrier protein
lasF	496 (1869)	cytochrome P450 monooxygenase
lasG	514 (1708)	major facilitator superfamily transporter

Table S2. Sequences of the *las* genes and their products. Highlighted in magenta are the regions predicted for an intron in this study.

Gene	DNA sequence	Protein sequence
lasA	atggagatggcttcacctacgaccaagtctcgccccgtcgaatcgcagctacgagggattagaaagaagcggatggcttgtgatgtctgccacggctcaaaggttc<br>gctgcagtggtggcacgccctgcaacacctgcaacaggctcggcaccgattgcgtctacagcttttccagcgtactcggaaggccgaagaatcgcaggacaaag<br>aagatgggagtggcacgtgaaggaaaggcggcggcaaagtcaacaacgacggatcaagccaaagatgccaccaaggattccaacgaccaagaccatgca<br>catatcccggacatggacatttccggcttttaccctggtgtgtaattcaacattcacacaattttcactcactaatctcgcaacagccgaacacaattactacgacgaca<br>ttccgctctgggagtcgtactcttgtctggattctgacccgacacaccttctttgcagcaacgtcgaacatgtccacatcaggccggaagacgctgtgacaagtatttcc<br>ccagagattcctccagcgagtctccccgaatcgaatgatgcggagtatgtcgcaggtctacaaccgctttccaggagcctttctcccgaaatgaacgccgaaatacc<br>gcagctcttgtgcgattgcttgaagcgttgcgcagtggtgctgtctgggttgaaggcgctggagatggatgtacagttccagatcgatgcggcgctgctacacatccgc<br>aaagccacagccacctggaagcaggtgttgcagtgtagttgctgcgagagaggaaatcctgaggaagccgtgtacatggccatcagcggtgtgagagcagcttt<br>acgctatctcgtggctgtcttcggcaacgagattgagaaagccactgatacatcatctggcagcctttattccgatccgtcccagagtctgcccactccacaggaaat<br>gccacctcaagaggactatcttgcgtcctctaagcacgtgagcagcgcaatcggaaagtcgaacttgggaggctacgacatggatggagaagagcaggaactc<br>gtcgtaaaattcattgcgctacgattggttcgcgagatgacggatgtcctgaaaatgttggaagagcgagttaataaacaacgaactccattgttctggcctgatgcgg<br>cactaactcaacaaccggccgcctggagtaacgatttatttcatctccaacccctactgcagggcgttaaggagacagccaaatcattacaggaaagcctgtgcaa<br>ctcaagtacagccgagcttcatcggtatatatga	MEMASPTTKSRPVESQLRGIRKKRMACDVCHGSK<br>VRCSGGTPCNTCNRLGTDCVYSFSSVLGRPKNRR<br>TKKMGVAREGKAAAKSTTTDQAKDATKDSNDQDH<br>AHIPDMDISGFYPAEHNYYDDIPLWESYSCLDSDPT<br>HLLCSNVEHVHIRPEDAVTSISPEIPPASLPESNDAE<br>YVAGLQPLSRSLSPEMNAEIPQLLCDCLKRCAVVLS<br>GLKALEMDVQFQIDAALLHIRKATATWKQVLQCSCC<br>ERGNPEEAVYMAISGVRAALRYLVAVFGNEIEKATD<br>TSSGSLYSDPSQSLPTPQEMPPQEDYLASSKHVSS<br>AIGKSNLGGYDMDGEEQELVVKFIALRLVREMTDVL<br>KMLEERVNKQRTPLFWPDAALTQQPAAWSNDLFH<br>LQPLLQGVKETAKSLQESLCNSSTAELHRYI*
lasB	atggcctcgacagaccgcacggccaagatccgcatcgtctgcctccacggcttcagctgcaacagcgccatctatcggttccagacggcccggctacgcaagcg<br>ctgggcggagcacctgcgccaacacccactagtagcagcaacgtcctccgcctccccatcgagtacctcctcctctcgagccgcgagtcccggcctcgacgatcc<br>ctggcaacgaagtaacactcaatactactacgacagcgatgaggagagcagtagcgccgccaccaccaccagctcgtcctcctcctcagtcgatctcgcggcat<br>caatcagcagccgaagcagcatcagtttcggcagcgactatcctcctccgccaagctacagcagcgggtccagcggcggcaacagcggcggcatcgtcgtgta<br>caacgacgtcgagttcatcttcctcgagtcgccacgcgaggtctcggccccggagcagccgctagcagaggtggcctcgctctttcccggaccgtaccggacgtg<br>gttcgagccaagcacaacaacaacaacaacaacaacaacaacaacgagaagaacagcggaaacagccaagccggacgacggaagccgcgcgcgccg<br>gcgcgacccggcaatcgagcggtgggtggtggagcaggtcaagcgcgcgggcggcgtgcacggggtgctggcgttcagcgacggcgcggcgctgacaaca<br>gcactaacgctcaaggccgaggcgggcgtcgcggcgtgcccgaagctgtgggactttgtcgtgctggcgtgcggtgtcgacccgtatccggatccttggctgcatg<br>gcagtggtagtaacggtggtattgcggatggggaggcgaaggaggtggaggaagaggcgaagtgtttggcacggttgaagacgcccggtctgcatatcgttgggt<br>tgcgagatgaggtctacagagatcgtagcgaggcgttgtggggcctcgtgcatccgcaggcggcggcggctgctgctgaccgcgacgagaagggaatgaatgct<br>gcttcgggcatggcggccgaggtctatcgcttcgatgatggccacaaggtgcccgttcaagacactgacttggacgcgatactaagcgcgtggcggagagtcgtg<br>gtgcgctcaggccttttcagagatgcgagtcctgacgaggtggtggccgcatcacgtgtcctagcggtgtag	MASTDRTAKIRIVCLHGFSCNSAIYRFQTARLRKRW<br>AEHLRQHPLVAATSSASPSSTSSSRAASPGLDDPW<br>QRSNTQYYYDSDEESSSAATTTSSSSSSVDLAASIS<br>SRSSISFGSDYPPPPSYSSGSSGGNSGGIVVYNDV<br>EFIFLESPREVSAPEQPLAEVASLFPGPYRTWFEPS<br>TTTTTTTTTTTRRTAETAKPDDGSRARRRDPAIERW<br>VVEQVKRAGGVHGVLAFSDGAALTTALTLKAEAGV<br>AACPKLWDFVVLACGVDPYPDPWLHGSGSNGGIA<br>DGEAKEVEEEAKCLARLKTPGLHIVGLRDEVYRDRS<br>EALWGLVHPQAAAAAADRDEKGMNAASGMAAEVY<br>RFDDGHKVPVQDTDLDAILSAWRRVVVRSGLFRDA<br>SPDEVVAASRVLAV*
lasC	atggactcaatcgaggaaatccaacaaaaggtggccggcgttattgccggcgttttggaggatagcagcttcataggcggcgatgcagaccgcagcatcttatacc<br>gcggcgttgactcccttcgcgctctcgagctgagcgcttgcctgcaaactgaattcgacttgggcccggaggagattccaaataccttcgtcttcgagaatcccaccgt<br>ccgccaggcagccgaaaccatcgccgcagcgatccgcgcaacggcatcggcgtccactaccttggagttcggctcgcccacgccattgagcagcggcgatgat<br>gaagacgataaccaggctggttcagataacgctcgcctggccattgttggcatctcctgccgtctccctggcggtgccgtctcccccgagcgctactgggacatcctc<br>aacgaaggcgtcgacggcgtgaccgaaattcccgcccaccggctcgatctggatcgtctagttgcggaagccaaagtgtacgtccgcaaaggcgctttcgtctcc	MDSIEEIQQKVAGVIAGVLEDSSFIGGDADRSILYRG<br>VDSLRALELSACLQTEFDLGPEEIPNTFVFENPTVR<br>QAAETIAAAIRATASASTTLEFGSPTPLSSGDDEDD<br>NQAGSDNARLAIVGISCRLPGGAVSPERYWDILNEG<br>VDGVTEIPAHRLDLDRLVAEAKVYVRKGAFVSDVEH

	gacgtcgagcattttgaccacgaattttttggcatccccgcgagcgaagcggaagccatggacccgcagcaacgcctgacgcttcacgtcgtctacgaaagcttga	FDHEFFGIPASEAEAMDPQQRLTLHVVYESLSRAG
	gccgcgccggctggaacaagcaatccctgtccggactcgaagcgggcgtctttgtcggcaacagcgcagtggagtggtctgacgtgcagccggaagcggccttg	WNKQSLSGLEAGVFVGNSAVEWSDVQPEAALHGV
	cacggtgtctacggccgttctggcactgcttccgccgtactggccaaccgcgtgtcccacgtcctcggtctcggaggcgtttcggtcactgtcgactctgcatgctcatc	YGRSGTASAVLANRVSHVLGLGGVSVTVDSACSSS
	atcgctggtagctgtggatctcgcgtgccaggctctccgcgagggcaggtgccaagtggccgtggcagccggcgtgcatctccacctctcagaaaggcgctggat	LVAVDLACQALREGRCQVAVAAGVHLHLSERRWIQ
	ccagctctgcagcaccaaaatgctctctccaagcgcacggtgccgcgccttcgacgcccaggctgacggcatggtgcgaggagagggcgtcgccgcggtcgcc	LCSTKMLSPSARCRAFDAQADGMVRGEGVAAVAIM
	atcatgcctctccacttagccttggctcaaggccgcccggttcacggcgtgattctgggctcttgggccaaccacgccggaaaggctgctaacatcaccacgcccac	PLHLALAQGRPVHGVILGSWANHAGKAANITTPTG
	aggccagtcgcaacgcagagccgtcagcgctgctctgcggagtgccaaggtacgccccagtcaggtgtcctacgtagaagctcacgggactggtactgccctcg	QSQRRAVSAALRSAKVRPSQVSYVEAHGTGTALG
	gcgaccccatcgaactgggcgcacttgccagcgtgtttactgcggattctgctgttggaggagatgatgacaagtcttcgaaagaggaggagctggttataggctcc	DPIELGALASVFTADSAVGGDDDKSSKEEELVIGSA
	gctaaagcaagcatcggccatctggaatcagctagcggcctggccggcctaatcaaggctattttcgtcctgaagcagcaacaggctacaccacagctgcacctc	KASIGHLESASGLAGLIKAIFVLKQQQATPQLHLGGL
	gggggcctcaatcccgagattgagcgggcgactcgaggcttccgctttcgtatagtgactccggaatccgagagtcgatatattgctcctaagggtcagctgacgga	NPEIERATRGFRFRIVTPESESRYIAPKGQLTDKGKE
	caagggaaaggagacttacggccttgtctccagctttggattctccggcaccaacgcctgtgttgtgctcgctgcacccccgggactgtcatcctcaaattcacttgac	TYGLVSSFGFSGTNACVVLAAPPGLSSSNSLDAPVT
	gcccctgtcaccagctcgttaggcccaggcgttggaaagcggttcttcatgacgccttctgggagggagcatccgttgctcctgagatcggagtacgagctgctcga	SSLGPGVGKRFFMTPSGREHPLLLRSEYELLDSDE
	ctcggacgagaagttggttacctcaaccttacggctggggtcttactctgcagtctgggacgaccagctggtacaccgtgccttcgggccacgcaaggggtccttccc	KLVTSTLRLGSYSAVWDDQLVHRAFGPRKGSFPFS
	attttctgtcatcctcgaactgatccttgctactgcctgcagggcactcagtgcagatagattcggcgtcaccattgaagatctggacatccaagacaaggctagagtt	VILELILATACRALSADRFGVTIEDLDIQDKARVAFLG
	gcctttctgggagagggagacgctgttagatgtttcgtcgatagagagacagagagttgcgcagttttcttagcgccagcagcgggcgttgggtcattttcggtgctggt	EGDAVRCFVDRETESCAVFLAPAAGVGSFSVLVRC
	acggtgccgggttcgcgcaacggcacaagaaagcatctctgaatctctttccctgcggtcatttagccaaattccggcacggataaaaaagcaggtggccgatttg	RVRATAQESISESLSLRSFSQIPARIKKQVADLEKVP
	gagaaagttccgcttccaacattatacgaccgcctagaccgccttggatattccgttggtggcgaggctcggagaatcacgcggatcttcaggtcaaatactgattct	LPTLYDRLDRLGYSVGGEARRITRIFRSNTDSTPRR
	actccccgccgaagagtggtcgcatttgtaccgtttagccatgccgattcgggctttcttgtgcatccaatggaatggtcgagtatcgtacaagcagtgctttttgctgcg	RVVAFVPFSHADSGFLVHPMEWSSIVQAVLFAAAG
	gccggcgacgaagagaagtcgcagtacctaccctcggttgaggagagcttatccaatctatctctctcggccagaccgattggacaaaccggcccacgttggattg	DEEKSQYLPSVEESLSNLSLSARPIGQTGPRWIVAE
	tggcggaggtattgccctgccctgatggtcggacaatcgcgaatgttacggtaatcgaccacgacagcgaaggtgttgtgctgcaggtagacggtctttctctcatgc	VLPCPDGRTIANVTVIDHDSEGVVLQVDGLSLMQIS
	agataagccagagccaacttaaccagcggtccgacactggcgacgtttctggggccgaagtgtatgtgtccgactggaagcatctcaggatccccgacttccaagt	QSQLNQRSDTGDVSGAEVYVSDWKHLRIPDFQVAT
	agcaaccagcatctctgaaacggaatcaaagagaatgttaatggtttcttccattctgcaaggcaccggagaggaaaccactctcacagacgctctccatcaagcc	SISETESKRMLMVSSILQGTGEETTLTDALHQAFPN
	tttccaaactcaatacaagcgtacaagacagtaccttgggagcaactaacaccaggcggtacttccccgctgctcattgtgccactaccacgtttggaaatggagtc	SIQAYKTVPWEQLTPGGTSPLLIVPLPRLEMESAST
	agcaagcacggacgatccaaatccaagtagttgggcggcggaagcaatggctttggttcacttctttggcgagttggcgtcacggtcgaccgagacctggaagcc	DDPNPSSWAAEAMALVHFFGELASRSTETWKPTVV
	cacggtagtcctggtgggaagcggaataatgaacactccggacgatgacactcactcgtacggcgcacaggcaggctacctcgggatatgcaggacggcccgc	LVGSGIMNTPDDDTHSYGAQAGYLGICRTARSEYE
	tccgagtacgagagcatcgacgtccgtttgatcgacgtgcaggcatcaggggaggaggaggcgggccgagccttgaccgccgaggacgcatcagcgctgtccg	SIDVRLIDVQASGEEEAGRALTAEDASALSAVFGWI
	ctgttttcggctggatccaggatcatcctgacttgtcccggcttctgggacccgagatcgccatccgccgaggcgaggtctacctgccgcgcctcgtgccgctcgatcc	QDHPDLSRLLGPEIAIRRGEVYLPRLVPLDPSHTRT
	cagccacacacgcacacgcagcgctgttggaactgatgcgtctgcagctgtcagctttaattttgcctcgccgcaggaagggacggcgtggaaaggcatgacaca	RSAVGTDASAAVSFNFASPQEGTAWKGMTQLLQH
	gctccttcagcacgccgccaagcagcaatccgaggttgagtgcctctttcttctggaaccatccgctccgctagtcggtggagagaaagatggtttgccagccggcg	AAKQQSEVECLFLLEPSAPLVGGEKDGLPAGARMT
	cgaggatgacatacgcctcgtcatgcccctccacgacagcccgccaggcgctgcaaccatgcttccggccgacacacgacaaattgtgccttctaccgaatgagg	YASSCPSTTARQALQPCFRPTHDKLCLLPNEDPTA
	acccgacagcccccagcctcttcgactacgcacacttcgacgtcattatcgtttcagcgtcaagtgccgatctgtcaaaggcggcaaattgccttcttcccggcggcat	PSLFDYAHFDVIIVSASSADLSKAANCLLPGGMMLV
	gatgctggtaatgctggaaccgggcaccgaggacgttggcgcggaggtgatggctgcggagctctctcggcttggcctctccgtctcccggaccgatgatcgaaat	MLEPGTEDVGAEVMAAELSRLGLSVSRTDDRNGIL
	gggatccttgcaacggcctggcccgacgcgagcggcacgaccgtcacttccgttgcctcttctgacgtcatctctgatggcgatcgtggctgcttcgtcgttacgggcg	ATAWPDASGTTVTSVASSDVISDGDRGCFVVTGGT
	gcactggtggcctcgggctgctgtctgcccagcatctcatccgccagggtgctcgtcgtgttgtcttgttatcacgcagcggacgtcctgcgcctggttgtgagcagctat	GGLGLLSAQHLIRQGARRVVLLSRSGRPAPGCEQL
	tacaggaagtgctctcgagcaagggcgccgaggtgcacgttgcggaagccgacatttccgatgctaacgccctgtcacagctccgggagacgctgcggcagag	LQEVLSSKGAEVHVAEADISDANALSQLRETLRQRQ
	acaatggctacctgtgcgtggcattgttcacgctgctggcgtacttgctgacgctactctggctcgcctggacggtagcagcttcgacgtcgtcaataagcccaagatc	WLPVRGIVHAAGVLADATLARLDGSSFDVVNKPKIV
	gtaggcacgcgtcgcctgctcgagcatctatgcggtgacgctcacaatcgcgacatcgaattcttcgttggcttctcgtccgtggcaacccttctgggcttgccagcgc	GTRRLLEHLCGDAHNRDIEFFVGFSSVATLLGLPAQ
	aggctagctacgcagctgcgaacgctcagttggacgcaatcgtagcccagatgcgtgtggtccatggaattccagcagccacggtgcagctgggtggtgtgtccg	ASYAAANAQLDAIVAQMRVVHGIPAATVQLGGVSD
	acgtaggcatgagcgctcgcgttgccgaccatgtgcatgcttggggcgtcgggcagattgcaagtggagacttcggcgcgatgctccaaaaggtcatccagatgg	VGMSARVADHVHAWGVGQIASGDFGAMLQKVIQM
	ccaagctgccgcgccatcgcactctagttgccgcgaacttggattggaagacactccactcgacccgcaagcctcagcagaaccgccttctatccgctttcctacca	AKLPRHRTLVAANLDWKTLHSTRKPQQNRLLSAFL
	gcaccgcacaaaccggctggacagaagcgtacacagaagcaagagagcagggacgcagcaggcaccgcggcaacattacagccagccatgtcgctcgag	PAPHKPAGQKRTQKQESRDAAGTAATLQPAMSLE
	gaggtggagcagattgtgaaccagactctggaagatgttgcagggttgcaagtagcaaaggaggagtcactgtacgagtgtggcctcgactccctatccgccgtcc	EVEQIVNQTLEDVAGLQVAKEESLYECGLDSLSAVQ
	agttcaggaacgtgctcgctcagcgcttcgaaggcgtcgagcttgctccaaccctcgtgttcgactatcccaccgtcgaccacctaacgcaatacatccaccaacag	FRNVLAQRFEGVELAPTLVFDYPTVDHLTQYIHQQL
	ctgcagcagcgtgccgcggctgcacgcccaccaccacctccccagccgataccgtctgcccccactaccagcaacggccccacctccaacggaggcgacatg	QQRAAAARPPPPPQPIPSAPTTSNGPTSNGGDMD
	gacggcgcagttgcggtcatcggcgtcgaagcccgcatgcccggctcagccaacagcttcgacgagttctgggcgaacctgtgcgcgggcgtcgacggcatcat	GAVAVIGVEARMPGSANSFDEFWANLCAGVDGIID
	cgacgtgccgtacgcgcgcttcgacgcggacgactacctcgacgacaaccagctggcgggcggcaagacggcgtacgtgctgcgagcgggcttcgtcgacgg	VPYARFDADDYLDDNQLAGGKTAYVLRAGFVDGVE
	cgtcgagtggttcgaccacgagcgcttcggcatcacgcccatggaggccaaggtcatggacccgtcgcagcgcgtcatgctcgagacggcgcaccgcgccttcg	WFDHERFGITPMEAKVMDPSQRVMLETAHRAFAAA
	cggccgccggctacgacaaggacgcgctcaagggcaccaagacgggcgtgttcgtcggcagcttgtcgccggactggcggcaggtgctgacggagcacacg	GYDKDALKGTKTGVFVGSLSPDWRQVLTEHTAYSG
	gcgtacagcggcacgggaagcgcgctgtccatcatcgcgaaccgcgtgtcgtacgtgctgggcctggtgggcccgtccatggccatcgacacggcgtgcagctc	TGSALSIIANRVSYVLGLVGPSMAIDTACSSSLVAMH
	gagcctggtggccatgcacctggcgctgcagagcatccgcgcgggccagtgcgagatggcgctggtcggcggcgtgcagacgatcctcaacgtcgcgcccttca	LALQSIRAGQCEMALVGGVQTILNVAPFINGCKARM
	tcaacggctgcaaggcgcgcatgctctcgcccgacgcgcactgcaagtcctttgacgcgagcgcggacggctacgtcaaggctgagggctgcggcgccctcgtc	LSPDAHCKSFDASADGYVKAEGCGALVIKDLAAAR
	atcaaagacctcgccgcggcccggcgcgacggcgaccgcatcctcgccctcgtccgtggctccgccgtgaaccaggacggccgttcggccaatctcaccacgc	RDGDRILALVRGSAVNQDGRSANLTTPYGPSQRAL
	cgtacgggccgtcgcagcgtgctctcatggagcaggcgctgcagggcgcgggagtctcacccgacgatgtgtgctatatcgagacccatggtaccggtacgcccc	MEQALQGAGVSPDDVCYIETHGTGTPLGDPIEVAAI
	tgggtgaccccatcgaggtcgccgcaatccgtgaggcattcactagcgagacacgcgctgctcagcgccgtccgctgtaccttggtgctgtcaagtcggccatcgg	REAFTSETRAAQRRPLYLGAVKSAIGHLEGAAGIAG
	acatcttgagggtgcagctggcatcgccgggcttgtcaaggctgttggagtcatgtcccataggcagctgccgccgaacctgcactatcgctccctcaacccgtatat	LVKAVGVMSHRQLPPNLHYRSLNPYIDLKDASSWIR
	cgacctcaaagacgcgagcagttggatccgcattcccaccgatgttgttgatctggcgaatgcagacaacgagcgtcggccccttgtctgtggcgtcagcggctttg	IPTDVVDLANADNERRPLVCGVSGFGFGGTNAHVV
	gctttggtggcacgaatgcacacgtcgtcctgcaaagctatgacggtggcgaccagagaaaggtagagacgaggacagagactcagccatcagtgcagccgat	LQSYDGGDQRKVETRTETQPSVQPIDKVAFLFPGQ
	tgataaggttgcgtttctgttccctggtcaaggctgccaattcgttggcatgggtcaaaagttgttcggcgctgatcgagcatttgcggacgcagttgagcgttgcgatcg	GCQFVGMGQKLFGADRAFADAVERCDRIMQEQGL
	gatcatgcaggagcaaggactgcttggtggcaagagtctgatacgggacgttctgtggccttcgataccgcaggacccgccgttggtggaggacgcgcggttcac	LGGKSLIRDVLWPSIPQDPPLVEDARFTQPAIFAIEY
	gcagccggccatcttcgccatcgaatacggtctggcggaggcgctggcggcgcgcggcatccgcccggctgctgtcttgggccattccttcggagagttcgcagcc	GLAEALAARGIRPAAVLGHSFGEFAAAVTAGIMTLE
	gccgtgactgcgggtataatgacgctagaggatgccgcgaagctggttgccgttagggccagagtggctgcggacatggtatcgaccggagccatgttcctcatcc	DAAKLVAVRARVAADMVSTGAMFLIHAPAETVQKAI
	acgcccctgcagagaccgtgcagaaggccattgacactttccatcgatccaacgggtcaaaggaccaagccgccatcgcagcgaccaatggcccgcgcatga	DTFHRSNGSKDQAAIAATNGPRMTNIAGTEEACEA
	ccaatatcgctggcactgaggaagcttgcgaggcggtttgcaaggccgtcagggctcccaaggttcgactcgccatcccgcacgccttccattccccgctcatgga	VCKAVRAPKVRLAIPHAFHSPLMEPLREHMSRDLQ
	gcccctccgggaacacatgagccgcgatttgcagggcgtacggcttgcagtaccgtcgttgcgcttcttccctaccgtcgcgggccacaccgacgtagataccagc	GVRLAVPSLRFFPTVAGHTDVDTSKPSSSPAREAE
	aagccctcttcatcgccggcgcgtgaagccgagtactggctggatcaagtagtgcgcggcgtggacttcatggcgggtatgcggcgtttggctgaggcgggcttcg	YWLDQVVRGVDFMAGMRRLAEAGFDTFVEIGPRP
	atacttttgtcgaaatcggtcctcggccaacgctgatcaagatgggccagcgttgcgttactgccgatgcacagggtcctcagcgtcattgggtagcggcgttgcatg	TLIKMGQRCVTADAQGPQRHWVAALHDEGPEAES
	atgaaggcccagaagcagagtcgcccctagaccaccttgagcagcttgctaaaatgctttcagagaagaaggagagtggagagaagacgaaggttcatggcg	PLDHLEQLAKMLSEKKESGEKTKVHGGARRFWRPL
	gagctaggcgcttctggagaccacttccgcaccgcattgtaaagcaggttattccgacgtcggagaagggtgttttcaccttcgaacgcaagctgggccggcagctc	PHRIVKQVIPTSEKGVFTFERKLGRQLGKLLEEHRV
	ggcaaactcctagaagagcatcgagtcaacgggatagctgtcgttcccggggccctctacattgacaccgccttagccctgtttgctggtgcgaggtcgatccgcgc	NGIAVVPGALYIDTALALFAGARSIRAPEQGPSPVLT

	gcctgagcaaggcccatcgcccgtgttgacggatgccaatgtgagcagcatcgagttgcatgatctcgtctttgagcggccactgcagctacccacggccatggac	DANVSSIELHDLVFERPLQLPTAMDLGGLSAKQGIIV
	cttggaggtctatccgccaagcagggcatcatcgtggaaggcaggttggctctttcagctactgaaggcacggtgcgtatcgcttccagagccatcgagaacccag	EGRLALSATEGTVRIASRAIENPATITPHFSCTVSKL
	ctaccataacgcctcacttttcctgcacggttagcaaattggccggtcgaagcgccgcttctgctgcaattaacattgaggctctaacgcaaggtctcctgcacattggc	AGRSAASAAINIEALTQGLLHIGADAVYEASAGFGVE
	gctgatgccgtctacgaagcatctgcaggatttggcgtcgagtacggcacacggttccgctgtatcgaggagctgtggaagggagaaggagtggcagtggcccgc	YGTRFRCIEELWKGEGVAVARLRAPRTDNGFLLDV
	ctgcgtgcgccgaggactgacaacggcttcctacttgatgtgggcatgctcgacggagcgctgcagacggcagcgttcacagttgagagggagcaggcgtacat	GMLDGALQTAAFTVEREQAYMPFSVESVTVKGSIA
	gcccttcagcgtcgagtcggtgacagtgaaagggtcgattgccaatgccggccaggagtactactcaattgcgcggctccgtgggcgtgagcagaatatggtgga	NAGQEYYSIARLRGREQNMVEMDITLVDARGTPVV
	gatggacatcacgctcgtagacgcgcgtggtactccggtcgttgaggtacggtcgatgaaatcaagggctgcaccgaggcaggattcaattctcaagaagtcggtc	EVRSMKSRAAPRQDSILKKSVYEVEWVPLPTGVER
	tacgaggtggaatgggtgccgctgccaactggagtcgaacgtacgcaccgtttccaaggagatatgctcctcataggcacccaggaaggttctgttaacgagacct	THRFQGDMLLIGTQEGSVNETWIRKLASSLGMKPK
	ggatccgcaaattggcttcgagcctcggtatgaaaccaaagtttgtcgacgcgacgaaggtctccggccaggactttctcgtgctggccttgttatggttgcgccggc	FVDATKVSGQDFSRAGLVMVAPAAPPSENDMIPAT
	ggcaccaccgtctgagaatgacatgattccggcaacagagacaacggtggaacaaggattgaccagcttgcaagccgccttgcgcgcactagctgcggtcgagt	ETTVEQGLTSLQAALRALAAVESADQVAPVCLITHN
	cggcagaccaggtggcacccgtgtgcctcatcactcacaacgccgtcccagccgggagtggtaaggccaatccagcccacgccacactttggggactcgcgcgt	AVPAGSGKANPAHATLWGLARSARVEYTDLTVATI
	agcgctagagtcgagtacacagacctcactgtcgctactatcgatgtggatgatgcggcagtctcaggaaacttgcatggcctcgtctgcatcatgacgcagcatcg	DVDDAAVSGNLHGLVCIMTQHRNTWGQGEAEFAV
	caacacatggggccagggagaggccgagttcgcggtgcgcggcgacaacgtcttggtgccgcgcatggtcgaatcgttcgctgagggctgtcgtctaccgcagc	RGDNVLVPRMVESFAEGCRLPQQLAWRQASQAG
	agctggcatggcgacaggcatcgcaggcgggcggactgagcagtctcacctggattccgctggccgagccgcgggcgcctggtcctggaagcgtgttgctgcaa	GLSSLTWIPLAEPRAPGPGSVLLQVKAVGLNFKDVL
	gtcaaagcggtaggattaaacttcaaagatgtgctgaccgctacgggcgttgatgtcgatgcggttgacgtcgggactgactgtgcggggattgtacgcgccattgg	TATGVDVDAVDVGTDCAGIVRAIGQGVTGISVGDDV
	ccagggtgtgacagggatctcggtgggtgatgacgtcttcggaatcgccccaggctgctttaaggacctcgtcatcgcgcgtgccgaccgcctaacctccacacctg	FGIAPGCFKDLVIARADRLTSTPVRLTAYEAATLPSA
	tccgcctaaccgcatatgaagcggctacactcccctcggcataccttactgcccgtctagcccttctcgatgctccttcaatcaggccaggagaccgcgtgctggtgc	YLTARLALLDAPSIRPGDRVLVHAASGAVGQALVRL
	atgcggcttcaggcgccgttggccaagcgctcgtacgcctggcccaccagcacggctgcaccgtctacgccactgccggaagtgaggccaaacgggaatatctg	AHQHGCTVYATAGSEAKREYLLKQLGVAAVFPSRD
	ttgaaacaactcggtgttgctgcagtttttccttctcgcgatgcagcggccttccagcgcgagctgcgcgcccatctggcccgcgagggtggcaccatcgatgtagtcg	AAAFQRELRAHLAREGGTIDVVVNSLADAYIPNSVS
	tgaacagccttgctgatgcctacatccccaactcggtatcatttctgcgcccaggcggccgtttcgtcgagattggccgcaaatccatctggacggcggagcagatgc	FLRPGGRFVEIGRKSIWTAEQMRAHRPDIEYRIVEL
	gtgctcatcgaccggacatcgagtaccgaatcgtcgagctggacaagttgcattcccaacctggcagtgtcgcccaacacctagccgaggtggcctcgctcgttga	DKLHSQPGSVAQHLAEVASLVDQGSLEPIISEVFPRI
	ccagggctctcttgagccaatcattagtgaagtcttccctagaatcaaagtcgtcgaggcattccgtcgcatgcagagggcgctgcatatcggcaaggtcgtggtctc	KVVEAFRRMQRALHIGKVVVSIEDMPDTRASADILS
	catcgaggatatgcccgacacacgcgccagcgctgacatcctctctccggcccgcatccagcccgcgagtgtcgaacgggccgtctccgatatctcgtctcgtgca	PARIQPASVERAVSDISSRAAADLGVPVDEIERGWR
	gccgccgatctgggcgtgcctgttgatgagatagaacgcggctggaggcactctctcttactgctttcagcaactacgtcacgcagctcagcgagttggtcctagtg	ALSLYCFQQLRHAAQRVGPSGVVERQRRLYQRYC
	gtgtggttgagaggcagcgaaggctttaccagcggtattgccgcggagatcctacgcagcagtttgctgctgacgaagaagcgcccctgggactggacggtattgc	RGDPTQQFAADEEAPLGLDGIANSYPFLVPQVEML
	aaattcgtaccccttcctggttcctcaggtggaaatgctacgccaggtcgacggccatcatgaagctgtcttccgtggtaccaccgacccccttgaccctgctattctctg	RQVDGHHEAVFRGTTDPLTLLFSGGASKRIAQDLY
	gtggcgccagcaagcgcattgcacaggatctctacgagaaaggaatcttcgcccggttctgcaaccaggtcgccgcagatgtcacgggctacctcgctggctcatt	EKGIFARFCNQVAADVTGYLAGSLMPGATMKMLEV
	aatgccgggtgcgacgatgaagatgctggaggttggcgctgggactggaggcaccaccagccgtgtactgcccaagctgggtgacgccgtacagtcctaccactt	GAGTGGTTSRVLPKLGDAVQSYHFTDLSTSFLRHA
	cactgacttgtcaacatccttcctgcgccacgccgagaagcgcttcacagcgcagtatcgctatgtcaactaccacctgtttgatgtcgagaaggaccccgtgcctca	EKRFTAQYRYVNYHLFDVEKDPVPQGVRSQSFDVV
	gggggtgcgcagccagagtttcgacgtagtgctggcctgtaacgttttgcatgccaccgaaagaccttactcgtaccctgacacacgtgcgccagatcctcaagcatg	LACNVLHATKDLTRTLTHVRQILKHGGYLVLSEVTA
	gtggttacctagttttgtcggaagttacggcaccctcatactttgctgatgcaacattcggcatgacttctggctggtggctctttgccgacctggagctgcgtccagacta	PSYFADATFGMTSGWWLFADLELRPDYPLLTAQR
	ccctttgctcactgcccagcgatggagatcattcctctcaacaccatcgggttcgagcacgtatggatcagcgagcaaggcggcccccctgcacaaccagtctgtc	WRSFLLNTIGFEHVWISEQGGPLHNQSVIVARTPSA
	attgtagcccgcactccctcagcatctgccatatcgccgcgcatcccacgcccccctatccaccgccagcattcaggaccggaatggtgttgtggtcctcgcgggtgg	SAISPRIPRPLSTASIQDRNGVVVLAGGLGGLGVLTA
	cttgggaggcctgggcgtcctgacagcacgcctgcttctagagcgcggctggaagcgcttcgccttcctctcgcgctccggcgaggtgccggccgacgccaccag	RLLLERGWKRFAFLSRSGEVPADATSEWKHFTAGG
	cgagtggaagcacttcacagccggcgggccggagcaccgcatctaccgctgtgacgttgcggaccgttctgccgtcgagcgcgtcctaagcgaggtgcggcagc	PEHRIYRCDVADRSAVERVLSEVRQQQGPIVGVVQ
	agcagggcccgatcgtgggcgtcgtgcaggccgccggcgtgctcgactcggcgacgcttcccaaccaggacatggcgcgcttccgggcgctgatgggccccaa	AAGVLDSATLPNQDMARFRALMGPKVAGSWNLHL
	ggtcgccggcagctggaacctgcacctggcgacgcagcgcgaccccgtccgcttcttcgccctcttctcgtccatggcgtcggtgctgggctcggccggccaggcc	ATQRDPVRFFALFSSMASVLGSAGQANHAACNSF
	aaccacgctgcatgcaactccttcatggatgctttagctgcgcatcgccgtgcccaaggccttcctgccgtgtctatccagtttagcagtgtatctgctgtaggcgaggct	MDALAAHRRAQGLPAVSIQFSSVSAVGEAARRRAD
	gctcgccgtcgtgccgatttgcgacataccatcggatacctttctatcccgaagctcctggctcgcaacggtattgaggccctgtggactcggacgcagtggctgttgc	LRHTIGYLSIPKLLARNGIEALVDSDAVAVALSPIDWA
	gctgagccccattgattgggctaagatgctagcaaagtaccgcgtgccaccgaatcttttccaagctttctttggacgtgccgtcccagcaaacgctggcggagcaa	KMLAKYRVPPNLFQAFFGRAVPANAGGATEKLTTS
	cagaaaaactaacgacgagtggaaagccagctgtgcggcagcaggacaggaaagccaagcccgattctaccagcaagaaggctgctgctacgtctcggcgg	GKPAVRQQDRKAKPDSTSKKAAATSRRDQKKTPK
	gatcagaaaaagacaccaaaagaaaagaaggcaacagcggtcaagcgcagcgccccgcctcctaccatgattccggtgccaaccatgaatcagaggcca	EKKATAVKRSAPPPTTDSGANHESEARTLVVSAFR
	ggactctcgtcgtgagcgcttccgcgaggtcctcggctacgagccgccacgtgatgatatgggctggaaggacgccggggtcgactcgctattgcgattgagctg	EVLGYEPPRDDMGWKDAGVDSLLSIELRNAICARL
	cgcaacgccatttgcgctcggttggatgacctgggtgtctcgaagaaggttcctgtgacgtttgtattcgattacccgacggtccggcaggcggtaggtttcctggccgc	DDLGVSKKVPVTFVFDYPTVRQAVGFLAAAIATSAG
	cgcgatcgcgacgagcgcgggtgctgcctcagtcgagcaggcgactgtggagaaaaagagttcttcttcgaggagtacgaggaagccaatcaagccgcgcaa	AASVEQATVEKKSSSSRSTRKPIKPRKKASKTPNAS
	gaaagcctcaaagacgcctaatgccagtaagaagcccgaggcacccaagtccacaggggtggagagcgaagactttttgagccttgtactagaatcctggcaa	KKPEAPKSTGVESEDFLSLVLESWQETISTPIRPEQ
	gagactatatcgacgccgattcgcccggaacaggctttcgtcgactgcggcctcgactccctctcgtcggtggagcttctattcgctttgcagaccctgctggaagatc	AFVDCGLDSLSSVELLFALQTLLEDRIQLPENLVMQ
	gcattcagctcccagagaacctcgtgatgcagcagcccacaccacaagacgtcgcttcgtggctcctgcaacggcttggaagtaatgaaaacgcccggagtgta	QPTPQDVASWLLQRLGSNENARSVATETSTSLVNG
	gcaacagaaacatcaacatcacttgtaaatggtactgatcatgtggccctgatcatagaagcctggcacagcaccgttggtacggatctcggtgcggacgatgactt	TDHVALIIEAWHSTVGTDLGADDDFVEAGLDSLSSV
	cgtcgaagcaggcttggattctcttcctccgtggagctcctgtacgcactgcagcaaaccgtcggcgaaagcatcgaggtgcctgaggggattttcctgtcgcagaa	ELLYALQQTVGESIEVPEGIFLSQKTPKFLAAWLDD
	aacgcccaaatttcttgcggcgtggcttgatgacagattacccgttcagatatggagcacggcactattgcagtggccagcgaacacgaagacctttctgaagata	RLPRSDMEHGTIAVASEHEDLSEDNESEESEDTSS
	acgaatctgaggagagcgaagatacctcatcgtcagaatcagacgatatgtcagaagagtctggccagacggagtccacggaagagagcgatgacgacactg	SESDDMSEESGQTESTEESDDDTDNHLNNVDKWG
	ataatcatctcaacaacgtggataaatggggtgatgccgagcgcattgtgcactctgccatcgaagacgtacttgggtatagcatcgactccaacgcaccattcacc	DAERIVHSAIEDVLGYSIDSNAPFTDSGVDSLSSLEI
	gacagtggtgtcgactcactctcatccctcgagatccgcaacgcactgcaagcaaaagtgccatccgcgatcaaggtgcctgccacagctgtcttcgactatccctc	RNALQAKVPSAIKVPATAVFDYPSIKKLAEFLSQRM
	tattaagaagcttgcggaattcttgtcgcagagaatggccgagttggaaagggccagcagacccataagtaagccatcgcgctcggcgaagcctcagacaagga	AELERASRPISKPSRSAKPQTRKPVAKSKSSSSKAP
	agcctgtcgccaaatccaaatcctccagcagcaaagcgcccaaacaaagggttgaaaaaggcccacgcaacaagattgaagagacggaggatccgatcgtc	KQRVEKGPRNKIEETEDPIVIVGMASRFPGQTDDRV
	attgtcggcatggccagtcgttttccaggccagaccgacgaccgggttgagggctggtttgagtggctgctggatggcaaggatgccttcgttgagatccctcaccgc	EGWFEWLLDGKDAFVEIPHRRFDIDEVYHPGGESR
	cggttcgacatcgacgaggtctatcacccaggcggcgaatcgcgagcgggcaaatcgtacgtcagagacgccgctttcgtccacggcgccgagatgtttgacca	AGKSYVRDAAFVHGAEMFDHAHFGIVEAEAAKMDP
	cgcgcacttcggcatcgtcgaggccgaagccgcaaagatggaccctcagcagaggctcctttggagacgagcttccaagcactgcattgcagcggctacacca	QQRLLLETSFQALHCSGYTKQSLDGLDIGVFVGCC
	agcagagcctcgatggactggacattggcgtcttcgtcgggtgctgctccaacgactggtccaagatgcccgaagccagaggcagcgccttttcgggcaccggtg	SNDWSKMPEARGSAFSGTGAAASILSNRVSYALNL
	ccgccgcgtcgatcctctctaaccgtgtctcgtacgcgctcaatctctgcgcaccgagcatgaccatcgacacggcctgtagcagtgcgcttgtggcgctggatgcg	CAPSMTIDTACSSALVALDAAASALKLGKCRAAVVS
	gctgcatcagctctcaagctgggcaagtgccgagctgccgttgtgagtgctgtcaacttgatgctctcgtatggcaccttcgtcaccgagtgtgaagcatcgatgctgtc	AVNLMLSYGTFVTECEASMLSPSGKCRAFDSRADG
	tccttcaggaaaatgcagagcgttcgactctcgcgcgatggcttcgctcgcggcgagggctgcggcagcgtggttgtgaagaagctctctcaagcccgtgccgac	FARGEGCGSVVVKKLSQARADGDEILAVLAATAINQ
	ggtgatgaaatcttagctgtcctcgctgccaccgccatcaaccaggatggtagaagtgctggacttaccgctcccaatggcccttctcaagaagctgtcatccgcagt	DGRSAGLTAPNGPSQEAVIRSAIGAAGLRPEQLIHL
	gccatcggggcggctggactccggcctgaacagctcattcatctggagagccacggcacggcgacgaaactgggtgacggtatcgagtggaccgctctggctaa	ESHGTATKLGDGIEWTALANVFGDRSSREPPLYVG
	cgtattcggtgaccgctcctccagagagccgccgctttatgtgggaagcgtgaaggccaacgtgggtcatctcgaaggtgctgcgggcatggcaggcttgataaag	SVKANVGHLEGAAGMAGLIKSVMILRSGKIPPQANF
	tccgtgatgatcctcagaagcggcaaaatccctccgcaggccaatttcgagacacttaacccgctcttcgaaacaggccacgccctagctgttcctaccaaattgac	ETLNPLFETGHALAVPTKLTSLNGIDLSKFACGISSF
	ttcgctcaatggcattgatctcagcaaattcgcttgcggcatctccagttttggattcggcggcaccaacgcccacgccatcgtgccgcctcccaacggccagcgaggt	GFGGTNAHAIVRLPTASEVPQSSFGTHRLLRTPWP
	gccacaaagttccttcgggactcatcgtcttcttcgaacgccatggccctttgatggacgcaagtatattccttggcgtgccagcgatagcccgttcgtgacctccaagg	FDGRKYIPWRASDSPFVTSKVFRDGDRWAVYRVAL

	tcttccgcgatggtgaccgttgggcggtttaccgcgttgccctgggtgagcgccttcgaaaggacctgctggcgcaacaccgtgtccgcggcacaattattgtacctg	GERLRKDLLAQHRVRGTIIVPAAAIIEMTRAALEAHL
	cggcagccatcatagaaatgacccgcgctgctcttgaagctcaccttgcggacgaatctgcgggtcttgaacttgtcgaacttatcctggccaaagcaatcttagtcc	ADESAGLELVELILAKAILVQDVSPASPVILQCATLAN
	aggacgtaagccctgctagcccggtgattctgcagtgtgcaacgctggccaacggtgaagacagcggaaagagttggcatatcgagacagaatgccagggag	GEDSGKSWHIETECQGEVILHATAAFGRCDGDTTE
	aggtcattcttcacgccacagccgcctttggccgttgcgacggggacacgactgagtctactttagacgtcgaagatgccagcatgttacacacaaccctcatcaag	STLDVEDASMLHTTLIKPSDFYRRAADRGLEYGPLF
	ccttctgacttttacaggcgtgctgcggacagaggtcttgaatacgggccactcttccaggcccttactcgcattgcagtcaagcgtgacggcagcagtgcctttgtca	QALTRIAVKRDGSSAFVKIGGSQYETSASPASTFILS
	aaattggcggctctcagtatgagacttcagcgtcgccagcgtctacgttcatcctctctcgaaacgattacttcgtccctccccatctctgcgacgctgctttccaggcttg	RNDYFVPPHLCDAAFQACSLLHREDAANQTFLPFQI
	ctctcttcttcatcgagaggacgcagcaaatcagacttttctgcctttccaaatcgggcaggtgcgttggcacactaccagacaccaggttgacaggcggcctaagta	GQVRWHTTRHQVDRRPKYAICQRQGGAPDSLVAS
	tgctatctgccagcgacagggcggagcacctgattcactggttgcttctgtggccatatatgaccagcgaggtgaacttctcctatcggtcgggaatttcgtggcccag	VAIYDQRGELLLSVGNFVAQAIQKSKESLDVIENAAG
	gctatacaaaaaagcaaggagagcctggacgtcatcgaaaacgctgctggaagggtccaggagggtacagatcagcacatgggtggctcaaacagcggagtt	RVQEGTDQHMGGSNSGVSESRAAVIIPKRMFRESL
	tccgagtcacgagcagcggtaataattcccaagcgcatgttcagagagtcgctacagctggtccccgccagccgatctctgggtagcgggacgggcttcacgcgc	QLVPASRSLGSGTGFTRFALIRSLSGRPTPRELLRL
	ttcgccctcattcgctctttgtccgggagacctacaccccgagagctcttgcggcttccgattcctcaggtcgatgttcaccccacgcacaacctccatcaacgactctc	PIPQVDVHPTHNLHQRLSSLSGLEGLLFAPSARLND
	gtcgctgtcaggcttagaagggttactctttgctccatcggcccgcctcaacgaccctgatggagatggctctcttgaaatcctcgaagtcgtcaaagcgttggacagg	PDGDGSLEILEVVKALDRMNVPVPLTIIDVTGVSTPT
	atgaatgtaccggtaccactgacgatcattgacgtaactggcgtctcgactcccacaagcaatagcagaggtacggcagaggttccggagaacaattggaccctttt	SNSRGTAEVPENNWTLSGLVRACRTELPDLKIFLW
	cgggcctcgtccgtgcatgtcgaacagagctccctgacctcaaaatcttcctctggcatgttgactcaacattggctcagcgcgacggctttttcacttctccgcccgca	HVDSTLAQRDGFFTSPPAMHAASLLPLEQELFTVAN
	atgcatgcagcttcactgctgccattggagcaagagctcttcaccgttgccaacgggcagtacacccccgcttgcaacccatcgcgccgatggagaaggaagta	GQYTPRLQPIAPMEKEVVGLECQVGADAEPDFGHL
	gtcggcctcgaatgccaggtcggcgcggacgcggaaccggactttgggcacctccgcatcataccccctgagacggcagcctggcgacggacccggaccagac	RIIPLRRQPGDGPGPDEVEIQVRAVGLNFRDVLNAM
	gaggtagaaatccaggttcgcgcggtgggactgaacttccgcgacgtactgaacgcgatgggcctgaacctaggcgtgccggggcccctgggtgccgactgcgc	GLNLGVPGPLGADCAGVVSRVGINVQKWRVGDEV
	tggtgtcgtcagccgtgtcggcatcaatgtgcagaagtggcgcgttggcgatgaggtgctcggaattgcccgcggctgtctgcgatcctatgccaccactgaccagc	LGIARGCLRSYATTDQRRLIAKPADMTFEQAASVAS
	gaaggctgatcgctaagcccgcggacatgacgttcgaacaggcggccagcgttgcatcggtctatgctaccgtttaccactcgttcgtcgacgtagctcggttgaga	VYATVYHSFVDVARLRAGQTVLVHAAAGGVGLFAI
	gctggacagacggtccttgtccacgccgctgcaggaggtgtcggcttgtttgctattcaactcgctaaacatctcggtgccaacatcgtagctaccgtcggcagcccg	QLAKHLGANIVATVGSPEKEKLVRSYGVQLVSSSRD
	gaaaaggaaaaactggtccgctcatacggtgtgcaactcgtgtcaagctccagggatgtccgcaagttcgagaccgatatggcacaatggagaaccaggccgg	VRKFETDMAQWRTRPDVVLNCLTGDFIRVSLDFMA
	acgtggtgctcaactgcctcaccggcgacttcatccgcgtttctctggacttcatggcaccttcgggtatattcatcgaactcggcaagcgtgagatctggacggaaga	PSGIFIELGKREIWTEEQVRSVRPDIQFVVVAIDKML
	gcaagtccgcagcgtgcggcccgacattcagttcgtggtggtcgccatcgacaagatgctggcggaagatcctcaggctcatgcggatctcctcttgagggtcgag	AEDPQAHADLLLRVERELLAGWSQPLPIQKFAFTDV
	cgtgagctgttggctggttggtcgcagccactaccgatccagaaattcgctttcaccgacgtggccaaggccttcgtcagcatgaggcgtgggcagcacgtgggca	AKAFVSMRRGQHVGKVVLSLEHASLSNATGSSALS
	aggttgttctgtctcttgagcatgcctctctttccaacgctaccggaagctcggccctgtcgaccgtaccgaacctcgaacgcctcctgcgaaccggcatggatgacg	TVPNLERLLRTGMDDAMAEFFTTEFDRTELLAGLEA
	ccatggccgaatttttcactaccgagttcgataggacagagttactggcaggacttgaggctgtggacaacattgcaagaacatatctgcaggattgtatgtccgctgc	VDNIARTYLQDCMSAAMPSQVTPHLRALYDLYRHQ
	catgccgtctcaagtaacaccacatctccgagcgctctacgatctttatcgccaccaacggccctcatatggtgtcgctaacggagcagcggatgtgaagcggatgt	RPSYGVANGAADVKRMYRSVGARVDFLEHVRREH
	accgtagtgttggtgcccgtgtggatttccttgagcatgttcgtcgagagcacctggccatgttaacaggagacacggatcccgtctcggtgcttttctctgacccagag	LAMLTGDTDPVSVLFSDPELTKRLYDESPLAALFNK
	ctaaccaagcgactctacgatgaatcaccacttgcggctctattcaacaaggttatatcgggagccttgaaatccgtcgctgcggaaacgggtagtctcaaggccatt	VISGALKSVAAETGSLKAIEIGAGTGGTTRSVCDVLG
	gagatcggtgccggcactggtggcacgacccgcagtgtttgtgacgtattggggtcgctttgccgctcattctgcttcaccgatatctccacttctttcttcaaccgcgcac	SLCRSFCFTDISTSFFNRAQQEFAPSIPGFHTALLDI
	agcaggaattcgcaccgtcgataccgggtttccataccgcactgctggatattgaacgcgaccctgaacatcaagggttcatcttgggcagctacgacctcatcctg	ERDPEHQGFILGSYDLILAANVLHATADLSVTLANIH
	gcggccaacgtgctacatgccacagccgatctgtcagtaaccctcgcaaacatccatgcgttgctgaagccgggcggcatcctcgtcctctctgagctcactgattcc	ALLKPGGILVLSELTDSSVWADATFGFTKGWWLST
	tcggtgtgggcggatgctaccttcggattcaccaagggctggtggctctccaccgacgaccgacgttccagcggccgcggtcccctgattagtttggctcgctggaat	DDRRSSGRGPLISLARWNNAFRRANLEPVTSSDETI
	aacgccttccgccgcgccaacctcgaacccgtaacgtcgagtgatgaaacgatagctgcgcagacaatcatggttgctcgcaggccgcttcatcctgtgaaccattt	AAQTIMVARRPLHPVNHFHSTIPLAVGQRARGVPSA
	ccactctacgattcctcttgcagtcggtcagcgtgcacgcggtgttccttctgcagtcaatgaaggggtgtatgtcatcactggcggtacgggtggacttggtcttctatcc	VNEGVYVITGGTGGLGLLSAHILILEGARRIALLSRS
	gctcatatcttgattctggagggtgctcggcgcattgctctgctctcgcgctcgggaactgtcccaggagaggctgaggcagagctttgggcccttctccaacggtcac	GTVPGEAEAELWALLQRSRCQISIHRCDVSDEVQL
	gctgccaaatcagcattcatcgctgcgacgtgagcgacgaggtgcagctcagacgcatcttcgactcgtttggcgtacccgtggtaggcatcatccacgctgccggt	RRIFDSFGVPVVGIIHAAGHLSDASIGNQSTATFGTV
	cacctatcggatgccagtatcggcaaccagtcaacagccaccttcggcacggtgatgcgcaccaaggccagcgggctgctcaacctgcatcgcctctccctgcag	MRTKASGLLNLHRLSLQHPVKMFVVFSSASAFFGS
	catccagttaagatgtttgtcgtcttttcctccgcctccgccttcttcggttccgcgggtcagaccaaccacgccgcagcgaactcgttcatagacggcctcgtccgctgg	AGQTNHAAANSFIDGLVRWRRRVGLPASSIMWGAV
	cgcaggcgcgtcggcctgcctgcttcgtccatcatgtggggagctgtttcccaagttggcgatgcagctcgcaggggtgccgatcgcaggaagaagatgctgtacg	SQVGDAARRGADRRKKMLYDPLSLVDAAEVLSTVL
	acccccctttctctcgtcgacgccgccgaggtgctttctaccgtgctctttggcgatttagacgtcgtgctcgctgctccgatccgaccggcgactatcagcagcgattaca	FGDLDVVLAAPIRPATISSDYRGNKRIFGGLIPSEDA
	ggggcaacaagcgcatcttcggtggtcttattccatcggaagatgcaaacaccgcaaatggcacccaggagaccattttcaggacccccgagccggtgccaagg	NTANGTQETIFRTPEPVPRPRPVLESQQKTAPSTSA
	ccacggcccgtccttgaatcccagcaaaagactgctccatcaacgagcgcaagtaagcatgcagactacgacagggtcttgtcgaccatccgctcgctagctacc	SKHADYDRVLSTIRSLATKYIGRQPADDELLVGAGL
	aagtatatcggtcgccagcctgcggatgacgagctgctggtgggtgctggccttgactcgttgtcggcgatcgaccttcgcaactccatccaagcaaaactgcttcctt	DSLSAIDLRNSIQAKLLPSGTRLPASTVFDHPSAADL
	cgggcacaaggctgcccgctagcaccgtgttcgatcaccccagtgcggccgatcttgctcggttggtctgttcgaaattgggaagcggttcggttccgagtgcgtcac	ARLVCSKLGSGSVPSASQPAPSSSTFYRPELAAPA
	aaccggcgcccagttcaagcaccttttacaggccggaattagcggcgccggcgccgacaccagcagtgacggtaccttcggtggcaactgacgatgttctgcttgt	PTPAVTVPSVATDDVLLVVRRVASRLLSRIPDDDEP
	agtacgtcgggtggccagccgtctgttgtcccgcatccccgacgatgatgagccccttgttgctgcagggctcgattctttgacggcgattgagttccgcaatatcctcc	LVAAGLDSLTAIEFRNILQKEFGITQRLKPTLVFDHPT
	agaaagagttcggaattacccaacgcctgaagccaacccttgtgttcgaccatccgacgtcaagagaaattgcagacttcctcctgccaattttgtcctctggaaacg	SREIADFLLPILSSGNVARTSTTVAAPSASFQPSNRA
	ttgcaaggacctccacgaccgttgcagctcccagcgccagttttcagccatcgaacagagccacgacgcaggcagtttctagccgactatcagaacagcctgcga	TTQAVSSRLSEQPARPVVSSTLEPTFVHRDTSRAFE
	ggcctgtcgtgtcatccacattggagccgacatttgtgcatcgtgatacttcgcgagcctttgaacaagtcaaggctgctcctgtaaacagcgccttggagattgcagg	QVKAAPVNSALEIAGIGCRLPGRATDPGKFWKNLIH
	catcgggtgccgacttccgggtagggccactgatccgggcaagttctggaagaacctgatacataaggtcgactgcattgtacctgttcctcacgaccgtttcgacat	KVDCIVPVPHDRFDMDDFYDPVPGTEGKSYVDQAA
	ggacgatttctacgatcccgtgccaggaacggaaggcaagtcatacgtcgaccaagccgcctttattgatgacattgacctgtttgatcggcacgcgttcaacatgtct	FIDDIDLFDRHAFNMSPSQVSQMDPQQRLALQVAL
	ccttcccaggtctcccagatggaccctcagcagcgtcttgcgctccaggtggcactagacgccttcatggacgctggatatgaagacaggacaaaactgagaggc	DAFMDAGYEDRTKLRGSNTGVFVGVCSNDWHKVK
	agcaacacaggtgtctttgtcggtgtctgctccaacgactggcacaaagtcaagaaggacccgagctttctaggcggcggttttgacgtcgccggcggggctaactc	KDPSFLGGGFDVAGGANSMIANRISFEFGLKGPSV
	gatgattgccaaccgtatctccttcgagtttggcctcaagggtccctcagttgccattgactctgcctgctccagttcactacaggctgttgacatggccaggcgagctat	AIDSACSSSLQAVDMARRAILSGVCDRALVLGVNLIL
	cctcagcggtgtctgtgaccgtgcgctggtgctgggtgtaaacctcattctctccccggatactttcatcggcgaatgtcaagcaacgatgttgagccccagctgcagg	SPDTFIGECQATMLSPSCRSKAFDDEADGFARGEG
	agcaaggcgttcgatgatgaggccgacggttttgctagaggagagggatgtgccgcaatagtgcttgagaagcctggtcacggtccgaagagcgacgcatatgc	CAAIVLEKPGHGPKSDAYAELIGSASNHDGRSASLL
	cgagctcatcggatctgcatcaaaccacgacggccgatccgcaagcttgctggcgcccaacggacctgcccagacctccgtgatcaacgcagcgctggccgag	APNGPAQTSVINAALAEAGIAGYEVDFLETHGTGTK
	gcgggtatcgcgggctatgaagttgactttctcgaaactcatggcacgggtaccaaagtcggggacccgctcgagttcaatgcattccaggaagccctggcctctgg	VGDPLEFNAFQEALASGRSPQRPLHIGALKTNVGHL
	ccgctctccacaacgcccactacacattggagcgctgaagacgaacgttggtcatcttgagggcgcagctggaattgcgggcctcgtcaaagcagcgctcgtcctc	EGAAGIAGLVKAALVLQHAQVPANLHFRKLNSHIDA
	cagcatgcgcaagtgccggcgaaccttcatttccgaaaactcaattcccacatcgacgctgagggtttcccggttgtcttccctagccagacagttgctctaccggca	EGFPVVFPSQTVALPATNGPLVGSVSSWGFGGSN
	accaacggcccgctggtgggctctgtgtccagttggggctttggaggatccaatgtgcacgcagtgctacgccaggcaactcatacgcgaaagccgcaggaaga	VHAVLRQATHTRKPQEEDIKRETGQGSVATDIAPCP
	agacatcaaacgtgaaacgggacaaggcagcgttgcaacggacatcgcgccgtgccctggactcgttgccatgttcacaggtcaggggagtcaatcggtcaac	GLVAMFTGQGSQSVNMASSLYRSDSTFRNCIDECA
	atggccagctctctataccgcagcgattccactttccgcaactgcatcgacgagtgcgccgccattgccgacgaccttctcgacagacctctgctatccgtcctctatc	AIADDLLDRPLLSVLYPPAGDEARAAQLIEDTSYSQV
	ctccagcgggtgacgaggccagggcggcacagctgattgaagacacgtcgtacagccaagtctgtcttttcgccgtcgaactcggacttttccgcatgctgcagcgt	CLFAVELGLFRMLQREGFCPAVVLGHSLGEFVAAH
	gagggcttctgcccggccgtcgtgttgggccactctctgggtgagttcgtcgcagcacatgtggcgggctgcctcagcctacgcgatgcgatgcgccttgttgccacc	VAGCLSLRDAMRLVATRGKLLGALPRNVGSMVALV
	cgcggcaagcttcttggtgcgcttcctcgcaacgtcggcagtatggttgctctagtggccagcgcgggggaagtagctgctacaattcggagactacagctttcccag	ASAGEVAATIRRLQLSQCSIAAINSPRMTVISGAKHE
	tgctccatcgccgccatcaacagtccgagaatgacagtaatatctggcgctaagcacgaggtcgacgaggtcagccggtccatcggctgtgtcgcgcgaccgctc	VDEVSRSIGCVARPLKVSHAFHSPLMRPALRGFEE
	aaggtgtctcacgctttccactcgccgctcatgcgtcctgcgctgcgaggcttcgaagaagctctagccaatgtcaagatccgggcaccgcaaccgggaggcatac	ALANVKIRAPQPGGIQLVSCVTGRLAGAELENPAH

	agctggtcagctgcgtaacgggcaggctcgccggcgcagagctggagaatccggcgcactggctctcgcacatcgtcgatccggtgcgcttcctcgacgcggttc	WLSHIVDPVRFLDAVQEAHRLGGRCFVEIGPRAVLA
	aagaggcccaccgcctgggcggccgctgcttcgtcgagatcggccctagagccgtccttgccaagctcgtgaggcagtgcatcacagcgccgttgaccattactg	KLVRQCITAPLTITATQDPPKDDHGAWSEAISKVRG
	ctactcaggacccgcccaaggacgatcatggtgcttggagtgaggcaatttccaaggtgcgtggggtcgtgaatgcacaactcccacctgccgttgccatcacaca	VVNAQLPPAVAITQPSTAPITRPAITKRAERFPYRKT
	gccgtctaccgctcccattacacgcccggctatcaccaagcgggctgaacgctttccgtaccgtaagacacagcacccgttcctcaaaaaccggaaagatcactc	QHPFLKNRKDHSDGRVTFTVSIRRSLASLLKQHVVH
	cgacggccgggtcaccttcaccgtttctattcgtcgttcactcgcatctctcctgaagcagcacgtcgtccacggacgcgtgtcatccctggagcacttcatatggagc	GRVVIPGALHMELAASAARQVHSSSAGCIVLEDVTF
	tagccgcaagtgctgctcgacaagtacacagctcctctgctggctgtatcgtgcttgaagatgtgacgttctctgccccctgtgtcctatccgcagaagatatgcaggg	SAPCVLSAEDMQGDDSEKILPSLFCTVCPSDRTISIS
	agacgactcagaaaagattcttccgtcgctcttctgcactgtctgcccctcggatcggactatcagcatttcgtttggtcaaggcagccctgagacactatcacccaatg	FGQGSPETLSPNVEAQYVVLDNNSNPRTGSVGAG
	tagaggcacagtatgttgtcttggacaacaacagcaaccctcgcacaggtagtgttggcgcaggcatgaataacggcacctcacccgacgacgggaatcaggc	MNNGTSPDDGNQAAAEVYRHFELKGLQYGPHFRT
	ggccgcagaagtgtataggcactttgagctcaaggggctccagtacggcccgcacttccggacacttcgaagcatctccggatcccgctcacaggcgtacgctcg	LRSISGSRSQAYARHSLALVGHGMFAHPCLLDGILQ
	ccactctttagcgctggttggacacggcatgttcgcccacccgtgtctgctagacggcatcttgcagacctccgccggtctcttcctcgacactctggcgtcaactccgg	TSAGLFLDTLASTPARMPFNVERIEFLRPLPSCVNV
	cgcgcatgccgttcaacgtcgagcgcatcgagttcctgcggccgctgccttcctgcgtcaacgtcgtggcgacgaggacggataacgcgactgacgacaggttcct	VATRTDNATDDRFLRIDLAVYSENDDELLMMVSGL
	gcgcatcgatctcgccgtatattccgagaatgacgacgagctgttaatgatggtgagtgggctgcagagccgcccagcgccttcgtcggggcggcattggcaccg	QSRPAPSSGAALAPLKEAGTPLFYQQAYANEEVRR
	cttaaagaagctggcacgcctctcttctaccagcaagcctacgcaaacgaggaagtacgacgcgatgctccgttctccgcgcagagtgtacaacaggtgtcatac	DAPFSAQSVQQVSYVVVGDRFNPIKRRLIDTLGYST
	gtggtggttggcgacaggtttaaccccatcaaacgacgcctcattgacacactgggatactccacgcaagtggtcgaattggaaacactcactcctcaccaagcaa	QVVELETLTPHQATSHRTNTSSARTVVVDCRAFDE
	caagccacagaacgaacactagcagtgcccgtacagtggtcgtcgactgtcgcgcgtttgatgaaagcagacctcggctgagtggcgttgacaccgactcggag	SRPRLSGVDTDSEEDLATSTLAYYASLCNSEHLPDC
	gaagacctcgcgacatcgaccttggcatactatgcctctcttgcaacagcgagcatcttccggattgcctcgtgcacatcacttcgggtgccctcaaccccggtcctg	LVHITSGALNPGPAVRTVKGAALAGMAKAFKVEQY
	cggtcagaacagtcaagggcgcagcattggcgggaatggcgaaggcgttcaaggtcgagcagtaccaccttccgattactgttatggctcttgatgttcaggatga	HLPITVMALDVQDEDVTAPSLIVDEIAAAVRGDSSN
	agatgtgactgcgccatcgctgatcgtagacgagatcgccgctgccgttaggggcgatagcagcaatggtggcttcattcaaattggccttcgaggaagcacaaga	GGFIQIGLRGSTRSVPVTVMAPVSCDSRSGFEDGR
	tctgttcctgtcacggttatggcgccagtttcctgcgacagccgaagcggctttgaggacggacgatcatctcttcgccttcagacggctgcagaggtaacgcgactg	SSLRLQTAAEVTRLLASSTTTFEEENRAIVERFSRAD
	ctagccagcagcacgaccacgtttgaagaggaaaatcgcgccatagtagagcgcttttcgcgcgccgatgccgagcttcaagatgttgctcgcaattgatggctca	AELQDVCSQLMAHAAWQTQRWEVTPHLLRTWQR
	tgccgcctggcaaacacagagatgggaagttacgccgcacctgctgcgcacatggcaacgataccgcaccagccctccacgcgaggcattttcagcattgctag	YRTSPPREAFSALLERLARLRTEYPDATGNKSLLGP
	agaggctggcacgactccgcaccgaatatcctgatgcgactggcaacaagagccttctaggcccgcagttggtgctgctcgagtccacagccaagcagttgaac	QLVLLESTAKQLNEVFTGRVDPLTLLFADQNQDAAG
	gaagtgtttacaggacgcgtggacccattaaccctcctttcgccgaccagaaccaggacgcggcaggcgagttgtacaagagcaccttcctcgcacgccacttca	ELYKSTFLARHFNKLVVAAVIDAIQTSALPADYTVQV
	acaaattagtcgtcgcggccgtcatcgatgcgatacagacgtcggcacttccagccgactacacggttcaggtgctcgagattggcgccggcacaggcggcactg	LEIGAGTGGTASFVLPALRDLGVRVRYCFTDLSSSL
	caagtttcgtgctgcctgcgctgcgcgacctgggtgtgcgtgtgcgctactgcttcaccgacctctctagcagcctgctcgctcgtgcgcgtaagaatttcagccgctat	LARARKNFSRYPFVEYRMLDVERPCEPQGFAPETF
	cctttcgtcgagtaccggatgctggacgtcgaacggccgtgtgagccacagggattcgctcccgagactttcgacatcgtgctcgccaccaacgtcctccatgctac	DIVLATNVLHATADLDAVMANVQELMLPGGRLIVSE
	ggctgaccttgacgccgtcatggccaacgtgcaggagctcatgcttcccggcggacgattgatcgtatcggaactgacacgcgtgatgcctttcgctgactgcacgtt	LTRVMPFADCTFGLTTGWNAHQDRYRHDGPLLSA
	tggcctgaccactggatggaacgctcatcaggaccgctaccgacacgacggcccgctactctctgcagcaagttggttcgacgttttccgcaacgctcgtctacagc	ASWFDVFRNARLQPLAHSPQESIFANQAILVAARDA
	ctctggcccattctcctcaggaaagcatcttcgccaaccaggcaattttggtggctgccagagacgccgcagtccatccttcgaccagacagaggctggacatccg	AVHPSTRQRLDIRGSDATYLITGGLGGLGLLTAKTM
	cgggtcagacgcaacgtacctcatcaccggtggcttgggtggtctcggcctgctcacagcgaaaaccatggttgagcaaggggcgcggaatataatcctcgtctcg	VEQGARNIILVSRSGKPAAGSKDDWDTLNALCARS
	cgcagcggcaagcccgccgccggctccaaggacgattgggacacattgaatgcactctgcgcgcgttcatccgtccgactggaggtcttcggctacgacgttagc	SVRLEVFGYDVSDEGNVTALFSRIRSLGLPPIRGVV
	gacgagggcaatgtgacggccctgttcagccgcatccgctccttaggcctaccgcccattcgtggtgtcgtgcacagtgcgggaatcttgcaggacggcacattcac	HSAGILQDGTFTAQTAASYRAVLKTKAAAARLLQAQ
	cgcgcagacggccgcatcgtaccgcgcggtgctcaagaccaaagctgctgctgcccgtctcctgcaggcccagctcccgcatctggacttcttcgtgagctacagc	LPHLDFFVSYSSVASLLGAPGQSNHTAANTLLDCAA
	tcagtggcgtcgcttctgggcgctccgggccagagcaaccacacagcagccaacaccttctcgattgcgctgcggaatggcaatgggcagcagcacacagcg	EWQWAAAHSGSRPSFASLAVQWGAVSGVGDAAR
	gctcccgcccctccttcgcctcgttggcagtgcaatggggcgccgtctccggcgtgggagacgcggcccgccgcggcgctgctgatgctgagaaacggtcttcga	RGAADAEKRSSSVHAAIPKDTAEALIRKLFTVAFQR
	gcgtccacgccgccatccccaaggacacggccgaggccctcatccggaagctcttcactgtcgccttccagcgtcccgttatcacgttcgtgccgttcttgtggcaca	PVITFVPFLWHNLWKADCKFASERFAAFKPARYLPP
	acctgtggaaagctgactgcaagtttgcttcggagcgcttcgctgccttcaagcccgctcgatacctaccgcccgctggcgctacccctgaggctcccactacccgtc	AGATPEAPTTRQGPAVAVNADKLVESRPGPSEQPA
	aaggtccggccgtcgctgtcaatgccgacaaactgtcgagtcccgacccggtccgtctgaacagcccgcagccgctgctcccgctctaacgcagattctggacga	AAAPALTQILDETVEDVLGRSVEPSESLVDAGVDSL
	gacggtcgaggatgtgttgggacgcagcgtcgagccaagcgaatcgctcgttgacgccggcgttgactcactgggtgctatcgatttccggaacaagctgcagaa	GAIDFRNKLQKKIPGLRAPATLLFEHPTKAALLEFLA
	gaagattcctgggcttcgcgcgccggcgacgctgctgttcgaacatcccaccaaggcagctctgctggaatttctcgcaaagcacgctccagcagccgcagcaaa	KHAPAAAAKPTPLPQQQAPVKPTPAISDAIATVSTG
	accaactccgctgcctcaacagcaagccccggtgaaaccaacaccagcaatctcagatgctatagccactgtatcgactggcgcccacagcgcgagttggcc	RPQRELAVVGLACRLPQADSPQEFWDLLSKHPSTA
	gtcgtcggcctcgcatgccgtctgccgcaagctgattcgccgcaagagttctgggacctcctctccaagcaccctcgaccgcgtttggccgcgtgccagccgacc	FGRVPADRCDMTSVLDPSGRSRAAAYTDTGAFVR
	gctgtgatatgacctccgtactcgatcctccggtaggagccgggcggcagcctatacagacactggcgccttcgtcagagatgccgacatgttcgatcactccgctt	DADMFDHSAFGLPAFEAAAMDPQQRVLLEVSLRAF
	tcggcctgcctgccttcgaggccgccgccatggatcctcagcaacgcgtgcttttggaggtctccctgcgcgccttgctaacgctgggttgaatcgagaaacgctca	ANAGLNRETLTGSRVGVYVGAMNFDAAFKSSQHA
	ccggttcccgtgtgggcgtctacgttggcgcaatgaactttgacgctgcgttcaagtcgtcacaacatgctggggaggcccacggcgccgcagcagccgctccgtc	GEAHGAAAAAPSLLSSRLSNVFGLQGPSLTIDTACS
	gctcctgtccagccgtctttccaatgtcttcggcctacagggtccttcgctcaccatcgatacagcgtgctcttcaagtcttgtggcactggaaatggccgccgaggcatt	SSLVALEMAAEALRQRKIDRALVASVNIISSTRTYVA
	gcgtcaacgcaagatcgaccgtgctctggtagccagcgtcaacattatctcctccacccgcacttacgtagccgaatgcgccgccggcatgctctcacgccggggt	ECAAGMLSRRGICAPFDNQADGFVRGEGAVAIVLQ
	atctgcgctcctttgacaaccaggccgacggtttcgtccgtggcgaggtgctgtggccatcgtgttgcagcgcctagaagatgctaccggtgaggtacacgctgtg	RLEDATGEVHAVVNGIATNHNGRTAANLTSPSPSA
	gtcaatggcatagccaccaaccacaatggccggaccgccgccaacctcacctcccccagcccatctgctcaggagtccgtcattcgcgccgcccttgctgatgctg	QESVIRAALADAAVDPAQVGYVECHGTGTKLGDPIE
	ctgtcgaccctgctcaggtgggatacgtggaatgccatggcactggaaccaaactcggcgatcccatcgaaatcagcgcgcttcagaacgtctttgacggtccgtct	ISALQNVFDGPSSLPQQPLFVGAAKATVGHTESAA
	tctctcccgcagcagcccttgttcgtcggcgcggccaaggccacagttggacataccgagagcgctgccggcctagtgggcttgctgaaggctgttctggttctgaa	GLVGLLKAVLVLKHGKVPPLSHISRINEHLQLEGSRL
	gcacggcaaagtgccgcctctctcgcacataagccgcatcaacgagcatcttcagctggaaggctcgcgtctagagcttccgaaggcatggacgcagctggata	ELPKAWTQLDSSNPIAGVSSFGFSGVNCHAVLRRP
	gctctaatcccatcgccggtgtatcctcattcggcttctcgggtgtcaattgccatgctgtcctccgccggcctaatgcagtatcactacagtcagtcaggagtatagcac	NAVSLQSVRSIALPEPKLNRVWFPLTSVHSAAGVEL
	taccggagcccaagctgaaccgtgtctggttccccttgacatctgtgcactccgctgccggagttgaactgacgcaagatgtaacattcaaacctggacaagtgtac	TQDVTFKPGQVYPDNGSSVQNTAFSASFRHGLSAA
	ccggacaatggctcatccgtgcagaatactgcattctcagcatctttccgccacggtctctccgccgctttcttggcggagaaattctgcggcgccagccgtccgcag	FLAEKFCGASRPQALLLSLELMSAVMQPQGVNKSA
	gccctgcttctcagcctcgagctcatgtccgccgtcatgcagccgcaaggcgtcaacaagagcgctctcgccctccaatctggcgttctgagagccccttacgacat	LALQSGVLRAPYDITSDSQSGLQAHTITDGLLGVRC
	cacctcagactcccagagcggccttcaggctcataccatcactgacggcttgcttggcgtgcgatgcgcgtttggctcgctgtcggccatcttggcaacggcgaccgt	AFGSLSAILATATVAPVAAAIPNQLNVQPGDDVLTNS
	ggctcctgtagcagcagctattccgaaccagctgaatgtgcagcccggcgatgatgtgctgacaaattcccacctcagccccgccttctaccctccgggcgtcagtg	HLSPAFYPPGVSGVHHDRSVRRVRFTFTRPKDQLY
	gcgtgcatcatgaccggagcgtgcgccgcgtccgcttcacattcaccaggcccaaggatcagctctacatctgcccgagccatgcaaacattatccaggccgcttg	ICPSHANIIQAACDVVVSLQGSEHRNPLVVGFDSLV
	tgacgttgtcgtctctcttcaaggctcagagcatcgcaaccccttgtcgtcggtttcgactccctcgtaactgtacccggctcgtcaaaattcacggtcgatatcgccgt	TVPGSSKFTVDIAVRSSGDGELCLDILAIAEESKSGL
	ccgttcttccggcgatggagagctatgcttagacatcctggctattgccgaagagagcaagagcggcctgcatgtccgtggccttcgtgtcgccctggcatccgcgg	HVRGLRVALASADPAAISLTKLDLRPVLDLYELSQST
	atcctgccgcaatcagccttacaaagcttgacctccgccccgtattggacctctatgagctttctcaatccacaagcggtcgtgcctggcgcgtcgatggacccaaa	SGRAWRVDGPQNRSNGFGSVNSISIDQLQADRMA
	accgctctaacggtttcggcagcgtcaactccatttccatcgatcagctgcaggccgaccgcatggccgcagacgacgttgcagtctactctgccacagacctttctg	ADDVAVYSATDLSATAEVLEKWATKANNRHLWILC
	ccactgcagaagtcctcgaaaagtgggcaaccaaggcgaacaaccgccacctgtggatcctctgcagcagagaaacccacctgatgcctgcactccggctct	SRETHLMPALRALVQTFRLEHPRCSIGIVTGEGHVE
	cgttcagacgttccgtctagaacacccacggtgcagcatcggcatcgttacaggcgaaggccatgtggaagatttggtgcctcgagtgcctgtagagctcggcccag	DLVPRVPVELGPEVHIAHGGRLHLPCLLPAAPTSTF
	aagtccacatcgcgcacggcgggcgtcttcacctcccgtgcctgcttcctgctgctcccacgtcaacattccagcagcaggcacgcacaggtcccagcctgatgca	QQQARTGPSLMHGTFVITGGTGGLGLATAEALLEA
	cggcactttcgtcatcactggcggaactggcggtcttggtctcgctacggccgaagcactcctcgaggcaggcgcggcgcatgtggcattactctcccgctccggac	GAAHVALLSRSGRATKGSNERLSRLMFDEPSRLSV
	gggcgaccaagggatcgaacgagcgtctttccagactgatgtttgatgagccgagccggctgtcggtccaccaagcagatgtgaccgacgaacgacaagtcacc	HQADVTDERQVTDVVRQLQLRRRSITGVFHAAGQL
	gatgtggtgcgccaactccagttgcgacggaggtcaattaccggcgtcttccacgcagccggccagctttccggggaagaggagggcgtcaccaccaagggatt	SGEEEGVTTKGFNGASFERHRAVKVAGLRTLITALR

	caacggcgcctctttcgagcgtcaccgcgccgtcaaagtcgccggcttgagaacactaataacagctctaaggaacaccagcatcgaagtcgtcgtcggcttctcg	NTSIEVVVGFSSTSALLGLRHQAEYAAANAAMDAVL
	tcgacgtcggcgctgctcgggctgcggcatcaagctgagtacgcagcggcaaacgcggccatggacgctgtgcttctggatgaggagcaacagcagagccgtta	LDEEQQQSRYQSATRYVSLQIDAVASVGFAARTLS
	ccaatcagcaacgcgctacgtctctctccagatcgatgccgtggcatctgtgggcttcgcggctcggacgctgtccagcggcgcggagcaagaaggaatgatgcc	SGAEQEGMMPLQDFKRAIVQVAQSASGVVSLIDNR
	gctgcaagacttcaagcgtgctatcgtacaggtcgcccagagcgcatcaggcgtcgtcagtctcatcgacaaccgccgtgctgcggtgtggctatctcgtcagggg	RAAVWLSRQGVKRTAAQPSPNGPFPDTKGKEADG
	gtcaaaaggacggcggcccagccgtcgccgaacggcccgttcccggacaccaaaggaaaagaagcagatggcgccaaagtaacagagagccccgtcaat	AKVTESPVNGSGPVPVLIRSAFEEVTGHSLESDSTP
	gggtctgggccggtgcccgtgctcattcgctctgcattcgaggaggtgacgggccacagtctcgagagcgattccacgcccttctttgatgccggccttgactcgctaa	FFDAGLDSLTSVSFRHVLEDRLGCTLPTSIAFDYPNL
	cttccgtctctttccgccacgtgcttgaagaccgcctgggttgcactctacccacgtcaatcgcgttcgactacccaaaccttaacaaattggtgtccttcctgcataagg	NKLVSFLHKETAVVTGSLQEAPKSINRTTAAISGAAT
	agacggctgtcgtgaccgggtctcttcaagaagcacccaagtcaatcaacaggaccacagctgctatttccggtgctgcaacttctgcccaagatgacgacgatat	SAQDDDDIVVTGMDCVFPGADGIEAFWDVLMSGKD
	cgtcgtcacgggcatggactgcgtcttccccggcgccgacggcatcgaagccttctgggacgttctcatgtcgggcaaagacatggtcgcccctatcagcccggac	MVAPISPDRLDLEGYNKHLLQTEGEEAAVTIKEAAM
	cgactcgacctcgaggggtacaacaagcatctgctgcagaccgaaggcgaagaagccgcggtgacgatcaaagaagcagccatgctatcgtacgaccaagtc	LSYDQVFSFDHAFFGMSRRESEATDPAQRLVLRTC
	ttcagcttcgaccacgccttcttcggcatgtctcggcgcgagagcgaagccacggacccggcccagcggctcgtcctgcgcacctgctggcaggccctgcaccgc	WQALHRAGFTRDHLEGKQVGVFVGAGPSEWSKAL
	gcgggattcacccgcgatcacctcgaggggaagcaggtcggcgtcttcgtgggcgccggccccagcgagtggagcaaggcgctgggcttccgcaacgcgctg	GFRNALTGAGSASSLLANRVSYCFGLRGPSLVVDT
	acgggcgcgggctcggcgtcgtcgctgctggcgaaccgcgtgtcgtactgcttcggcctgcgcgggcccagcctcgtcgtcgacacggcctgctcgtccagcctcg	ACSSSLVALHLARAAIRRGECEAAVVCGVQVHLHP
	tcgcgctgcacctcgcgcgcgcggccatccggcgtggcgagtgcgaggccgccgtcgtgtgcggcgtgcaggtgcacctgcacccgtcatctttcgagcaggtctc	SSFEQVSRSKMVSPSDNRSKPFDAAADGYGRGEG
	gcgctcgaagatggtctcgccgtccgacaaccgctccaagcccttcgacgccgctgccgacggctacggccgcggcgagggctgcggcgccgtcgtgctcgtca	CGAVVLVRARDAVDVPPLAVLKGSAVNSDGRSASL
	gggcgcgcgatgctgttgatgtaccgcccctcgcggtgctgaagggctcggcggtcaattcggacggtcggtcggcgagcctgacggcgccgagcgggccctcg	TAPSGPSQVDVIREALADACLEPGHVAYVETHGTG
	caggtcgatgtcatccgcgaggcgctggcggacgcgtgcctggagcccggtcatgtcgcgtacgtcgagacgcacggcactggcacgagtctgggcgacccga	TSLGDPIEFNALKSVFGSPSSSSSSSSSSSPRRQQ
	tcgagttcaacgcgctcaagagcgtcttcgggtcgccgtcgtcgtcgtcgtcgtcgtcgtcatcgtcgtcctcaccgcgaaggcagcagccgctggtcctcggcgcgc	PLVLGALKGQIGHLEGAAGIASFIKAVLALNHGVAPP
	tcaagggccagatcggccatctcgagggcgcggcaggcatcgccagcttcatcaaggccgtcctggcgctcaaccatggcgtcgcgccgcccaacgtcaacttc	NVNFRRLNPAVVGDGFDFVLPTRPEPLAPVHHHSH
	cgccgcctcaacccggccgttgttggtgatggatttgactttgtgcttcccacgcggcctgagccccttgcgccagttcatcatcattcgcacgacaggggtagtagtac	DRGSSTLAAGISSFGFGGTNAHVVVTAAPATTAAEE
	cctcgcagcaggcatcagctccttcggcttcggcggcacgaacgcccacgtcgttgtgacggctgcccctgcgactactgcggctgaggaaacaagcttcagcag	TSFSSAADDGPSPAPLRRLHAPHRFAPTLVEPPAR
	cgccgccgacgacggaccgtcgcccgccccgctacgccgtctccacgcgccgcaccgcttcgctccgaccctcgtcgagccgcctgcccgctcgctgcatcccc	SLHPRRGGRRQQSIHATSPFIGAPASPATGVASLAK
	gtcgtggtggccgccgccagcagtcgatccatgctacttctcctttcatcggtgcgccggcttcccctgccaccggtgtggcgtctctcgccaaggtctcgtcgccgcc	VSSPPATTAAAIPSRLSSPSPLSQGIRSRGGGTFQV
	ggccactaccgccgccgccattccttctcgtctctcctcccccttcgccgctttcgcaggggattcgcagccgcggtggtggtaccttccaggtgcggtttcagagcggtg	RFQSGGGRGTRAMTTAVPVVSTSTTTMMARSSGN
	gtggtaggggaacgagagccatgaccacggctgttcctgtcgtttcgacgagtacgacgaccatgatggcgcggagtagtggtaatgaagggggtgcgggtgttg	EGGAGVAGSSSSTTAVHRQFRFKFSERMG*
	ctgggagcagcagctccactacagcggtgcatcggcagttccggttcaagttcagtgagaggatgggttga	
lasD	atggccgaatctgaagatgtcggctacatgctggccaggctgaacagcatggccttcctcgctgcagacgcctacaacaatcagcagcacggacgcacacaag	MAESEDVGYMLARLNSMAFLAADAYNNQQHGRTQ
	atgtgcaatctctgttcgattccgtcttccaccttgattctatccggaacactttgagccagtcgcccactgatctggaatacgtggtcgacatcggagaagacgagaaa	DVQSLFDSVFHLDSIRNTLSQSPTDLEYVVDIGEDE
	cttgctgtctatggaccggatgacggcgagaatggcccgccgatgccgctcttgactaccggcataatgggttctgcacttcccgtggccctggaggcagatgccaa	KLAVYGPDDGENGPPMPLLTTGIMGSALPVALEAD
	ctaccacgctgaattggtcccggatgaaatagttgagatgctgtgcggggcagcagcggacggagaagaagtctccctcgacgagcgtctcctcgcctacatcttg	ANYHAELVPDEIVEMLCGAAADGEEVSLDERLLAYI
	accgcatcatcgcccacgcttttgctggctccacgacggattggagtgcagtgggccttgttgcttttgaccaccagaatttccatgcgcgcaaccttctcagtctgcaa	FDRIIAHAFAGSTTDWSAVGLVAFDHQNFHARNLLS
	cagcgcctctcgcgtggtctttcctctccccaattcgagcttcttttcaacatagactctcgcaaaaatgccagacgggatagcggagaagtggccatatctgtagcac	LQQRLSRGLSSPQFELLFNIDSRKNARRDSGEVAIS
	tccatgctatgctccctggcgcagtatctcgcggatccgagcgacttttgatctggagcgcagccttaagggtcgctgctgctacgccagccgacagaaatacccag	VALHAMLPGAVSRGSERLLIWSAALRVAAATPADR
	caagctgtgcctgccctccggccccgatacgtggactgggtgcacgtcggcgacgtaccttcatcgctaccaactcacctgcaccacaacggcagagctcgccgc	NTQQAVPALRPRYVDWVHVGDVPSSLPTHLHHNG
	catttcctcctccaaggatccgcgagtggactgatcgacgacctgcacacgaaactgcaggccaccagcttggatgtccagcgccacgagatcaaatcgttctctc	RARRHFLLQGSASGLIDDLHTKLQATSLDVQRHEIK
	caaccacgctactccactcctggcaggattccaccaccatcatcgtcgacctcgcctccatcgccaccgctgaaaacctccccagagcgccaagtgacccatgtc	SFSPTTLLHSWQDSTTIIVDLASIATAENLPRAPSDP
	aggagctcctcgagcggctggcgacgacctcggccggcgcccgggtcatcctggtcacgcaaggcgtgctcgccgtcaccggctacgaggaccggcaagtcg	CQELLERLATTSAGARVILVTQGVLAVTGYEDRQVE
	aggcggacaacgcaggcgccgcggagcttgtgagccgctacgccagctcgaagcagcagctcgacgtgtggcaagtcgacgtcgacgcacgcgcagcgacc	ADNAGAAELVSRYASSKQQLDVWQVDVDARAATS
	agcgagggcgccgacgcgctctgccggttgctgttgctgccggacgacgatgaaagcaacatcttcaagagcacccgcggcggaatcgtggctctgcgcgacg	EGADALCRLLLLPDDDESNIFKSTRGGIVALRDGSK
	gctcgaagctcttcgagcccgcgtgggttgagtctgggcgcgtggtggacgctgctggcgacgaggaggcgcttctcaatgccccttttgcggcctgcttccgggag	LFEPAWVESGRVVDAAGDEEALLNAPFAACFRERE
	agggagcaggtgttcgagatggagcggctgctttctgacgcctgcacggaggctctgcggtcggttgtcgaccctgcgacgtttgagcgggcgttcgcgaagctgg	QVFEMERLLSDACTEALRSVVDPATFERAFAKLDRL
	accggctggcggagcagtctctggttgctgctgttgcctcgctgaaccagcaggacctgcagcactctgtgccagcccacctcaagcagctgtgtgagcggtattcc	AEQSLVAAVASLNQQDLQHSVPAHLKQLCERYSRQ
	agacaacagtactccggtcactcggccatcgcggatgccggacgactacgggccgagctcgaggagctctatacggaatgcccgcccctacgcccctcagttgc	QYSGHSAIADAGRLRAELEELYTECPPLRPSVALLG
	gctcctaggaaatgtcctcggacggcatcgtgacgtccttcttagccgtctcgcgcccctcgatgtcctcttcggcaagaatgatgcagcagcgaacaccgccgccg	NVLGRHRDVLLSRLAPLDVLFGKNDAAANTAAEQA
	agcaggctggaggagcggcggcggaggtctacgacgaagccgtcctcgcacgcgtccacaaacgcgtcgtcgaacgcaccatctcccacctctgcaccctcgc	GGAAAEVYDEAVLARVHKRVVERTISHLCTLASRCN
	ctcccgctgcaaccgccgcctgcgcatcctcgaagtcggcgccggcaccggcggcaccgctttgcccctcctccgcctcctccaacaacaccacccaggcctctg	RRLRILEVGAGTGGTALPLLRLLQQHHPGLCAEYAF
	cgccgagtacgccttcaccgacctcggcgcgcacttcgtccacgccttcgcccggcgccaccgcgcctccttcccctccatcccccctccgcacgcagacgttcgatg	TDLGAHFVHAFARRHRASFPSIPLRTQTFDAARHPS
	ccgcccggcacccgtcgctgcaaggcttcgcggcgggcgagtacgacgtcgtcgtctgcgtcaacgtgctgcacgccacgccgagcgtggccgcgacgctggc	LQGFAAGEYDVVVCVNVLHATPSVAATLANVHALLA
	caacgtccacgcgctgctggcgccgggcggcgcgctcgtgctgcaggagatcacgacgcaggcgtcggcgttcctggacgcgacgttcggcttgaccgaggggt	PGGALVLQEITTQASAFLDATFGLTEGWWAAEPDD
	ggtgggcggccgagccggacgatgtgtttcgccccggtggatatccgacgctgccgccgcgcgcgtggcagcaggcgcttggaaggacggggttcgcttggtcg	VFRPGGYPTLPPRAWQQALGRTGFAWSACTPAGG
	gcgtgtacgcctgcgggtggggcgttggatcagcaggcggtgtttgtggcgcagaagggcgtggcgtggggcgctggggatggtgtgggttctgcgatgcgcgtgc	ALDQQAVFVAQKGVAWGAGDGVGSAMRVRGEES
	ggggggaggagagtgagggctggtatgtgcttgctggggagcagggcgatctcgtgacagccctcgctgcgcaggagcttgttgggacgtttgatgcccggcggtt	EGWYVLAGEQGDLVTALAAQELVGTFDARRLIWVR
	gatctgggtccgctttggtgttgggggtgcggcttcgtacgcgcaggaggaggaggaggaggaggatcgagcattccagctggcacacgaagcaggcgtggac	FGVGGAASYAQEEEEEEDRAFQLAHEAGVDLQVFE
	ctccaagtttttgagggaacgcgtggaagcaagatcatcaccccagaggtgcagtctttgttgaagggcggcgatgttcgaggtgtggtgtgggtcacgaaggatag	GTRGSKIITPEVQSLLKGGDVRGVVWVTKDSGSGP
	cggcagtggtccgcagtcctctacgttttcagcattgcagcaacttcacgtcgacttggaccgtttggataggatcccgaggcgactggtcatccttcatggcattggtta	QSSTFSALQQLHVDLDRLDRIPRRLVILHGIGYEGN
	tgagggcaaccaccccccttcaggatccagtggaatcattcgtgcggttccgccgtggccgcttgggcctaccaacgctgttggctcggtggcacttgggtgaggaaa	HPLQDPVESFVRFRRGRLGLPTLLARWHLGEESGL
	gtggccttcccagcgcagttgtcaaatcgaccatcaccgcagccatccgttcttgcgacttccgttccaagaaagttgccgtcctcgactttattggcacatggaaagc	PSAVVKSTITAAIRSCDFRSKKVAVLDFIGTWKAAQN
	ggcccaaaatcaaccgcaaaccacgaagactatagcaactaaagcctctgtggcgggtagaactgtctttggagccagcaaatctgcgcagatcaccaacggtg	QPQTTKTIATKASVAGRTVFGASKSAQITNGVKKPT
	tgaaaaaaccaacggcaacaagggcgagcaacgtaccctcaatcgacacgatcatagccgttgtcatcgccgaggccgaggcactgctgggatcttctggatca	ATRASNVPSIDTIIAVVIAEAEALLGSSGSVDADDPL
	gtagacgcggatgaccctctcatgcaggcagggattgactcactggctgtcgttgagttgcgcgcggggctcaagagattatttcccctggctcctttaccgagcacct	MQAGIDSLAVVELRAGLKRLFPLAPLPSTYIYEYATA
	acatctacgagtatgctactgctagagctatagctgaggccattgcagctatgctggcagaagtagaagaagagcaagaagaggagcgcgcagaagttgcacg	RAIAEAIAAMLAEVEEEQEEERAEVARAAPPLTTAAT
	cgctgctccaccattaactaccgcagcaacggcggtgagaaacatctccatacccgcaacagttcaatcgcccttcccaatcaccgctctgaatggcgtcgccgat	AVRNISIPATVQSPFPITALNGVADTLSEDRGNKVAV
	accctctctgaggatagaggcaacaaggttgccgtgattgccatggactgtcggatgccgggcggcatgaactcgagcgatgaattctgggagggtctgaagctcg	IAMDCRMPGGMNSSDEFWEGLKLGRDAMVDIPLS
	gaagagacgccatggtcgacatcccactatctcgttttgacgtcgacgaggtgtacgacggcgacgtagacgcactagccaagggcaagacatatgtgcgtaga	RFDVDEVYDGDVDALAKGKTYVRRGGFLDRAEHF
	ggaggcttcctggatcgggcggagcattttgaccggtcccgcttcacgattcccgaggtgcaactccaacagatggatccacagcaacggatcctgctcgaaacg	DRSRFTIPEVQLQQMDPQQRILLETVDSALTDAGYS
	gtcgactcggccctcacggatgcaggatattcacggaaggatatggcagggagggatgcttctgtttacatcggcgtctcttgcaaggactgggcgaaggtggctcc	RKDMAGRDASVYIGVSCKDWAKVAPLDGAFIGTGA
	cctagatggtgctttcatcggcactggagcagcagcggccatcgtgtcgaaccgaatttccttcgagcttggcctgagcggtacgtccatgagcatcgacacggcgt	AAAIVSNRISFELGLSGTSMSIDTACSSSLVAVDLAV
	gctcttccagtctggttgctgtcgacctcgctgtccaggcgctgaggctcggaaggactgacatggccgtcgccggcggtgtcaacatgatcctccacccggaccctt	QALRLGRTDMAVAGGVNMILHPDPYVVFCQNKMLS
	acgtcgtcttctgccaaaacaagatgctctcgcccgacggcctctgcaagacgtttgatcgcgatgccaagggatacgtccgctccgagggctgcggtgtcgtcattc	PDGLCKTFDRDAKGYVRSEGCGVVILKRLSDALRD

	tcaagcggctctcggatgcgctgcgcgacgacgatcccgtgctgggcgtcatcaccggctcgtgttccaaccaggacggccggtcaactaacatcagcgcgcctt	DDPVLGVITGSCSNQDGRSTNISAPSGKAQQEVVL
	cgggaaaggcgcagcaggaggtcgtactgagggctctccgtgacgcaggccggcggccggacgaagtcgacttcgtagagctccacggtaccggcaccgctc	RALRDAGRRPDEVDFVELHGTGTALGDPVEVHSLK
	tgggtgaccccgtcgaggtgcattcgctcaaggccgtcttcggagcgccgacgcgcaagcgaaaggctgctgcaacagacgtcgtccgcacccgtccgctgtac	AVFGAPTRKRKAAATDVVRTRPLYLGAVKTNIGHPE
	ctaggcgctgtcaagaccaacatcgggcatcctgaagcagcagcaggtatcgcgggcttgatcaagtgcgtgaatgtggtgcagcaacgccaggttcccccaaa	AAAGIAGLIKCVNVVQQRQVPPNIHLENINPGIEFEG
	catccacctcgagaacatcaacccgggaatcgaattcgaaggcttcgaggtggcacggcttccggagcgcgtcgaagacgtcggccgcgggccggacagagc	FEVARLPERVEDVGRGPDRAVVAGCSSFGFGGTN
	ggtggtggccgggtgctcgtcgttcggctttggcggtactaacgttcatgtcattgtcgaatctccgccagcgcaggcacgcagtcgtgcaccaaagccggtcaaga	VHVIVESPPAQARSRAPKPVKKEIEKIPDVDVPRRA
	aggagatcgagaagattcccgatgttgacgttccgcgcagggccaggactgatgatgaggaaaagcttatcatcgccttcactggacaaggcagccagttcgcct	RTDDEEKLIIAFTGQGSQFASMAKELYRDDSVFAAT
	ccatggccaaggagctgtaccgcgatgattccgtgttcgccgcaactgtagatcgctgcgtcaagatttgttccccgtggattgcgccagaagacctacgcaatgcg	VDRCVKICSPWIAPEDLRNALLKPNADGTEAIKPAH
	ctgctgaagcccaacgcagatggaaccgaggccatcaaaccggcacatgcctctcaagcgagtctgttcgtgatggagtacgcaatctatcagaccctcgtggag	ASQASLFVMEYAIYQTLVERGLQADVVLGHSIGEIVA
	cgtggtctgcaggccgacgtggttctcggccactcgattggcgagatcgtggcatcagtagtgtcgggctgcttgaacctggaggatggccttcgcctggtatgtcag	SVVSGCLNLEDGLRLVCQRGIIMEQADVEGTMYAIM
	cgtggcatcatcatggaacaagcagacgttgaaggaaccatgtacgccatcatgctgccgcacaccactatccaggccgccatcgatgacctgagactaggtga	LPHTTIQAAIDDLRLGETASVAAINGSNLTTVAGLHE
	gacggcatctgtagcagccatcaacggtagcaatctcaccaccgtggctggtttgcacgagtcgctgcagcgtttgctgagccacctggggcaaggcgtcaccag	SLQRLLSHLGQGVTSAPLKVSRPFHSPLLRPCMQA
	cgctcctctcaaggtgagccggccattccactcgccgctgctgaggccatgcatgcaggcgtttgcggagcgcatcggtggccttaacgccggtgcgcctactacc	FAERIGGLNAGAPTTVRFWSTVTAAEVAAAPDVNH
	gtacgcttctggtccacagtgacggccgccgaggtcgctgccgctcctgatgtcaaccattgggtaagacacgctctgcaagctgtgcttttcgagccaaccgtgcga	WVRHALQAVLFEPTVRAVSEAFAGRVRLLEVGPKS
	gcagtgtcagaggccttcgcaggcagagtgagactgttggaagtaggtcccaagagcgttctctacgacatgatgcggcggtgggcaagggtgccgccaggact	VLYDMMRRWARVPPGLPAPCALIGSSSVATKSGITL
	gccggcgccgtgcgcattaataggctcatcaagcgtagccacgaagtcagggatcacgcttgccacactccaggcggcagtgaaggccaatgagcagcacttc	ATLQAAVKANEQHFDKRDDLFEDGEEGECDPSGS
	gacaagcgcgatgacctcttcgaggacggtgaggagggcgaatgcgaccctagcggtagcaacattgtgctagtaggtcaagggcgccgcgaagggttcttccc	NIVLVGQGRREGFFPWRRGYHALITSPEILQLDAET
	ctggcgtcgtggctatcacgctctcatcaccagtcccgagatactccaattggatgcagaaacgcgtgccgtgcgcttcagagttaaccaccgtactatccagctcgt	RAVRFRVNHRTIQLVADHVVRGRPILPGAAVFDLAL
	cgccgatcatgtcgtgcgagggcgcccgatcctacctggggctgctgtcttcgacctggcgttgggtggctaccaagtactccacaatctgcaccctctcgaggcgct	GGYQVLHNLHPLEALPMELRDAVISRPVVLAADME
	gccaatggagctacgtgacgccgtcatctcacggccggtggtgttggccgcggatatggaaccaccgtggctacagctgacctacaacaacactgatggaacggt	PPWLQLTYNNTDGTVQVMSAADPSHEFVEHARAT
	gcaggtaatgtcggcagcagacccatctcacgaattcgtcgagcatgcacgcgccacagtagtccgcgtcgagcccacacagcgccatcaacgcgtgcggctc	VVRVEPTQRHQRVRLATIQHRCTDAVDVTDLRSKL
	gccaccatccaacaccggtgtaccgacgccgtagacgtcactgatctccgttcgaagctcgctgctcgcgggttggcgtacggcccgcagttccaagccatggtga	AARGLAYGPQFQAMVSAQRRDGECLAELRLPASG
	gcgcacaacgacgcgacggcgagtgcctggccgagctgcggctgccggcatctggctcttccggttatgtcgtccatccaggcgtgcttgacagcgccatgcaga	SSGYVVHPGVLDSAMQTCAATLSSMAGKDAFAVG
	cgtgtgccgcaaccctgagctccatggccggaaaggacgcctttgccgtgggctctctcgggcgtctgcgcgtgcgttccttcttgggcgagcaccgcagcaatgct	SLGRLRVRSFLGEHRSNAEVRVWSRVVSSTADCYE
	gaggtgagggtgtggtcccgtgttgtctcctcaactgcagactgctatgaggccaacgtcgatgtttgcgacacactgggcaacgtgctgctgtcactcgaagcccttc	ANVDVCDTLGNVLLSLEALRLVRVSSQVALVASSRC
	gtctggtgcgtgtgtcgtcgcaggttgccttggttgccagctcgcggtgtctgtacttgaccgaatgggcgcctctgcagccagctcctggacagagcctttgcgcggc	LYLTEWAPLQPAPGQSLCAASAARLPEKTAIDVDQV
	atccgccgctcgtctaccggagaagacggccattgacgttgatcaggtattgggcattgtcacgggtactaacttcgcgaggcgtggtcctgttgctgctctccgcgat	LGIVTGTNFARRGPVAALRDALRTACGAGSLMDVV
	gccctccgcactgcgtgcggcgctggctcccttatggatgttgtgttgccctttacgacgtcggagaagccaattcatacacttctctacgtttgttccccaaccgaaaca	LPFTTSEKPIHTLLYVCSPTETAYTITSQLCRLFQAIL
	gcatatacaatcacctcacagctctgtcgtctgtttcaggctattctcgacacacctttcctttccgaactccgttcagtcgtcgtagttacgttgggcagccaattcgtgctc	DTPFLSELRSVVVVTLGSQFVLEDDACSSPESSAA
	gaggacgacgcttgcagctcacccgagtcctcggccgcatggggtctgctacgatctgcgaggctggagctcccacagcttcgccttgtcacgctcgacgtcgacc	WGLLRSARLELPQLRLVTLDVDPTASAWKQALAVL
	ctaccgcgtccgcgtggaagcaggccttggcagtgctcgacgccgccctcgacgcgtctgaggacgaagtggcctggcgtgatgggcgccgctacgtgccccga	DAALDASEDEVAWRDGRRYVPRLTDPGFQGTEAE
	ctgaccgatccaggattccaaggaacagaagccgaggccatcgatttgatgaggcctgcgttcgatttctccatcgagactgctctgcccgcgacgctcgacttgaa	AIDLMRPAFDFSIETALPATLDLNTVVPVDVDDAELA
	caccgtggtgccagttgacgtggacgatgccgaactggcacgcgttcgcacgggctacgccctcgtcaactccctggcgctgcagagcctcatccagggcgctgc	RVRTGYALVNSLALQSLIQGAASVSSEADVVPLVQQ
	ttccgtctcctccgaggcagatgtcgtgcccctcgtccaacagctgtggagacgataccgcaacatgcccgccagcagcgtccagtcagaactggtgccacctgac	LWRRYRNMPASSVQSELVPPDLGDASTWIDSAIKQ
	ctcggtgatgcgagcacctggattgattcggcgatcaagcaatacccagaggttacttccgagctggccatgctcaagtccctgtacggccaacacggcgccgtctt	YPEVTSELAMLKSLYGQHGAVFRGERDPLELLFRT
	ccgcggcgaacgcgatcccctagagctcctcttccgcaccgatcgccacaccgagaccgagggcatctactccgagtccttctacgcccgctactacaacagcgt	DRHTETEGIYSESFYARYYNSVVTYVLSALVQQQP
	cgtgacgtacgtgctgtccgccctggtgcagcagcaaccgcaaaaccgccgcaaactccgcatcctcgaggtcggcgcgggcagcggcagcacgacccgtag	QNRRKLRILEVGAGSGSTTRRILETAAALDIEVDYHF
	aattctggagacggccgccgcgctcgacatcgaggtcgactaccacttcaccgacatctccgagaccttcatccgcaaggcttccagcagcggcaagctgtccgc	TDISETFIRKASSSGKLSASPGSSARVTYGVFDAER
	gtcgcccggcagcagtgcccgcgtcacgtacggcgtgttcgacgccgagcgcgacccccgtctccagggcttagccagctcgtccttcgacctcatcgtcgccgtc	DPRLQGLASSSFDLIVAVNVIHATRVLRETMANLRQ
	aacgtgatacacgcgacgcgcgtgctgcgcgagaccatggccaacctcaggcagctgctgcggccgcggggcgtcgtggtgctctccgagttcacgcgcccga	LLRPRGVVVLSEFTRPSPMTDASFGMTEGWWRFD
	gcccgatgacggatgcgtcgttcggtatgaccgagggctggtggcggttcgatgataaggatttgcggcctgagtacccgcttatggaggcggcgcggtgggagg	DKDLRPEYPLMEAARWEGLLLATGFDRVRTLENPK
	gcttgctcttggcgacgggctttgatagggtgcggacgctggagaatccaaaggctggggagtcgcaggctgtcattgtcgctcaggcttccgcccaggaggatgat	AGESQAVIVAQASAQEDDDSRDHEREERGERGVYI
	gattcgagggaccatgagcgtgaggagcgcggagagcgcggcgtctatatcatctctggtggcaccggtggtatcggccttctgaccgccatcgtcttgatcgagc	ISGGTGGIGLLTAIVLIERGCRKVYLLSRSGKVSSNS
	ggggttgccggaaggtctaccttctctcccggagcgggaaggtgtcttctaactcaacccacttgtgggacagactgatggccgtggctcagccggaccaggtggtc	THLWDRLMAVAQPDQVVKVTCDVSNGVQVAEVFA
	aaggtgacatgtgatgtcagcaacggcgtccaggtggctgaagtctttgccgggatcagagaacgtggcgaatgggttgaaggcatcatccattcggctggagcg	GIRERGEWVEGIIHSAGALRDAMLRNQTPATISTVF
	ctgagggatgccatgctccgcaaccagactccggctaccatctcgaccgtgttcgccgccaaggtcgaggctctgcgccatctccactcttgcagtctcgacgagcc	AAKVEALRHLHSCSLDEPLLRQFVIYSSCAALLGSP
	tctgctgcgccagtttgtcatttattcctcctgcgccgcacttcttggctcgccgggccagagcaatcacgccgcggcgaacgcattccttgactccttcactgcctaccg	GQSNHAAANAFLDSFTAYRRTCGLPALSIQWGTVS
	ccgtacctgcggccttcctgcgctgagtatacagtggggtactgttagcggcattggagaggcggctcggaagggcgcaaataagcttgccgtcaagttcggccac	GIGEAARKGANKLAVKFGHGQVSETEAESFLKAVF
	ggccaagtgtccgagacggaggccgagagcttcctgaaggccgtcttttcgtctgaccattctcgcacactgtcaggaccgatgtgcatctcacccttcaactggacc	SSDHSRTLSGPMCISPFNWTTFTKMRTRVPSLVSA
	accttcaccaaaatgagaactcgcgtgccatcgctagtgagtgcattctctgtcattcagaaggctgccccgtcccgtcccgcgggcactgcaccggcaacaaagt	FSVIQKAAPSRPAGTAPATKSVPVSNTRGNAAVSLL
	ctgttcctgtttccaacacacgtggaaatgccgccgttagcctactagagacgatacaaagctgcgtcgaagctacggttggtacgagaatgccgagcgcttcgtcg	ETIQSCVEATVGTRMPSASSTFFDVGMDSLSAIEFR
	actttcttcgatgttggcatggactctctgtcggcaattgagttccgtaaccgtctacagtcgagcctgggcgcgggtgtcaaactttcggcatccttggtcttcgactacc	NRLQSSLGAGVKLSASLVFDYPTLARLQEYLEGLPE
	ccacgttggcgcgactacaggagtacctcgaaggcttgccagaactcatgggcattatgaatgagaccgagcagccccctgcggcgccgctggccccggccgc	LMGIMNETEQPPAAPLAPAASTVDPSPQQQLQTPA
	ctcgacagtagacccttctccacagcaacagctccaaaccccgcaccaagcatttcttgcatgatccgcagtgcgccgcaggtcgtcgacaaagttatatcgattg	PSISCMIRSAPQVVDKVISIVGMDARLPGADTIEDLW
	taggtatggacgctcggcttcccggcgccgacaccatcgaagacttgtggaccttgttgctctcagacgaggatcccttccgcgacataccattctctcggtgggatctt	TLLLSDEDPFRDIPFSRWDLDDNFYVPKSAAGDGK
	gatgacaatttctacgtccccaagtctgcagcaggagatggcaaggcagccgggagatcatacgtcaggcaaggttccttcctcagaccagaccaactggagtg	AAGRSYVRQGSFLRPDQLEWFDNSFFGISNAEART
	gttcgacaacagcttcttcggcatctcgaacgcggaagcacgcaccatggacccgaaccagcggctgctcctcgaggtctcctaccaggcgctcgccgacgccg	MDPNQRLLLEVSYQALADAGYTRDSLRGQNVGVFV
	gctacacccgcgacagcctgcgcggccagaacgtaggcgtcttcgtcggcttcatgaactacgaccaattcttcctcactgccggcggcggggacaagggcggtg	GFMNYDQFFLTAGGGDKGGASAFTASSASPAVLSN
	caagcgccttcactgcctcatccgcctcccccgccgtgctgtccaaccgcgtcagccacgtcctcggcctggaaggcccgtcgtgcaccctcgacacggcctgcag	RVSHVLGLEGPSCTLDTACSASLYALHTAIGALRSG
	cgcctcgctctacgcgctgcacacagccatcggcgccctccgctcaggacagtgcgatgcggccgtcgtggccggcgtcaacgtcatgatgtcgccggccgcctt	QCDAAVVAGVNVMMSPAAFVAECAAGMLSASQRC
	cgtcgccgagtgtgccgccggcatgctgagcgcctcgcagcggtgccggccgctggacgacggtgcggatggctttgcccgcggcgagggctgcgtcgctgtggt	RPLDDGADGFARGEGCVAVVLQRRGDAVAQGRYI
	gttgcagcgtcgcggggacgcggtggcgcaaggacgatatatccatgccgatatccttggcagttcgattggccatgacgggacgacggttaacctcacgtcgccc	HADILGSSIGHDGTTVNLTSPNGPAQARVIQRALQD
	aacggccctgcgcaggcaagggttatacagcgcgctcttcaggacgccggtgccagcccctcctctatcgcgtttgtggagggccacctgactggcacggttctgg	AGASPSSIAFVEGHLTGTVLGDPIEFSALKSVFGTPT
	gtgaccccatcgagttcagcgcgctcaagagcgtctttggcacgccgacagcccaggagaccaggtccgctcctctccacctgggagccatcaagagcaacttg	AQETRSAPLHLGAIKSNLGHLEGVAGVAGLVKAVLS
	ggccatctggaaggtgtagccggtgtcgccggactcgtcaaagccgttctttcgctccggcaccgcgtcttgcccccaatcgcacacttgaagaagctcaaccgcc	LRHRVLPPIAHLKKLNRHLDFEGFKVNVPGSGSSSV
	acctcgatttcgagggcttcaaggtcaacgtgcccggatcgggcagcagcagcgtcggcttggtgcccagctcgtcttccggcccggagctcaggtgcggcgtgtct	GLVPSSSSGPELRCGVSSFGFSGANAHIVLEAASTS
	tccttcggcttctccggcgccaacgcgcacatcgtcctggaggcggcgtcgacgagcgcgtctcagcgctgcttggtgccgcggaagcgccatgtgttctcccgcaa	ASQRCLVPRKRHVFSRNHFPLRRGGSHALLGRLPA
	ccatttcccgctgcggaggggaggttcacacgcgcttctcggccgtctgccagcaggggacggcacatggaagatgggaggcgagcatgacgtgcttatcgggc	GDGTWKMGGEHDVLIGHRLFALLEQHVVRGTPTVP
	accgcctgttcgccctgctggagcagcacgtcgtcaggggcactcccaccgtccctggggccatgcttgtagagttggccgcctccgtcgttgccggcagcggacc	GAMLVELAASVVAGSGPSPSAHLQEIVFAAPFTVSA

	ctcgccgtcggctcatctgcaggaaatcgtctttgcagcaccattcaccgtgtcggcgccccgttccaagaccatgtcgatcgcccacgaagccgacggccagttca	PRSKTMSIAHEADGQFTISWAGVDEEDDESHVTAY
	ccatttcgtgggcgggcgtagacgaagaagacgatgagtcccacgtcacagcatacggacgactcaaaggacctgcagacccccgtccgcggacgaacga	GRLKGPADPPSADERTVMLDRLRRHQDACKQAVDI
	accgtcatgctggaccggctgcggcggcatcaagacgcctgcaagcaagcagtagacatcaactccgtgtggcagaaactcgaagacgtcggcatctgctaca	NSVWQKLEDVGICYTGLFRSLQRAYTSINERAALAY
	ccggcctcttccgcagcctgcagcgcgcctacacctcgatcaatgagcgcgccgccctggcatacggtaacatcagcctcgccgagaacggcgaagacggattc	GNISLAENGEDGFLAHPALLDSAMQVCAAVVLAKLS
	cttgcccatcctgccctgctcgactcagccatgcaggtctgcgccgccgtggttctggccaagctatctacaacagaggagcagcaaagccgcagcggcaccgcc	TTEEQQSRSGTAARTFLPFAMTGVRFWAPRPRES
	gcacgaaccttcctgcccttcgccatgacgggagtcaggttctgggcgccccgcccgcgcgagagccgcaggcgcgagttcgtagcgacggcggaggtagtttct	RRREFVATAEVVSVGAAEVKARLSVFDDAGEPVFDI
	gtaggcgccgctgaggtcaaggccaggctttccgtcttcgatgacgcgggtgagcccgttttcgacatcgaagagatcgtcttccgtccggcgggagcgaagggg	EEIVFRPAGAKGGEQNQSQSQNKLPDPLHTVCWA
	ggcgagcagaaccagagccagagccagaacaagctccctgatccgctgcataccgtctgctgggcgaggatgcccgccgctcctgagctggaggagattgctc	RMPAAPELEEIAQPCVLVVEDEQDRDALASKLPPSV
	agccttgcgttctcgttgtcgaggatgagcaagaccgggacgctcttgcctcgaagttaccgccgtctgtcaccgtagcgcttctcacggagcacagagccggcga	TVALLTEHRAGEGLPQTLYYLPSLSSTAADLCTSAL
	gggcctcccgcagacgctttactacctgccctcgctgtccagcacagcggcggatctgtgcacgtccgccttggaacttatcaacactgcggtcgaggccgcatctc	ELINTAVEAASPGTSANSSLWILTRASQVSPISHPQQ
	ccggaacgtcggccaattcctcgctctggatactcactcgcgccagccaggtgtctccgatctcccacccacagcaggctgctttgtggggcctgatgcgttccgtcgt	AALWGLMRSVVLEDTRAVSIGLVDLVAGKSDDGAA
	cctcgaagatacccgagccgtaagcatcggcctcgtcgacctcgtggcgggcaaatcggacgacggagccgccgtcgcccacgaagacgttctcaagcttctgg	VAHEDVLKLLGRLKAPSRPISELAWDAETHTWFAP
	gcaggctgaaagcccccagccgcccgatctccgagctggcgtgggatgccgaaacacacacgtggttcgcgcctcagatggtgccggaccgcctctgcgagct	QMVPDRLCELDVPTMAVATTGLDSLEAVPLIEPDGF
	cgacgtgcccaccatggccgtagcgacaacgggcctggactcgctcgaagccgtgccattgatcgagcctgacggcttcgcagagctcggcgaggaccaggtat	AELGEDQVSVRVGAVGINFHDVLFAAGGLLQHGSS
	ccgtccgcgtcggcgccgtcggcatcaacttccacgacgtcctcttcgcagccggcggcctgctgcagcacggcagcagcgccctaggcgccgacttcgccggc	ALGADFAGTVQAVGSAVQHVAPGDAVFGMAEACM
	accgtgcaagccgtcggatctgcggtgcagcacgttgcgccgggcgacgcggtcttcggcatggccgaggcgtgcatgcggagcgtcgtgcgctgcagcgcccg	RSVVRCSARLVRKMPEGLSMVDGASLPTVFATAYH
	cttggtccgtaagatgccggaggggctgtcaatggtcgacggtgcttccctgcccaccgtcttcgccacggcgtatcattgtctagtcgagctggcggagctgcggcc	CLVELAELRPGQTVLVHTATGGLGHLLVSIARSHGA
	ggggcagacggtgctggtgcacacggccacgggcggcctgggccacctgctcgtcagcatcgcgcggtcccacggcgcgcaggtcgtcgtcgccacggcggg	QVVVATAGSEEKRAYLRDRLGLESRMVSTTRDARA
	gtcggaggagaagcgggcgtacctgcgcgatcggctggggttggagagccgcatggtttcgacaacgagggacgcgcgcgcgttcgccgcggaactgaagca	FAAELKHVAGKVDVAINTLSGDYIPETLRLLKPGGTF
	tgtcgcaggaaaggtcgatgtggccatcaacaccctctccggcgactacatacccgagacgctgcggctgctcaagcccggcggcacgttcatcgagacgggca	IETGKVAIWSQDKVAASRPDVVYRVVDLDITGSASV
	aggtcgccatctggagccaggacaaggtggcggcgtcgcgtcccgacgtcgtttaccgagtcgtcgacctggacatcacgggctccgcgtccgtcgagctgatgg	ELMDAFLSRLAEGVADGSVRPPPVTAFDYPAQLRD
	atgcgttcctgtcgcggctggctgagggtgtagcggacggcagcgtgcggccgccgcctgtcactgcgttcgactacccggcgcagttgcgggatgctttccgctat	AFRYVRTAKHIGKVVLRMPQATLPRTIAAANTSPGF
	gtgcgcacggctaagcacattggtaaggttgtgctgcgcatgccgcaggcgaccctgccccggacgattgcggccgcgaacacttcccccggcttctcgccgcctg	SPPEVPVAFDQSRYQEGIEQVARFAARQLRAAAGS
	aggttcccgtcgcgttcgatcagtcgaggtaccaggagggtatcgagcaagtggcgcggtttgccgctcgccagcttcgcgcggctgctggaagtcttacggagga	LTEDEVDGRWRKLYGRYAHRGDEGAEDGEHLGVE
	tgaggtggatggccggtggcggaagttgtacggcaggtatgctcatcgtggggatgagggggctgaggatggtgagcatcttggagttgaggaaattattgagacc	EIIETWPEVRPEARLVGLMEGKVEELFRGRVEAPTL
	tggcccgaagtgcgtcctgaggcacggttggtgggtctcatggaagggaaggtggaggagttgttcaggggtcgcgtcgaggcaccgacacttctcttctcggaag	LFSEEAMEVVEGFYVGSLALDYYNTVLKETLASLLR
	aggcgatggaagtggtggagggcttctacgtgggatcattggcactggactactacaacacggtgctcaaggagacgctcgcctctctcctgcgcgatcgaccaa	DRPNGRKLRILEVGAGTGATTAKLLPVLESVEFEYV
	acggcaggaagctgcgtatcctcgaggtgggtgccggcacgggagccaccaccgccaaattgcttcccgtgctggagtccgtcgagttcgagtacgtcttcacag	FTDVSSVFLKKAKRDIASRLPANGSMVFDILDIEVDP
	acgtcagcagcgtcttcctcaagaaggcgaagcgggacatagcgagccgcttgcccgccaacggatccatggtcttcgacattctggacatcgaggtggatccct	CAQGFQPAQFDIVLAANCIHATRRMKETVANMRSLL
	gcgcgcagggcttccagcccgcccagttcgacatcgtgctcgctgccaattgcatccacgccacgcgccggatgaaagagacggtggccaacatgcgatctctcc	RPSGAMLLFESTRQSAISDCTFGFTDGWWRFEDLE
	tgcgtccttccggtgctatgctgctgttcgagtctacccgccaatctgccatctcggattgcacttttggcttcactgacggctggtggagattcgaggaccttgaactgag	LRPDYPLLSFDTWNSLLMSNGFVSVDRADRLDGAL
	acctgactacccgctcttgtcgtttgacacctggaacagcctcttaatgagtaacggttttgtctcagtcgatcgtgctgatcgtctggatggagcgctcgagtcgtcagc	ESSAIIVARNSSTTEDSPEDTSRMIRGDAAYLVTGG
	cattatcgtagctcgcaacagctcgaccacggaagactccccggaagacacgtcaagaatgatccggggagatgctgcatatctggtcaccggtggcaatagcg	NSGLGLVAARALMDAGARHLILLSRSGAIKEDDAHL
	gcctaggactcgtagccgccagggcgctcatggatgccggtgcacgccacctcatcctgctatcccgatctggagctataaaagaggacgatgctcatctctggcat	WHQLLSAAANMGAKIEHLPCDVGDGTALGQTLASL
	cagctcctctcagcagccgcaaacatgggagccaagatagaacaccttccatgcgatgttggcgatggcaccgctctggggcaaacattagcctcgctgcgtgaa	REQGCPEIRGVIHAAGLLDDAPFRQQSKKTLNTVIS
	cagggctgcccagaaatccgaggtgtgatccacgcggcaggcttgctggacgacgcacccttccgtcagcagtccaagaagacgctcaacaccgtcatttcacc	PKVTGAITLRQALVPQPDFILLFSSAAGILGSPTQSN
	caaggtcaccggagccatcaccctgcgccaagcgctggtgccacaaccagacttcatccttctcttcagctcagccgccggaattctcggaagccccacgcaaag	HCAANATLDALAAQWRHEGLPAMSLAWGMVLGLG
	caaccactgcgctgccaacgccacgctcgatgcactagcggcccagtggcgccatgagggactcccggcaatgagtcttgcctggggaatggtgctcggcttggg	TLRRQPPGRAAIERLGGISPDLAYRTICAAIAATAGC
	aacactacggagacaaccgccgggccgcgcagccattgaaagactgggtggcatctcgcccgatctcgcgtaccgaaccatctgcgccgccattgccgccaca	RHHSGYLLCSPIQWSKLLANSDGTETDAVNSPQSG
	gcgggctgcaggcatcattccggctaccttctctgctctcccatccaatggtccaagcttttggcgaacagtgacggcacagagacagatgccgtcaacagcccgc	HRQLFSTYRKNLALQRQLEPKRKPTRKTARSATSK
	agtccggacaccgtcagctcttctccacgtaccgcaaaaacctcgccctccaacgccaactcgagcccaagcggaagccaaccaggaagaccgcacggtcag	STSAAPKPTQQAAAVIPDLDSITTTIRNILADVTGTNI
	caacgagcaagtcaacgtccgctgcccccaagccaacccagcaggcagcagcagtaatcccggacctcgactccatcaccacgacgatccgcaacatcctcg	PSSEPDPRFEDHGVDSLSSMELGNRLNAAFPGLAI
	ccgacgtgacgggcacgaacatcccgtcatccgaaccagacccgcgcttcgaagaccacggcgtcgactcgctcagctcaatggagctgggcaaccggctca	SPMALNGFPTLSSLSQHVRQELLRNSGGAATTTAP
	acgccgcctttccggggctcgccatctcgcccatggcgctcaacggcttcccgacgctgagttcgctgagccagcacgtccgccaggagttactccgcaacagcg	EEQQQQQQNLIPLNAVPDDGHPDTPIAIVIHGGAG
	gaggagcagccaccaccacagcgccagaagagcagcaacagcaacaacaacaaaacctcatcccccctcaacgccgtccccgacgacggccacccggac	EVHHVKQLAARLPFRVVGVRQTDDVPADSVASMAA
	acccccatcgccatcgtcatccacggcggcgccggcgaggtccaccatgtcaagcagctcgccgcgcgcctgccgttccgcgtcgtcggcgtgcggcagacgg	HYVRVLQRAGGLLGRRADGTVRRRVQLIGYSFGGC
	acgacgtgccggcggattcggtcgcgtcgatggcggcgcactacgtccgcgttctccagcgcgcaggtggcctgctcggtcgccgtgcggacgggacggttcgtc	VAWEMARLLEMQHVQQPERLVLLDGGPTTDVSME
	gccgcgtgcagttgatagggtactcgtttggcggctgtgtcgcgtgggagatggcgcggctgctggagatgcagcacgtgcagcagccggagcggttggtgctgct	QVLGCRLESGSDDAEEEGVGARSIRLMGALLLASG
	ggacggcgggccgacgacggacgtgtcgatggagcaggtgttggggtgtaggttggaatccggtagcgacgatgcagaggaggagggagtgggcgcgcgctc	AKVIGTADIVSTLQRVGLVKKPGSDGKEKQQSSFDL
	gatccgcttgatgggcgcgctgctgctggccagcggcgccaaggtcatcggcaccgcggatatcgtgagcacgctgcaacgcgtcggtctggtcaagaagccgg	DLISRTTREIVELIPPSLRGAPLQRLTDLFAANMRAY
	gctccgatggcaaagagaagcagcagtcgtccttcgacctcgacctcatctcccgcaccacgcgcgagatcgtcgagctcatcccgccgtcgctgcgtggtgcgc	DGYLAFHAAGKAPAYDGPVWVARSKLPRDEEERR
	cgctgcagcggctgacggaccttttcgccgccaatatgcgcgcttacgacggctacctcgccttccacgccgcgggcaaggcaccggcctatgacgggcccgtttg	RAQLWLDSDHYAFMDLPELAEAVAGLVVG*
	ggtcgcgcggtcgaagctgccgcgggacgaggaagagcgacgtcgcgcgcagctgtggctggattcggatcattatgcttttatggacttgcccgagttggcggag	
	gcggtggcggggttggtggttggttga	
lasE	atggcagctactagtgctaaaccttacgagccatcaactgcccctttgagcggttttcagctctactggcgctttggcttcgccggagctgtttgctgctcacttactcatg	MAATSAKPYEPSTAPLSGFQLYWRFGFAGAVCCSL
	gcgccctgactccagttgatgtgtaagtgtcccaaaatacctcgccggatggtcacaaggctgctctgtttggttccctcactccgaacttgcccacatcttcgtattccc	THGALTPVDVVKTSIQLDPVKYNRGLIGGFRQIIGEK
	cgacgctgacgtccattcactgaagtgtgaaaacatcaatccagctcgacccagtcaagtacaaccgcggcttgattggtggattcaggcagattatcggtgagaa	GFGAVWTGVGPTFVGYFFQGALKFGGYEFFKQLSI
	gggcttcggtgccgtgtggactggcgtgggacccaccttcgttgggtacttcttccagggcgctttgaaattcggcggatacgagttcttcaagcagctgtccatcgaca	DTLGYETAAKNRTAVYFASSAAAEFVADIALCPLEA
	cgcttggatacgaaaccgctgctaagaaccgcaccgccgtctacttcgcctcctccgccgctgccgagttcgttgccgatattgcgctctgcccactcgaggccactc	TRIRLVSDPGFADGLIGGFSKLAKAEGLASFYSGFG
	ggatccgcctggtgtctgaccccggctttgccgacggcctgatcggaggcttctcaaagcttgcaaaggccgagggccttgcctccttctactccggctttggtcccatt	PILFKQIPYTCAKFVVYEKVVEAVYKRIDKRTLPDAA
	ttgttcaagcagtacgtagaacgggtccacacctccccactcaccaactcacttcaacgtgtcttccatgcacaggatcccgtacacctgcgccaaattcgtcgtctac	HTAVNLGSGLVAGFAAALVSQPADTMLSKINKVKLL
	gaaaaagtcgtcgaggccgtgtacaagcgcatcgacaagcgcaccctgcccgacgccgcccacacggccgtcaacctcggctccggcctcgtcgccggcttcg	PGEKVSARLVQTAKELGVRGSFTGVGPRLFMVGTL
	ccgcggcgcttgtctcgcagcccgccgatactatgctctccaagatcaacaaggtcaagcttctgccgggcgagaaggtctccgcccgcttggttcagactgccaa	TAFQFAIYGDLKKAIGAVGGIEIAPK*
	ggagctcggcgtacgcggctcctttaccggcgtcgggcctcgtctattcatggtgtaagtgaagaagcaattgcccgcggaagcctgtggcccctcgctgtgactcc	
	agatcccctctgctgatgattaattttctagtggaactcttacggcttttcagtttgccatatacggcgacctgaagaaggctattggtgctgttggtggcatcgagattgcg	
	ccgaagtag	
lasF	atgacttccccttctgcaaccgctctcacggtgctttcgtgtgtctttggctatgtaagctgacgacccttcgtccagccagtagagtctgacagaagacctgtagatattg	MTSPSATALTVLSCVFGYILYKVAHNVFFHPLSRIPG
	tacaaagttgcacacaatgtgttctttcacccactttctcgcatcccaggcccctggactgcagcactgggtgatttctggatggcttctcactggtgagtagttctaagca	PWTAALGDFWMASHWLSGTWHRDIEALHRKYGPV

	cgccaagcaaatgactgaagcttacattactaggctgagcggcacttggcatcgagacatagaagcactccaccgaaaatacggtatgtcaaatcatccataggc<br>catcaaggtggatctaagatctgttttctcttcgaacagggcctgtggtgcgcgttgcgacaaacgaactctctttctgcggagtccagtccatcaaagatatttacgcc<br>acccgccaatacaccgcgcccaattttttaagaagtgtagcacgttctatcttcagtcggacgtgaagtacccgtccattgtaaggaagtcttgctattcggagagcgc<br>caagagagactaattggttaagggaacggaagttgatccggtgaagcattcggagctccgcaagatgatctcccccggcttcagcccaccgactttgagatctcaa<br>ggatatatcatcatgggatacgtgaacaccttggtgaagcagattcaggagcgcagtggtcagcctttggatatgaacaggtggtttctgtggttcacgtttgatgttatc<br>accgatttagtattcggagagtcactgaaaaacgtggagcgaggtgagcttcattaatcgatgctcgaatgacgaatgtctgattgaaagaacaggcgaacccgac<br>cagtggcttacgatgctccaaggaagtgggcccaccctggctgttgcttacatcttacggcgacagcccaaagcagtcatctctgtgctcaagcggctgttggctaac<br>aagaagagcgctgagatgcgagcctaccacgtcagaatgagccaggaaatggcgaagcgacggattgccaaaggcgcaacagaccgggaggacattttca<br>accatctcgttggcgcaggctcacaacagctggacccagaggtccttgccttgcagggtccgacactcatcggtgctggatccgaaacaacttccttgaccctcatg<br>gcaaccacctatcatcttcttcggaatcccgccaagttatccgtgctccaagacgaagtccgcagcgcgttccaagaccgcagcgaaattgacagtgaaagcacc<br>aagactctccgctatctcaacgccgtcattgaggaagccctccgtcttacgtctccagcatcttttggactaccgcgcatatctccaggcgccatggtcgatggcgtatg<br>ggttccaaaagggg taagtagtgggaatcgcgttgcaacgagttgtccggctaattgatgtctcgtgtaccagacaagggtgttcgtagcagaaaacgtcactgccc<br>gtgatccgcggtatttcttggagcctgaaaagtttcaccccgagagatggctacctaaagaccatccattgtatgacagcaggtttgagaatgatgtcaaggatgcat<br>cgaagcctttcttgatggggccacgggcgtgcttggggatgcatctggcatatttggaaatgcggtatgccctctttacagttcgtttttgggtgaatgcttatctttgtgtac<br>aggttggtcatttcaaatctagtctggcaatttgactggacgctgggtgatgaaggcgtggactttgataaagatacgaagctactcggactgtggcgtccagcaccat<br>tgcccgtcacatacaagcccgtcaactaa	VRVATNELSFCGVQSIKDIYATRQYTAPNFFKKCST<br>FYLQSDVKYPSIGTEVDPVKHSELRKMISPGFSPPT<br>LRSQGYIIMGYVNTLVKQIQERSGQPLDMNRWFLW<br>FTFDVITDLVFGESLKNVERGEPDQWLTMLQGSGP<br>TLAVAYILRRQPKAVISVLKRLLANKKSAEMRAYHVR<br>MSQEMAKRRIAKGATDREDIFNHLVGAGSQQLDPE<br>VLALQGPTLIGAGSETTSLTLMATTYHLLRNPAKLSV<br>LQDEVRSAFQDRSEIDSESTKTLRYLNAVIEEALRLT<br>SPASFGLPRISPGAMVDGVWVPKGTRVFVAENVTA<br>RDPRYFLEPEKFHPERWLPKDHPLYDSRFENDVKD<br>ASKPFLMGPRACLGMHLAYLEMRLVISNLVWQFDW<br>TLGDEGVDFDKDTKLLGLWRPAPLPVTYKPVN*
lasG	atgacttccgaaaacgaaaagccggatggctactcttctaccagcaatctcgagaaatcagacgcggaagagcaaaacgatctccccaaggatattgagagag<br>gcgccgatcgagataatcaaaacgagagtggaagccaagaggcccgagatccgaatttggtggagtgggatccgaacgacccggagaatcccttgaactggc<br>cttccttcaagaggactggccatgttgttcttgcaagcgttgctgcgctctttgcgtaagtgtagtagcgtccctatacagagctcacactgacaacttgacggcctacag<br>gaatatcacgtctaccgctttcgctcctgcggctgcacaggtcgttgaagagttcaacattacggactcaaccgtcaccgccttgacagtgagcatatatctcctcgga<br>ttcgcagttgggccactggttattgcacctctctcggaacactatggccgccttcctatctatataattagcatgattacgtccgtcggctttctggtcggctgctcgcaagc<br>cccaagtctgggggtatttctcgcatgtcgattcatcactggcgttgctggatcaggcccaaacacggtgggtggtggtaccattgcagacgtcactcctccagagac<br>aagaggccgcgccatgggcggattcgccgtcgggccgctcttgggcccagtgattggccctatgacggctgggttcatcgcccaatatgtagggtggcgttgggtat<br>ttagggtcctctacattgcggtaagcagcaaaagcactcatttctcacaagtgcacggaaactgagcttccacagactggcgtcgtcacagtattgatcatctctatga<br>tgcgggagaccttcggaccactgctactcgcacgtaaggcagcacggcttcggaaagagacaggtaacccagcgttgtacacgaagtttaggctgagcagcgg<br>gagcgcggctacgtcgctacggacgtccctccaacggcctgtaaagctgctcatcttttcgccgattacgctccttctgtccctctacagtgcctttgtcttcggcttgctga<br>acctgctttttacaacattctccagcgtcttcaaaggacagtaccgtttcggacctggagtttcagggctatcgtatctaggcttagggctgggtatggcggctggagtcg<br>ttatttttagcatcgtgtcggatagagtggcaaagaaagcggcggcgaaaggtaaggcgtggaagccggagtcgaggctgctgcttatggtgtggtttgcgccggtc<br>atcacggtagggtttttctggtacggatggagcgcgtactacaaggtgcactggattgttcccattatcggtacatccttcattggaataggtgcccttatgatcatagtaa<br>gccatcattctcccctttctcgaaccccaccaagctgactacgaatagatgccggtccagatctatcttgtcgatgcgtttggcgcatctattgcggcgtcggcgctggg<br>cgctagcgctatcctccgctctttaactgggtgctttttaccattggctgggccctcactctacgccgctctagggcttggctgggggaacacgttactaggattcctctcttt<br>ggtactttccgcttttcccatattgttctaccgctatggtgaattgataaggactaagtggccggtcgatttctga	MTSENEKPDGYSSTSNLEKSDAEEQNDLPKDIERG<br>ADRDNQNESGSQEARDPNLVEWDPNDPENPLNW<br>PSFKRTGHVVLASVAALFANITSTAFAPAAAQVVEE<br>FNITDSTVTALTVSIYLLGFAVGPLVIAPLSEHYGRLP<br>IYIISMITSVGFLVGCSQAPSLGVFLACRFITGVAGSG<br>PNTVGGGTIADVTPPETRGRAMGGFAVGPLLGPVI<br>GPMTAGFIAQYVGWRWVFRVLYIATGVVTVLIISMM<br>RETFGPLLLARKAARLRKETGNPALYTKFRLSSGSA<br>ATSLRTSLQRPVKLLIFSPITLLLSLYSAFVFGLLNLLF<br>TTFSSVFKGQYRFGPGVSGLSYLGLGLGMAAGVVI<br>FSIVSDRVAKKAAAKGKAWKPESRLLLMVWFAPVIT<br>VGFFWYGWSAYYKVHWIVPIIGTSFIGIGALMIIMPV<br>QIYLVDAFGASIAASALGASAILRSLTGCFLPLAGPSL<br>YAALGLGWGNTLLGFLSLVLSAFPILFYRYGELIRTK<br>WPVDF*

Table S3. NMR data of strasseriolide C (**1**) in CD_3OD.

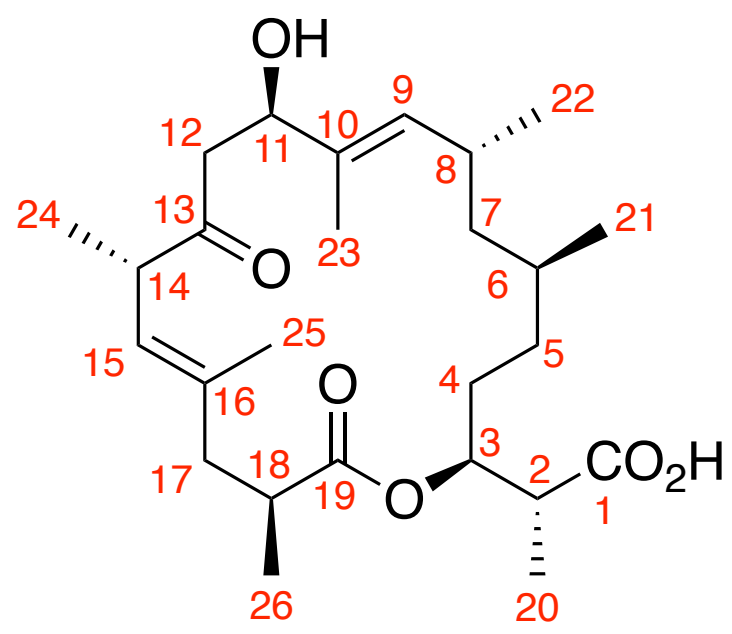

1

position	δC, type	δC (lit.[15a])	δC (lit.[15b])	δH, mult. (J in Hz)	δH, mult. (J in Hz) (lit.[15a])	δH, mult. (J in Hz) (lit.[15b])
1	177.7, C	178.0	177.6			
2	43.4, CH	43.5	43.5	2.73, dq (9.6, 6.8)	2.73, m	2.74, dq (9.8, 6.9)
3	75.1, CH	75.1	75.1	5.11, dt (9.6, 3.5)	5.11, dt (9.7, 3.5)	5.11, dt (9.8, 3.7)
4	30.5, CH_2	30.5	30.5	1.76, tdd (13.7, 4.3, 3.3)	1.76, m	1.75, dddd (14.3, 13.3, 4.6, 3.2)
				1.36, tt (13.8, 3.5)	1.36, m	1.36, m
5	28.9, CH_2	28.9	28.9	1.63, tt (12.9, 3.0)	1.63, m	1.63, tt (13.1, 2.8)
				0.99, m	1.00, m	1.00, m
6	31.3, CH	31.3	31.3	1.19, m	1.19, m	1.18, m
7	48.7, CH_2	48.7	48.7	1.23, m	1.23, m	1.23, m
				1.19, m	1.19, m	1.19, m
8	30.3, CH	30.3	30.3	2.45, m	2.44, m	2.44, m
9	136.2, CH	136.2	136.2	5.04, brd (8.2)	5.04, brd (8.2)	5.04, dq (8.2, 1.3)
10	136.5, C	136.6	136.6			
11	76.2, CH	76.2	76.2	4.46, dd (11.2, 4.3)	4.46, dd (11.1, 4.3)	4.46, dd (11.1, 4.3)
12	46.3, CH_2	46.3	46.4	3.03, dd (14.7, 11.2)	3.03, dd (14.7, 11.2)	3.03, dd (14.7, 11.2)
				2.29, dd (14.7, 4.3)	2.23, m	2.29, dd (14.7, 4.3)
13	210.7, C	210.8	210.8			
14	48.9, CH	48.9	48.9	3.37, dq (10.5, 6.6)	3.37, m	3.37, dq (10.5, 6.6)
15	126.3, CH	126.3	126.3	4.67, brd (10.4)	4.68, brd (10.4)	4.68, d (10.4)
16	138.4, C	138.5	138.5			
17	46.3, CH_2	46.4	46.3	2.32, t (12.8)	2.38, t (12.8)	2.32, t (12.8)
				2.20, dd (13.0, 2.9)	2.20, dd (12.9, 2.8)	2.20, dd (12.8, 3.2)
18	42.2, CH_2	42.2	42.2	2.68, dqd (12.1, 6.9, 3.3)	2.68, m	2.68, dqd (12.1, 6.9, 3.2)
19	175.4, C	175.4	175.4			
20	15.1, CH_3	15.1	15.1	1.12, d (6.9)	1.12, d (6.9)	1.12, d (6.9)
21	21.4, CH_3	21.4	21.4	0.83, d (5.7)	0.83, d (5.6)	0.83, d (5.9)
22	22.2, CH_3	22.2	22.2	0.93, d (6.8)	0.93, d (6.8)	0.93, d (6.8)
23	10.2, CH_3	10.2	10.2	1.57, d (0.9)	1.57, d (0.9)	1.57, d (1.3)
24	15.0, CH_3	15.0	15.0	0.90, d (6.6)	0.90, d (6.6)	0.90, d (6.6)
25	16.0, CH_3	16.0	16.0	1.81, brs	1.81, d (0.8)	1.81, d (1.3)
26	18.5, CH_3	18.5	18.5	1.25, d (6.9)	1.25, d (6.9)	1.25, d (6.9)

^1H NMR: 600 MHz, ^{13}C NMR: 150 MHz (in CD_3OD)

Table S4. NMR data of strasseriolide C (**1**) in $CDCl_3$.

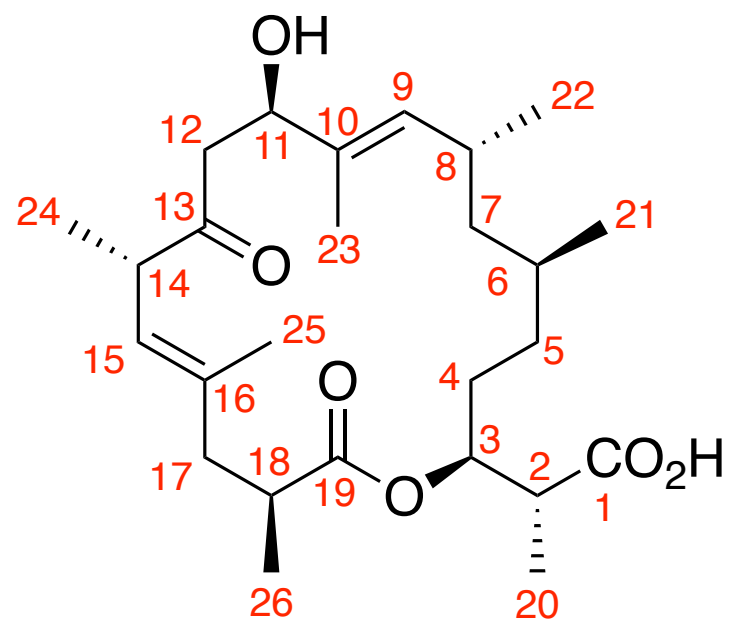

1

position	δ_C, type	δ_H, mult. (J in Hz)
1	178.4, C	
2	41.8, CH	2.74, quin (7.3)
3	73.6, CH	5.11, dt (8.0, 4.0)
4	29.0, CH_2	1.68, m
		1.41, ddt (14.7, 11.0, 4.5)
5	28.7, CH_2	1.54, tt (12.1, 4.0)
		0.94, m
6	29.7, CH	1.25, m
7	46.4, CH_2	1.24, m
		1.19, m
8	29.1, CH	2.44, m
9	135.4, CH	5.15, brd (8.5)
10	134.4, C	
11	75.1, CH	4.57, dd (8.7, 5.8)
12	45.3, CH_2	2.93, dd (15.5, 8.9)
		2,39, dd (15.5, 5.7)
13	209.8, C	
14	47.7, CH	3.22, dq (10.2, 6.6)
15	124.9, CH	4.73, brd (10.2)
16	136.8, C	
17	44.2, CH_2	2.29, dd (13.6, 11.6)
		2.12, dd (13.6, 2.6)
18	40.6, CH_2	2.66, dqd (11.6, 6.9, 3.2)
19	173.9, C	
20	13.4, CH_3	1.16, d (6.9)
21	21.1, CH_3	0.82, d (6.3)
22	21.8, CH_3	0.92, d (6.7)
23	9.8, CH_3	1.59, brs
24	14.5, CH_3	0.96, d (6.6)
25	16.7, CH_3	1.76, brs
26	18.3, CH_3	1.22, d (6.9)

^1H NMR: 600 MHz, ^{13}C NMR: 150 MHz (in $CDCl_3$)

Table S5. NMR data of strasseriolide C (**1**) obtained from the isotope incorporation experiments (see Figure S20 for the ^{13}C NMR spectrum).

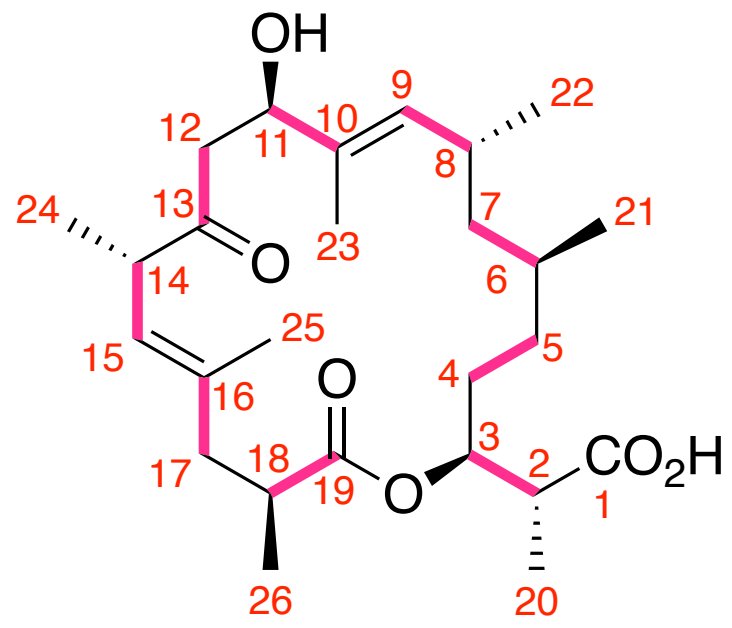

1

position	δ_C	$^1J_{CC}$ from [1, 2-$^{13}C_2$]-acetate (Hz) / Coupled carbon
1	177.7, C	–
2	43.4, CH	19.7 / C-3
3	75.1, CH	19.7 / C-2
4	30.5, CH_2	17.6 / C-5
5	28.9, CH_2	17.6 / C-4
6	31.3, CH	17.6 / C-7
7	48.7, CH_2	17.6 / C-6
8	30.3, CH	21.7 / C-9
9	136.2, CH	21.7 / C-8
10	136.5, C	23.5 / C-11
11	76.2, CH	23.5 / C-10
12	46.3, CH_2	20.2 / C-13
13	210.7, C	20.2 / C-12
14	48.9, CH	overlapped
15	126.3, CH	20.7 / n.d. (deduced to be C-14)
16	138.4, C	21.3 / C-17
17	46.3, CH_2	21.2 / C-16
18	42.2, CH_2	29.2 / C-19
19	175.4, C	29.2 / C-18
20	15.1, CH_3	–
21	21.4, CH_3	–
22	22.2, CH_3	–
23	10.2, CH_3	–
24	15.0, CH_3	–
25	16.0, CH_3	–
26	18.5, CH_3	–

^1H NMR: 600 MHz, ^{13}C NMR: 150 MHz (in CD_3OD)

Table S6. Pairwise sequence identity matrix of ACP domains in LasC and LasD.

	ACPL	ACP1	ACP2a	ACP2b	ACP2c	ACP2d	ACP3a	ACP3b	ACP4	ACP5	ACP6	ACP7	ACP8
**LasC loading module ACP (ACPL)**		23.6	31.5	17.4	21.7	22.9	19.4	27.9	22.5	16.2	32.4	19.1	16.9
**LasC module 1 ACP (ACP1)**	23.6		19.4	23.2	18.8	28.6	28.4	29.4	38.0	26.5	23.9	30.9	25.4
**LasC module 2 ACPa (ACP2a)**	31.5	19.4		23.2	20.3	34.3	26.9	32.4	23.9	33.8	25.4	27.9	23.9
**LasC module 2 ACPb (ACP2b)**	17.4	23.2	23.2		**46.4**	31.9	23.9	27.9	17.4	20.6	17.4	25.0	24.6
**LasC module 2 ACPc (ACP2c)**	21.7	18.8	20.3	**46.4**		31.9	22.4	22.1	17.4	22.1	20.3	23.5	21.7
**LasC module 2 ACPd (ACP2d)**	22.9	28.6	34.3	31.9	31.9		29.9	30.9	35.7	29.4	21.4	35.3	27.1
**LasC module 3 ACPa (ACP3a)**	19.4	28.4	26.9	23.9	22.4	29.9		37.3	34.3	20.9	20.9	29.9	19.4
**LasC module 3 ACPb (ACP3b)**	27.9	29.4	32.4	27.9	22.1	30.9	37.3		35.3	25.0	27.9	35.3	16.2
**LasC module 4 ACP (ACP4)**	22.5	38.0	23.9	17.4	17.4	35.7	34.3	35.3		25.0	26.8	33.8	26.8
**LasC module 5 ACP (ACP5)**	16.2	26.5	33.8	20.6	22.1	29.4	20.9	25.0	25.0		17.6	35.3	19.1
**LasD module 6 ACP (ACP6)**	32.4	23.9	25.4	17.4	20.3	21.4	20.9	27.9	26.8	17.6		16.2	19.7
**LasD module 7 ACP (ACP7)**	19.1	30.9	27.9	25.0	23.5	35.3	29.9	35.3	33.8	35.3	16.2		25.0
**LasD module 8 ACP (ACP8)**	16.9	25.4	23.9	24.6	21.7	27.1	19.4	16.2	26.8	19.1	19.7	25.0	

Table S7. Primers used in this study.

Primer	Sequence (5′ to 3′)
sC5'-lasC-F	GGCGGGCTCTGTCTTCTAAAGGTTG
PenoA142R	GGGTACCGAGCTCGAATTCGTTTG
PenoA-lasC1-F	CAAACGAATTCGAGCTCGGTACCCAAGATGGACTCAATCGAGGAAATCC
lasC1-R1	CGGATCCAGGTCTCGTTAACAGAAC
lasC1-F1	GTTAACGAGACCTGGATCCGCAAATTGG
lasC1-sC_up-R	CTTTAGAAGACAGAGCCCGCCAGGGAGCTCTGTTCGACATGCAC
sC-3'-F	GGTCTTGAATGGTGGGATATTTTGTCC
sC_down-lasC2-R	GGCGTTACCCAACTTAATCGCCTTGC
lasC2-sC_down-F	CGATTAAGTTGGGTAACGCCAGCATGTTACACACAACCCTC
lasC2-R1	GTGGCTTGTTGCTTGGTGAGG
lasC2-F1	CTCACCAAGCAACAAGCCACAG
lasC2-TamyB-R	CTTCACGAGCTACTACAGATCCCCGTCTTCAACCCATCCTCTCACTG
TamyB-F	GATCTGTAGTAGCTCGTGAAGGGTG
adeA-sC_down-R	CAAAATATCCCACCATTCAAGACCGTCATGTCCAGGAAGATAGGTCAG
lasD-F	TCGAGCTCGGTACCCATGGCCGAATCTGAAGATGTCGG
lasD-R1	GGCCGCAAATCCTTATCATCGAAC
lasD-F1	GATGATAAGGATTTGCGGCCTGAG
lasD-R	CTACTACAGATCCCCTCAACCAACCACCAACCCCG
lasB-F	TCGAGCTCGGTACCCATCACGATGGCCTCGACAGAC
lasB-R	CTACTACAGATCCCCTACACCGCTAGGACACGTGATG
lasF-F	TCGAGCTCGGTACCCATGACTTCCCCTTCTGCAACCG
lasF-R	CTACTACAGATCCCCTTAGTTGACGGGCTTGTATGTGACG
lasE-F	TCGAGCTCGGTACCCAGATGGCAGCTACTAGTGC
lasE-R	CTACTACAGATCCCCACTACTTCGGCGCAATCTCGATG
lasG-F	TCGAGCTCGGTACCCACAAAGGTAGTCTTCATGACTTCCG
lasG-R	CTACTACAGATCCCCTCCACCATCCTCAGAAATCGAC
InF-Linker1-F	GCTCGCGAGCGCGTTCCCCATCATGGTGTTTTGATC
InF-Linker1-R	AACGCGCTCGCGAGCGTAAGATACATGAGCTTCGG
InF_pPTRI_SmaI_F	CTCTAGAGGATCCCCATCATGGTGTTTTGATC
InF_pPTRI_SmaI_R	TCGAGCTCGGTACCCGTAAGATACATGAGCTTCGG
InF-pPTRI-SdaI-F2	TTACGCCAAGCTTGCATGCCCCCCCATCATGGTGTTTTGATCG
InF-pPTRI-SdaI-R2	GGGATCCTCTAGAGTCGACCGTAAGATACATGAGCTTCGGAGGGATTACTCAAC
InF-pPTRI-SdaI-R	CCTCTAGAGTCGACCGTAAGATACATGAGCTTCGGTG
InF-linker-F1	GCTCGCGAGCGCGTTCCACTGCATCATCAGTCTAG
HS601-pyrG-F	ATGTTTCAAGTTGCAATGACTATCATCTGTTAG
HS601-SdaI-R	CTCACTACCCGAATGGGTTCAGTG
HS601-SdaI-pyrG-F	GAACCCATTCGGGTAGTGAGCTAACGCCAAGCACGCTAGT
pyrG-HS601-R	GTCATTGCAACTTGAAACATCTGCAGGTCGGATTCAAGGC
PgpdA1-F	TCGAGCTCGGTACCCCCATCATGGTGTTTTGATCAATCGGTCCTTGGGAGATGATC
PgpdA1-R	AGATCCCCGGGTACCGAGCTCGATGTGTTCTTTGGTAGTTTTGGCTTGC
TgpdA1-F	CGGTACCCGGGGATCTGTAGTAGAGGGTCATGATTGATGATATGGAC
TgpdA1-R	CTCTAGAGGATCCCCGTAAGATACATGAGCTTCGGCAGAATTAGGAACAGACTTGCGG
PenoA142h-F	TCGAGCTCGGTACCCCCATCATGGTGTTTTGATCGTGAAAGGCGCCAACG
PenoA142h-R	AGATCCCCGGGTACCGAGCTCGAATTCG
TpgkA-F	CGGTACCCGGGGATCTGTAGTAGCGAAACGCAAATAATGTGTTGAAAG

TpgkA-R	CTCTAGAGGATCCCCGTAAGATACATGAGCTTCGGACGACTTGAGTTTTTCTCGCCAC
Pubi-F	TCGAGCTCGGTACCCCCATCATGGTGTTTTGATCGTCGCCAGAGTCAGGACACAG
Pubi-R	AGATCCCCGGGTACCGAGCTCGATGACGACGGTGAAAGTATTTCAG
Tubi-F	CGGTACCCGGGGATCTGTAGTAGCCGTGGTGGAAACTAATGTCTTG
Tubi-R	CTCTAGAGGATCCCCGTAAGATACATGAGCTTCGGAGGGATTACTCAACTTTAACTTGTCGC
HS601-F2	GCGCTACAGATTTATCCGGCGATC
HS601-R2	GCTCGATTACACTATCATCCACAAGACCC
pPTRI_HS601-F1	ACCTGGTATCCCACCAAAGGCACGACAGGTTTCCCGACTGGAAAG
pPTRI_HS601-R1	GCCGGATAAATCTGTAGCGCCCCTTCGATAAACCTTTGGTGATTGG
pPTRI_HS601-F2	GGATGATAGTGTAATCGAGCGGGAGGAAGACTTGTTCAGTTTCGTC
pPTRI_HS601-R2	TCATCCTTGAGCTTTCTGCAGCCAGCTGCATTAATGAATCGGCC
HS601-F1	TGCAGAAAGCTCAAGGATGACGTAC
HS601-R1	CCTTTGGTGGGATACCAGGTGGAC
sC-5'-argB-F	GAATGTAACTGTACTTCTTGTTGCATGCCTGCAGGTTCGAC
sC-5'-R	CAAGAAGTACAGTTACATTCGAAGGTGAC
sC-5'-F	CGGGTTGTTTGAGCTAGCAATGGC
sC-5'-up-R	TTGCTAGCTCAAACAACCCGGCCGTCGTTTTACAACGTCGT
lasC-H3660A-F	GTAACGTTTTGGCTGCCACGAAAGACCTTACTCG
lasC-H3660A-R	CGTGGCAGCCAAAACGTTACAGGCCAGC
lasC-H6171-F	CGTGCTAGCTGCCACAGCCGATCTGTCAG
lasC-H6171-R	GGCTGTGGCAGCTAGCACGTTGGCCGCCAG
lasC-H8444A-F	CAACGTCCTCGCTGCTACGGCTGACCTTGAC
lasC-H8444A-R	CCGTAGCAGCGAGGACGTTGGTGGCGAG
lasD-H765A-F	GTCAACGTGCTGGCCGCCACGCCGAGCGTG
lasD-H765A-R	CGTGGCGGCCAGCACGTTGACGCAG
lasD-H2898A-F	CAACGTGATAGCCGCGACGCGCGTGCTG
lasD-H2898A-R	GCGTCGCGGCTATCACGTTGACGGCG
lasD-H4989A-F	CAATTGCATCGCCGCCACGCGCCGGATG
lasD-H4989A-R	GCGTGGCGGCGATGCAATTGGCAGC
adeA-lasC-F2	GGATGAATGTTCTCATCATGCTCGC
adeA-LasC-R2	CATGATGAGAACATTCATCCGGATCAG
lasC-F3	CTCAAGGCCATTGAGATCGGTGCC
lasC-R3	CCGATCTCAATGGCCTTGAGAC
lasC-F4	GGATGGAACGCTCATCAGGAC
lasC-R4	CCTGATGAGCGTTCCATCCAG
lasC-F1	GAATAATGAACACTCCGGACGATGACAC
lasC-R1	GTCCGGAGTGTTCATTATTCCGCTTC
lasD-E791Q-F	GTGCTGCAGCAGATCACGACGCAGG
lasD-E791Q-R	GTGATCTGCTGCAGCACGAGCGC
lasC-S4132A-F	GGTCGACGCGCTATTGTCGATTGAGC
lasC-S4132A-R	GACAATAGCGCGTCGACCCCGGCGTCCTTC
lasC-S4263A-F	CTCGACGCCCTCTCGTCGGTGG
lasC-S4263A-R	GACGAGAGGGCGTCGAGGCCGCAGTCG
lasC-S4357A-F	CTTGGATGCTCTTTCCTCCGTGGAGCTC
lasC-S4357A-R	CGGAGGAAAGAGCATCCAAGCCTGCTTC
lasC-S4497A-F	GTGTCGACGCACTCTCATCCCTCGAG
lasC-S4497A-R	GGATGAGAGTGCGTCGACACCACTGTCG

lasC-S40S-F	GTTGACGCCCTTCGCGCTCTCGAG
lasC-S40S-R	CGCGAAGGGCGTCAACGCCGCGGTATAAG
pET28a-ACPL-F	AGGAGATATACCATGGACTCAATCGAGGAAATCCAACAAAAG
pET28a-ACPL-R	GGTGGTGGTGCTCGATAGTGGACGCCGATGCCGTT
pET28a-ACP2a-F	AGGAGATATACCATGACCACTGATTCCGGTGCCAA
pET28a-ACP2a-R	GGTGGTGGTGCTCGATCGAAGAAGAACTCTTTTTCTCCACAG
pET28a-ACP2d-F	AGGAGATATACCATGCATCTCAACAACGTGGATAAATGGGGT
pET28a-ACP2d-R	GGTGGTGGTGCTCGATTATGGGTCTGCTGGCCCTTTC
pET28a-MT2-F	AGGAGATATACCATGACACGCGCCAGCGCTGACATCCTCTC
pET28a-MT2-R	GTGGTGGTGCTCGACAGATGCTGAGGGAGTGCGG
pET28a-MT3-F	CGCGCGGCAGCCATATGCTTTCCAACGCTACCGGAAG
pET28a-MT3-R	GACGGAGCTCGAATTTCAAGGATGAAGCGGCCTGC
pET28a-MT4-F	CGCGCGGCAGCCATATGAGCGGCTTTGAGGACGGACGATC
pET28a-MT4-R	GACGGAGCTCGAATTTCAATGGACTGCGGCGTCTC
pET28a-MT6-F	CGCGCGGCAGCCATATGGGCGACGAGGAGGCGCTTCTCAATG
pET28a-MT6-R	GACGGAGCTCGAATTTCACGCCACGCCCTTCTG
pET28a-MT7-F	CGCGCGGCAGCCATATGGCGTTCGATTTCTCCATCGAGAC
pET28a-MT7-R	GACGGAGCTCGAATTTCAATCCTCCTGGGCGGAAGCCTGAG
pET28a-MT8-F	CGCGCGGCAGCCATATGATTGCGGCCGCGAACACTTC
pET28a-MT8-R	GACGGAGCTCGAATTTCATTCCGTGGTCGAGCTGTTG

Table S8. Plasmids constructed in this study and PCR conditions for the amplification of the inserts for the plasmid constructions.

Plasmid	Inserts	Primer 1	Primer 2	PCR template	Vector
pPgpdA1	*PgpdA1*<br>*TpgdA1*	PgpdA1-F<br>TpgdA1-F	PgpdA1-R<br>TpgdA1-R	gDNA of *A. oryzae* NSARU1	pUC19 digested with *Sma*I
pPubi	*Pubi*<br>*Tubi*	Pubi-F<br>Tubi-F	Pubi-R<br>Tubi-R	gDNA of *A. oryzae* NSARU1	pUC19 digested with *Sma*I
pPenoA142h	*PenoA142h*<br>*TpgkA*	PenoA142h-F<br>TpgkA-F	PenoA142h-R<br>TpgkA-R	pTAex3A-HR<br>gDNA of *A. oryzae* NSARU1	pUC19 digested with *Sma*I
pPTRI-HR	HS601 upstream region<br>part of pPTRI (1)<br>HS601 downstream region<br>part of pPTRI (2)	HS601-F1<br>pPTRI_HS601-F1<br>HS601-F2<br>pPTRI_HS601-F2	HS601-R1<br>pPTRI_HS601-R1<br>HS601-R2<br>pPTRI_HS601-R2	gDNA of *A. oryzae* NSAR1<br>pPTRI<br>gDNA of *A. oryzae* NSAR1<br>pPTRI	N/A
pPyrG-HS601	part of pPTRI-HR<br>part of pPyrG	HS601-pyrG-F<br>HS601-SdaI-pyrG-F	HS601-SdaI-R<br>pyrG-HS601-R	pPTRI-HR<br>pPyrG	N/A
pLasC1	part of pTAex3A-HR<br>1st fragment of *lasC*<br>2nd fragment of *LasC*	sC5'-lasC-F<br>PenoA-lasC1-F<br>lasC1-F1	PenoA142R<br>lasC1-R1<br>lasC1-sC_up-R	pTAex3A-HR<br>gDNA of *L. theobromae*<br>gDNA of *L. theobromae*	N/A
pLasC2	part of pTAex3A-HR<br>3rd fragment of *lasC*<br>4th fragment of*lasC*<br>part of pAdeA	sC-3'-F<br>lasC2-sC_down-F<br>lasC2-F1<br>TamyB-F	sC_down-LasC2-R<br>lasC2-R1<br>lasC2-TamyB-R<br>adeA-sC_down-R	pTAex3A-HR<br>gDNA of *L. theobromae*<br>gDNA of *L. theobromae*<br>pAdeA-HR	N/A
pPyrG-HR-LasD	1st fragment of *LasD*<br>2nd fragment of *LasD*	lasD-F<br>lasD-F1	lasD-R1<br>lasD-R	gDNA of *L. theobromae*	pPyrG-HR digested with *Sma*I
pPgpdA1-lasB	*lasB*	lasB-F	lasB-R	gDNA of *L. theobromae*	pPgpdA1 digested with *Sma*I
pPenoA142h-lasF	*lasF*	lasF-F	lasF-R	gDNA of *L. theobromae*	pPenoA142h digested with *Sma*I
pPubi-lasE	*lasE*	lasE-F	lasE-R	gDNA of *L. theobromae*	pPubi digested with *Sma*I
pTAex3-lasG	*lasG*	lasF-F	lasG-R	gDNA of *L. theobromae*	pTAex3 digested with *Sma*I
pPyrG-HS601-lasB	*PgpdA1-lasB-TgpdA1*	InF_pPTRI_SmaI_F	InF_pPTRI_SmaI_R	pPgpdA1-lasB	pPyrG-HS601 digested with *SmaI*
pPyrG-HS601-lasF	*pPenoA142h-lasF-TpgkA*	InF_pPTRI_SmaI_F	InF_pPTRI_SmaI_R	pPenoA142h-lasF	pPyrG-HS601 digested with *SmaI*
pPyrG-HS601-lasB+F	*PgpdA1-lasB-TgpdA1*<br>*pPenoA142h-lasF-TpgkA*	InF_pPTRI_SmaI_F<br>Inf-Linker1-F	InF-Linker1-R<br>InF_pPTRI_SmaI_R	pPgpdA1-lasB<br>pPenoA142h-lasF	pPyrG-HS601 digested with *SmaI*
pPyrG-HS601-lasB+F+E	*Pubi-lasE-Tubi*	InF-pPTRI-SdaI-F2	InF-pPTRI-SdaI-R2	pPubi-lasE	pPyrG-HS601-lasB+F digested with *Sda*I
pPyrG-HS601-lasB+F+E+G	*Pubi-lasE-Tubi*<br>*PamyB-lasG-TamyB*	InF-pPTRI-SdaI-F2<br>InF-linker-F1	InF-Linker1-R<br>InF-pPTRI-SdaI-R	pPubi-lasE<br>pTAex3-lasG	pPyrG-HS601-lasB+F digested with *Sda*I

pLasC1-H3660A	1st part of pLasC1-H3660A<br>2nd part of pLasC1-H3660A<br>3rd part of pLasC1-H3660A	sC-5'-F<br>lasC-F1<br>lasC-H3660A-F	lasC-R1<br>lasC-H3660A-R<br>sC-5'-up-R	pLasC1	N/A
pLasC2-H6171A	1st part of pLasC1-H6171A<br>2nd part of pLasC1-H6171A<br>3rd part of pLasC1-H6171A	adeA-lasC-F2<br>lasC-H6171-F<br>lasC-F4	lasC-H6171-R<br>lasC-R4<br>adeA-lasC-R2	pLasC1	N/A
pLasC2-H8444A	1st part of pLasC1-H8444A<br>2nd part of pLasC1-H8444A<br>3rd part of pLasC1-H8444A	adeA-LasC-F2<br>lasC-F3<br>lasC-H8444A-F	lasC-R3<br>lasC-H8444A-R<br>adeA-lasC-R2	pLasC2	N/A
pPyrG-HR-lasD-H765A	1st part of lasD-H765A<br>2nd part of lasD-H765A<br>3rd part of lasD-H765A	lasD-F<br>lASD-H765A-F<br>lasD-F1	lasD-H765A-R<br>lasD-R1<br>lasD-R	pPyrG-HR-lasD	pPyrG-HR digested with *Sma*I
pPyrG-HR-lasD-H2898A	1st part of lasD-H2898A<br>2nd part of lasD-H2898A	lasD-F<br>lasD-H2898A-F	lasD-H2898A-R<br>lasD-R	pPyrG-HR-lasD	pPyrG-HR digested with *Sma*I
pPyrG-HR-lasD-H4989A	1st part of lasD-H4989A<br>2nd part of lasD-H4989A<br>3rd part of lasD-H4989A	lasD-F<br>lasD-F1<br>lasD-H4989A-F	lasD-R1<br>lasD-H4989A-R<br>lasD-R	pPyrG-HR-lasD	pPyrG-HR digested with *Sma*I
pPyrG-HR-lasD-H765A/E791Q	1st part of lasD-H765A/E791AQ<br>2nd part of lasD-H765A/E791AQ<br>3rd part of lasD-H765A/E791AQ	lasD-F<br>lasD-E791Q-F<br>lasD-F1	lasD-E791Q-R<br>lasD-R1<br>lasD-R	pPyrG-HR-lasD-H765A<br>pPyrG-HR-lasD<br>pPyrG-HR-lasD	pPyrG-HR digested with *Sma*I
pLasC1-S4132A	1st part of pLasC1-S4132A<br>2nd part of pLasC1-S4132A<br>3rd part of pLasC1-S4132A	lasC-S4132A-F<br>sC-5'-argB-F<br>lasC-F1	sC-5'-R<br>lasC-R1<br>lasC-S4132A-R	pLasC1	N/A
pLasC1-S4263A	1st part of pLasC1-S4263A<br>2nd part of pLasC1-S4263A<br>3rd part of pLasC1-S4263A	lasC-S4263A-F<br>sC-5'-argB-F<br>lasC-F1	sC-5'-R<br>lasC-R1<br>lasC-S4263A-R	pLasC1	N/A
pLasC1-S4263A	1st part of pLasC1-S4357A<br>2nd part of pLasC1-S4357A<br>3rd part of pLasC1-S4357A	lasC-S4357A-F<br>sC-5'-argB-F<br>lasC-F1	sC-5'-R<br>lasC-R1<br>lasC-S4357A-R	pLasC1	N/A
pLasC1-S4497A	1st part of pLasC1-S4497A<br>2nd part of pLasC1-S4497A<br>3rd part of pLasC1-S4497A	lasC-S4497A-F<br>sC-5'-argB-F<br>lasC-F1	sC-5'-R<br>lasC-R1<br>lasC-S4497A-R	pLasC1	N/A
pLasC1-S4132A/S4263A	1st part of pLasC1-S4132A/S4263A<br>2nd part of pLasC1-S4132A/S4263A<br>3rd part of pLasC1-S4132A/S4263A	sC-5'-argB-F<br>lasC-F1<br>lasC-S4263A-F	lasC-R1<br>lasC-S4263A-R<br>sC-5'-R	pLasC1<br>pLasC1-S4132A<br>pLasC1	N/A
pLasC1-S4132A/S4357A	1st part of pLasC1-S4132A/S4357A<br>2nd part of pLasC1-S4132A/S4357A<br>3rd part of pLasC1-S4132A/S4357A	sC-5'-argB-F<br>lasC-F1<br>lasC-S4357A-F	lasC-R1<br>lasC-S4357A-R<br>sC-5'-R	pLasC1<br>pLasC1-S4132A<br>pLasC1	N/A
pLasC1-S4132A/S4497A	1st part of pLasC1-S4132A/S4497A<br>2nd part of pLasC1-S4132A/S4497A<br>3rd part of pLasC1-S4132A/S4497A	sC-5'-argB-F<br>lasC-F1<br>lasC-S4497A-F	lasC-R1<br>lasC-S4497A-R<br>sC-5'-R	pLasC1<br>pLasC1-S4132A<br>pLasC1	N/A
pLasC1-S4263A/S4357A	1st part of pLasC1-S4263A/S4357A<br>2nd part of pLasC1-S4263A/S4357A<br>3rd part of pLasC1-S4263A/S4357A	sC-5'-argB-F<br>lasC-F1<br>lasC-S4357A-F	lasC-R1<br>lasC-S4357A-R<br>sC-5'-R	pLasC1<br>pLasC1-S4263A<br>pLasC1	N/A
pLasC1-S4263A/S4497A	1st part of pLasC1-S4263A/S4497A<br>2nd part of pLasC1-S4263A/S4497A<br>3rd part of pLasC1-S4263A/S4497A	sC-5'-argB-F<br>lasC-F1<br>lasC-S4497A-F	lasC-R1<br>lasC-S4497A-R<br>sC-5'-R	pLasC1<br>pLasC1-S4263A<br>pLasC1	N/A
pLasC1-S4357A/S4497A	1st part of pLasC1-S4357A/S4497A<br>2nd part of pLasC1-S4357A/S4497A<br>3rd part of pLasC1-S4357A/S4497A	sC-5'-argB-F<br>lasC-F1<br>lasC-S4497A-F	lasC-R1<br>lasC-S4497A-R<br>sC-5'-R	pLasC1<br>pLasC1-S4357A<br>pLasC1	N/A
pLasC1-S40A	1st part of pLasC1-S40A<br>2nd part of pLasC1-S40A<br>3rd part of pLasC1-S40A	sC5'-lasC-F<br>lasC-S40S-F<br>lasC1-F1	lasC-S40S-R<br>lasC1-R1<br>lasC1-sC_up-R	pLasC1	N/A
pET28a-ACPL	*ACPL*	pET28a-ACPL-F	pET28a-ACPL-R	pLasC1	pET28a digested with *Nco*I and *Xho*I
pET28a-ACP2a	*ACP2a*	pET28a-ACP2a-F	pET28a-ACP2a-R	pLasC1	pET28a digested with *Nco*I and *Xho*I
pET28a-ACP2d	*ACP2d*	pET28a-ACP2d-F	pET28a-ACP2d-R	pLasC1	pET28a digested with *Nco*I and *Xho*I
pET28a-MT2	*MT2*	pET28a-MT2-F	pET28a-MT2-R	pLasC1	pET28a digested with *Nco*I and *Xho*I
pET28a-MT3	*MT3*	pET28a-MT3-F	pET28a-MT3-R	pLasC1	pET28a digested with *Nde*I and *Eco*RI
pET28a-MT4	*MT4*	pET28a-MT4-F	pET28a-MT4-R	pLasC1	pET28a digested with *Nde*I and *Eco*RI
pET28a-MT6	*MT6*	pET28a-MT6-F	pET28a-MT6-R	pLasC2	pET28a digested with *Nde*I and *Eco*RI
pET28a-MT7	*MT7*	pET28a-MT7-F	pET28a-MT7-R	pLasC2	pET28a digested with *Nde*I and *Eco*RI
pET28a-MT8	*MT8*	pET28a-MT8-F	pET28a-MT8-R	pLasC2	pET28a digested with *Nde*I and *Eco*RI

Table S9. *Aspergillus oryzae* transformants constructed in this study.

**Strain**	**Host strain**	**Plasmids used for transformation**
A. oryzae/lasC	*A. oryzae* NSARU1	pLasC1, pLasC2
A. oryzae/lasD	*A. oryzae* NSARU1	pPyrG-HR-lasD
A. oryzae/lasCD	*A. oryzae/lasC*	pPyrG-HR-lasD
A. oryzae/lasCD (pyrG⁻)	*A. oryzae/lasCD*	N/A
A. oryzae/lasCDB	*A. oryzae/lasCD (pyrG⁻)*	pPyrG-HS601-lasB
A. oryzae/lasCDF	*A. oryzae/lasCD (pyrG⁻)*	pPyrG-HS601-lasF
A. oryzae/lasCDBF	*A. oryzae/lasCD (pyrG⁻)*	pPyrG-HS601-lasB+F
A. oryzae/lasCDBFE	*A. oryzae/lasCD (pyrG⁻)*	pPyrG-HS601-lasB+F+E
A. oryzae/lasCDBFEG	*A. oryzae/lasCD (pyrG⁻)*	pPyrG-HS601-lasB+F+E+G
A. oryzae/lasC (H3660A)+*D*	*A. oryzae/lasD*	pLasC1-H3660A
A. oryzae/lasC (H6171A)+*D*	*A. oryzae/lasD*	pLasC2-H6171A
A. oryzae/lasC (H8444A)+*D*	*A. oryzae/lasD*	pLasC2-H8444A
A. oryzae/lasC+D (H765A)	*A. oryzae/lasC*	pPyrG-HR-lasD-H765A
A. oryzae/lasC+D (H765A/E791Q)	*A. oryzae/lasC*	pPyrG-HR-lasD-H765A/E791Q
A. oryzae/lasC+D (H2898A)	*A. oryzae/lasC*	pPyrG-HR-lasD-H2898A
A. oryzae/lasC+D (H4989A)	*A. oryzae/lasC*	pPyrG-HR-lasD-H4989A
A. oryzae/lasC (S4132A)+*D*	*A. oryzae/lasD*	pLasC1-S4132A, pLasC2
A. oryzae/lasC (S4263A)+*D*	*A. oryzae/lasD*	pLasC1-S4263A, pLasC2
A. oryzae/lasC (S4357A)+*D*	*A. oryzae/lasD*	pLasC1-S4263A, pLasC2
A. oryzae/lasC (S4497A)+*D*	*A. oryzae/lasD*	pLasC1-S4497A, pLasC2
A. oryzae/lasC (S4132A/S4263A)+*D*	*A. oryzae/lasD*	pLasC1-S4132A/S4263A, pLasC2
A. oryzae/lasC (S4132A/S4357A)+*D*	*A. oryzae/lasD*	pLasC1-S4132A/S4357A, pLasC2
A. oryzae/lasC (S4132A/S4497A)+*D*	*A. oryzae/lasD*	pLasC1-S4132A/S4497A, pLasC2
A. oryzae/lasC (S4263A/S4357A)+*D*	*A. oryzae/lasD*	pLasC1-S4263A/S4357A, pLasC2
A. oryzae/lasC (S4263A/S4497A)+*D*	*A. oryzae/lasD*	pLasC1-S4263A/S4497A, pLasC2
A. oryzae/lasC (S4357A/S4497A)+*D*	*A. oryzae/lasD*	pLasC1-S4357A/S4497A, pLasC2
A. oryzae/lasC (S40A)+*D*	*A. oryzae/lasD*	pLasC1-S40A, pLasC2

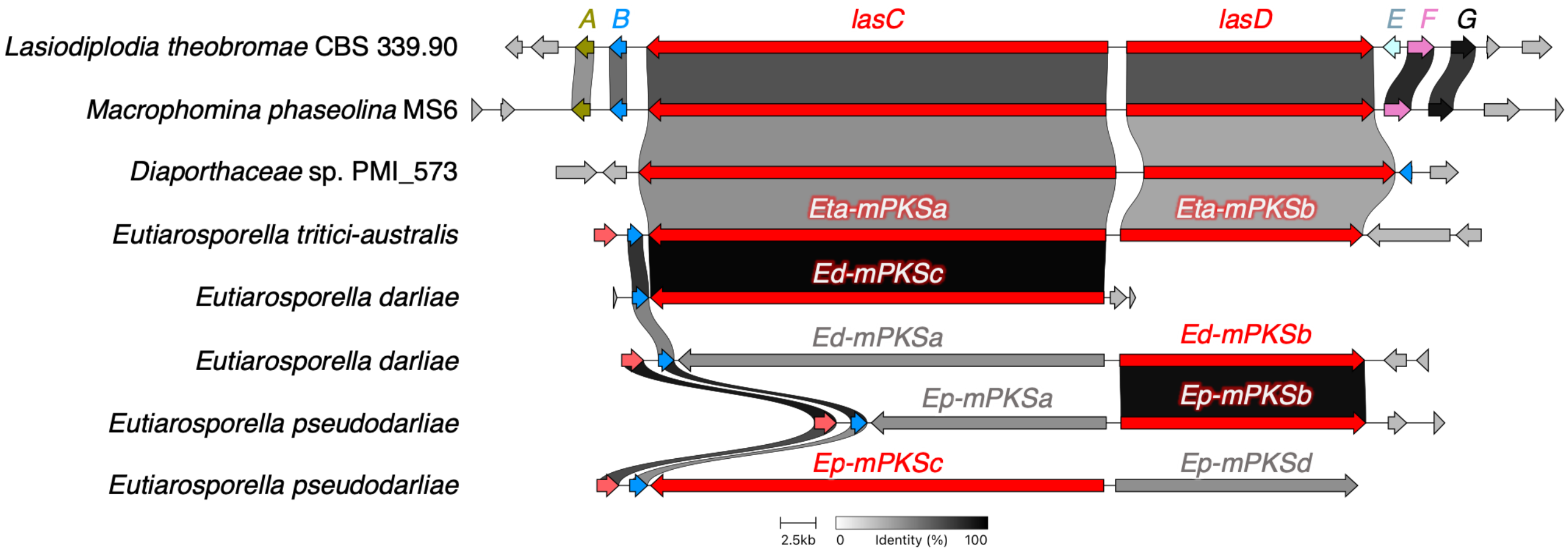

Figure S1. Comparison of fungal modular PKS gene clusters identified by BLASTp analysis or reported previously,[16] visualized using clinker.[17] The *Ed-mPKSa*, *Ep-mPKSa*, and *Ep-mPKSd* genes are predicted to be nonfunctional due to sequence deletions or internal stop codons and were therefore excluded from the clinker analysis.

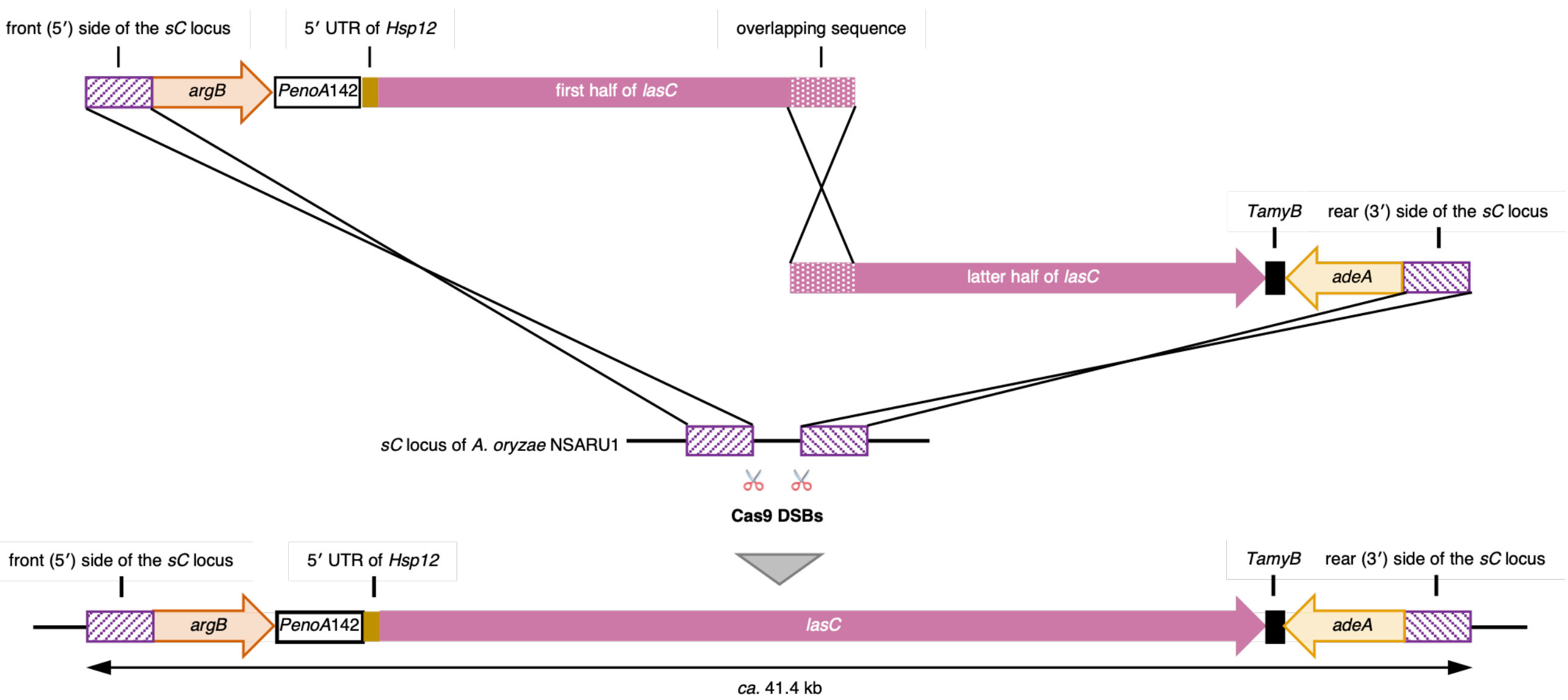

Figure S2. Introduction of *lasC* in a single transformation event. The *argB* and *adeA* genes are used as selection markers. The 5′-untranslated region (5′-UTR) of *Hsp12*[4] was added to increase the gene expression level. *PenoA*142, improved *enoA* promoter; *TamyB*, *amyB* terminator; DSB, double-strand break.

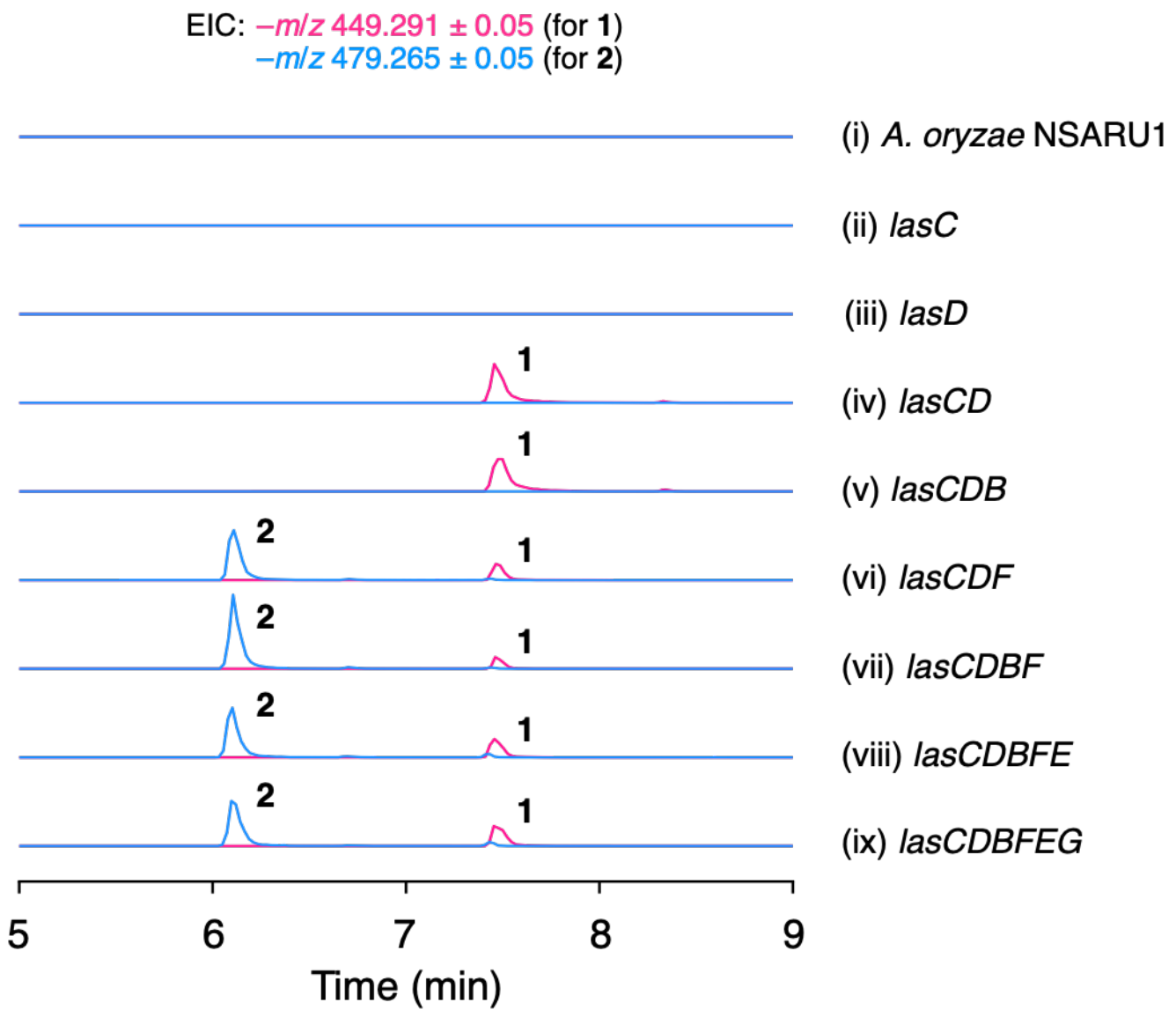

Figure S3. LC–MS profiles of metabolites produced by *A. oryzae* transformants expressing the *las* genes.

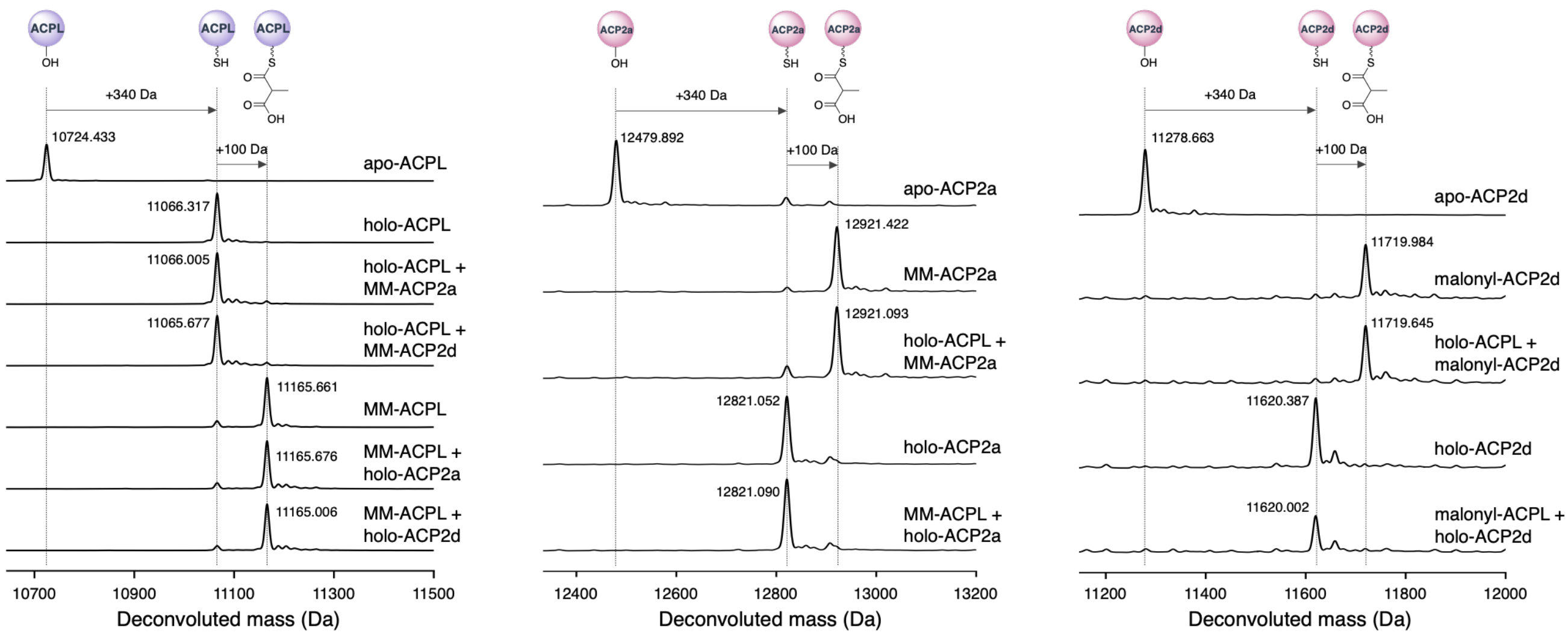

Figure S4. Deconvoluted mass spectra of ACP species following incubation of holo loading ACP (ACPL) with methylmalonyl (MM)-ACP2a or MM-ACP2d, or MM-ACPL with holo-ACP2a or holo-ACP2d.

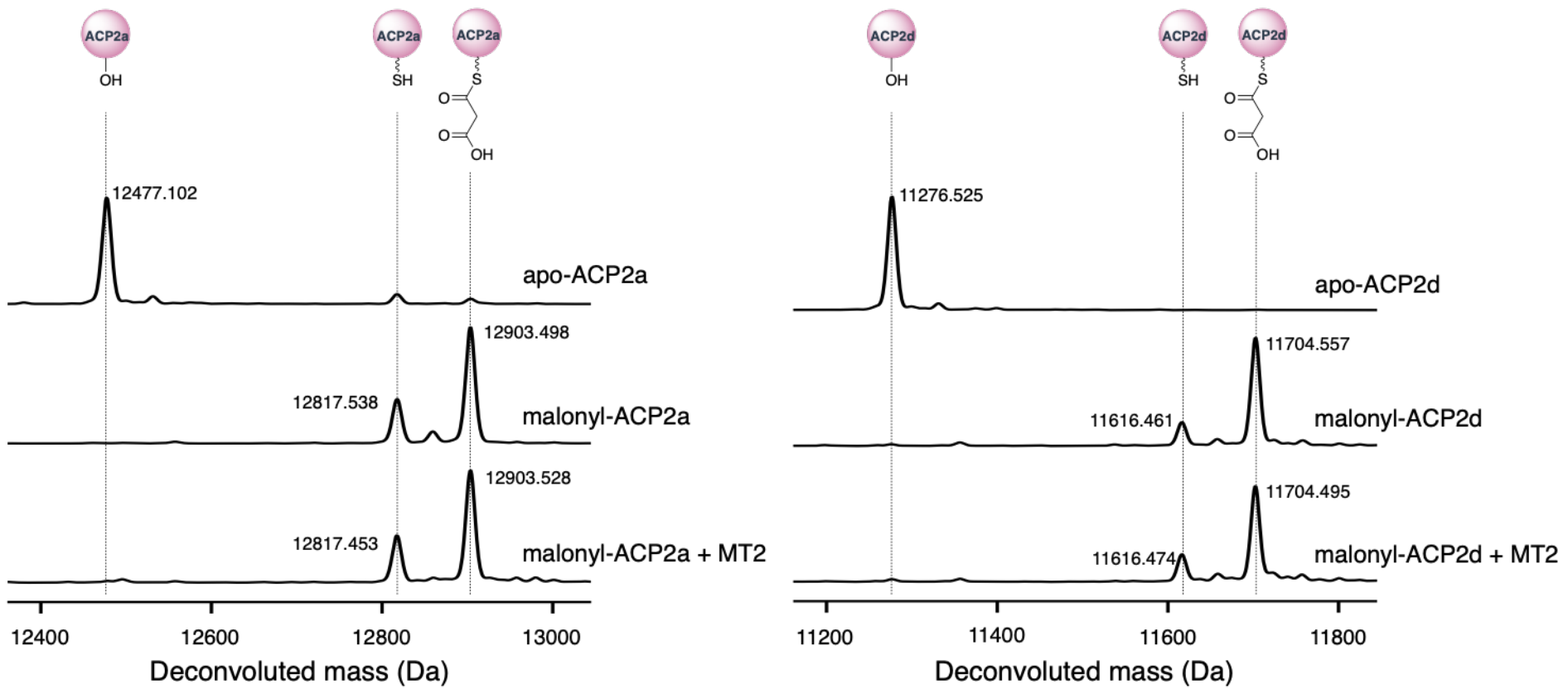

Figure S5. Deconvoluted mass spectra of malonyl-ACP species after incubation with MT2. Malonyl-ACP2a and malonyl-ACP2d underwent spontaneous hydrolysis during incubation, resulting in detectable holo-ACP species.

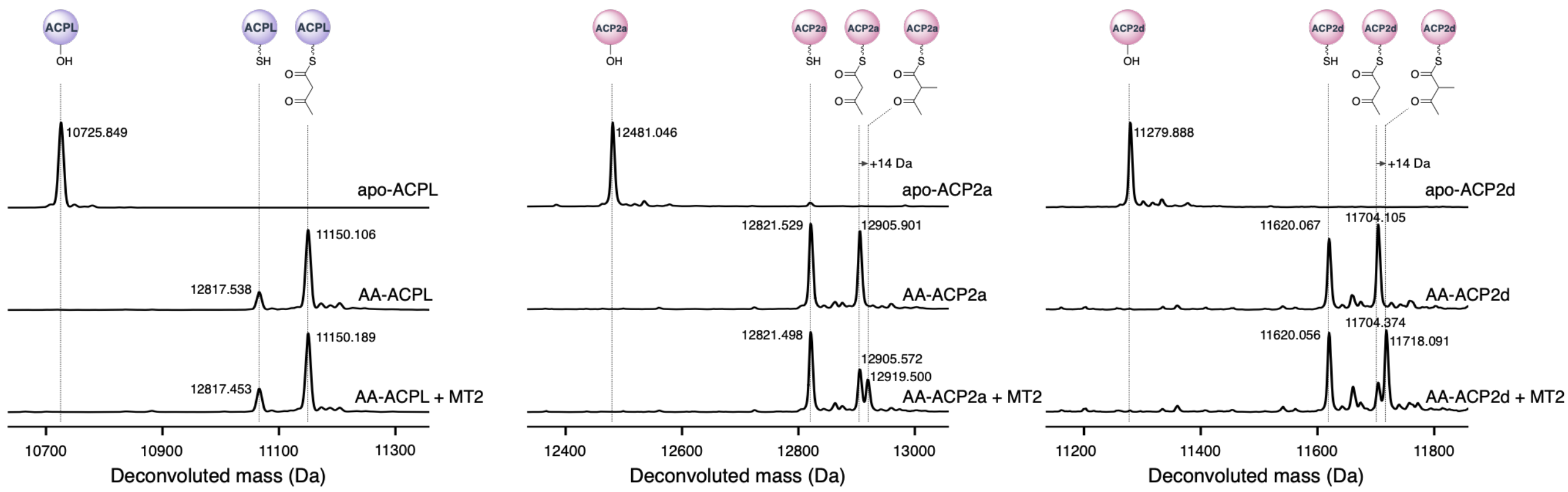

Figure S6. Deconvoluted mass spectra of acetoacetyl (AA)-ACP species after incubation with MT2. Acetoacetyl-ACP species underwent spontaneous hydrolysis during incubation, resulting in detectable holo-ACP species.

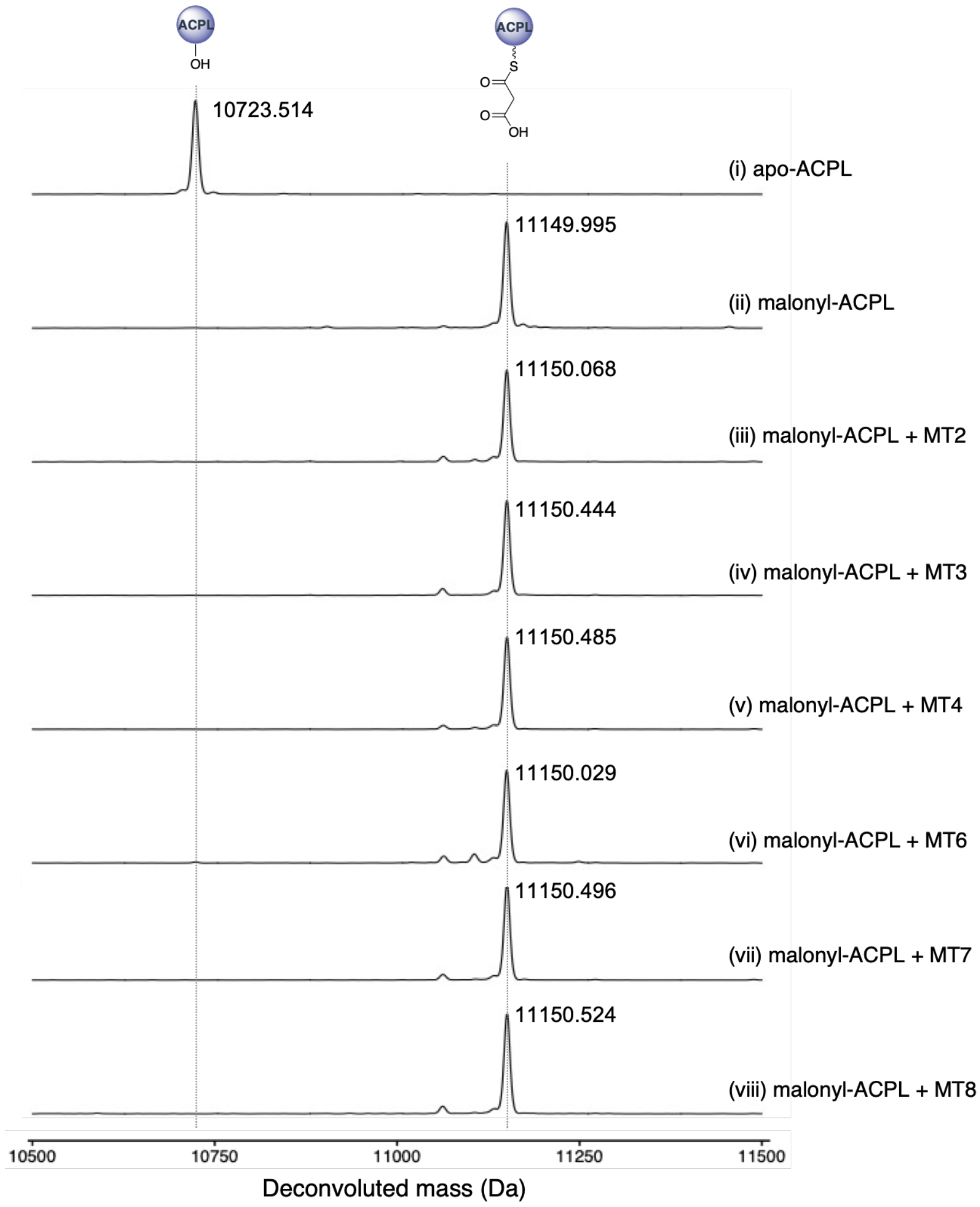

Figure S7. Deconvoluted mass spectra of malonyl-ACPL after incubation with each MT domain.

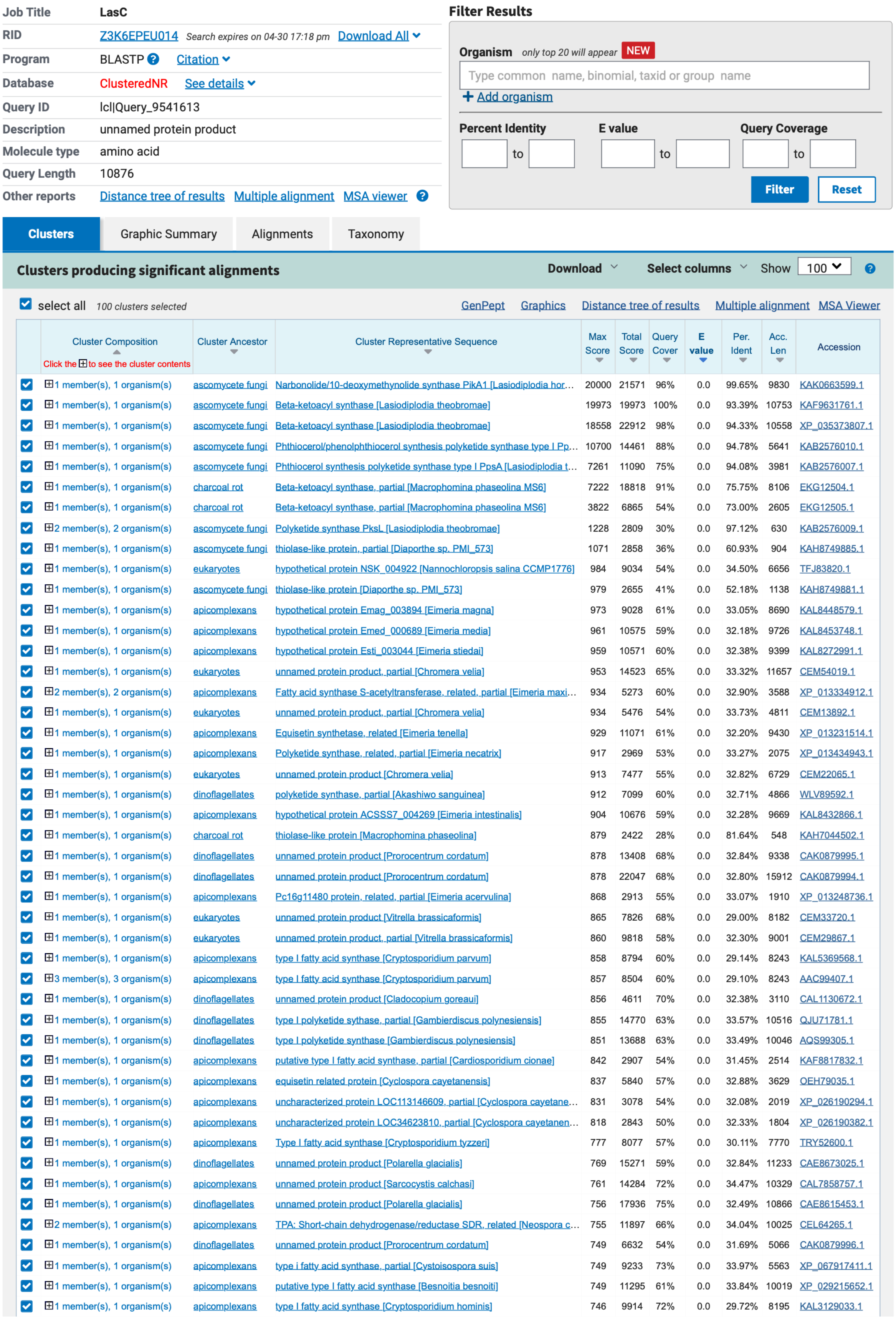

Figure S8. BLASTp search results using the LasC sequence as a query against the NCBI ClusteredNR database. The search was performed on April 29, 2026.

Job Title	LasD
RID	Z3K5AB7F014 *Search expires on 04-30 17:17 pm* Download All
Program	BLASTP Citation
Database	ClusteredNR See details
Query ID	lcl|Query_12521711
Description	unnamed protein product
Molecule type	amino acid
Query Length	5815
Other reports	Distance tree of results Multiple alignment MSA viewer

Filter Results

Organism *only top 20 will appear* NEW

Type common name, binomial, taxid or group name

+ Add organism

Percent Identity ___ to ___ E value ___ to ___ Query Coverage ___ to ___

Filter Reset

Clusters | Graphic Summary | Alignments | Taxonomy

Clusters producing significant alignments Download Select columns Show 100

select all *100 clusters selected* GenPept Graphics Distance tree of results Multiple alignment MSA Viewer

Cluster Composition (Click the ⊞ to see the cluster contents)	Cluster Ancestor	Cluster Representative Sequence	Max Score	Total Score	Query Cover	E value	Per. Ident	Acc. Len	Accession
4 member(s), 2 organism(s)	ascomycete fungi	Beta-ketoacyl synthase [Lasiodiplodia theobromae]	10167	10167	96%	0.0	92.39%	5548	KAF9631760.1
1 member(s), 1 organism(s)	charcoal rot	Beta-ketoacyl synthase [Macrophomina phaseolina MS6]	8339	8339	100%	0.0	72.83%	5799	EKG12506.1
1 member(s), 1 organism(s)	charcoal rot	thiolase-like protein, partial [Macrophomina phaseolina]	1194	1510	20%	0.0	82.04%	716	KAH7044499.1
1 member(s), 1 organism(s)	apicomplexans	Equisetin synthetase, related [Eimeria tenella]	709	5411	62%	0.0	31.51%	9430	XP_013231514.1
1 member(s), 1 organism(s)	apicomplexans	hypothetical protein ACSSS7_004269 [Eimeria intestinalis]	709	5332	64%	0.0	31.03%	9669	KAL8432866.1
1 member(s), 1 organism(s)	apicomplexans	hypothetical protein Emag_003894 [Eimeria magna]	705	5032	64%	0.0	31.23%	8690	KAL8448579.1
1 member(s), 1 organism(s)	apicomplexans	hypothetical protein Emed_000689 [Eimeria media]	702	5356	64%	0.0	30.91%	9726	KAL8453748.1
1 member(s), 1 organism(s)	apicomplexans	hypothetical protein EBH_0017680 [Eimeria brunetti]	696	5405	62%	0.0	31.54%	9680	CDJ53564.1
2 member(s), 1 organism(s)	apicomplexans	TPA: Short-chain dehydrogenase/reductase SDR, related [Neospora c…	679	5469	66%	0.0	31.77%	10025	CEL64265.1
1 member(s), 1 organism(s)	ascomycete fungi	beta-ketoacyl synthase, partial [Diaporthe sp. PMI_573]	648	964	15%	0.0	69.69%	478	KAH8749888.1
1 member(s), 1 organism(s)	apicomplexans	hypothetical protein Esti_003044 [Eimeria stiedai]	644	5043	63%	4e-180	30.96%	9399	KAL8272991.1
1 member(s), 1 organism(s)	dinoflagellates	unnamed protein product [Polarella glacialis]	633	6581	65%	6e-177	31.05%	11233	CAE8673025.1
1 member(s), 1 organism(s)	dinoflagellates	unnamed protein product [Polarella glacialis]	633	6509	66%	8e-177	31.05%	10866	CAE8615453.1
1 member(s), 1 organism(s)	ascomycete fungi	Beta-ketoacyl synthase [Lasiodiplodia theobromae]	625	7420	78%	2e-174	36.29%	10558	XP_035373807.1
1 member(s), 1 organism(s)	eukaryotes	unnamed protein product [Chromera velia]	623	3625	52%	4e-174	32.56%	6729	CEM22065.1
1 member(s), 1 organism(s)	ascomycete fungi	Phthiocerol synthesis polyketide synthase type I PpsA [Lasiodiplodia t…	619	2846	64%	4e-173	36.06%	3981	KAB2576007.1
1 member(s), 1 organism(s)	ascomycete fungi	Beta-ketoacyl synthase [Lasiodiplodia theobromae]	620	7211	78%	4e-173	36.66%	10753	KAF9631761.1
1 member(s), 1 organism(s)	charcoal rot	Beta-ketoacyl synthase, partial [Macrophomina phaseolina MS6]	619	5525	81%	8e-173	37.38%	8106	EKG12504.1
1 member(s), 1 organism(s)	eukaryotes	hypothetical protein NSK_004922 [Nannochloropsis salina CCMP1776]	618	4510	59%	2e-172	33.80%	6656	TFJ83820.1
1 member(s), 1 organism(s)	ascomycete fungi	Narbonolide/10-deoxymethynolide synthase PikA1 [Lasiodiplodia hor…	609	6675	82%	1e-169	35.88%	9830	KAK0663599.1
1 member(s), 1 organism(s)	apicomplexans	LOW QUALITY PROTEIN: PKSN polyketide synthase for alternapyron…	601	2001	51%	2e-169	31.07%	2556	XP_037878241.1
1 member(s), 1 organism(s)	apicomplexans	unnamed protein product [Sarcocystis calchasi]	600	5382	66%	5e-167	33.59%	10329	CAL7858757.1
1 member(s), 1 organism(s)	apicomplexans	type i fatty acid synthase, partial [Cystoisospora suis]	583	1294	41%	1e-166	33.07%	2000	XP_067919354.1
1 member(s), 1 organism(s)	bacteria	acyltransferase domain-containing protein [Planctomycetota bacterium]	558	909	25%	3e-166	36.77%	1068	MBM4057354.1
7 member(s), 7 organism(s)	apicomplexans	putative type I fatty acid synthase, partial [Toxoplasma gondii ARI]	595	1590	60%	3e-166	31.58%	3311	KYF39670.1
1 member(s), 1 organism(s)	apicomplexans	putative type I fatty acid synthase, partial [Toxoplasma gondii RUB]	597	4644	63%	5e-166	31.64%	8102	KFG59574.1
1 member(s), 1 organism(s)	eukaryotes	unnamed protein product, partial [Chrysoparadoxa australica]	574	1234	39%	9e-166	34.63%	1667	CAM9882194.1
1 member(s), 1 organism(s)	dinoflagellates	unnamed protein product [Prorocentrum cordatum]	596	10077	65%	1e-165	29.65%	15912	CAK0879994.1
2 member(s), 2 organism(s)	apicomplexans	type I fatty acid synthase, putative [Toxoplasma gondii ME49]	595	5520	64%	2e-165	31.58%	10021	XP_002370251.1
2 member(s), 2 organism(s)	apicomplexans	Fatty acid synthase S-acetyltransferase, related, partial [Eimeria maxi…	585	2559	61%	7e-163	31.33%	3588	XP_013334912.1
1 member(s), 1 organism(s)	apicomplexans	putative type I fatty acid synthase [Besnoitia besnoiti]	586	5463	67%	9e-163	33.10%	10019	XP_029215652.1
1 member(s), 1 organism(s)	apicomplexans	type i fatty acid synthase, partial [Cystoisospora suis]	583	3289	61%	4e-162	32.08%	5563	XP_067917411.1
1 member(s), 1 organism(s)	apicomplexans	putative type I fatty acid synthase, partial [Toxoplasma gondii COUG]	577	1677	54%	2e-161	31.96%	2629	PIL97818.1
1 member(s), 1 organism(s)	apicomplexans	hypothetical protein EPH_0047980 [Eimeria praecox]	562	1176	46%	5e-161	30.84%	1752	CDI87737.1
5 member(s), 5 organism(s)	apicomplexans	putative type I fatty acid synthase, partial [Toxoplasma gondii ARI]	577	2651	62%	3e-160	31.96%	4958	KYF39683.1
2 member(s), 2 organism(s)	apicomplexans	putative type I fatty acid synthase, partial [Toxoplasma gondii GAB2-2…	575	2629	62%	1e-159	33.05%	4402	KFG37333.1
1 member(s), 1 organism(s)	apicomplexans	uncharacterized protein LOC113146609, partial [Cyclospora cayetane…	563	1453	58%	2e-159	31.93%	2019	XP_026190294.1
1 member(s), 1 organism(s)	eukaryotes	unnamed protein product, partial [Chromera velia]	575	6457	77%	2e-159	31.30%	11657	CEM54019.1
1 member(s), 1 organism(s)	apicomplexans	uncharacterized protein LOC34623810, partial [Cyclospora cayetanen…	558	1212	48%	2e-159	32.03%	1804	XP_026190382.1
1 member(s), 1 organism(s)	eukaryotes	type i fatty acid synthase, partial [Nannochloropsis oceanica]	553	924	26%	2e-159	36.97%	1597	GAB5036342.1
1 member(s), 1 organism(s)	apicomplexans	Polyketide synthase, related, partial [Eimeria acervulina]	561	1387	56%	6e-159	32.35%	2052	XP_013248779.1
1 member(s), 1 organism(s)	dinoflagellates	unnamed protein product [Prorocentrum cordatum]	573	6083	64%	6e-159	29.27%	9338	CAK0879995.1
1 member(s), 1 organism(s)	apicomplexans	hypothetical protein EMWEY_00055660, partial [Eimeria maxima]	560	1371	49%	6e-159	32.58%	1992	XP_013335773.1
1 member(s), 1 organism(s)	enterobacteria	SDR family NAD(P)-dependent oxidoreductase [Serratia marcescens]	539	1093	35%	5e-158	34.53%	1229	HIO6998240.1
1 member(s), 1 organism(s)	dinoflagellates	unnamed protein product [Effrenium voratum]	569	3314	48%	9e-158	29.53%	4531	CAJ1415650.1
1 member(s), 1 organism(s)	ascomycete fungi	Phthiocerol/phenolphthiocerol synthesis polyketide synthase type I Pp…	566	3800	77%	6e-157	30.90%	5641	KAB2576010.1

Figure S9. BLASTp search results using the LasD sequence as a query against the NCBI ClusteredNR database. The search was performed on April 29, 2026.

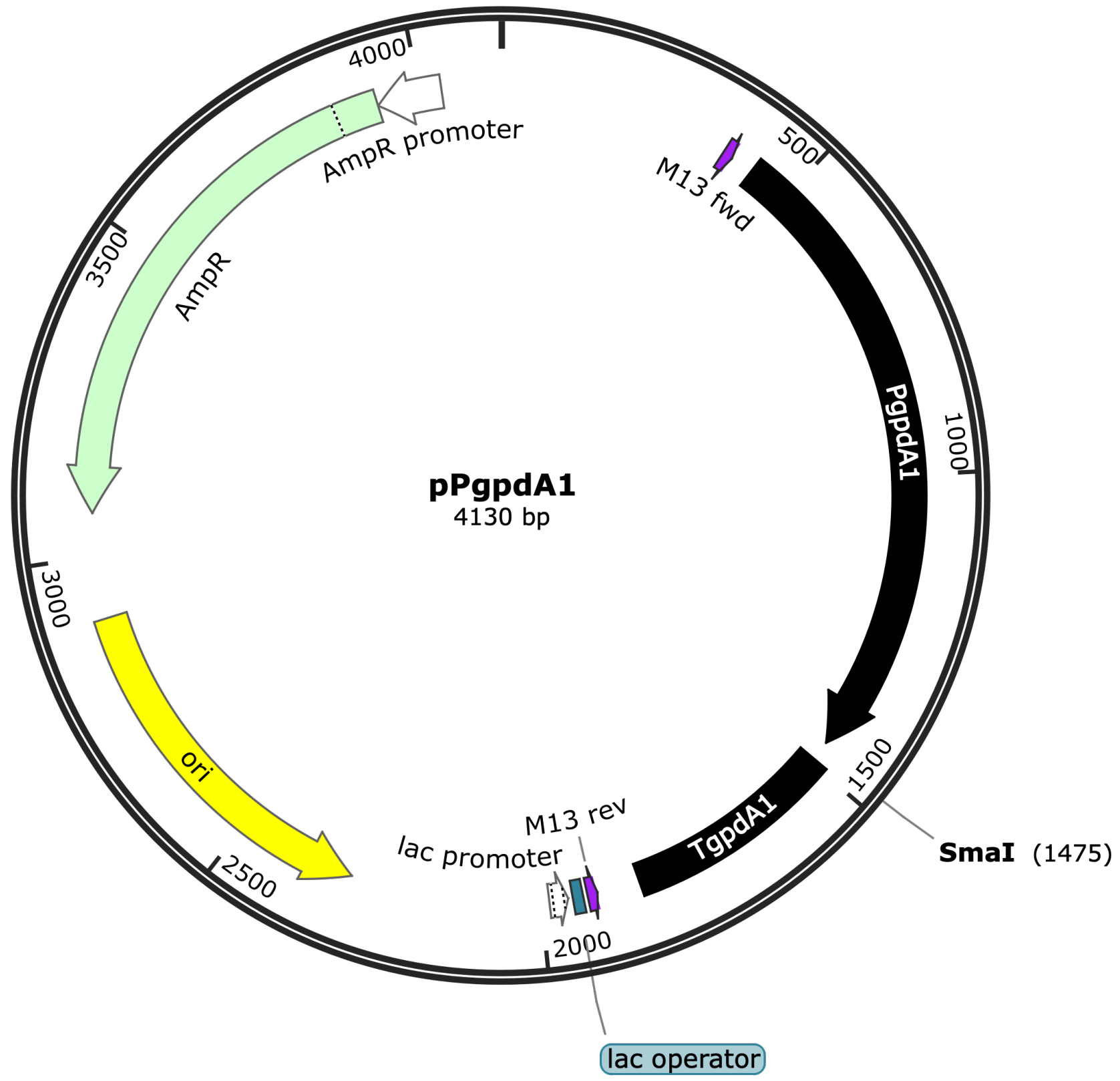

Figure S10. Vector map of pPgpdA1.

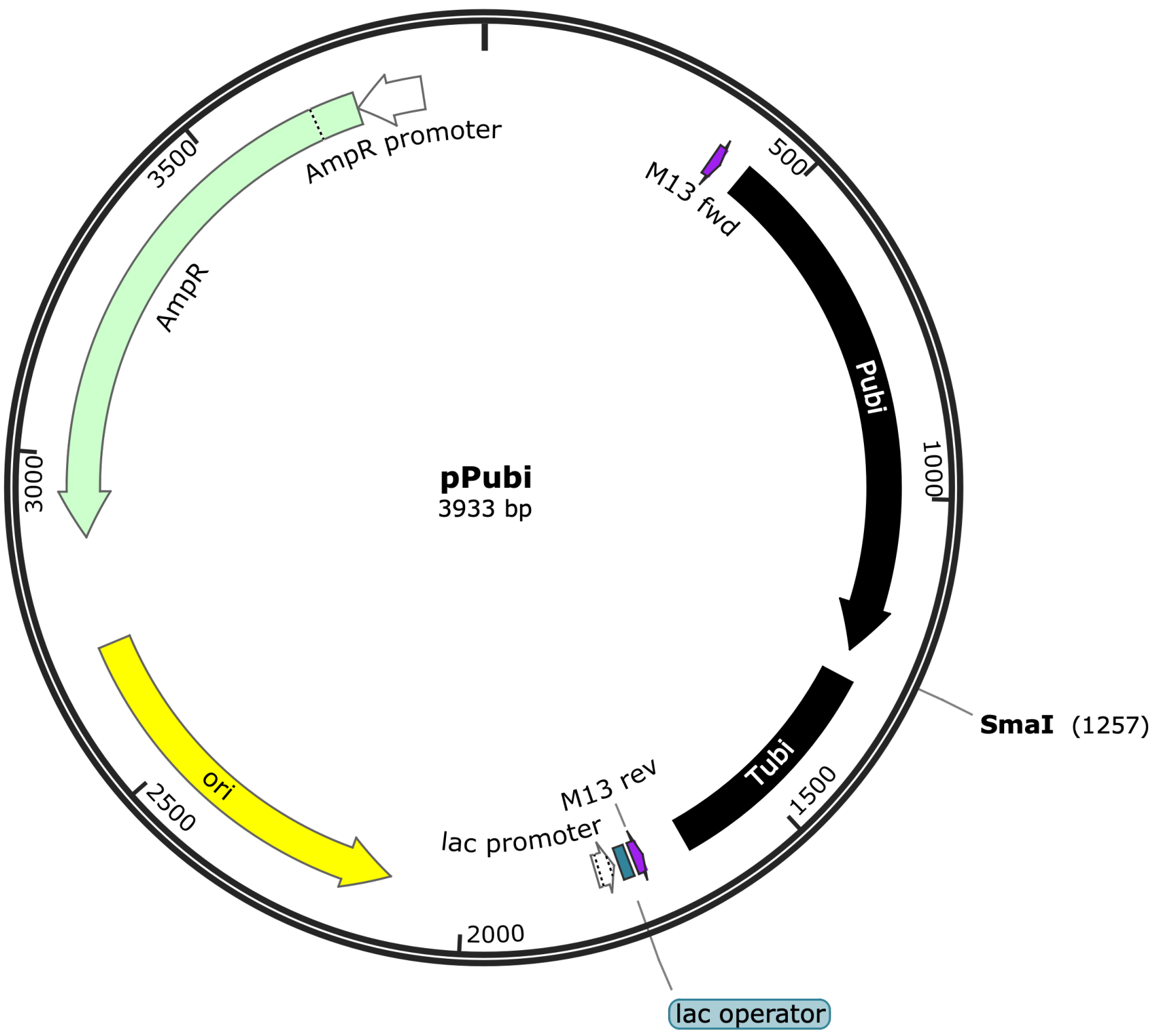

Figure S11. Vector map of pPubi.

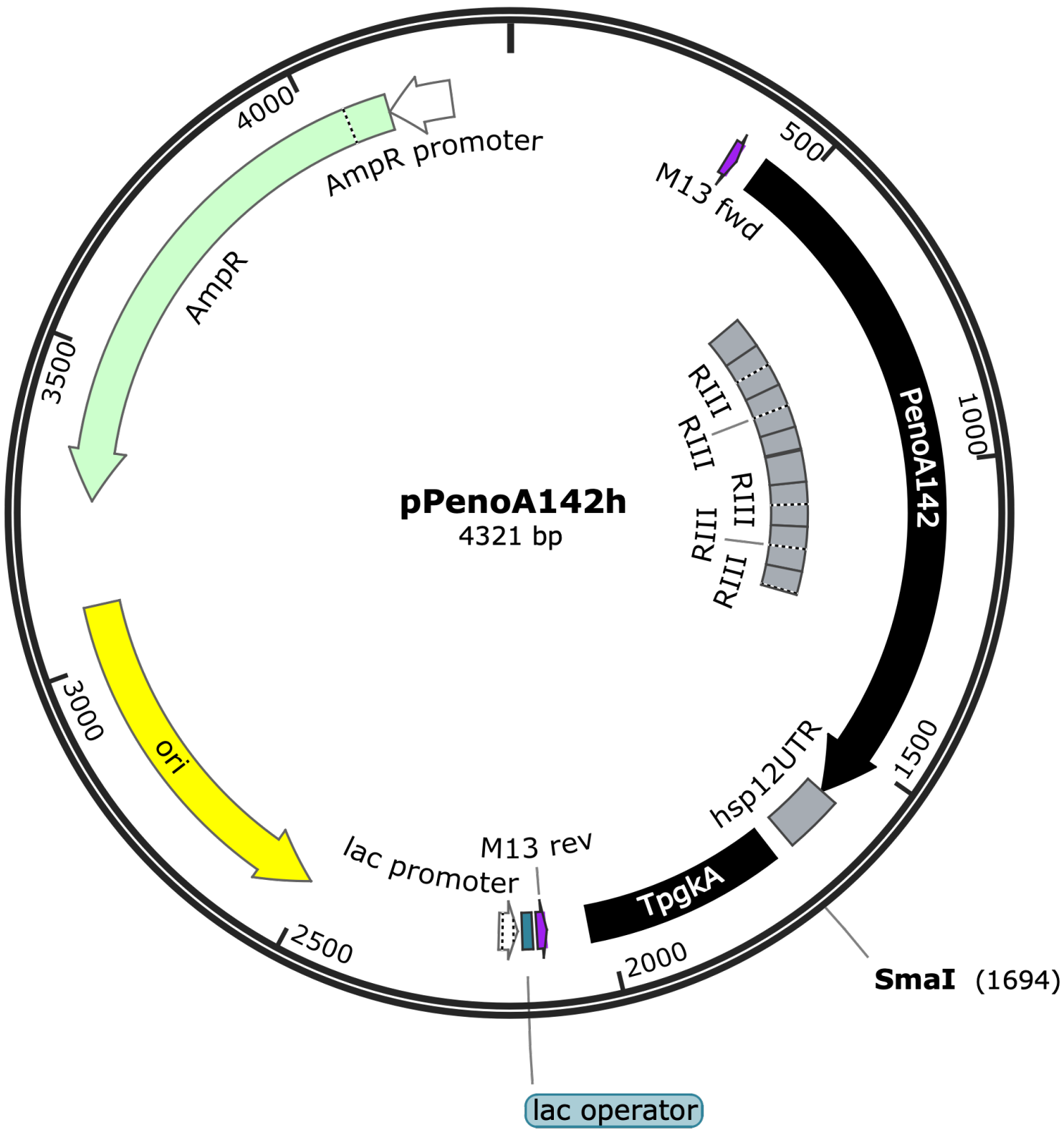

Figure S12. Vector map of pPenoA142h.

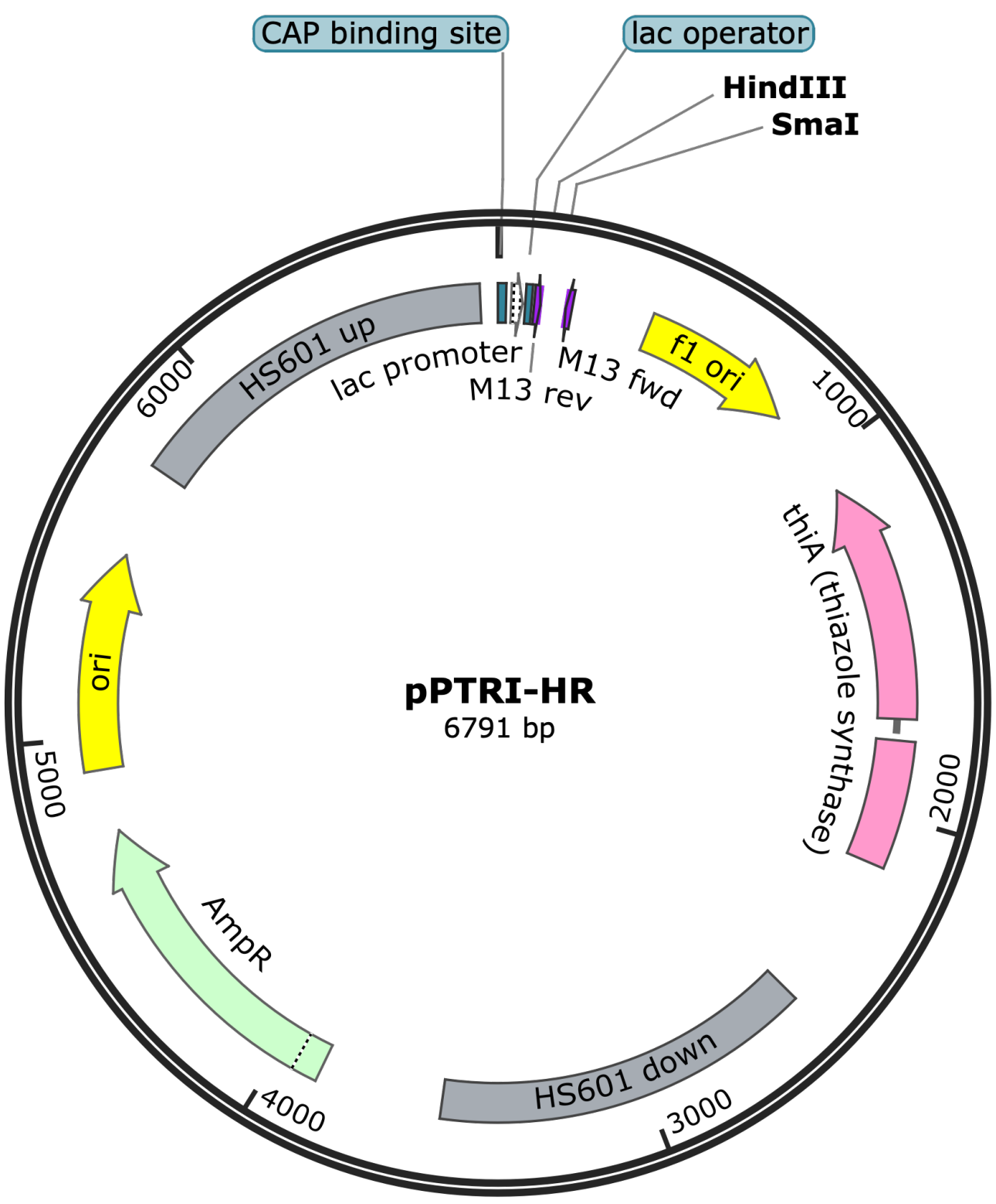

Figure S13. Vector map of pPTRI-HR.

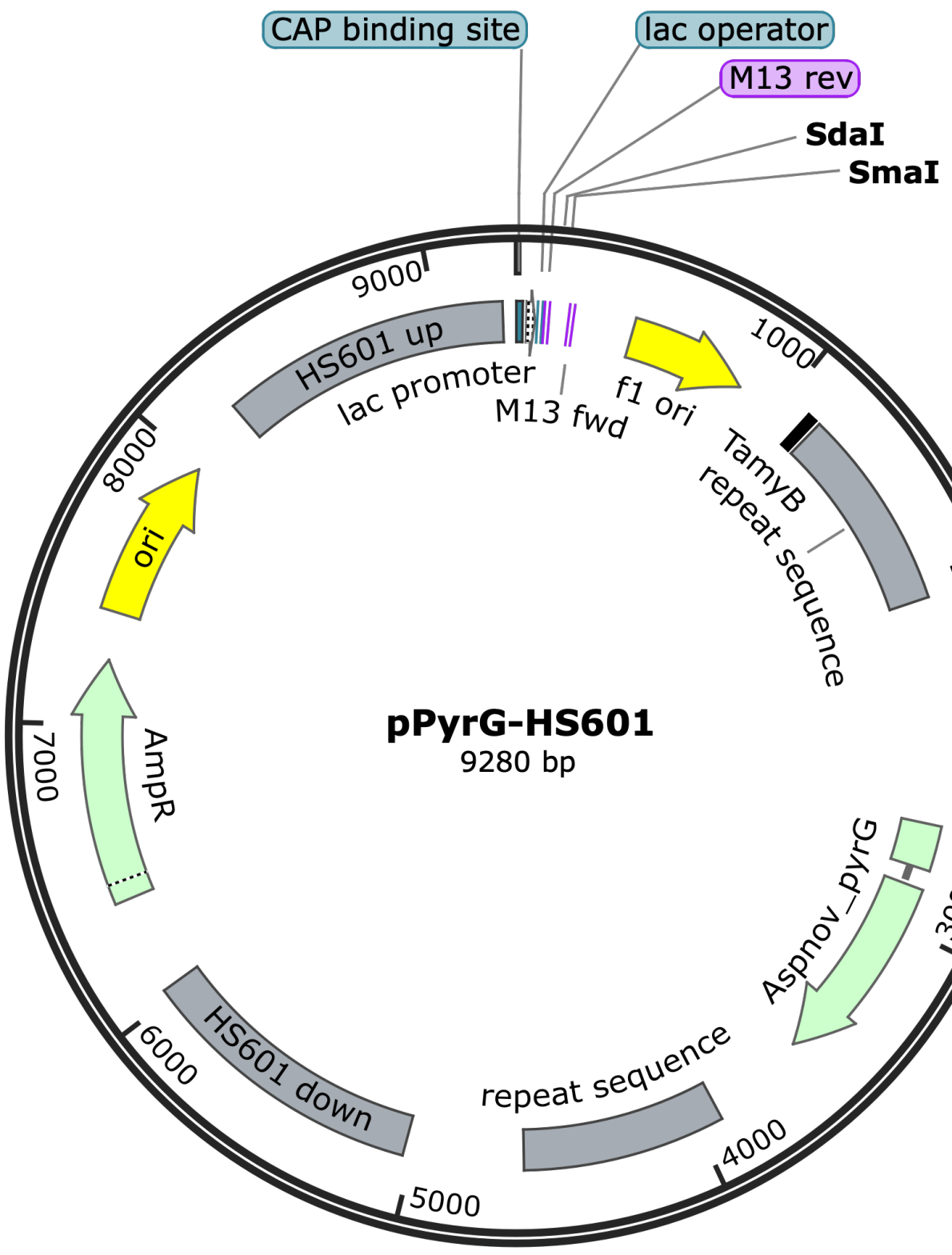

Figure S14. Vector map of pPyrG-HS601.

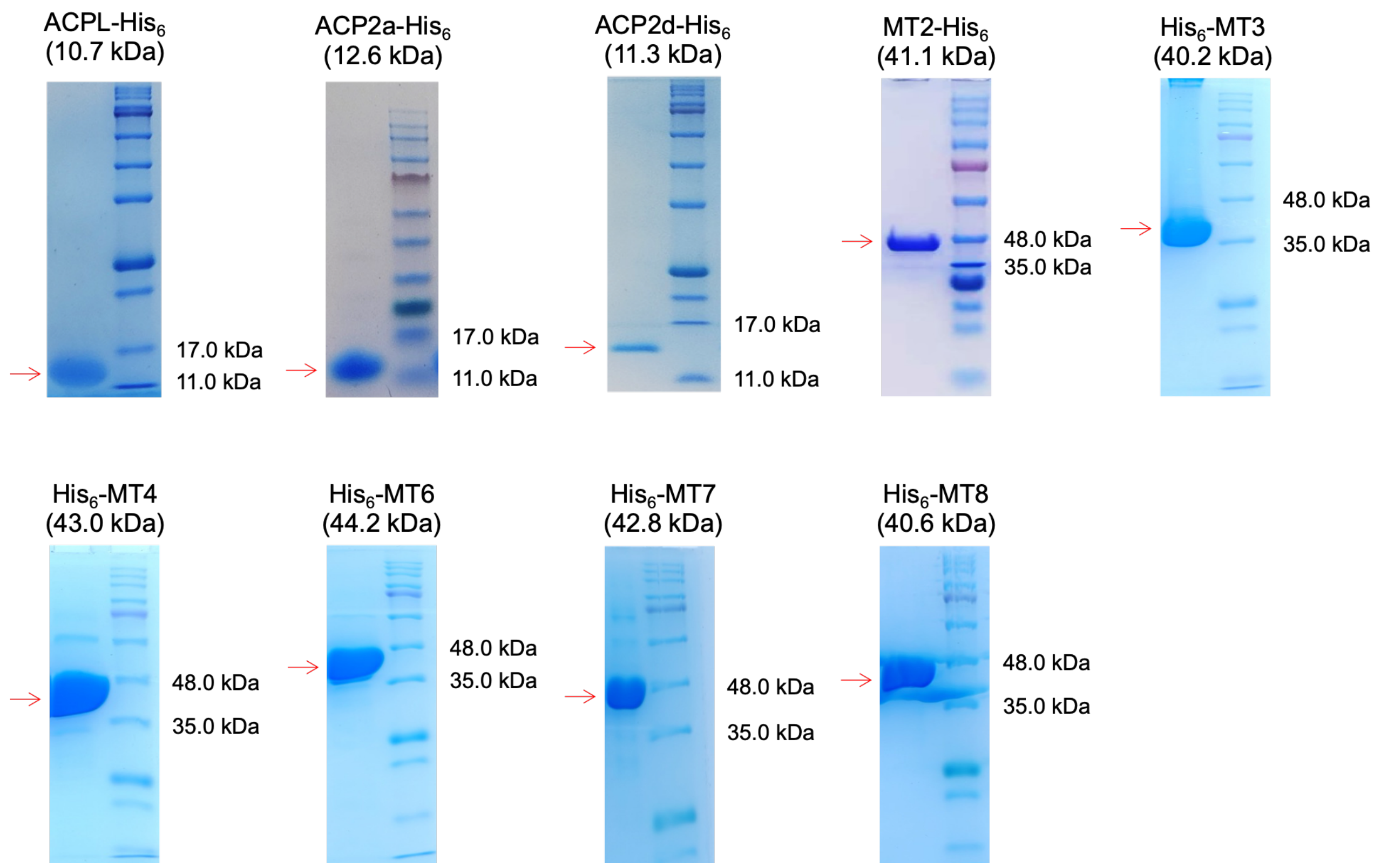

Figure S15. SDS-PAGE analysis of the purified proteins.

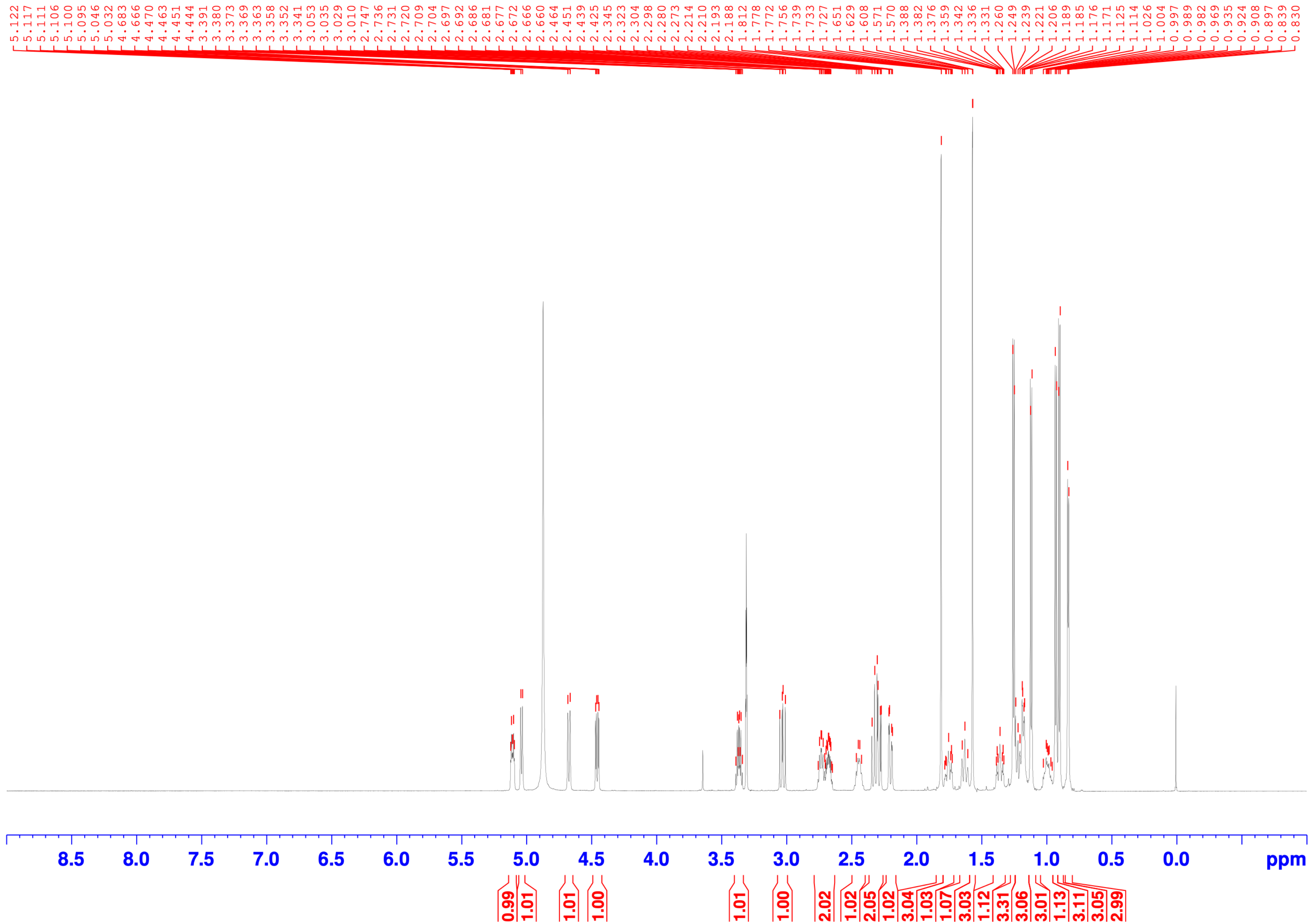

Figure S16. ^{1}H NMR spectrum of **1** in CD_3OD at 600 MHz.

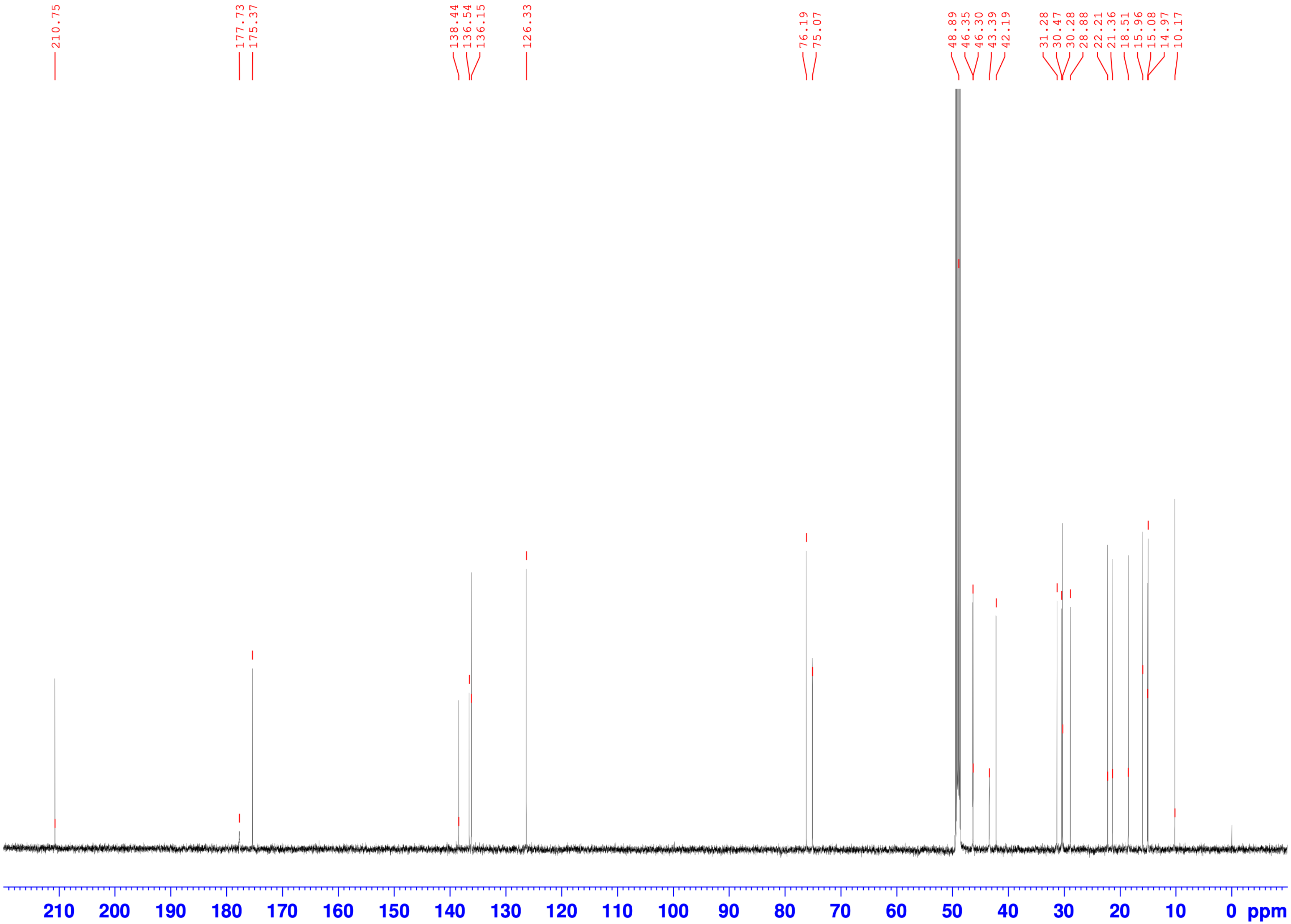

Figure S17. $^{13}C\{^{1}H\}$ NMR spectrum of **1** in CD_3OD at 150 MHz.

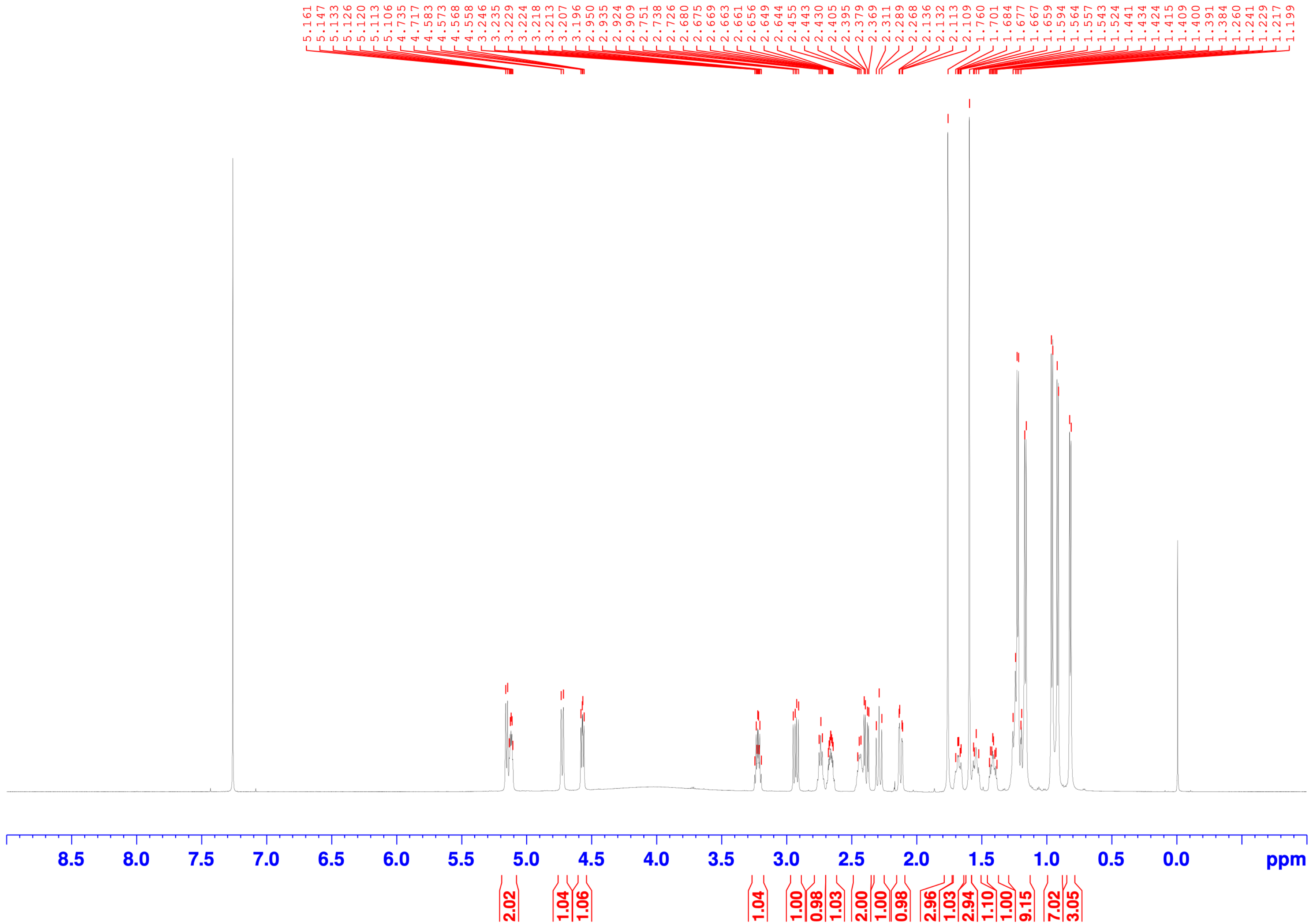

Figure S18. ^{1}H NMR spectrum of **1** in $CDCl_3$ at 600 MHz.

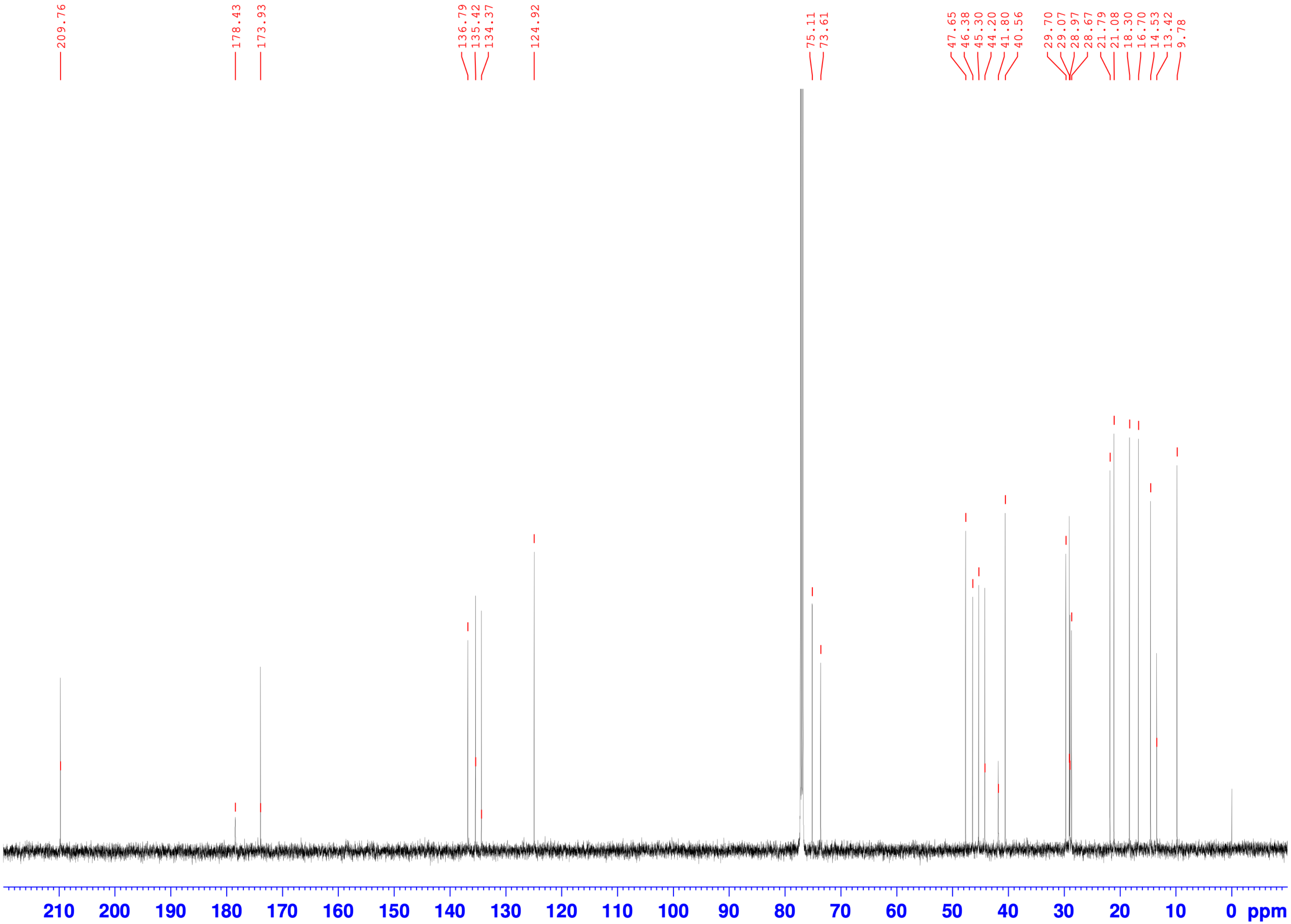

Figure S19. $^{13}C\{^{1}H\}$ NMR spectrum of **1** in $CDCl_3$ at 150 MHz.

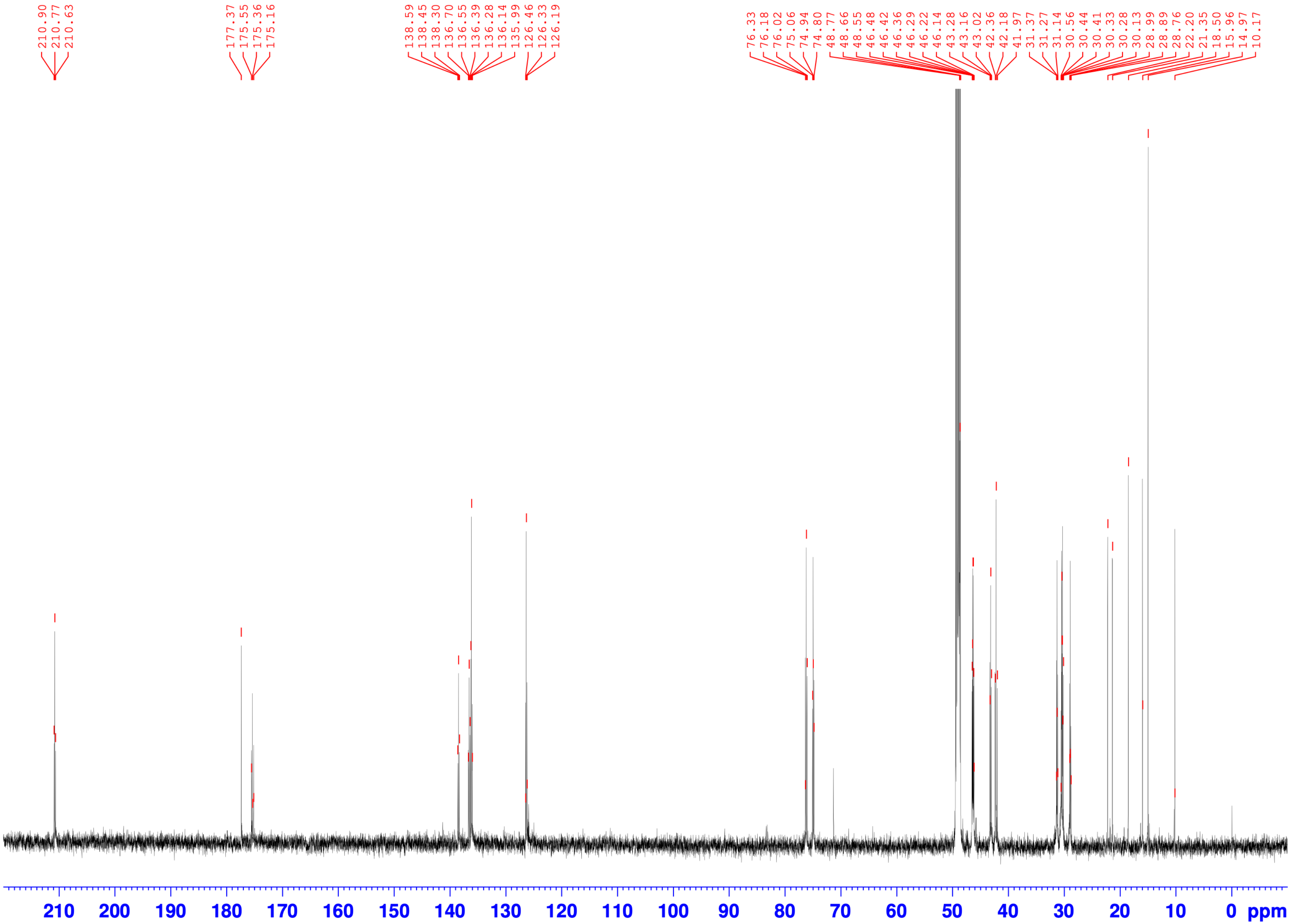

Figure S20. $^{13}C\{^{1}H\}$ NMR spectrum of **1** labeled with sodium [1,2-$^{13}C_2$]acetate in CD_3OD at 150 MHz.

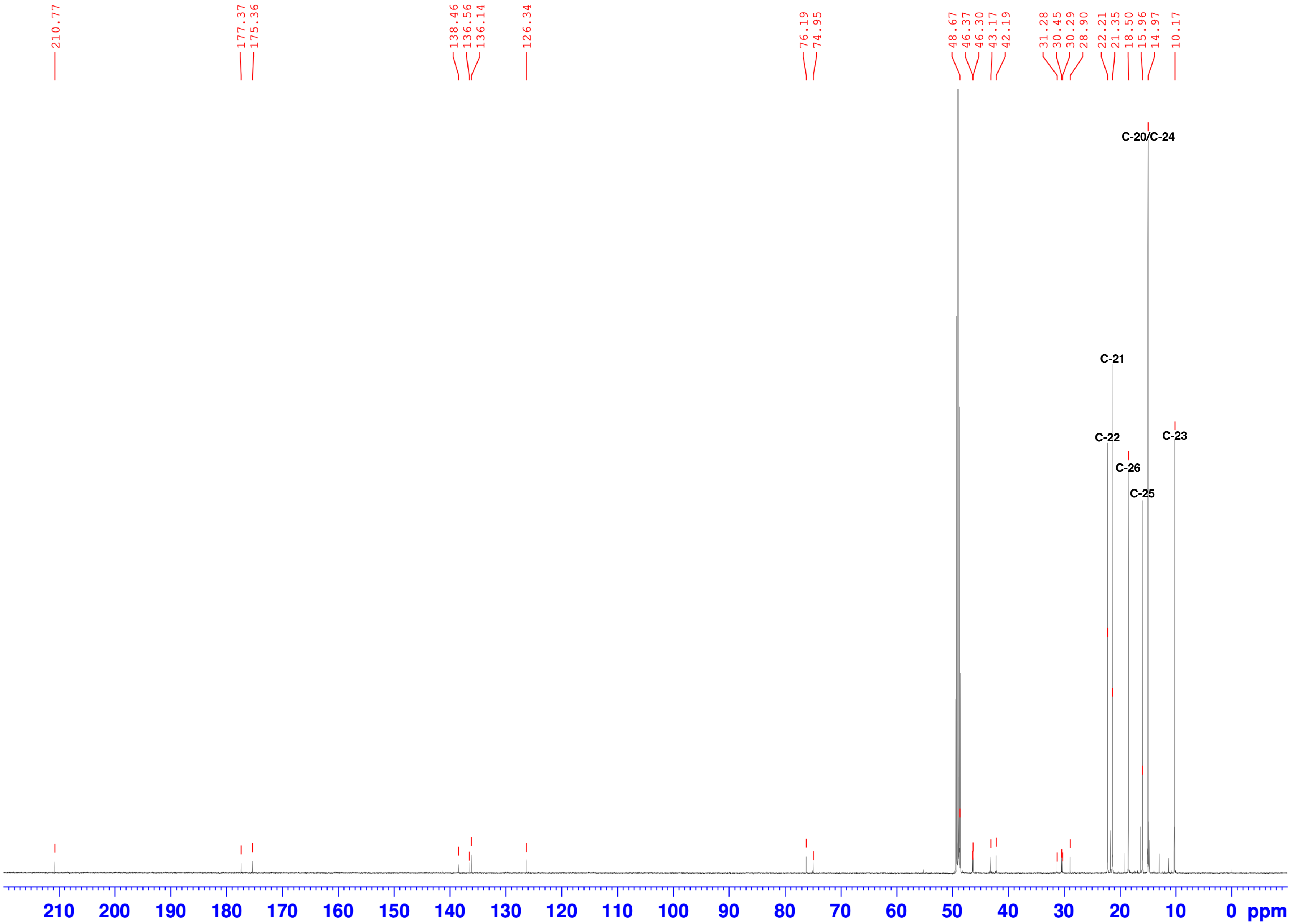

Figure S21. $^{13}C\{^{1}H\}$ NMR spectrum of **1** labeled with [methyl-^{13}C]methionine in CD_3OD at 150 MHz.

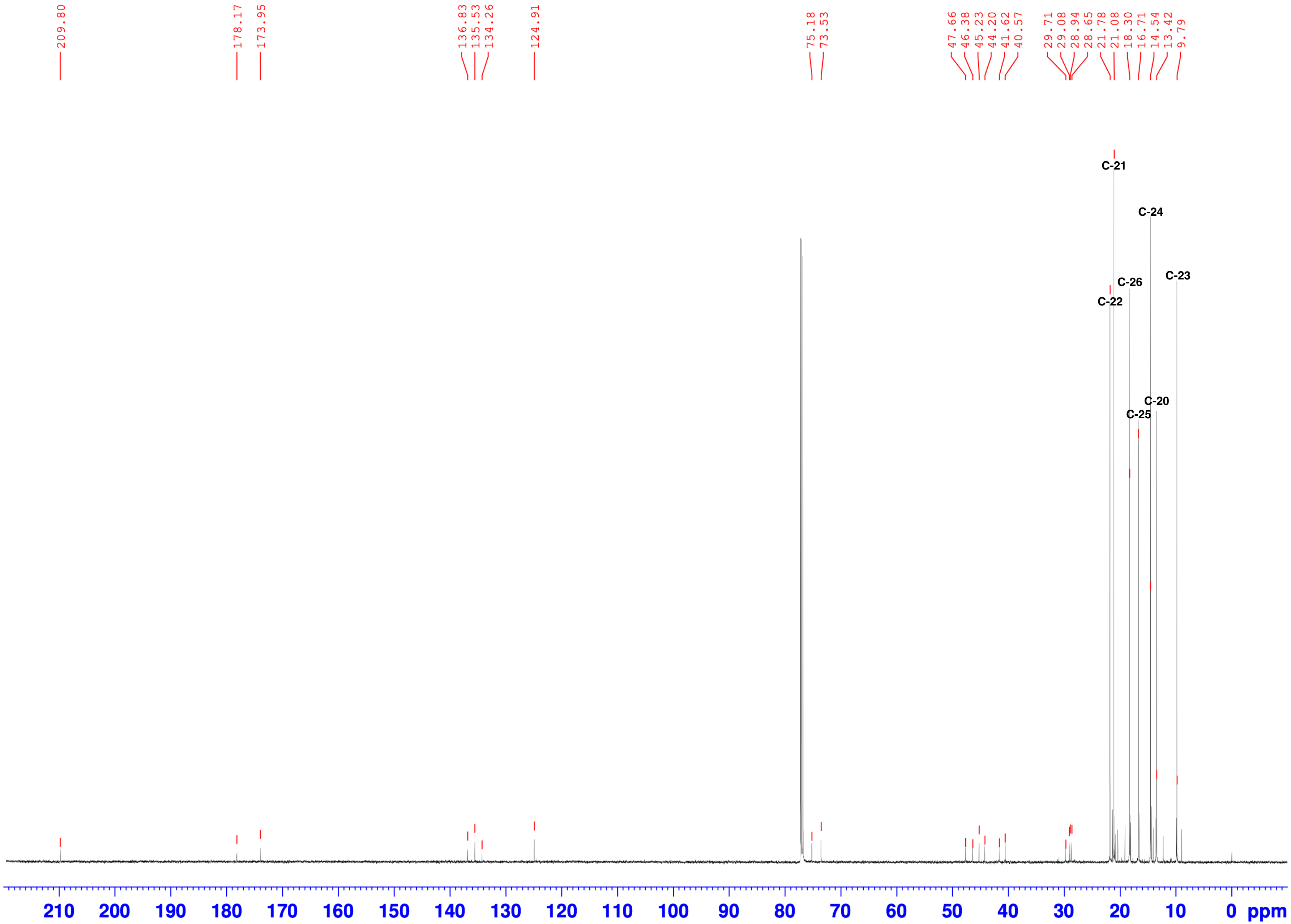

Figure S22. $^{13}C\{^{1}H\}$ NMR spectrum of **1** labeled with [methyl-^{13}C]methionine in $CDCl_3$ at 150 MHz.

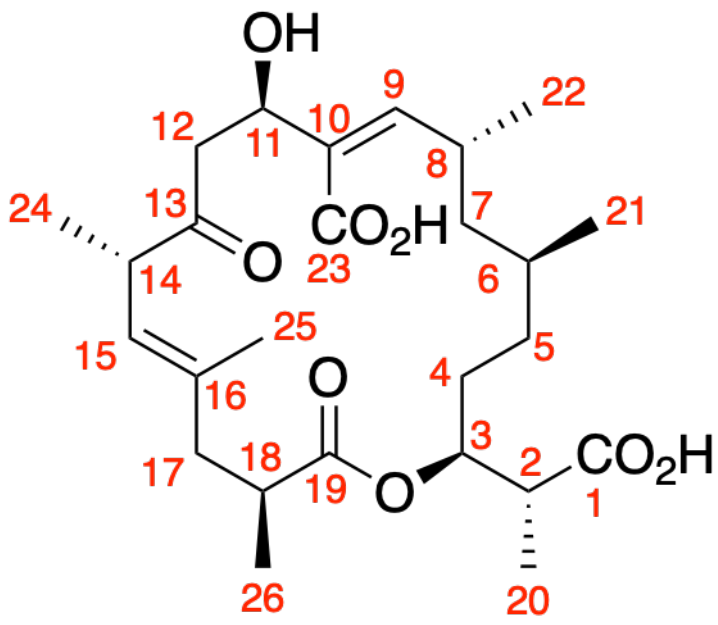

2

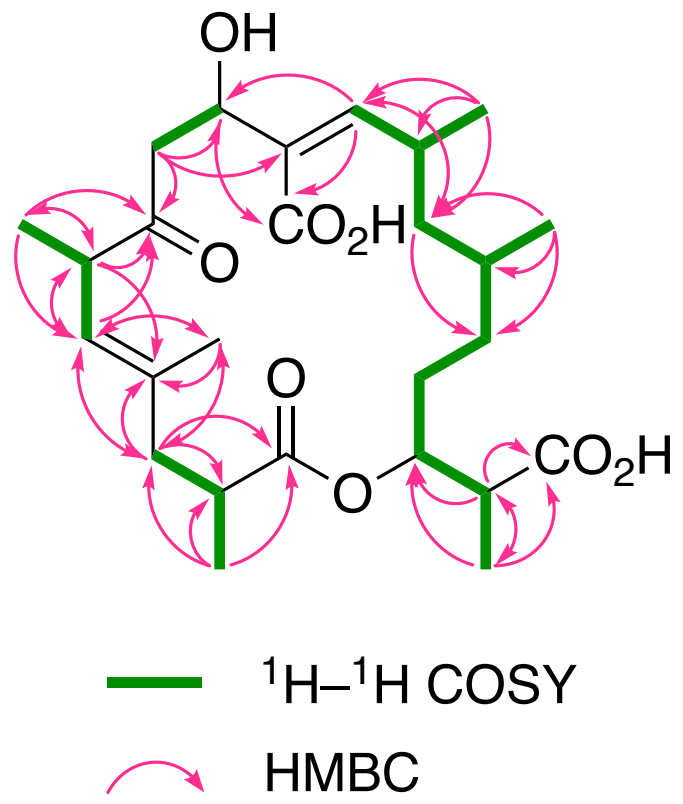

position	δ_C, type	δ_H, mult. (J in Hz)
1	179.2, C	
2	41.6, CH	2.82, dq (8.7, 7.0)
3	73.4, CH	5.16, dt (9.1, 3.7)
4	28.8, CH_2	1.74, m
		1.44, m
5	28.0, CH_2	1.65, m
		0.96, m
6	30.6, CH	1.17, m
7	47.0, CH_2	1.33, m
		1.26, m
8	31.0, CH	3.46, m
9	158.9, CH	6.15, d (9.5)
10	128.4, C	
11	74.2, CH	4.67, dd (10.0, 5.2)
12	46.8, CH_2	3.26, dd (15.1, 10.0)
		2.64, dd (15.1, 5.0)
13	209.1, C	
14	47.8, CH	3.30, dq (10.3, 6.5)
15	124.2, CH	4.60, d (10.3)
16	138.0, C	
17	44.0, CH_2	2.30, t (12.7)
		1.98, brd (11.3)
18	41.0, CH	2.60, m
19	174.2, C	
20	14.1, CH_3	1.18, d (7.0)
21	20.8, CH_3	0.86, d (6.4)
22	21.3, CH_3	1.02, d (6.7)
23	170.9, C	
24	14.4, CH_3	0.96, d (6.5)
25	16.6, CH_3	1.74, s
26	18.3, CH_3	1.19, d (7.0)

^{1}H NMR: 600 MHz, ^{13}C NMR: 150 MHz (in $CDCl_3$)

Figure S23. NMR data of lasiodiplolide A (**2**).

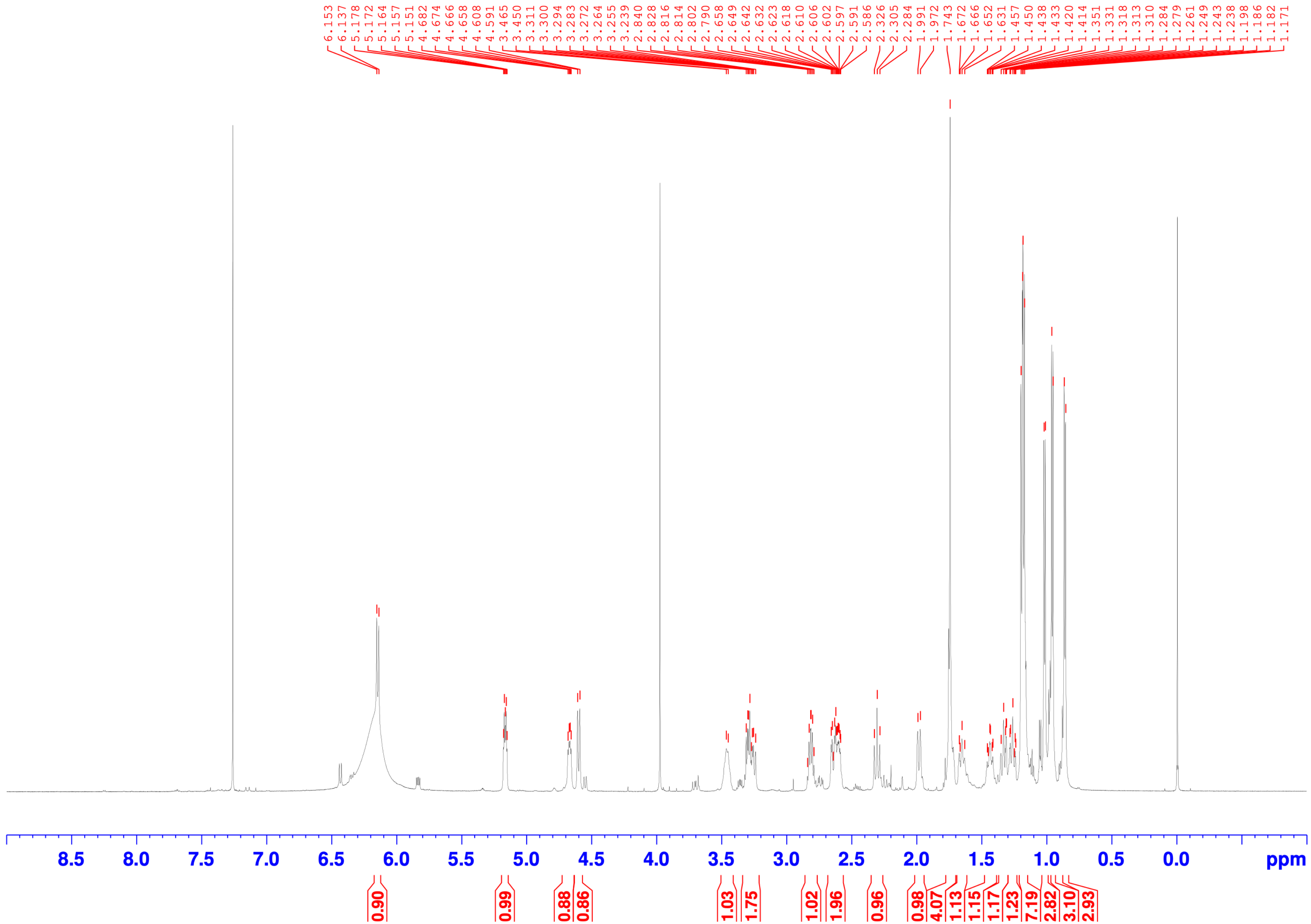

Figure S24. ^{1}H NMR spectrum of **2** in $CDCl_3$ at 600 MHz.

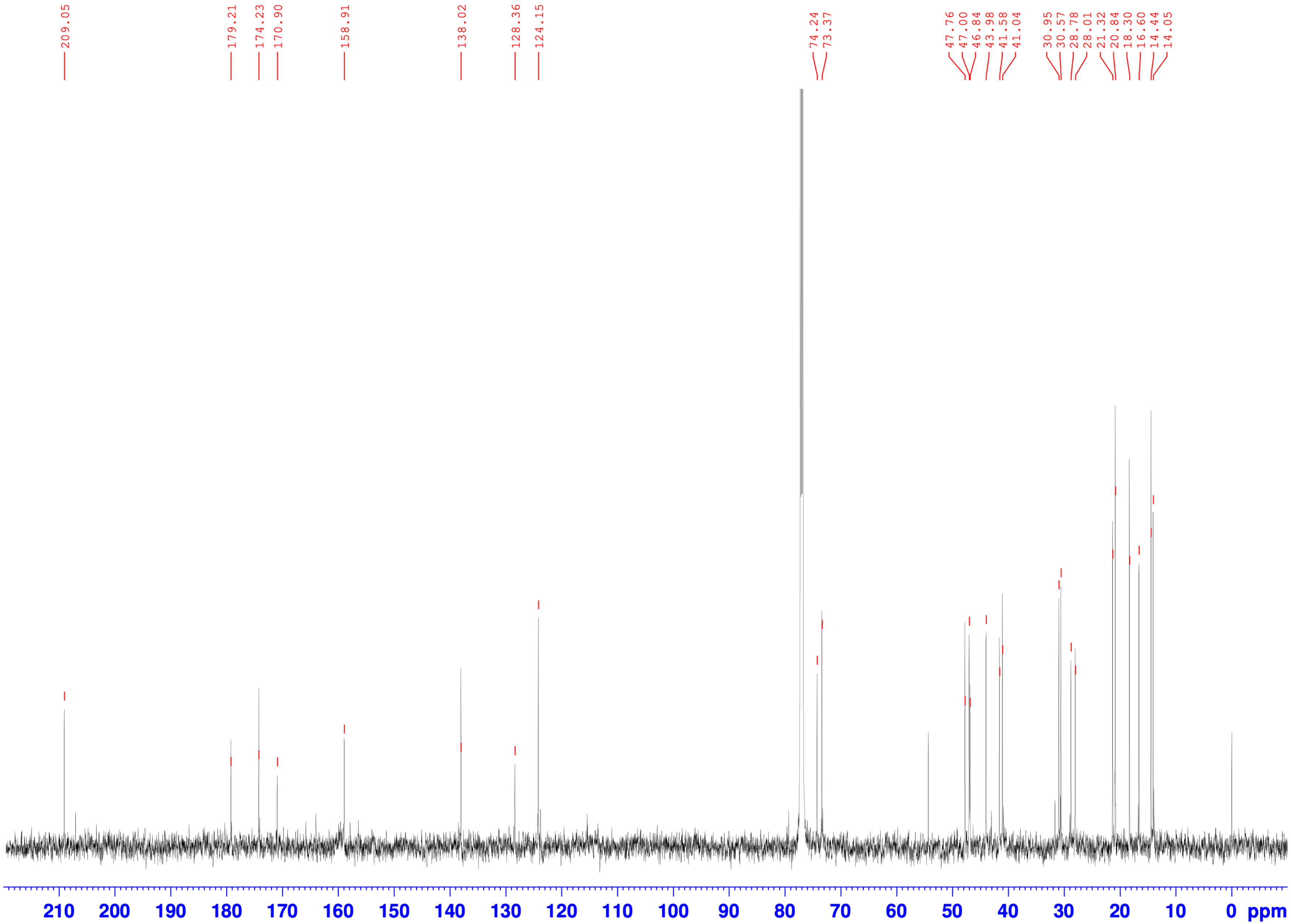

Figure S25. $^{13}C\{^{1}H\}$ NMR spectrum of **2** in $CDCl_3$ at 150 MHz.

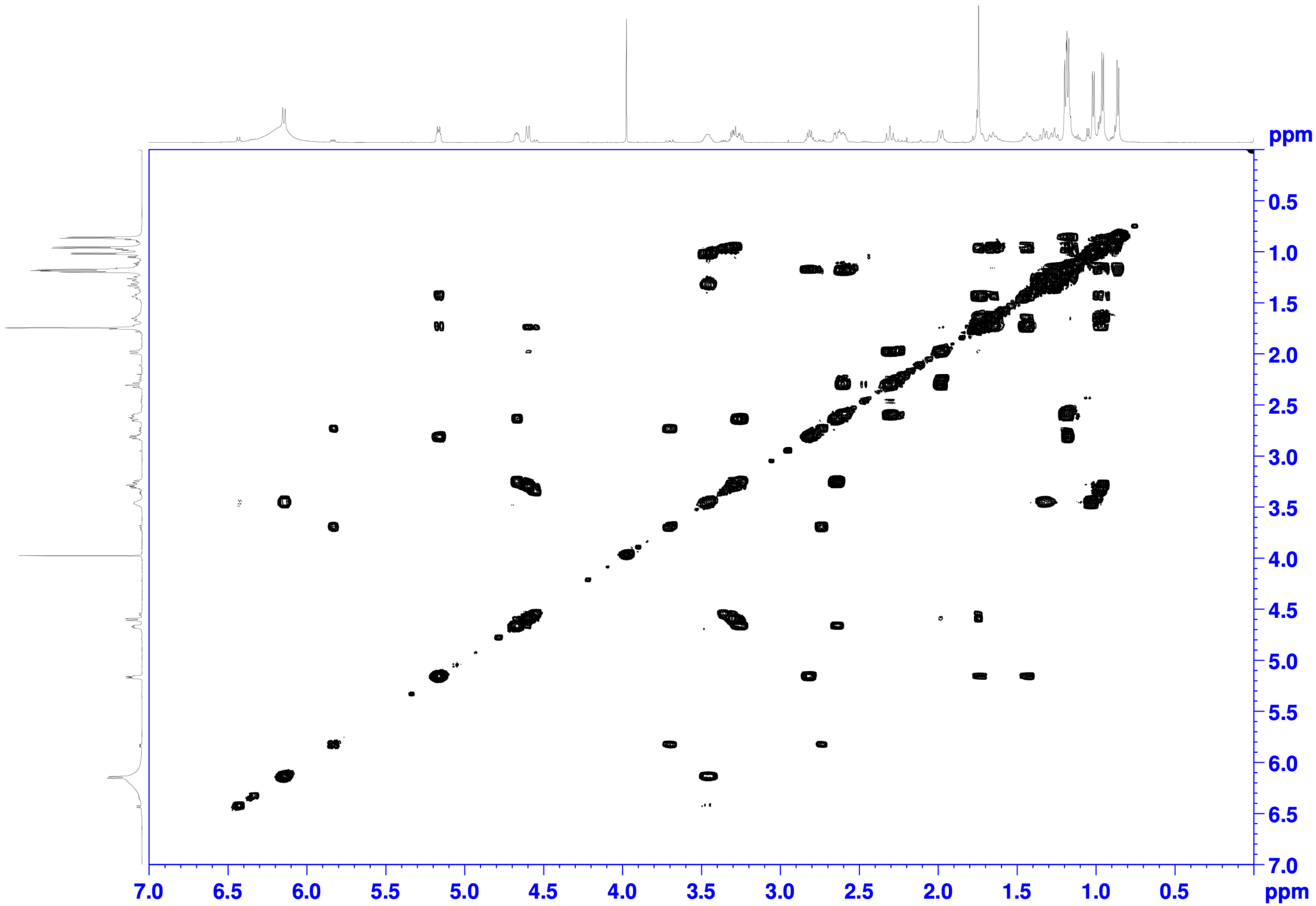

Figure S26. ^{1}H-^{1}H COSY spectrum of **2** in $CDCl_3$.

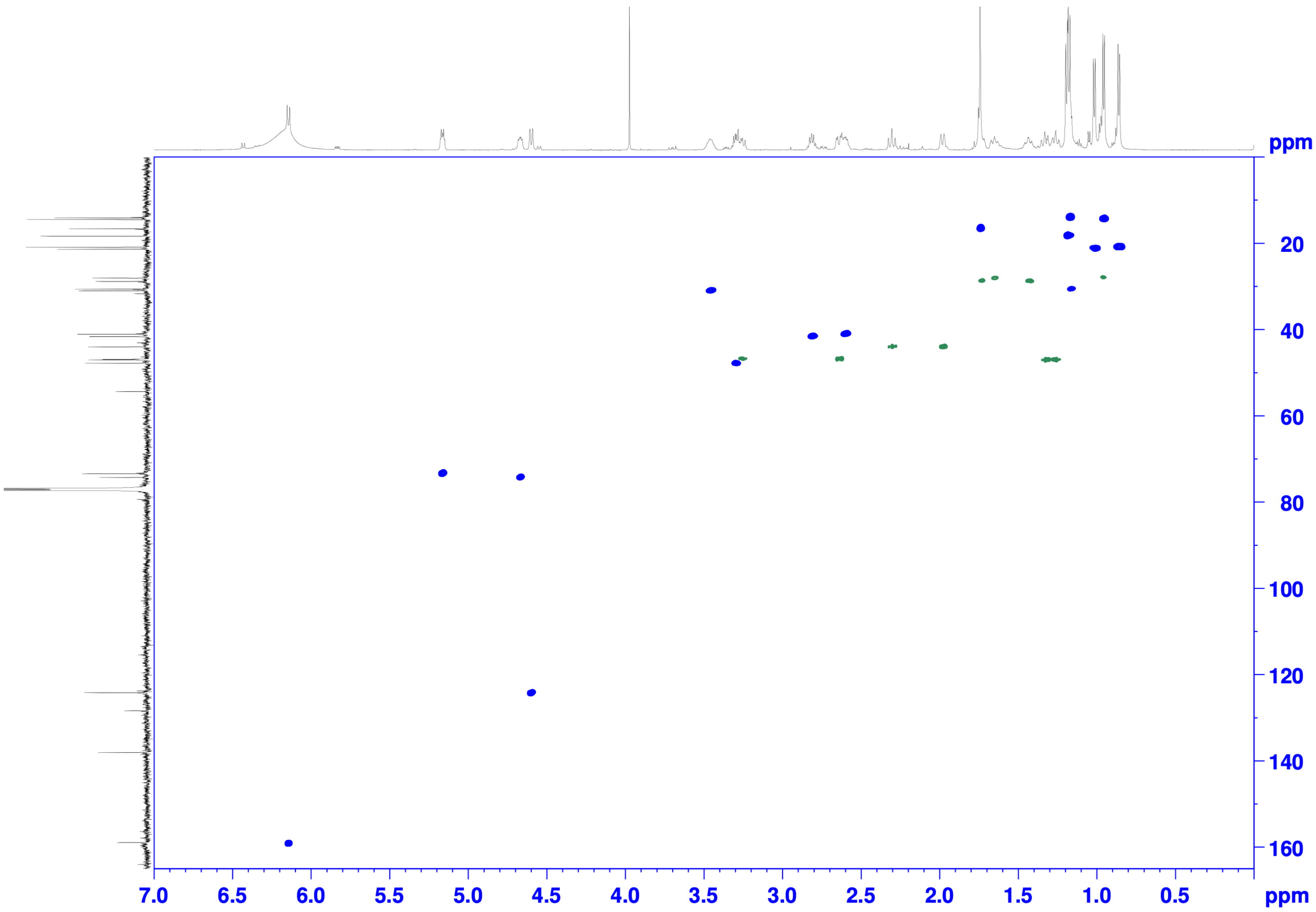

Figure S27. HSQC spectrum of **2** in $CDCl_3$.

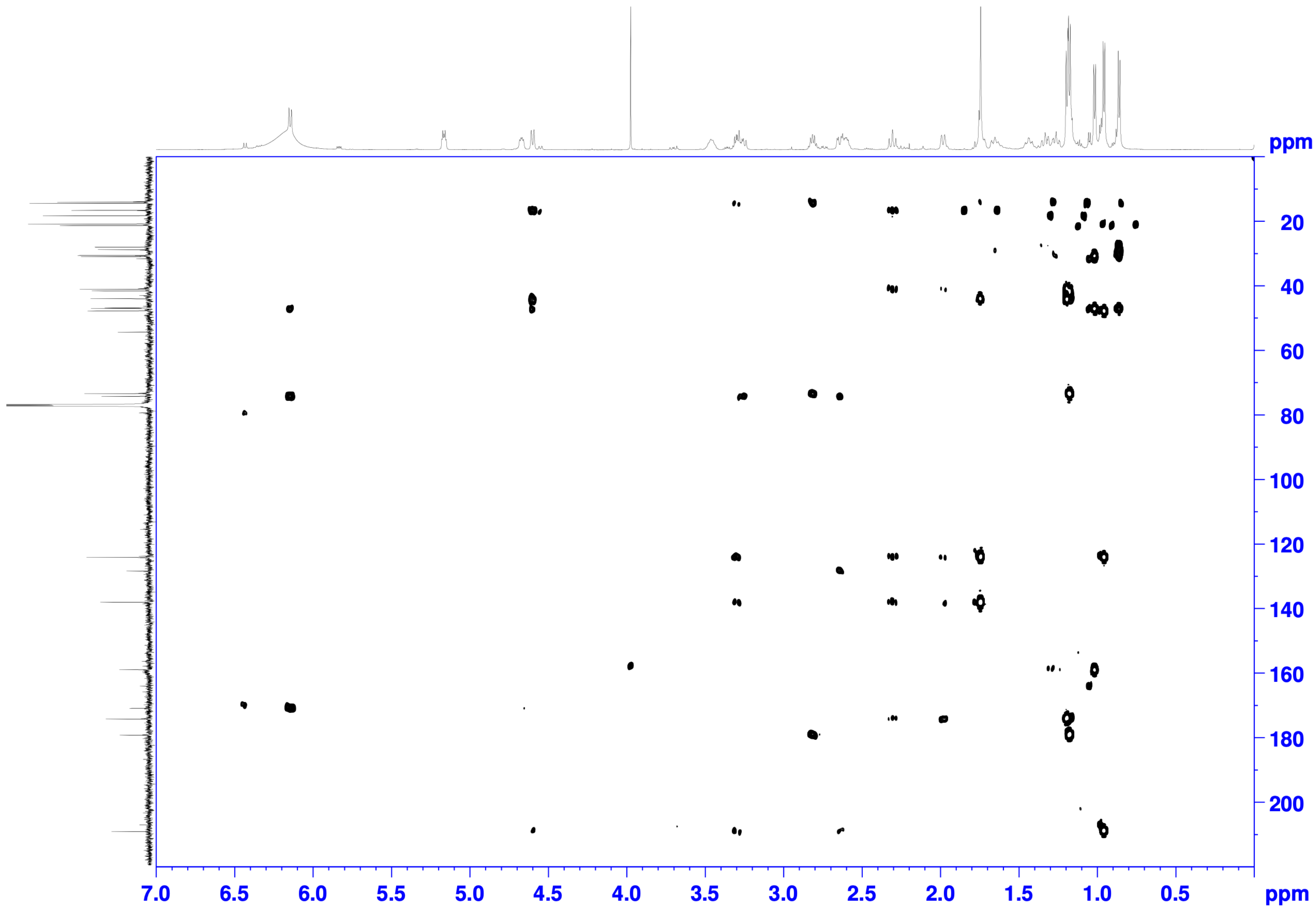

Figure S28. HMBC spectrum of **2** in $CDCl_3$.

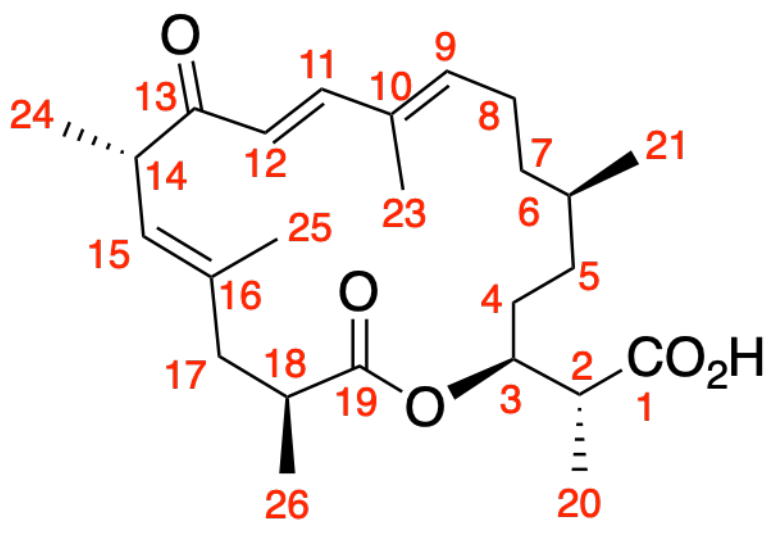

3′

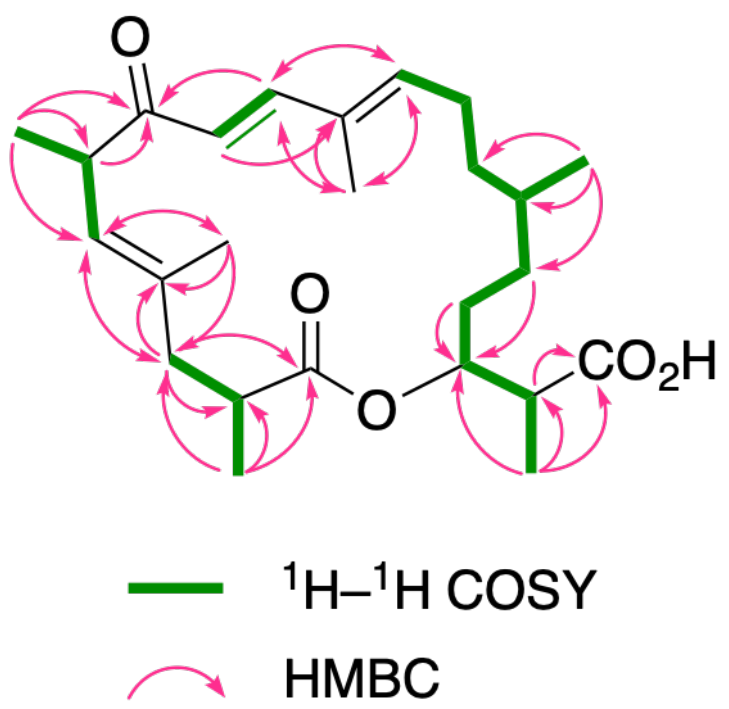

position	δ_C, type	δ_H, mult. (*J* in Hz)
1	177.3, C	
2	41.8, CH	2.62, m
3	75.7, CH	4.85, m
4	28.0, CH_2	1.79, m
		1.45, m
5	33.3, CH_2	1.21, m
6	31.0, CH	1.40, m
7	35.4, CH_2	1.62, m
		1.31, m
8	27.2, CH_2	2.34, m
		2.29, m
9	144.3, CH	6.07, dd (9.3, 6.4)
10	134.8, C	
11	150.4, CH	7.13, d (16.1)
12	125.1, CH	5.98, d (16.0)
13	205.2, C	
14	44.2, CH	3.76, dq (9.7, 6.7)
15	126.4, CH	5.02, dd (9.7, 1,1)
16	136.9, C	
17	42.6, CH_2	2.21, m
18	39.9, CH	2.62, m
19	176.4, C	
20	10.6, CH_3	1.11, d (7.2)
21	21.3, CH_3	0.92, d (6.6)
23	12.6, CH_3	1.82, s
24	15.7, CH_3	1.07, d (6.6)
25	18.9, CH_3	1.82, s
26	18.8, CH_3	1.17, d (7.2)

^1H NMR: 600 MHz, ^{13}C NMR: 150 MHz (in CD_3OD)

Figure S29. NMR data of **3′**.

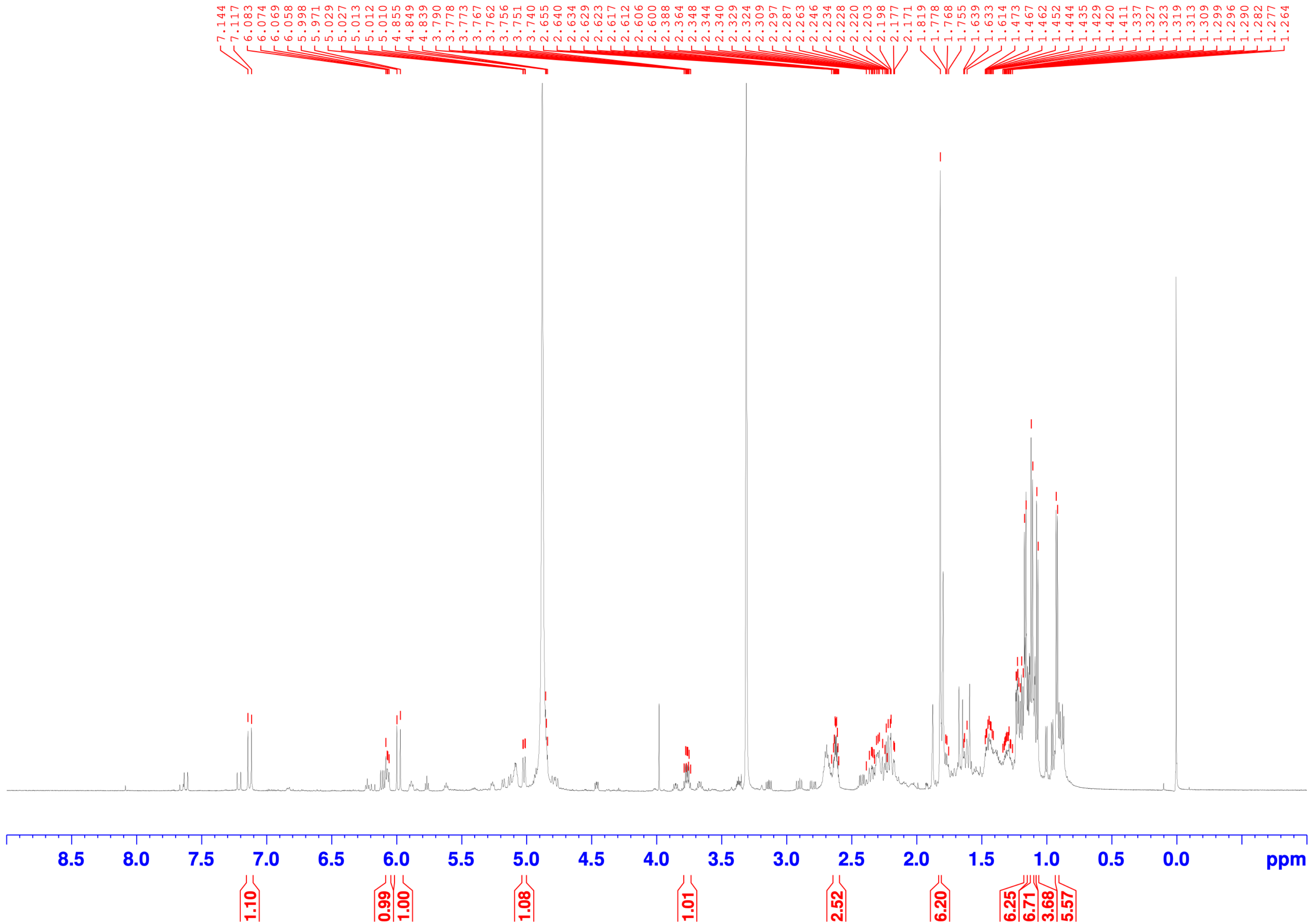

Figure S30. ^{1}H NMR spectrum of **3′** in CD_3OD at 600 MHz.

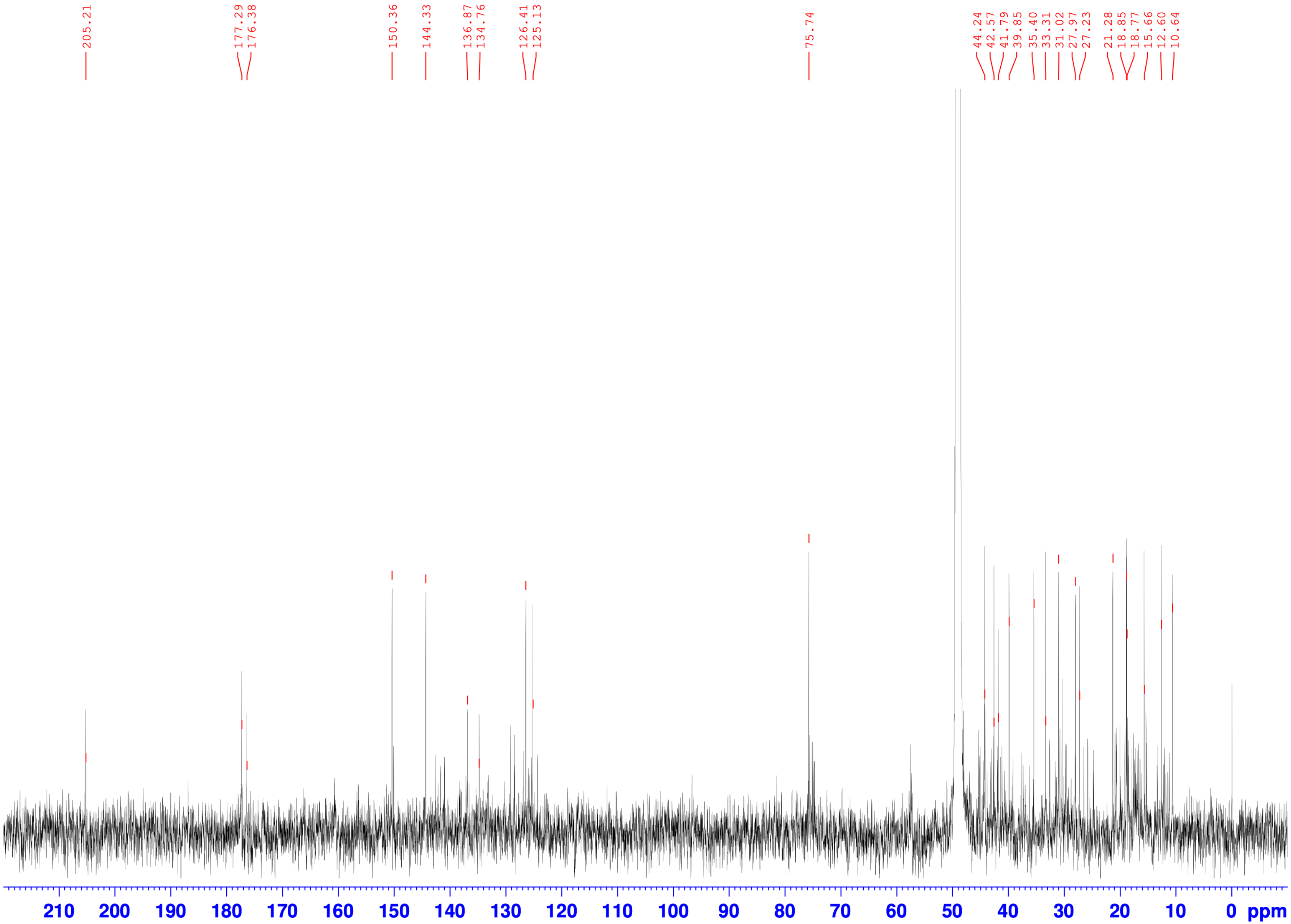

Figure S31. $^{13}C\{^{1}H\}$ NMR spectrum of **3′** in CD_3OD at 150 MHz.

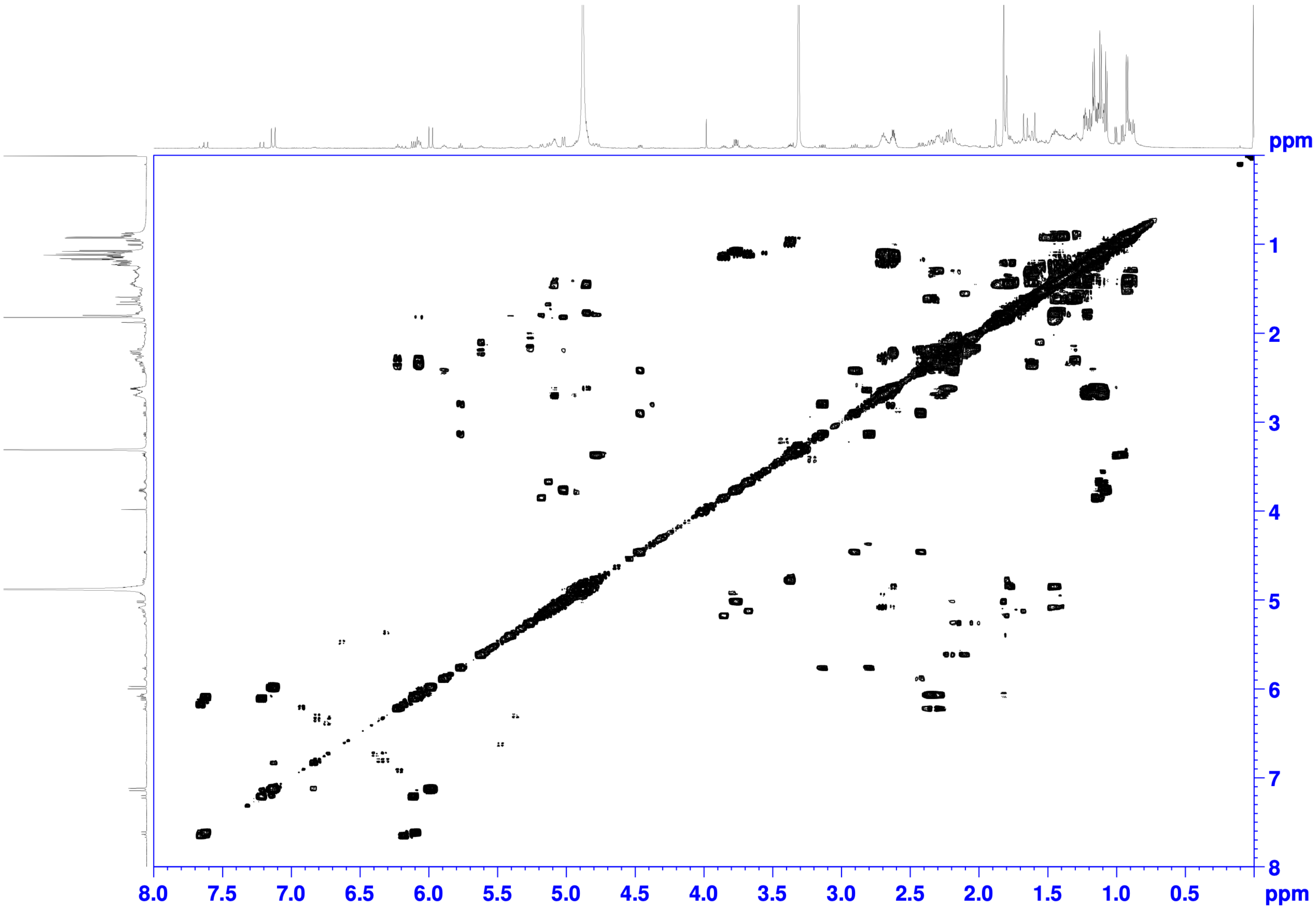

Figure S32. ^{1}H-^{1}H COSY spectrum of **3′** in CD_3OD.

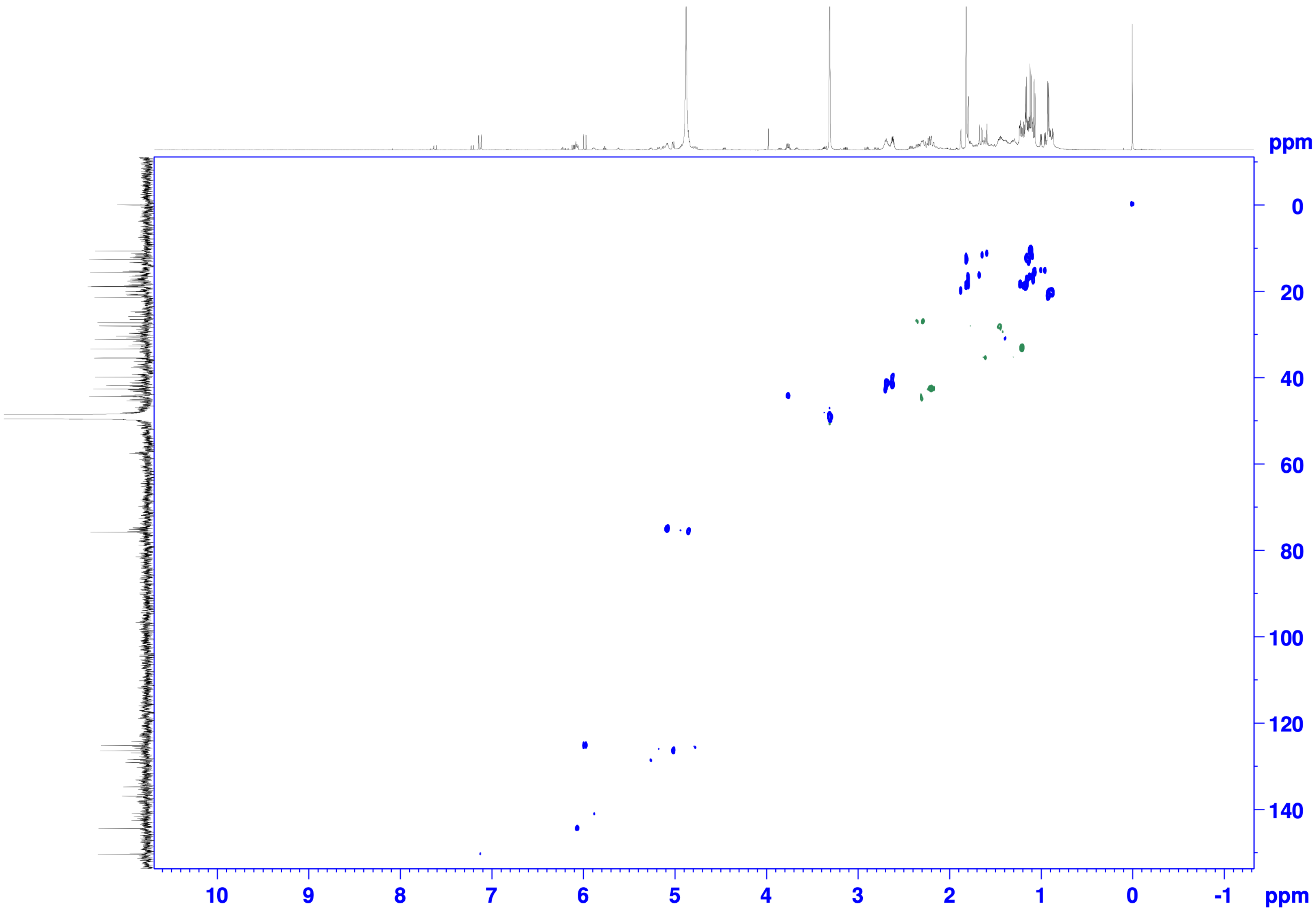

Figure S33. HSQC spectrum of **3′** in CD_3OD.

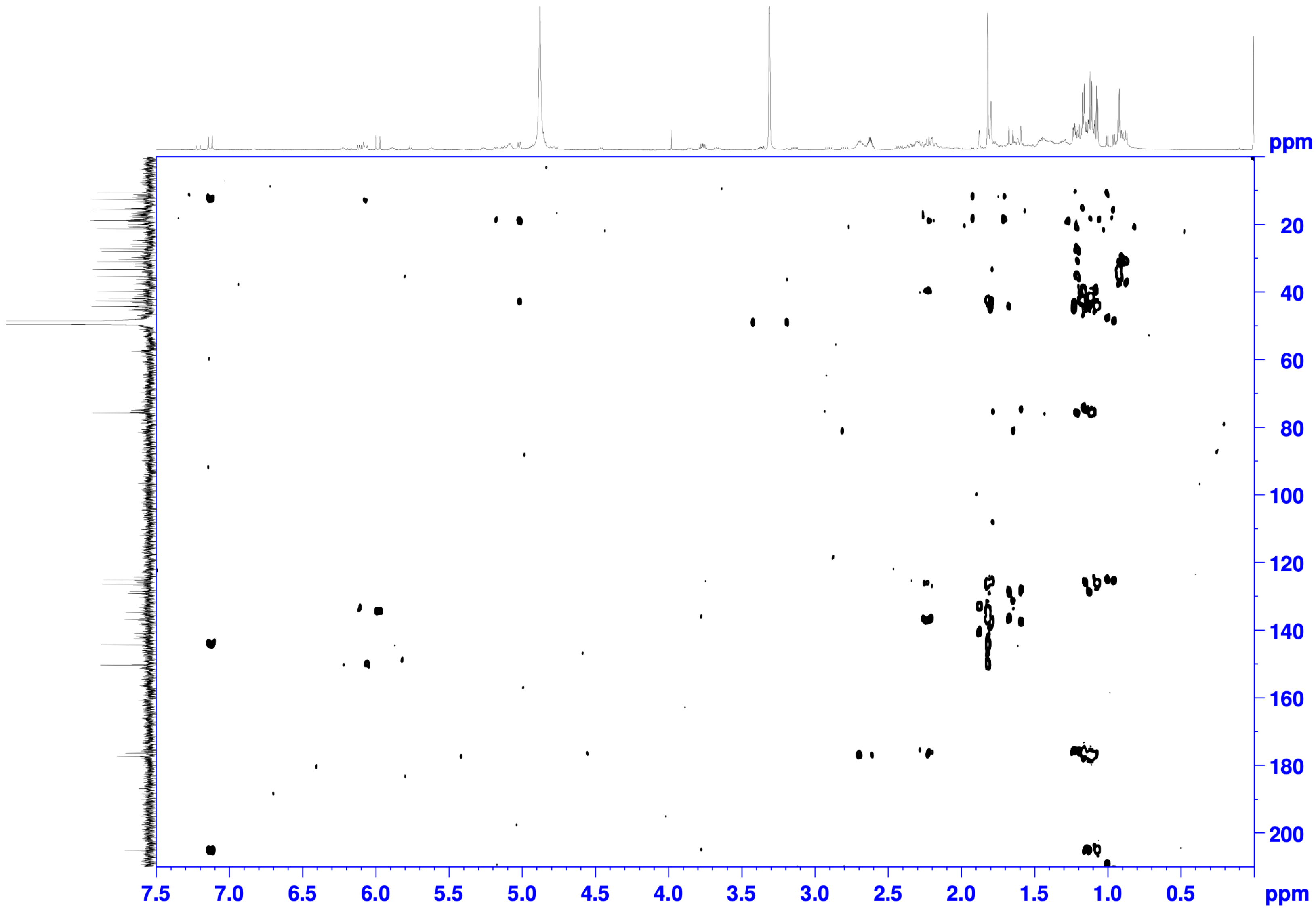

Figure S34. HMBC spectrum of **3′** in CD_3OD.

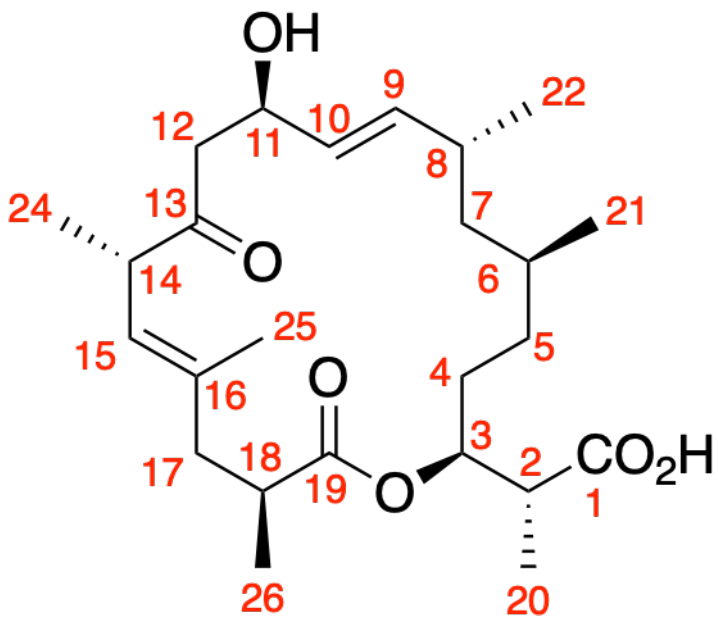

4

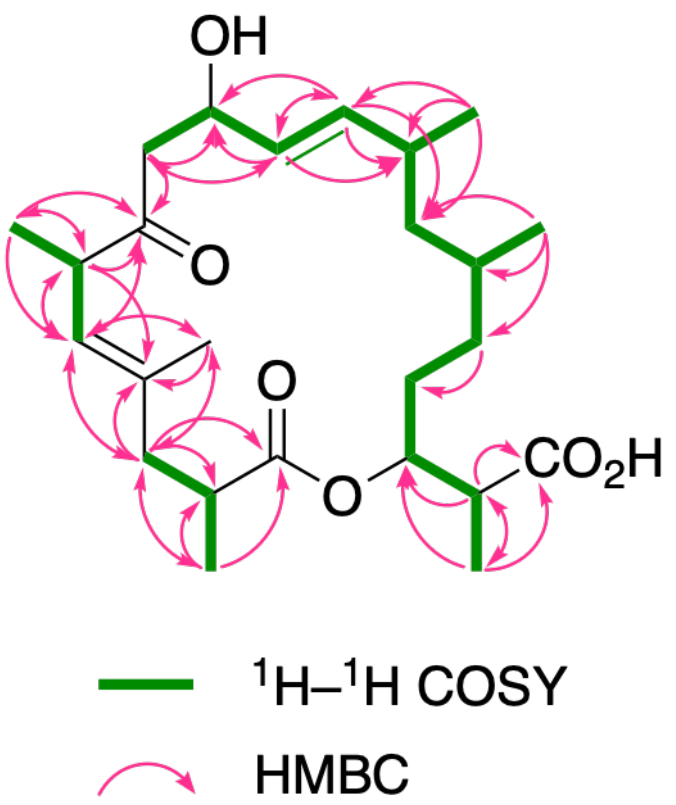

position	δ_C, type	δ_H, mult. (J in Hz)
1	177.4, C	
2	43.3, CH	2.74, dq (9.2, 6.9)
3	75.0, CH	5.12, dt (9.2, 3.9)
4	30.2, CH_2	1.73, tt (13.5, 4.2)
		1.49, tt (13.5, 3.9)
5	29.2, CH_2	1.64, tt (12.5, 3.2)
		1.03, m
6	31.1, CH	1.21, m
7	47.6, CH_2	1.22, m
		1.20, m
8	35.8, CH	2.23, m
9	141.5, CH	5.28, dd (15.2, 8.8)
10	132.4, CH	5.20, dd (15.2, 8.7)
11	71.7, CH	4.33, ddd (10.3, 8.8, 5.1)
12	48.4, CH_2	2.85, dd (14.6, 10.4)
		2.45, dd (14.6, 5.1)
13	210.9, C	
14	48.7, CH	3.38, dq (10.5, 6.6)
15	126.7, CH	4.84, brd (10.5)
16	138.3, C	
17	45.6, CH_2	2.38, t (12.6)
		2.25, dd (13.5, 2.9)
18	42.1, CH	2.69, dqd (11.8, 6.9, 3.3)
19	175.5, C	
20	14.7, CH_3	1.13, d (6.9)
21	21.3, CH_3	0.83, d (6.0)
22	23.6, CH_3	0.98, d (6.8)
24	14.9, CH_3	0.92, d (6.6)
25	16.4, CH_3	1.82, d (0.9)
26	18.5, CH_3	1.26, d (6.9)

^{1}H NMR: 600 MHz, ^{13}C NMR: 150 MHz (in CD_3OD)

Figure S35. NMR data of **4**.

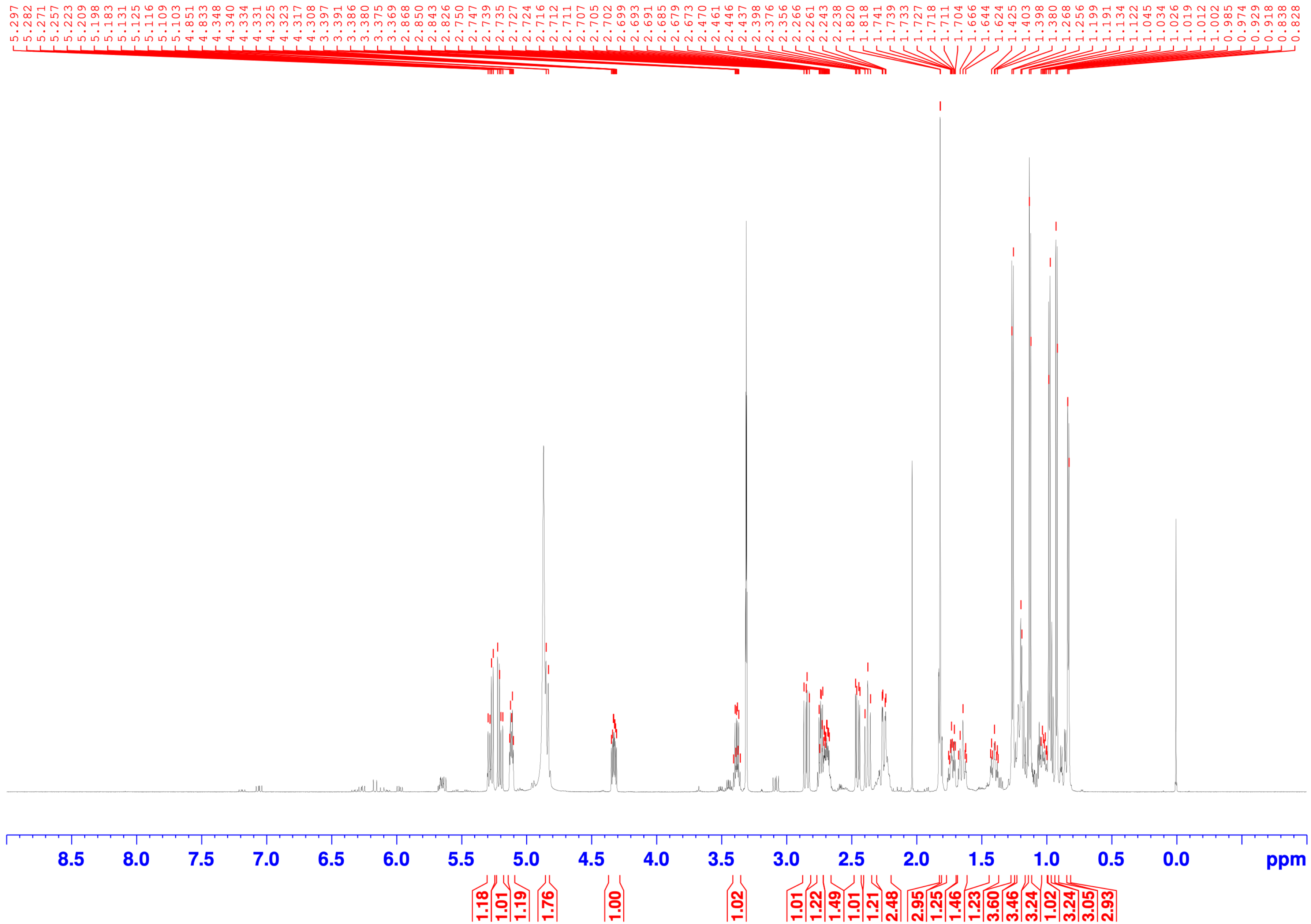

Figure S36. ^{1}H NMR spectrum of **4** in CD_3OD at 600 MHz.

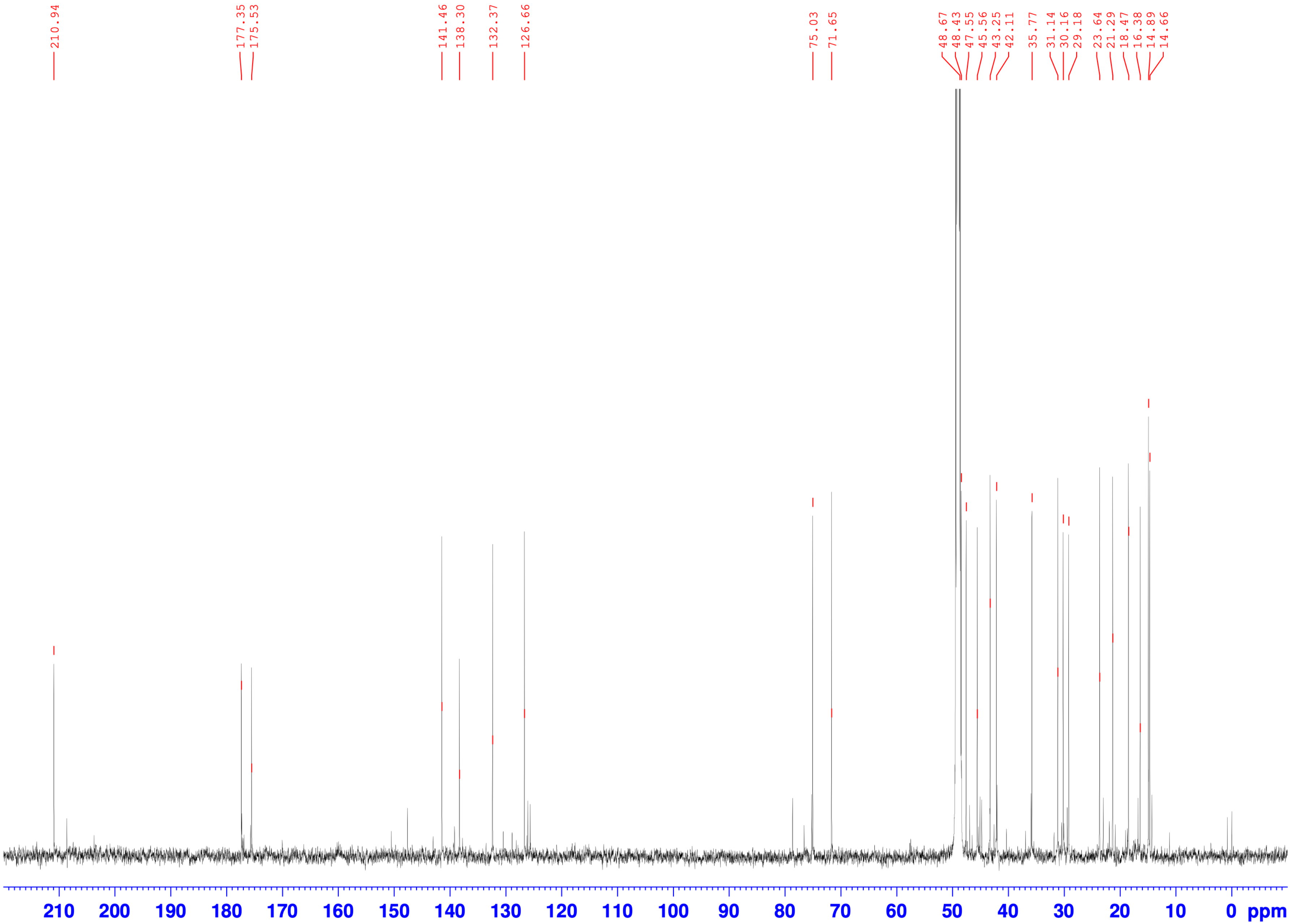

Figure S37. $^{13}C\{^{1}H\}$ NMR spectrum of **4** in CD_3OD at 150 MHz.

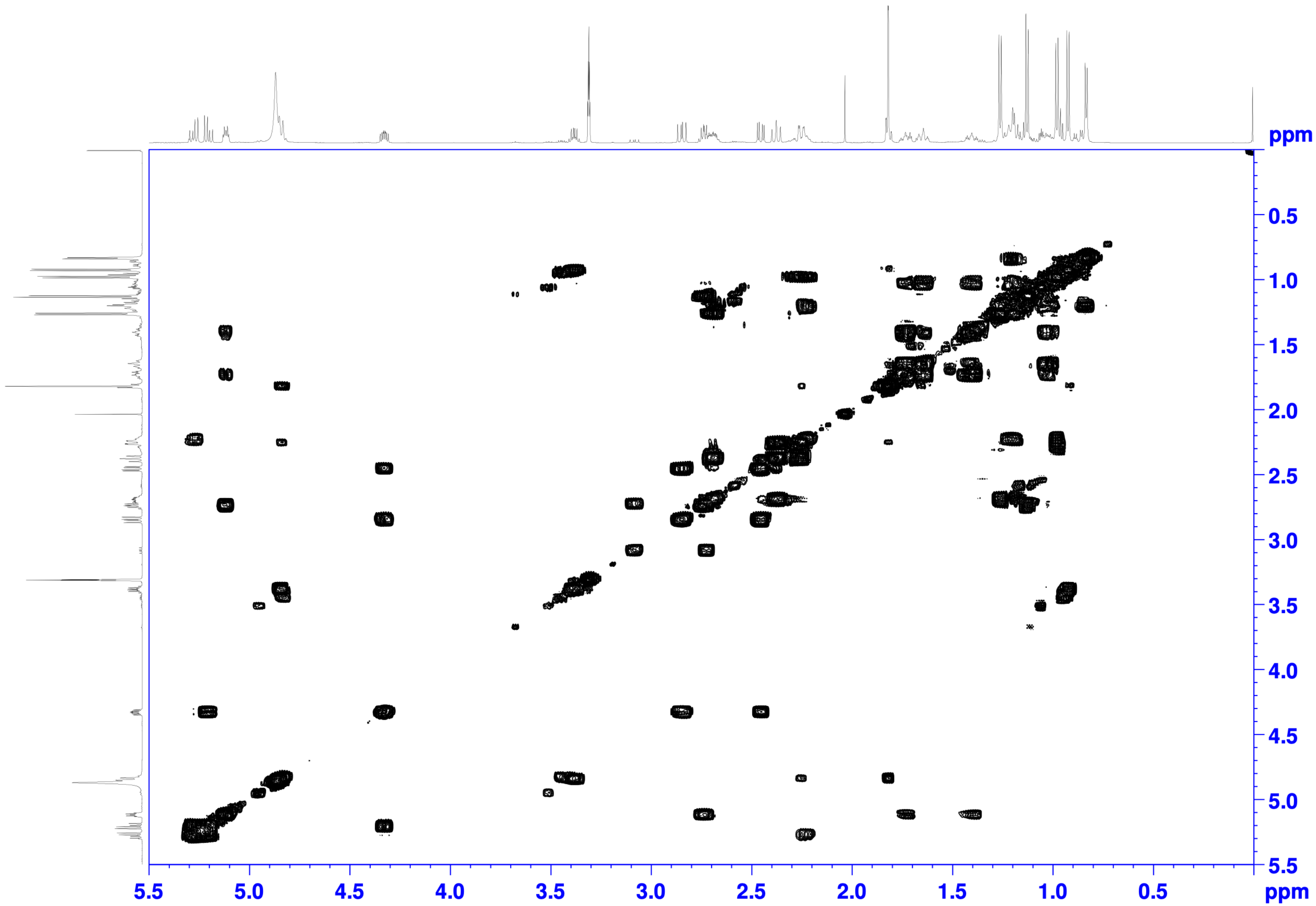

Figure S38. ^{1}H-^{1}H COSY spectrum of **4** in CD_3OD.

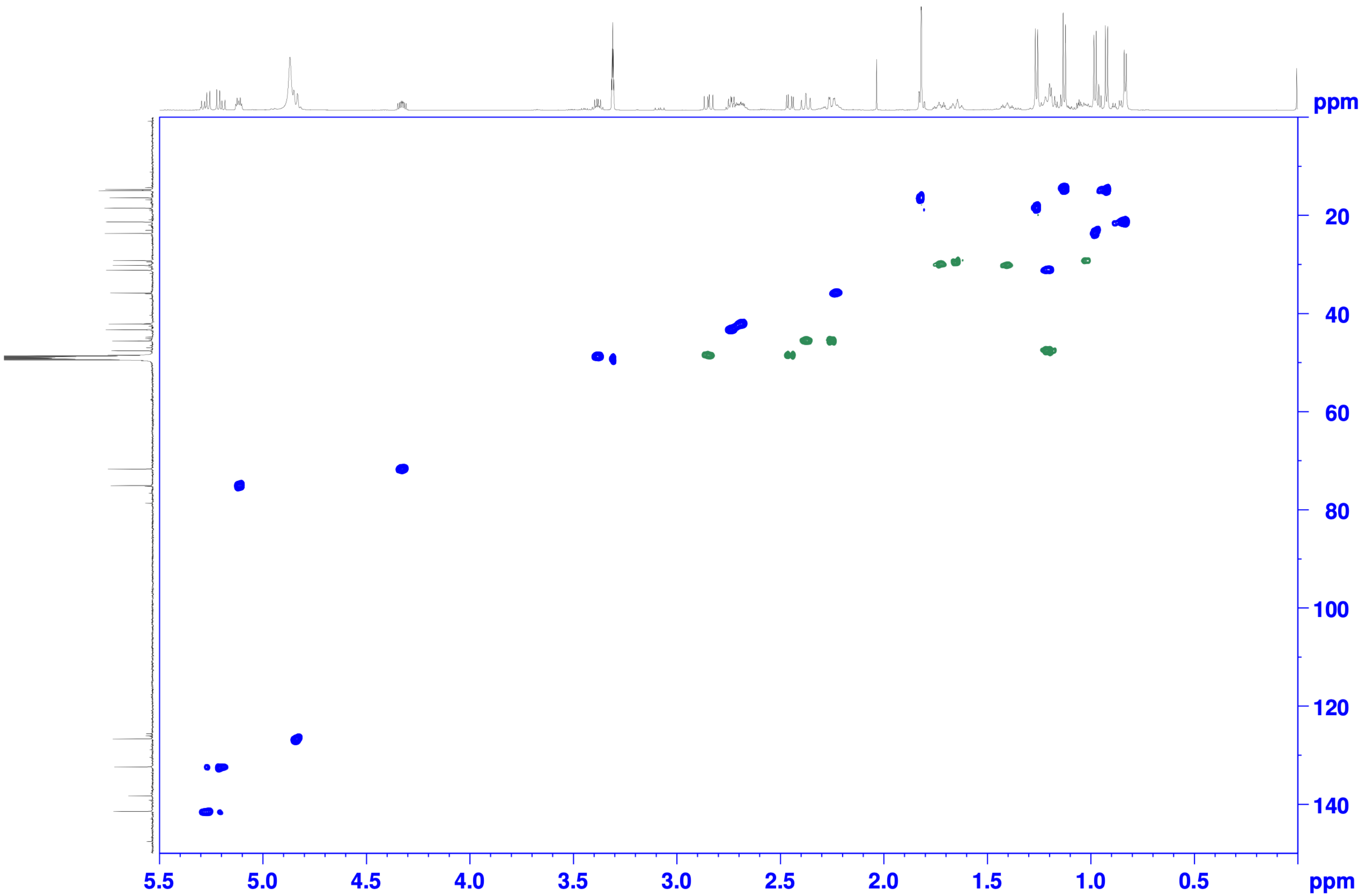

Figure S39. HSQC spectrum of **4** in CD_3OD.

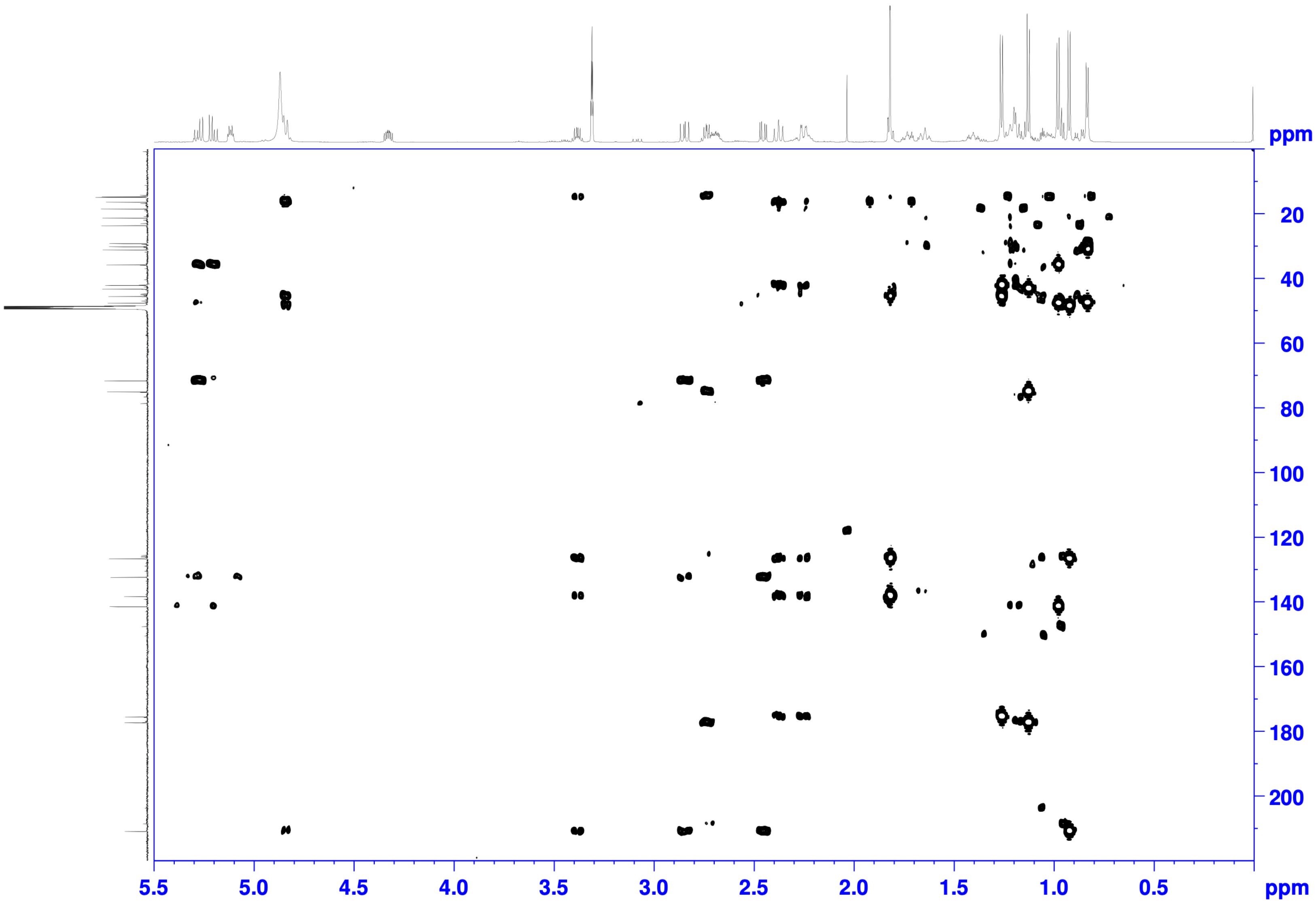

Figure S40. HMBC spectrum of **4** in CD_3OD.

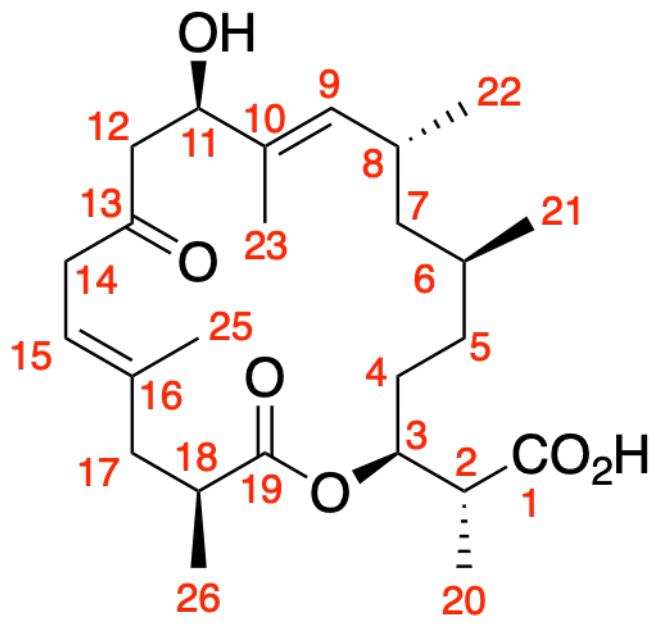

5

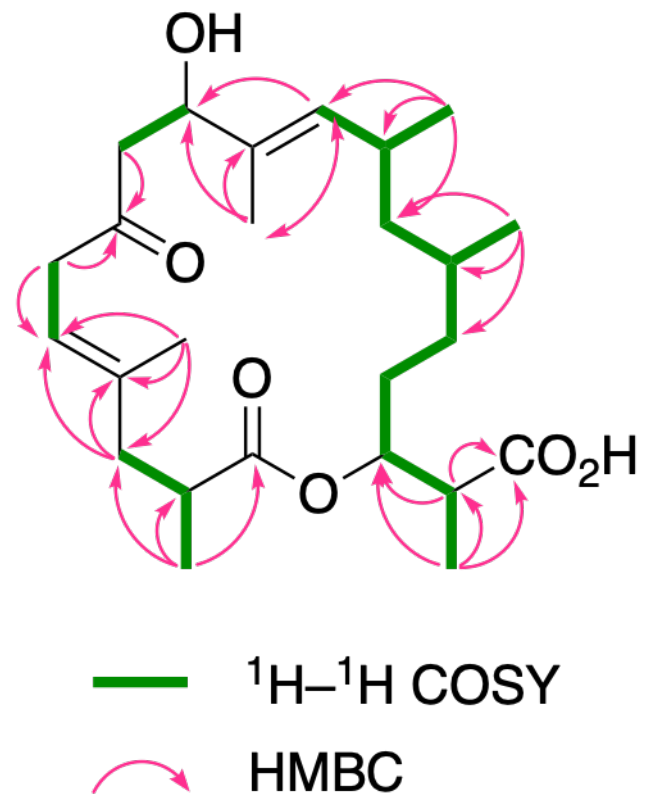

position	δC, type	δH, mult. (*J* in Hz)
1	177.4, C	
2	43.1, CH	2.70, m
3	75.2, CH	5.11, m
4	30.2, CH_2	1.66, m
		1.38, m
5	30.6, CH_2	1.44, m
		1.05, m
6	30.7, CH	1.37, m
7	46.9, CH_2	1.17, m
8	30.1, CH	2.48, m
9	136.5, CH	5.11, brd (10.4)
10	135.6, C	
11	76.1, CH	4.43, dd (10.2, 5.1)
12	47.3, CH_2	2.87, dd (14.3, 10.1)
		2.49, dd (14.3, 5.1)
13	209.1, C	
14	44.8, CH_2	3.24, dd (16.9, 9.0)
		2.87, dd (17.0, 3.5)
15	119.1, CH	5.09, m
16	138.4, C	
17	45.8, CH_2	2.34, dd (13.6, 11.7)
		2.23, brd (13.6)
18	41.5, CH	2.70, m
19	176.1, C	
20	13.4, CH_3	1.14, d (7.0)
21	20.9, CH_3	0.86, d (6.6)
22	21.5, CH_3	0.93, d (6.7)
23	10.2, CH_3	1.62, d (1.1)
25	16.4, CH_3	1.70, brs
26	18.6, CH_3	1.22, d (6.9)

^{1}H NMR: 600 MHz, ^{13}C NMR: 150 MHz (in CD_3OD)

Figure S41. NMR data of **5**.

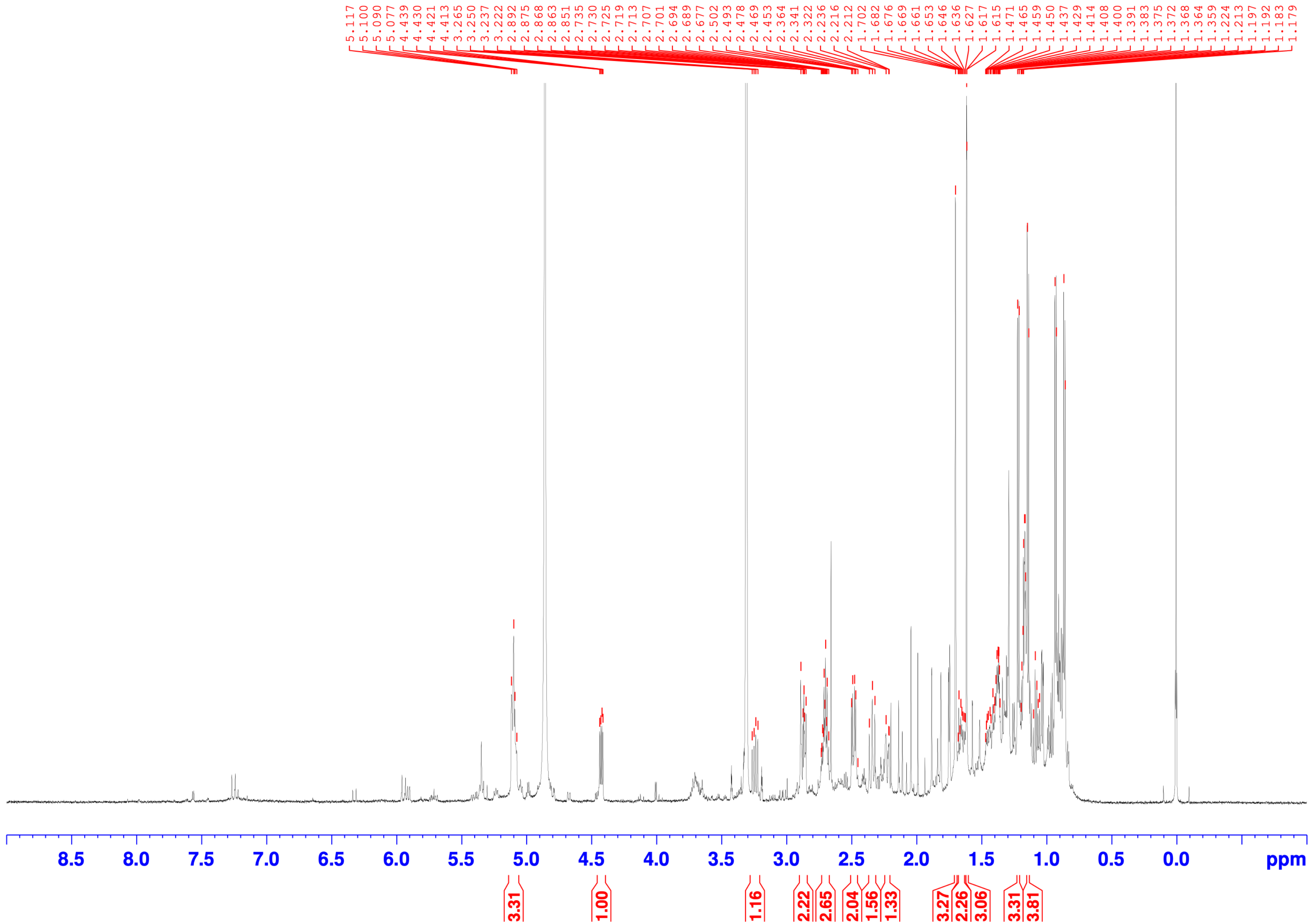

Figure S42. ^{1}H NMR spectrum of **5** in CD_3OD at 600 MHz.

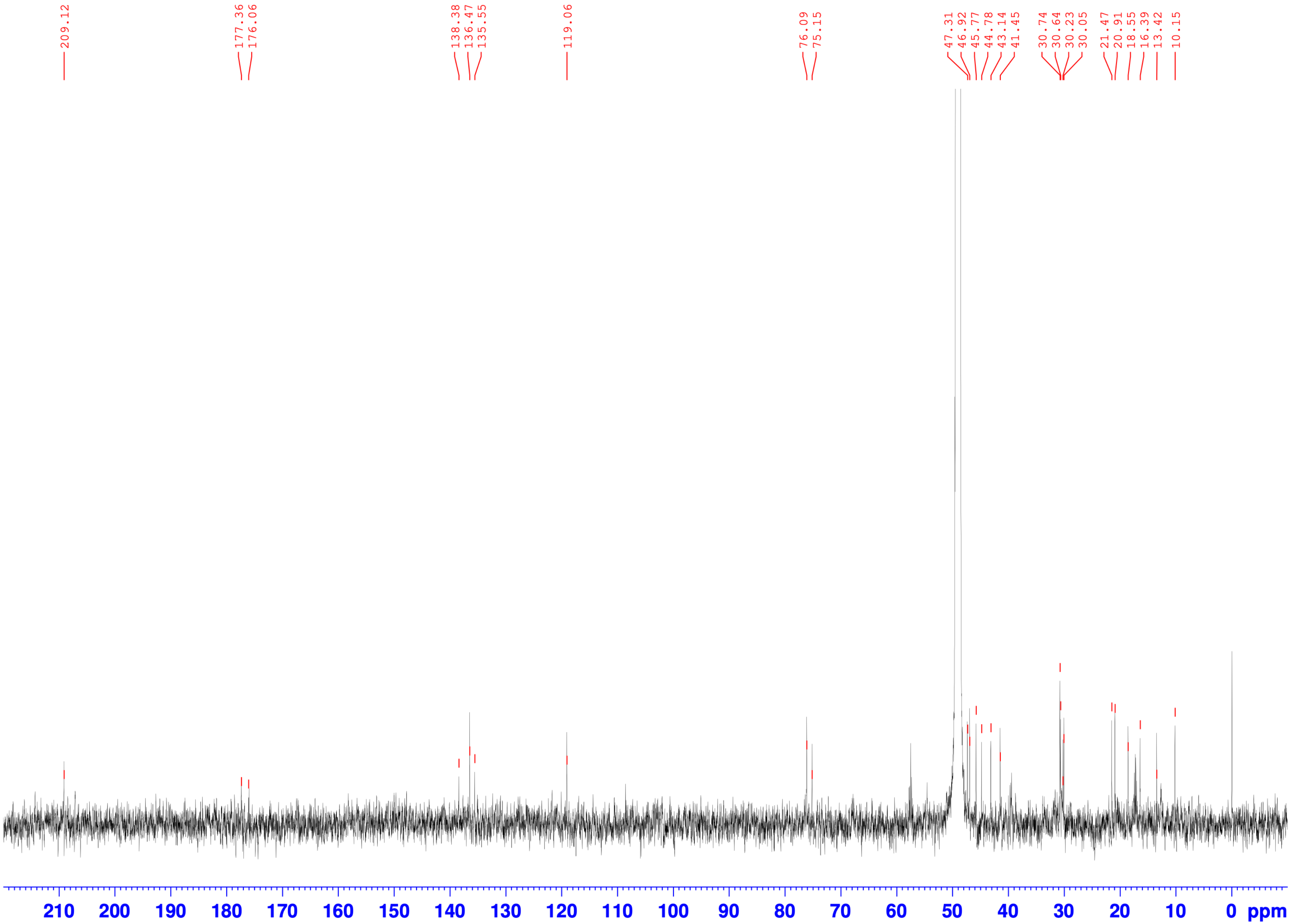

Figure S43. $^{13}C\{^1H\}$ NMR spectrum of **5** in CD_3OD at 150 MHz.

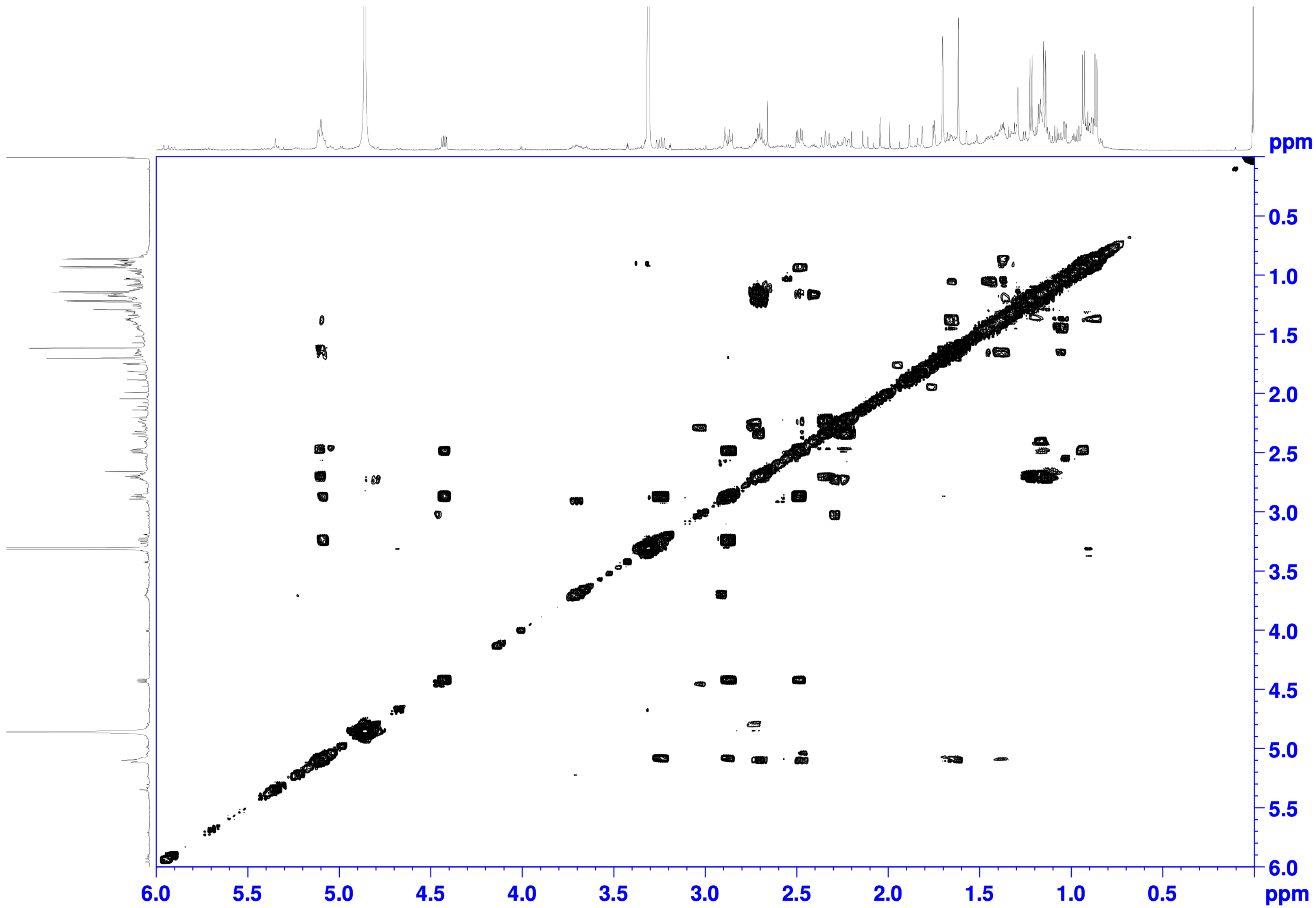

Figure S44. ^{1}H-^{1}H COSY spectrum of **5** in CD_3OD.

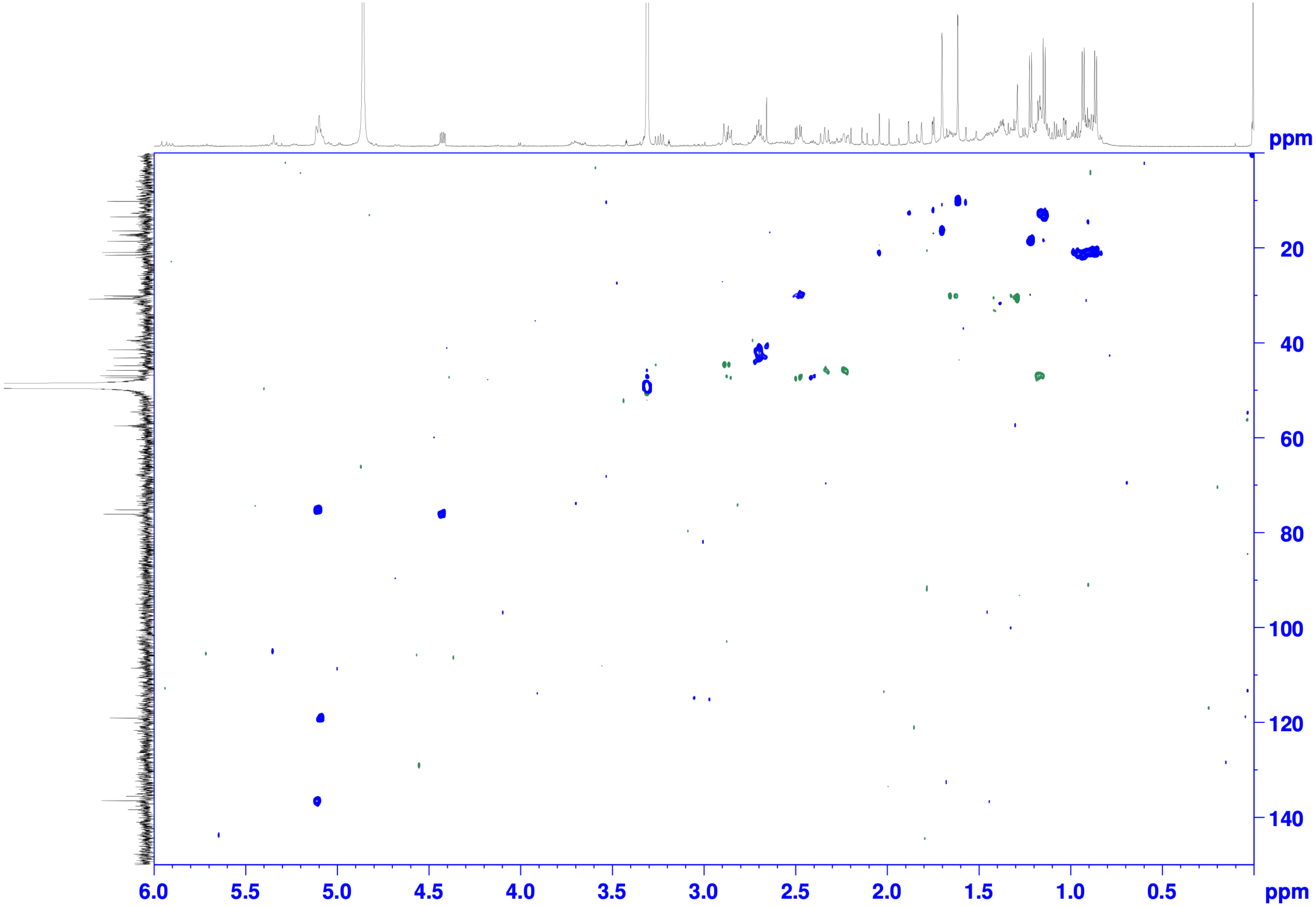

Figure S45. HSQC spectrum of **5** in CD_3OD.

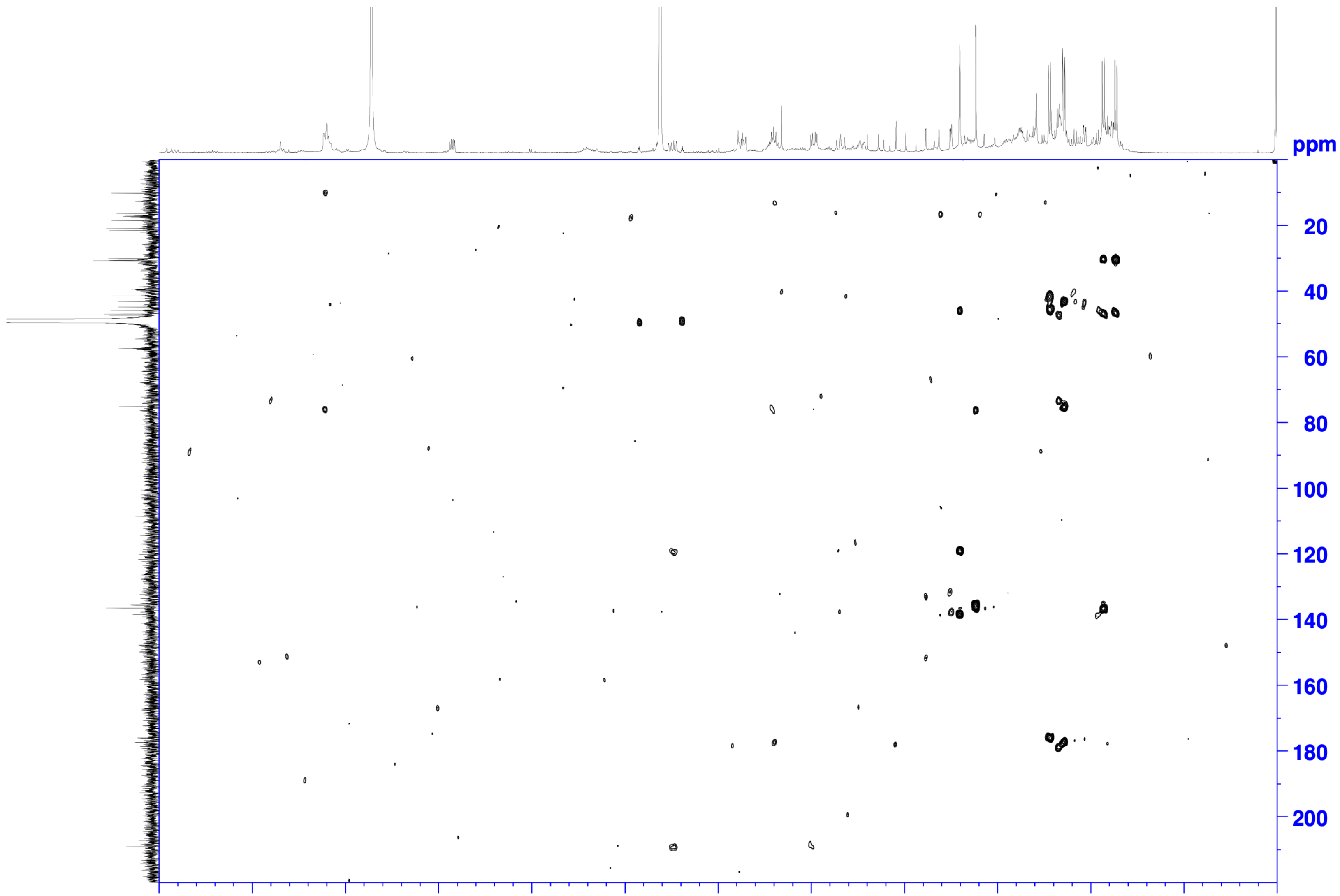

Figure S46. HMBC spectrum of **5** in CD_3OD.

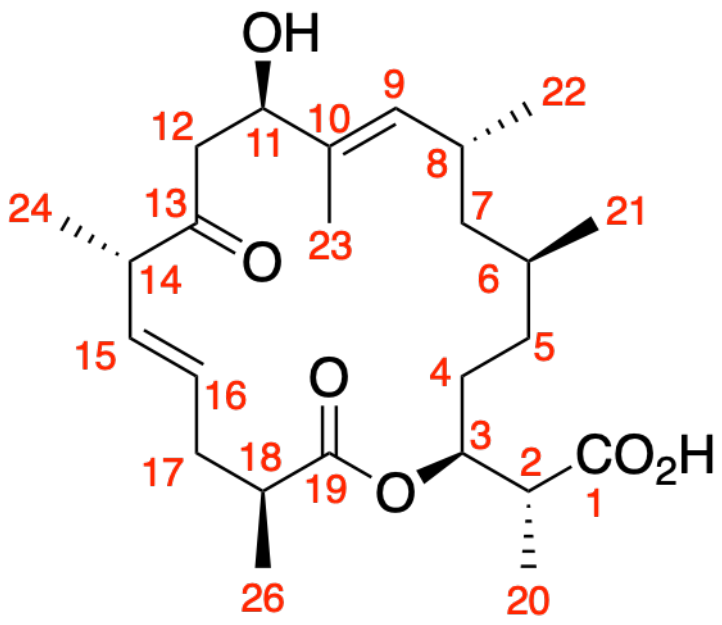

6

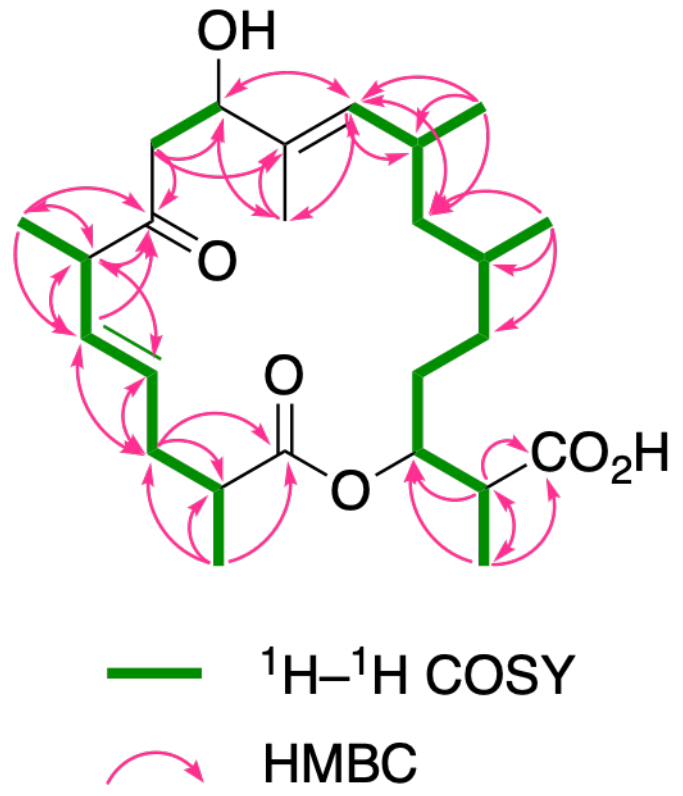

position	δ_C, type	δ_H, mult. (J in Hz)
1	177.3, C	
2	43.2, CH	2.71, dq (8.4, 6.9)
3	75.6, CH	5.09, dt (8.4, 4.2)
4	30.2, CH_2	1.70, m
		1.45, ddt (14.2, 11.0, 4.9)
5	29.9, CH_2	1.63, m
		0.98, m
6	31.0, CH	1.32, m
7	48.1, CH_2	1.24, m
		1.19, m
8	30.3, CH	2.48, m
9	135.7, CH	5.08, brd (8.4)
10	136.7, C	
11	75.7, CH	4.45, dd (8.8, 5.7)
12	46.6, CH_2	3.02, dd (15.8, 8.9)
		2.44, dd (15.8, 5.7)
13	210.5, C	
14	52.7, CH	3.15, dq (9.5, 6.7)
15	132.9, CH	5.17, dd (15.3, 9.6)
16	132.6, CH	5.70, dt (15.3, 7.6)
17	38.3, CH_2	2.29, m
		2.23, m
18	43.1, CH	2.50, m
19	176.0, C	
20	14.2, CH_3	1.15, d (6.9)
21	21.4, CH_3	0.85, d (6.6)
22	22.1, CH_3	0.94, d (6.7)
23	10.5, CH_3	1.62, d (1.1)
24	15.9, CH_3	0.99, d (6.7)
26	18.0, CH_3	1.22, d (7.0)

^1H NMR: 600 MHz, ^{13}C NMR: 150 MHz (in CD_3OD)

Figure S47. NMR data of **6**.

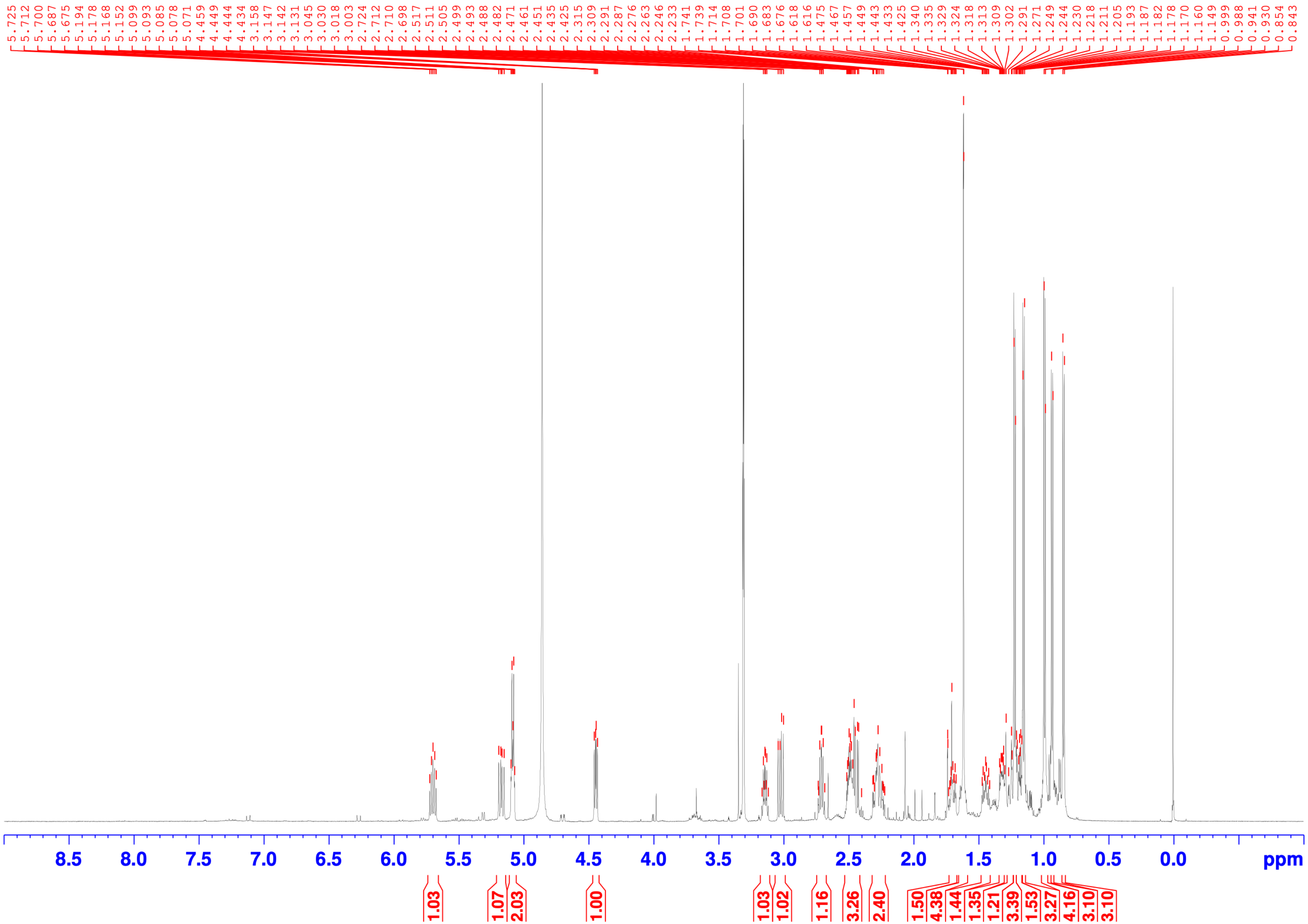

Figure S48. ^{1}H NMR spectrum of **6** in CD_3OD at 600 MHz.

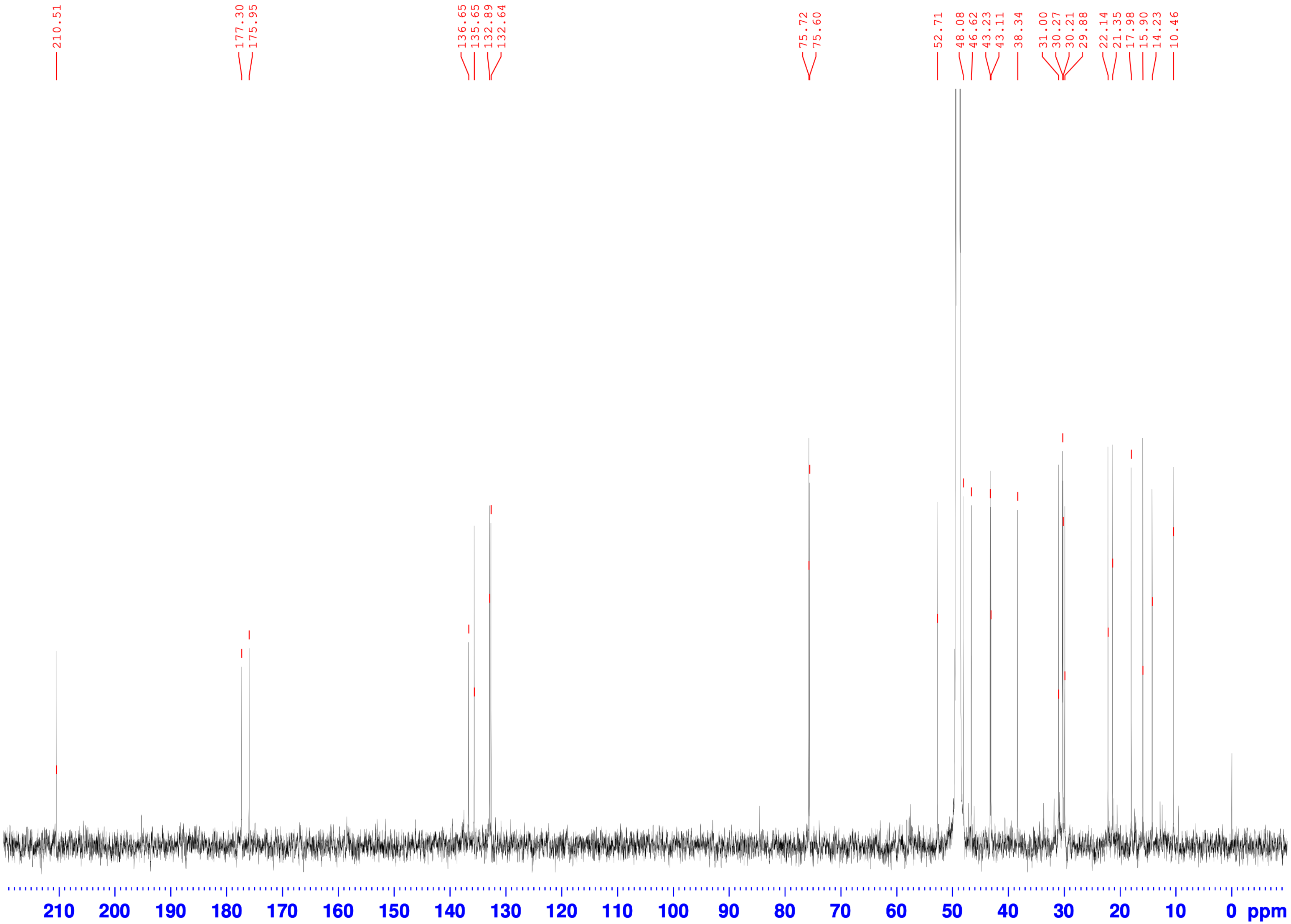

Figure S49. $^{13}C\{^{1}H\}$ NMR spectrum of **6** in CD_3OD at 150 MHz.

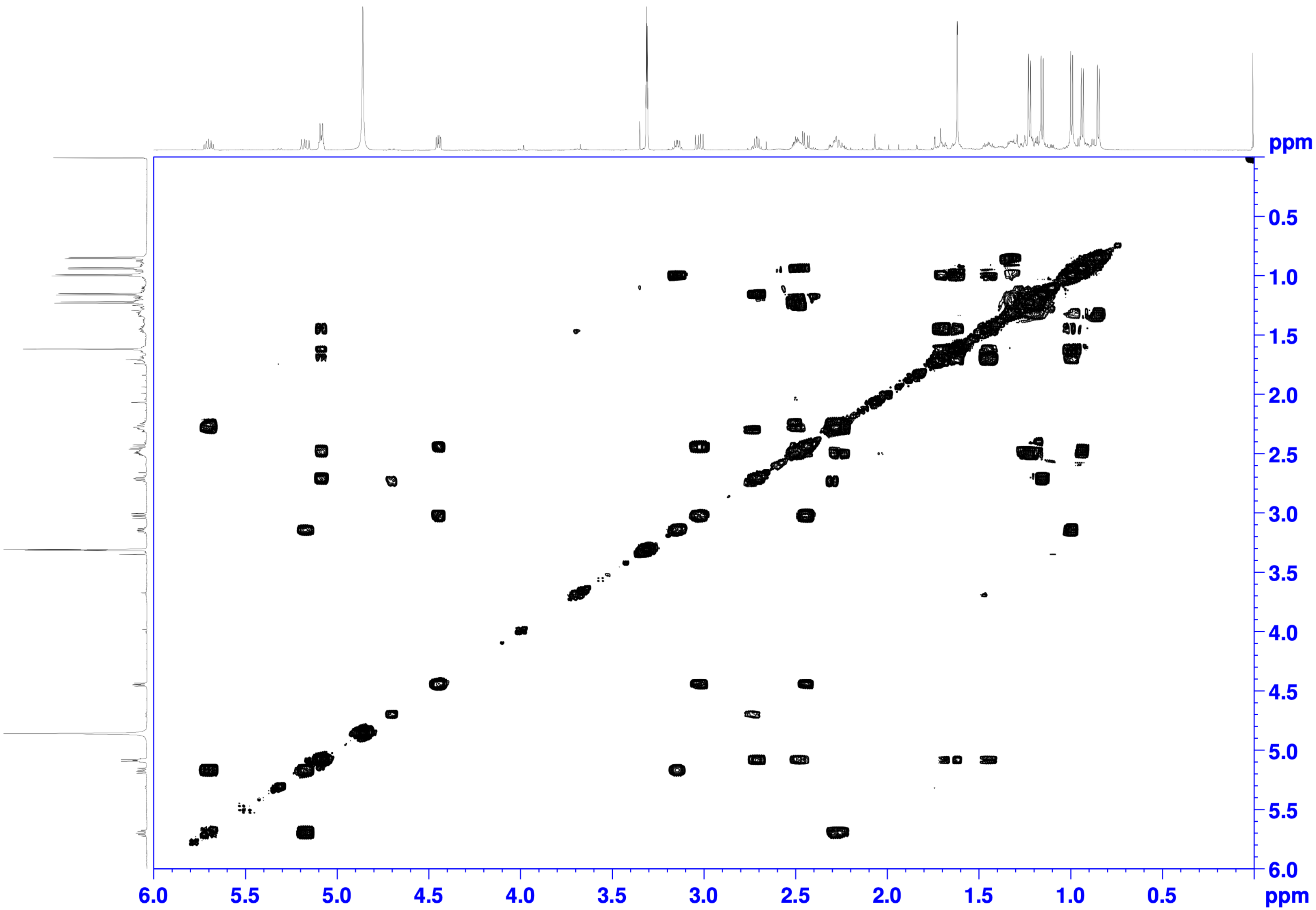

Figure S50. ^{1}H-^{1}H COSY spectrum of **6** in CD_3OD.

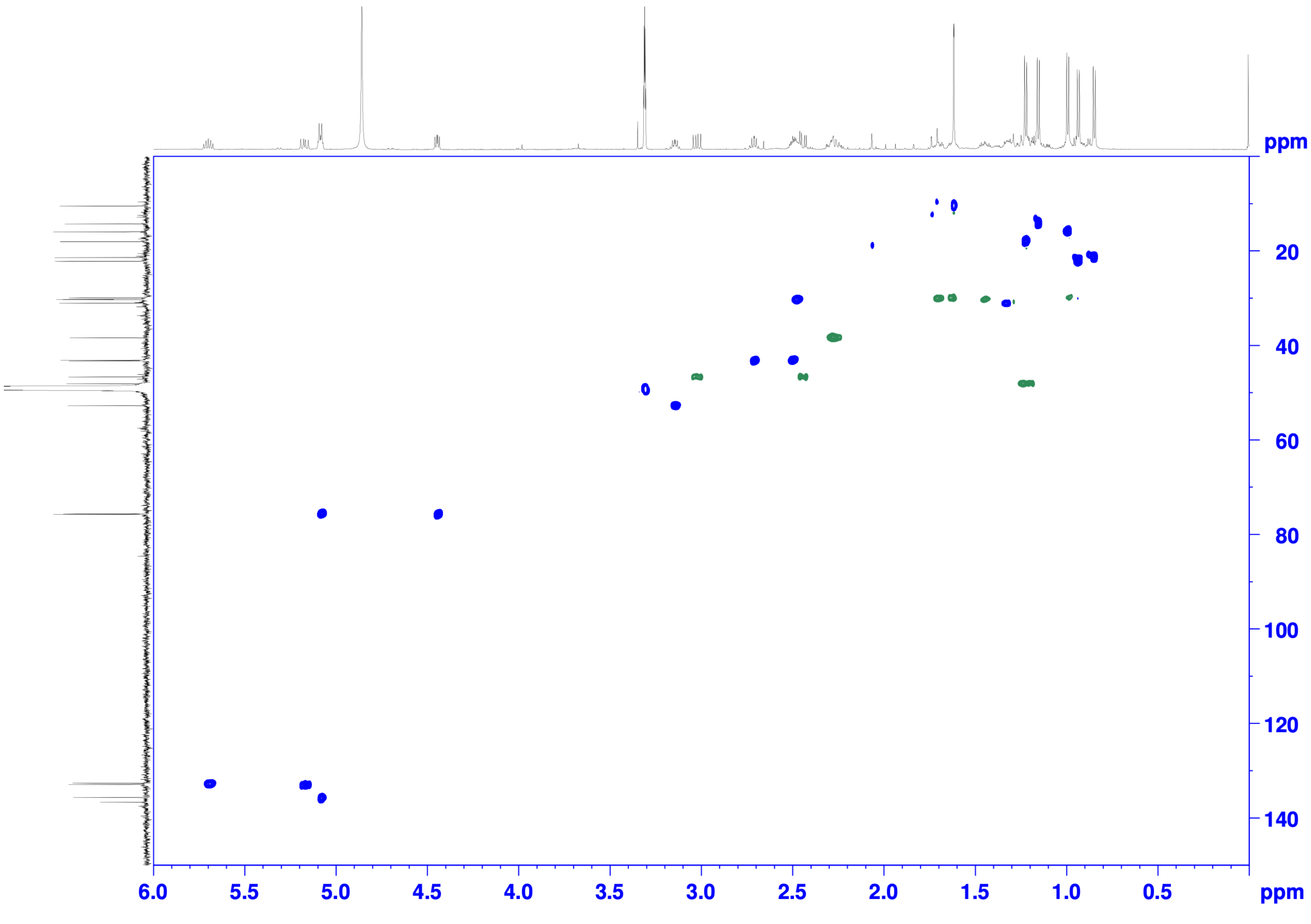

Figure S51. HSQC spectrum of **6** in CD_3OD.

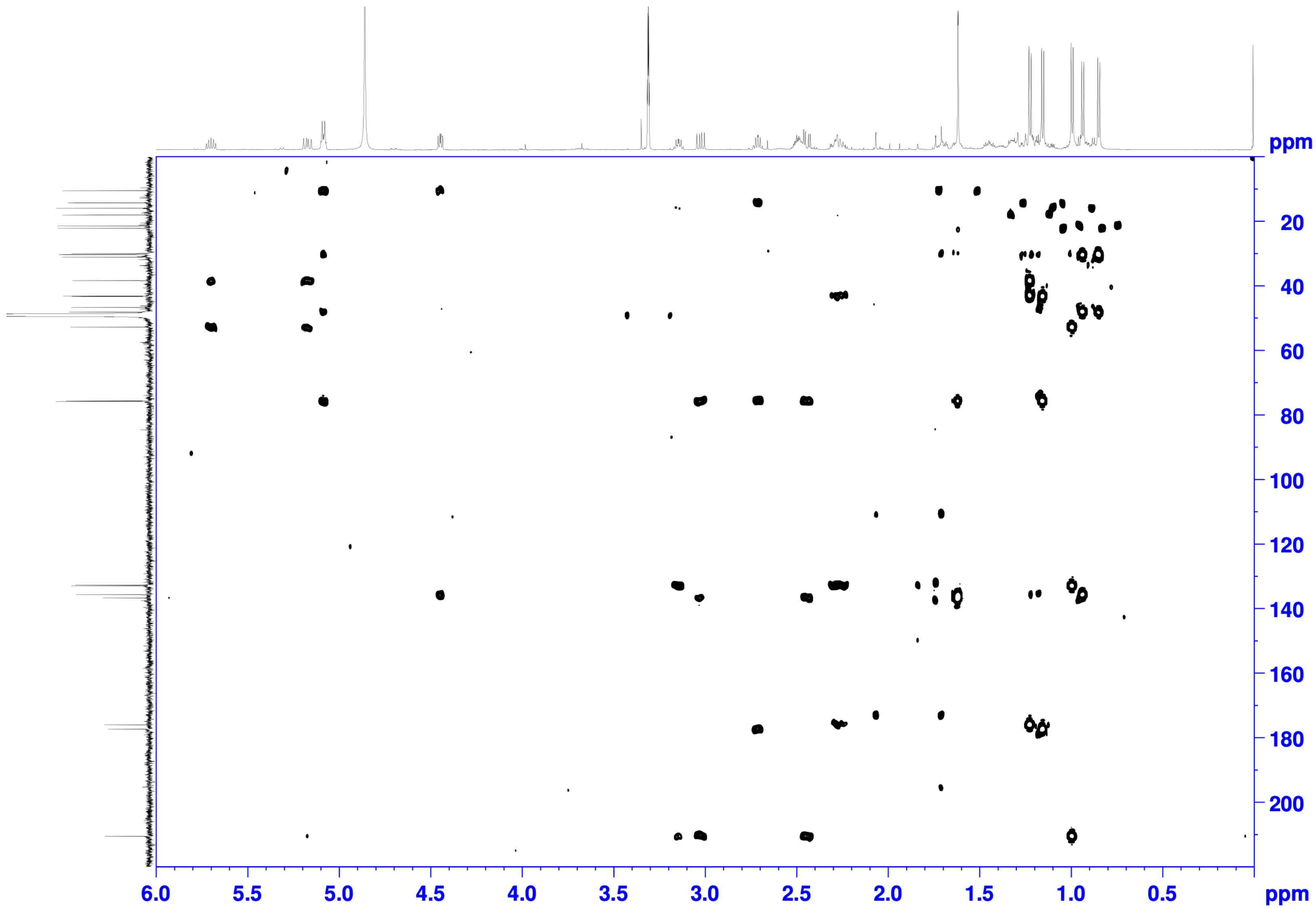

Figure S52. HMBC spectrum of **6** in CD_3OD.

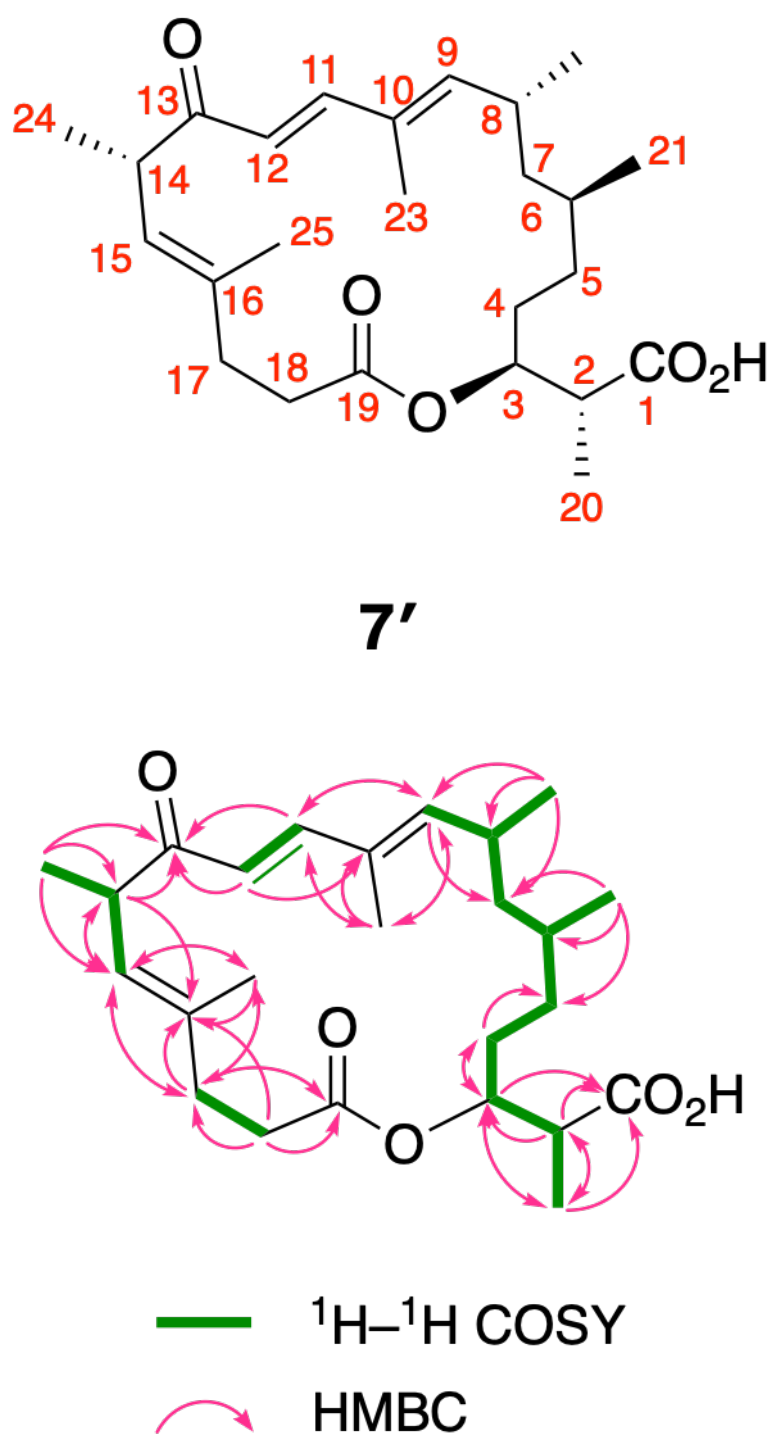

position	δ_C, type	δ_H, mult. (J in Hz)
1	177.4, C	
2	43.4, CH	2.61, m
3	76.0, CH	4.83, q (5.3)
4	30.8, CH_2	1.58, m
		1.48, m
5	30.9, CH_2	1.48, m
		0.93, m
6	31.7, CH	1.43, m
7	46.6, CH_2	1.42, m
8	31.4, CH	2.78, m
9	149.3, CH	5.71, d (10.0)
10	133.0, C	
11	146.8, CH	7.04, d (15.5)
12	125.0, CH	6.17, d (15.5)
13	203.1, C	
14	47.5, CH	3.53, dq (10.5, 6.5)
15	127.8, CH	4.93, brd (10.5)
16	138.4, C	
17	35.2, CH_2	2.45, ddd (13.4, 9.3, 4.4)
		2.15, m
18	33.1, CH_2	2.54, ddd (17.1, 7.1, 4.6)
		2.26, ddd (17.0, 9.4, 4.5)
19	173.7, C	
20	12.5, CH_3	1.10, d (7.0)
21	21.0, CH_3	0.87, d (6.0)
22	21.7, CH_3	1.00, d (6.7)
23	12.8, CH_3	1.83, brs
24	14.6, CH_3	1.08, d (6.5)
25	16.5, CH_3	1.84, brs

^{1}H NMR: 600 MHz, ^{13}C NMR: 150 MHz (in CD_3OD)

Figure S53. NMR data of **7′**.

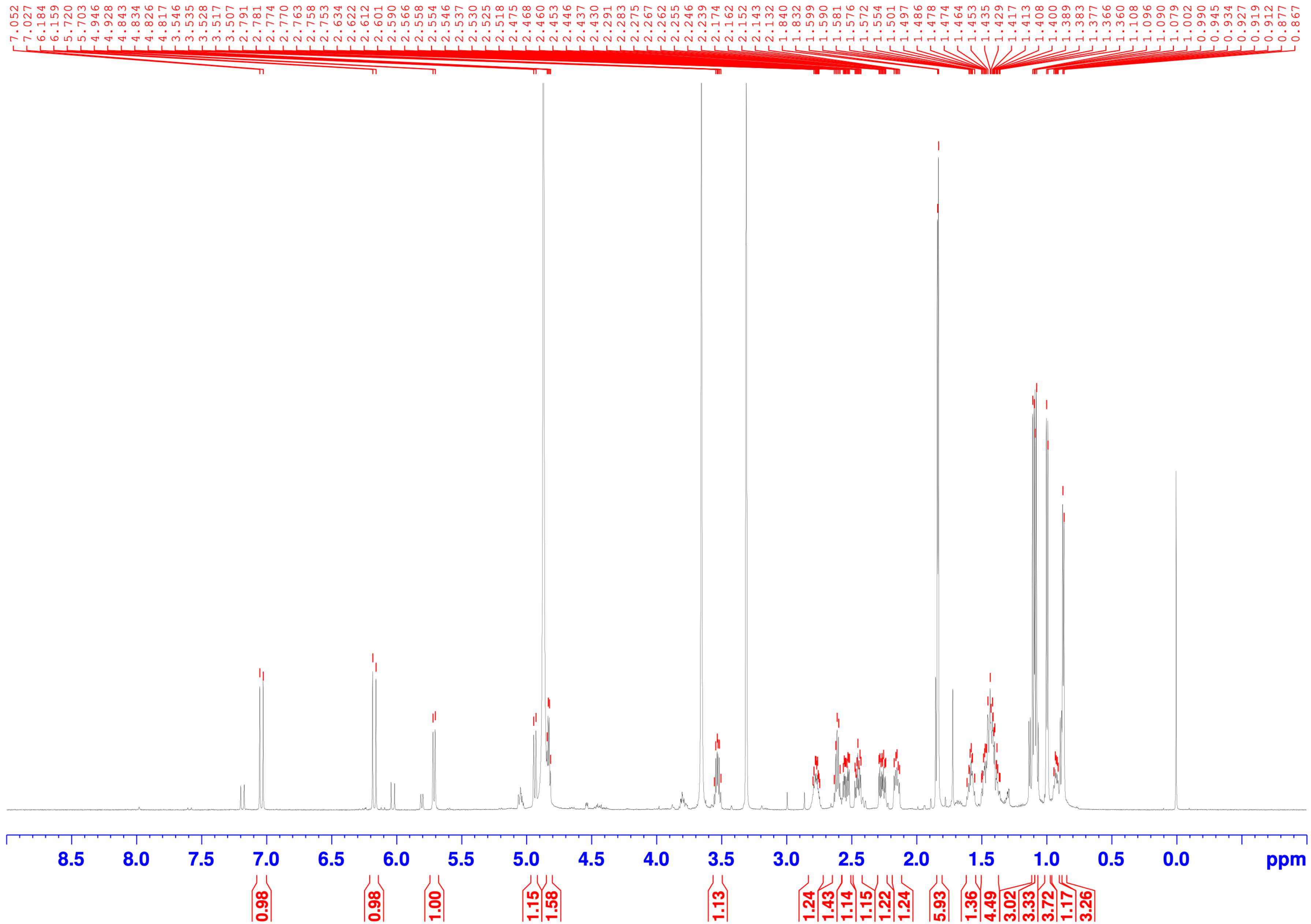

Figure S54. ^{1}H NMR spectrum of **7′** in CD_3OD at 600 MHz.

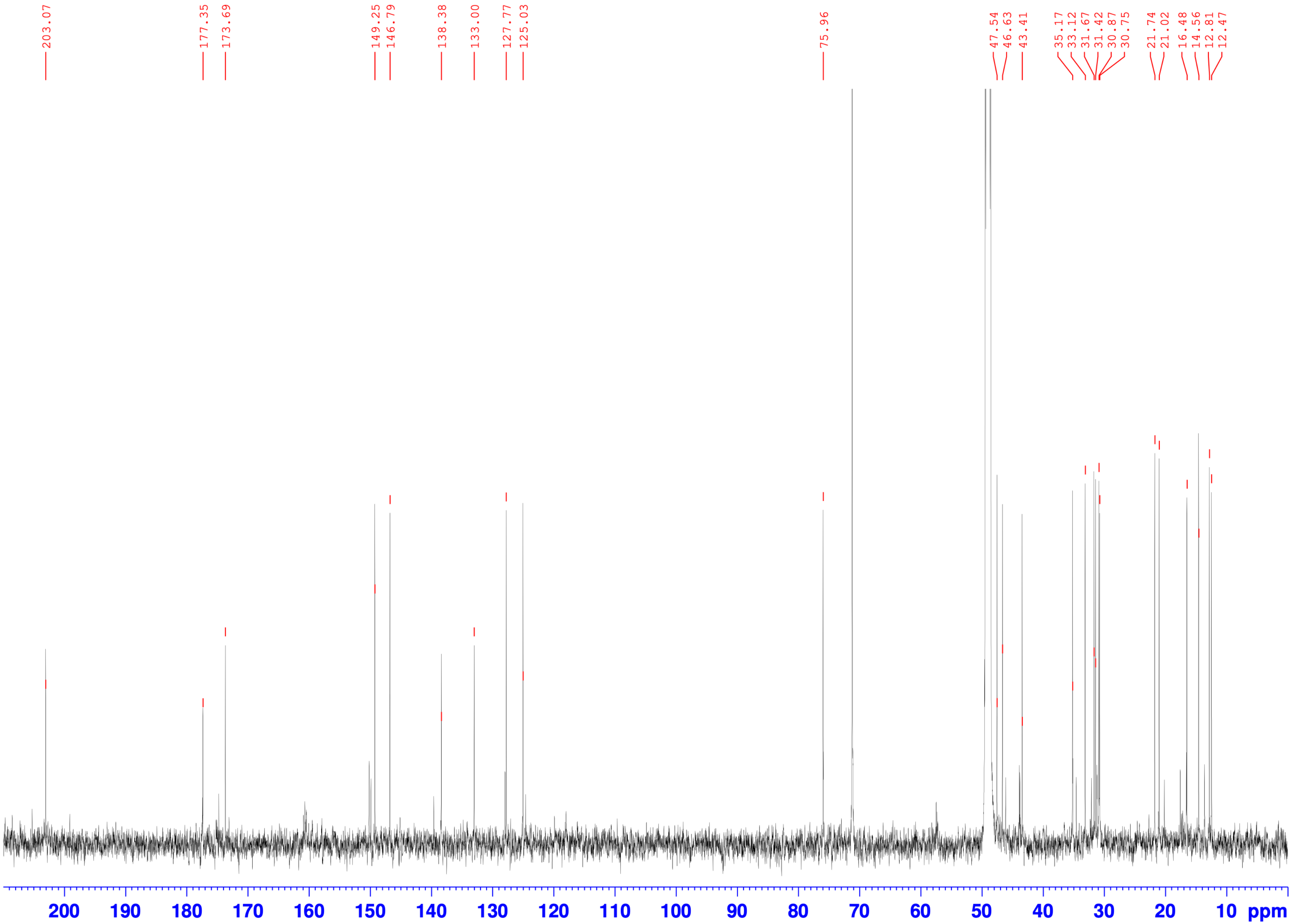

Figure S55. $^{13}C\{^1H\}$ NMR spectrum of **7′** in CD_3OD at 150 MHz.

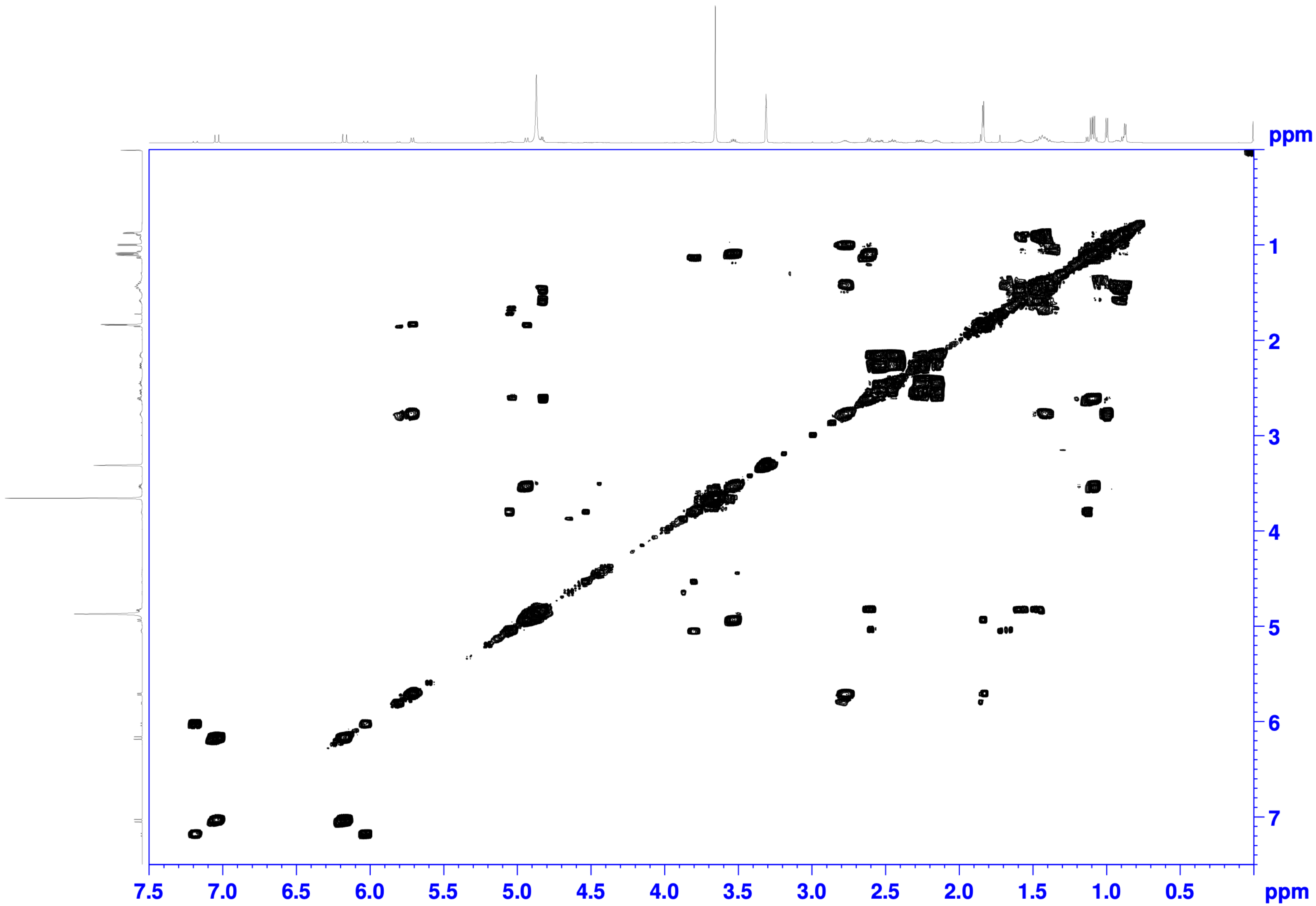

Figure S56. ^{1}H-^{1}H COSY spectrum of **7′** in CD_3OD.

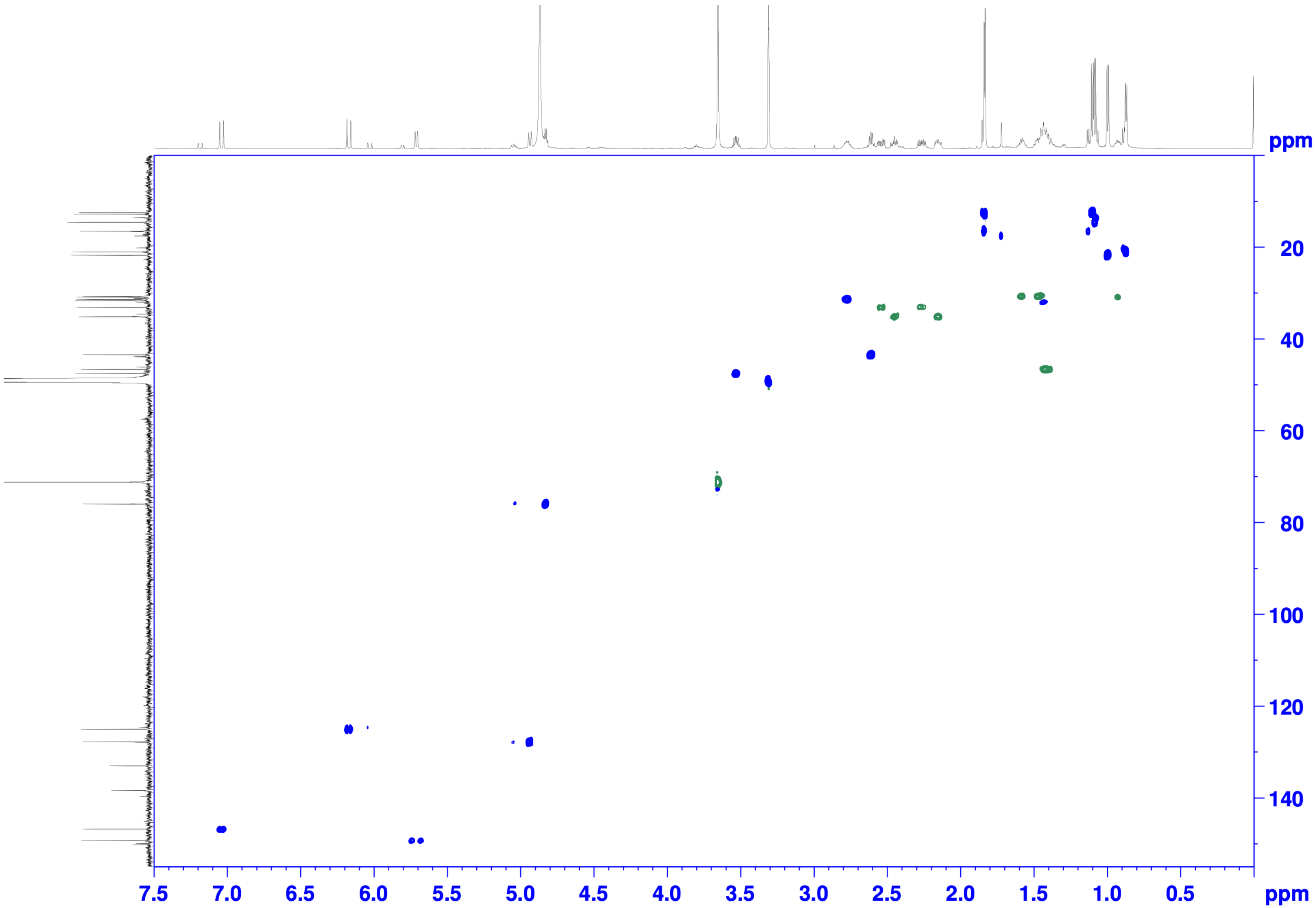

Figure S57. HSQC spectrum of **7′** in CD_3OD.

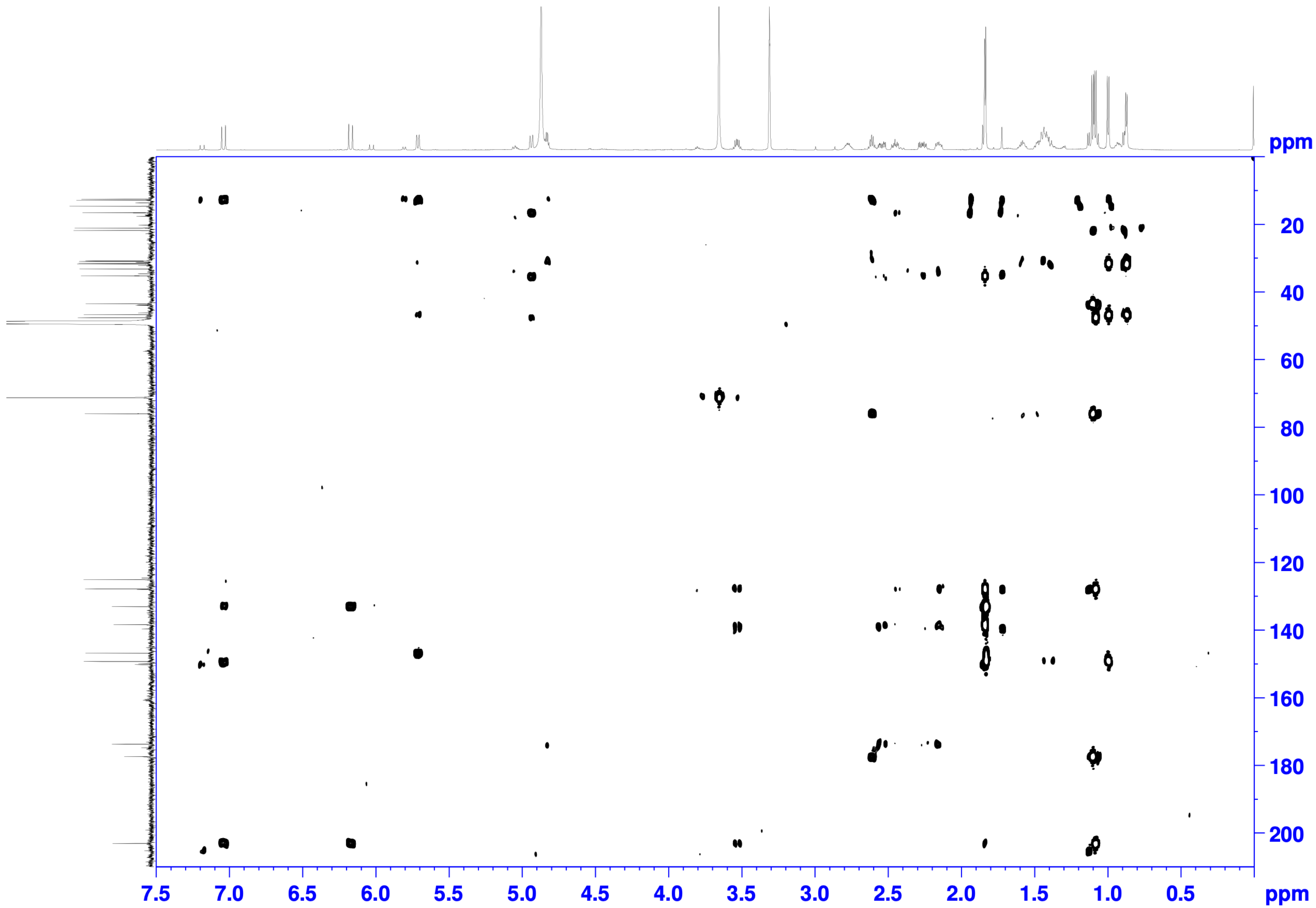

Figure S58. HMBC spectrum of **7′** in CD_3OD.

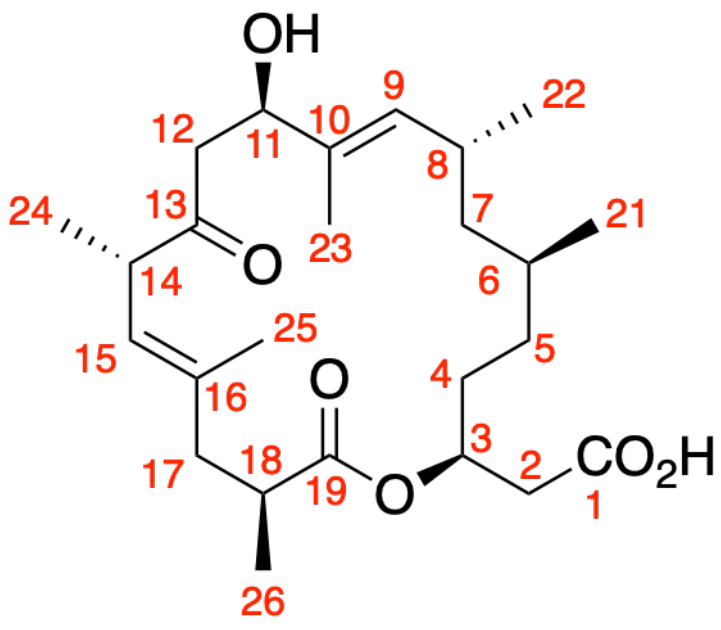

8

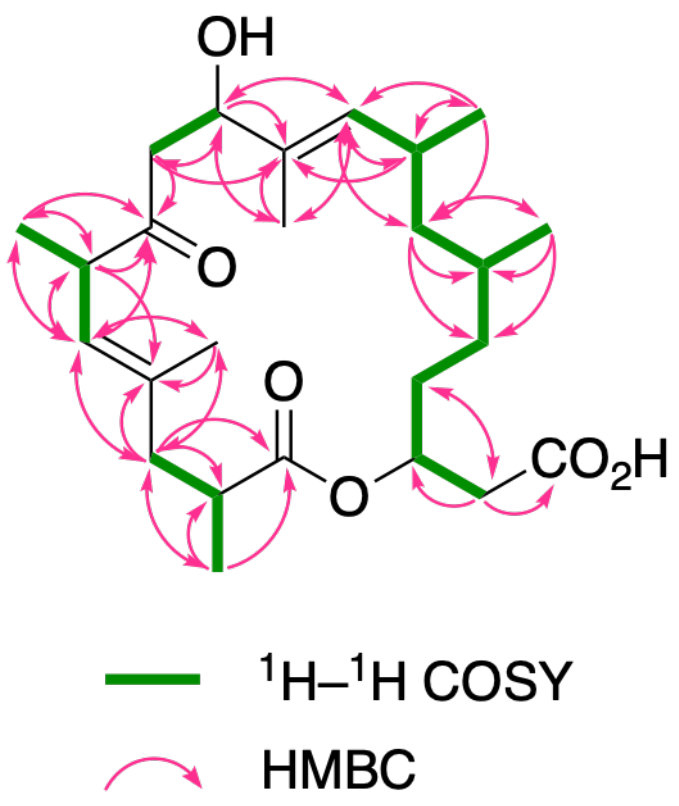

position	δ_C, type	δ_H, mult. (J in Hz)
1	174.1, C	
2	38.1, CH_2	2.61, dd (15.9, 9.6)
		2.48, dd (15.8, 4.4)
3	70.7, CH	5.44, m
4	31.7, CH_2	1.70, m
		1.45, m
5	28.9, CH_2	1.68, m
		0.87, m
6	31.3, CH	1.20, m
7	49.0, CH_2	1.25, brt (11.0)
		1.20, m
8	30.3, CH	2.46, m
9	136.2, CH	5.04, dd (8.5, 1.2)
10	136.5, C	
11	76.2, CH	4.46, dd (10.9, 4.5)
12	46.3, CH_2	3.04, dd (14.9, 11.0)
		2.30, dd (14.9, 4.7)
13	210.9, C	
14	48.9, CH	3.36, dq (10.5, 6.6)
15	126.4, CH	4.67, d (10.4)
16	138.5, C	
17	46.2, CH_2	2.29, t (12.9)
		2.17, dd (12.9, 3.0)
18	42.2, CH	2.59, m
19	175.3, C	
21	21.3, CH_3	0.86, d (5.9)
22	22.3, CH_3	0.93, d (6.8)
23	10.2, CH_3	1.58, d (1.2)
24	14.9, CH_3	0.91, d (6.6)
25	16.1, CH_3	1.80, d (1.1)
26	18.3, CH_3	1.17, d (6.9)

^{1}H NMR: 600 MHz, ^{13}C NMR: 150 MHz (in CD_3OD)

Figure S59. NMR data of **8**.

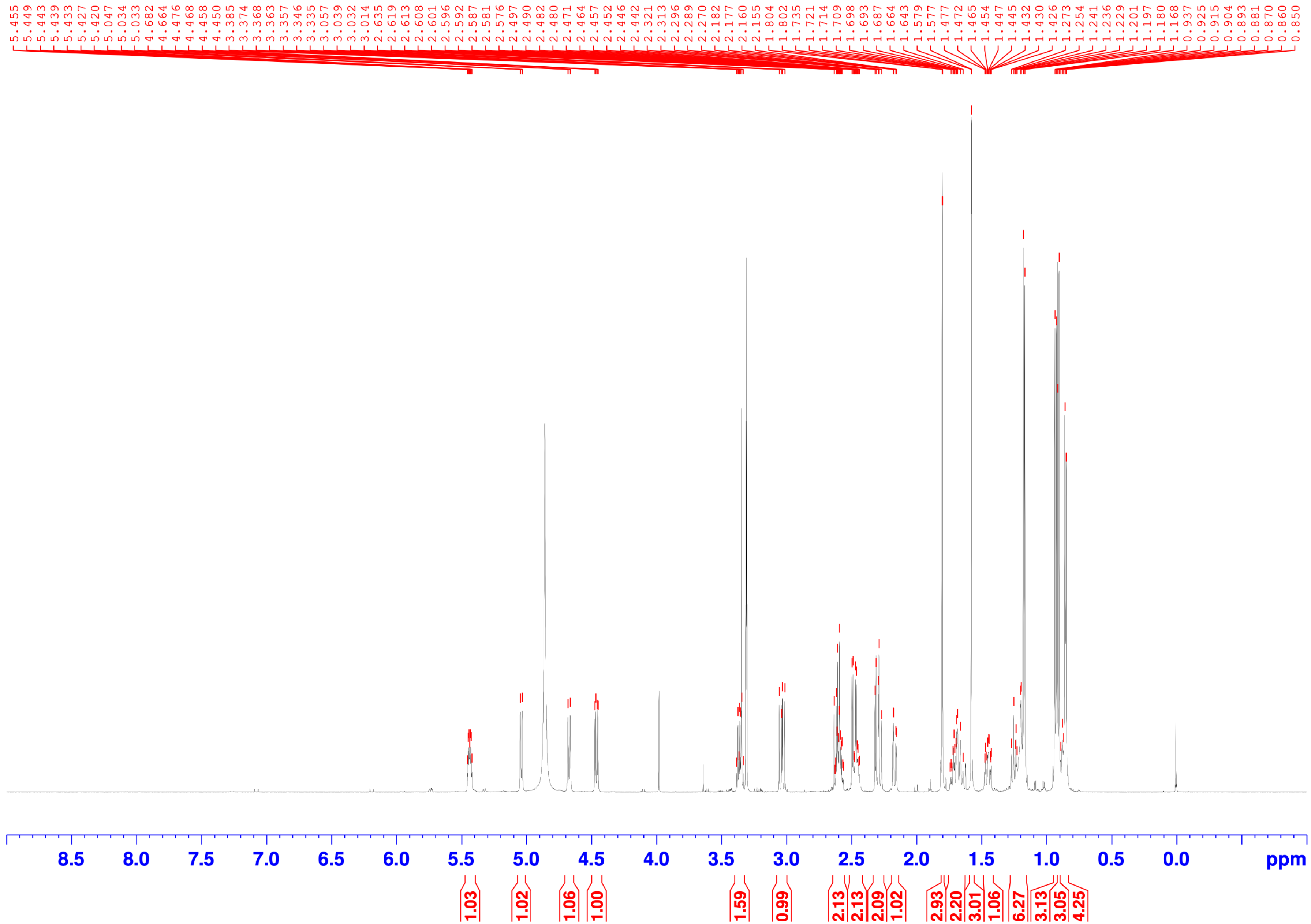

Figure S60. ^{1}H NMR spectrum of **8** in CD_3OD at 600 MHz.

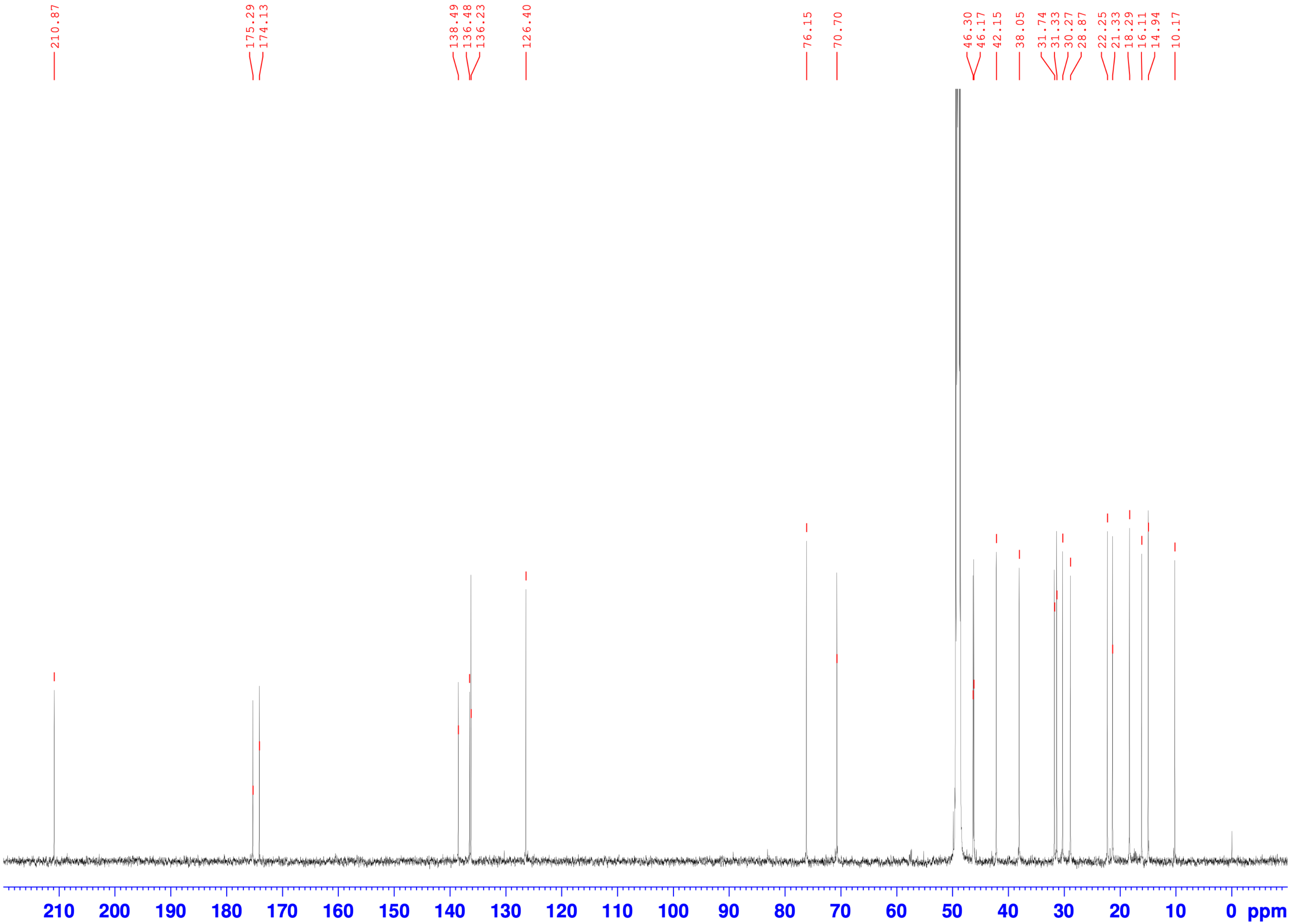

Figure S61. $^{13}C\{^{1}H\}$ NMR spectrum of **8** in CD_3OD at 150 MHz.

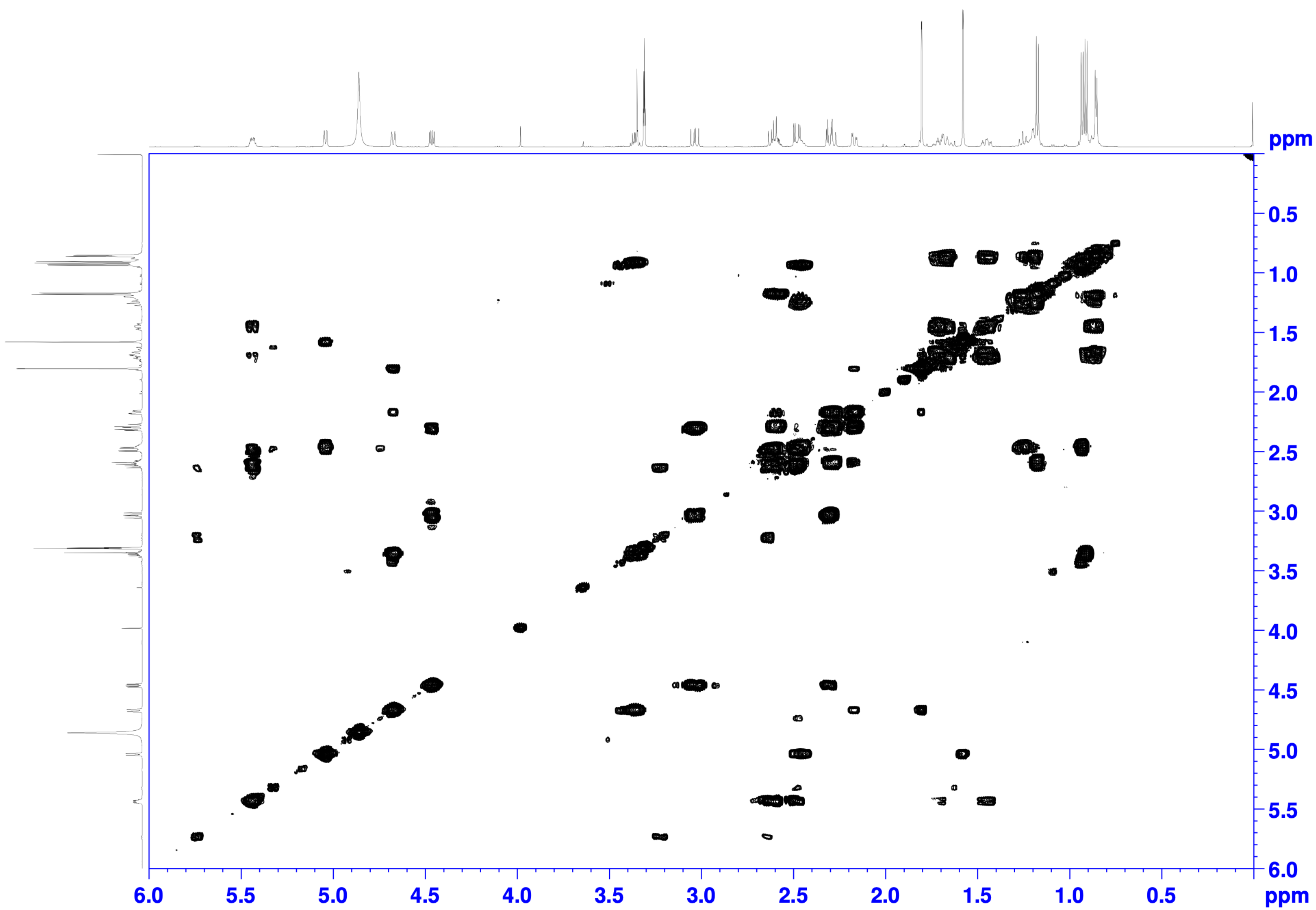

Figure S62. ^{1}H-^{1}H COSY spectrum of **8** in CD_3OD.

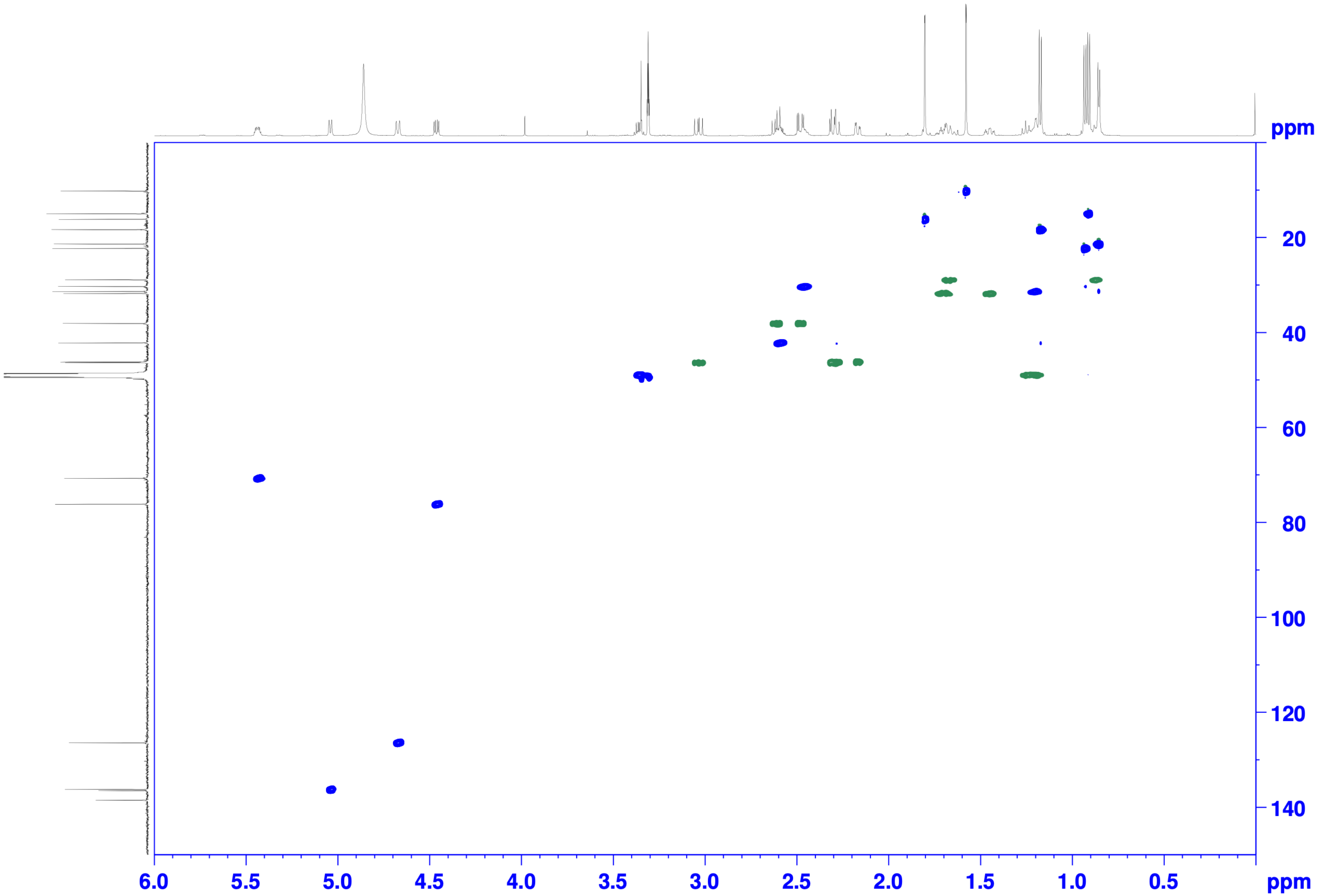

Figure S63. HSQC spectrum of **8** in CD_3OD.

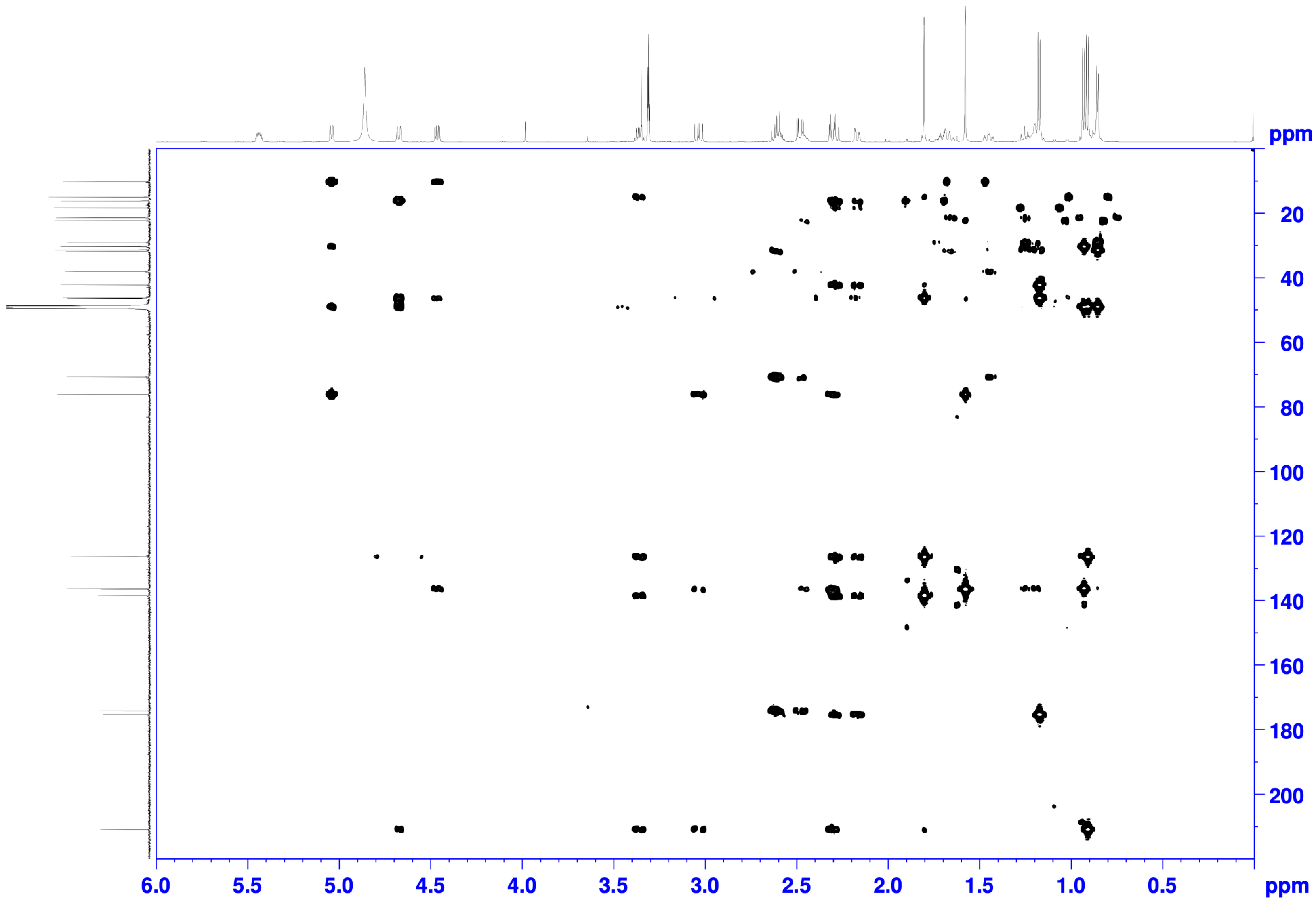

Figure S64. HMBC spectrum of **8** in CD_3OD.

Supplementary References

(1) Tang, J.; Matsuda, Y., Discovery of branching meroterpenoid biosynthetic pathways in *Aspergillus insuetus*: Involvement of two terpene cyclases with distinct cyclization modes. *Chem. Sci.* **2022**, *13*, 10361-10369.

(2) Laoteng, K.; Anantayanon, J.; Chutrakul, C.; Panchanawaporn, S.; Jeennor, S., Transcriptome-based mining of the constitutive promoters for tuning gene expression in *Aspergillus oryzae*. *J. Microbiol.* **2023**, *61*, 199-210.

(3) Tsuboi, H.; Koda, A.; Toda, T.; Minetoki, T.; Hirotsune, M.; Machida, M., Improvement of the *Aspergillus oryzae* enolase promoter (*P-enoA*) by the introduction of *cis*-element repeats. *Biosci. Biotechnol. Biochem.* **2005**, *69*, 206-208.

(4) Koda, A.; Bogaki, T.; Minetoki, T.; Hirotsune, M., 5′ Untranslated region of the *Hsp12* gene contributes to efficient translation in *Aspergillus oryzae*. *Appl. Microbiol. Biotechnol.* **2006**, *70*, 333-336.

(5) Wei, X.; Chen, X.; Chen, L.; Yan, D.; Wang, W.-G.; Matsuda, Y., Heterologous biosynthesis of tetrahydroxanthone dimers: Determination of key factors for selective or divergent synthesis. *J. Nat. Prod.* **2021**, *84*, 1544-1549.

(6) Kubodera, T.; Yamashita, N.; Nishimura, A., Pyrithiamine resistance gene (*ptrA*) of *Aspergillus oryzae*: Cloning, characterization and application as a dominant selectable marker for transformation. *Biosci. Biotechnol. Biochem.* **2000**, *64*, 1416-1421.

(7) Liu, C.; Minami, A.; Ozaki, T.; Wu, J.; Kawagishi, H.; Maruyama, J.-i.; Oikawa, H., Efficient reconstitution of Basidiomycota diterpene erinacine gene cluster in Ascomycota host *Aspergillus oryzae* based on genomic DNA sequences. *J. Am. Chem. Soc.* **2019**, *141*, 15519-15523.

(8) Jin, F. J.; Maruyama, J.; Juvvadi, P. R.; Arioka, M.; Kitamoto, K., Development of a novel quadruple auxotrophic host transformation system by *argB* gene disruption using *adeA* gene and exploiting adenine auxotrophy in *Aspergillus oryzae*. *FEMS Microbiol. Lett.* **2004**, *239*, 79-85.

(9) Wei, X.; Chan, T. K.; Kong, C. T. D.; Matsuda, Y., Biosynthetic characterization, heterologous production, and genomics-guided discovery of GABA-containing fungal heptapeptides. *J. Nat. Prod.* **2023**, *86*, 416-422.

(10) Fujii, T.; Yamaoka, H.; Gomi, K.; Kitamoto, K.; Kumagai, C., Cloning and nucleotide sequence of the ribonuclease T1 gene (*rntA*) from *Aspergillus oryzae* and its expression in *Saccharomyces cerevisiae* and *Aspergillus oryzae*. *Biosci. Biotechnol. Biochem.* **1995**, *59*, 1869-1874.

(11) Matsuda, Y.; Bai, T.; Phippen, C. B. W.; Nødvig, C. S.; Kjærbølling, I.; Vesth, T. C.; Andersen, M. R.; Mortensen, U. H.; Gotfredsen, C. H.; Abe, I.; Larsen, T. O., Novofumigatonin biosynthesis involves a non-heme iron-dependent endoperoxide isomerase for orthoester formation. *Nat. Commun.* **2018**, *9*, 2587.

(12) (a) Wei, X.; Matsuyama, T.; Sato, H.; Yan, D.; Chan, P. M.; Miyamoto, K.; Uchiyama, M.; Matsuda, Y., Molecular and computational bases for spirofuranone formation in setosusin biosynthesis. *J. Am. Chem. Soc.* **2021**, *143*, 17708-17715; (b) Chen, L.; Tang, J.-W.; Liu, Y. Y.; Matsuda, Y., Aspcandine: A pyrrolobenzazepine alkaloid synthesized by a fungal nonribosomal peptide synthetase-polyketide synthase hybrid. *Org. Lett.* **2022**, *24*, 4816-4819.

(13) Wang, W.-G.; Wang, H.; Du, L.-Q.; Li, M.; Chen, L.; Yu, J.; Cheng, G.-G.; Zhan, M.-T.; Hu, Q.-F.; Zhang, L.; Yao, M.; Matsuda, Y., Molecular basis for the biosynthesis of an unusual chain-fused polyketide, gregatin A. *J. Am. Chem. Soc.* **2020**, *142*, 8464-8472.

(14) Marty, M. T.; Baldwin, A. J.; Marklund, E. G.; Hochberg, G. K. A.; Benesch, J. L. P.; Robinson, C. V., Bayesian deconvolution of mass and ion mobility spectra: From binary interactions to polydisperse ensembles. *Anal. Chem.* **2015**, *87*, 4370-4376.

(15) (a) Annang, F.; Pérez-Moreno, G.; González-Menéndez, V.; Lacret, R.; Pérez-Victoria, I.; Martín, J.; Cantizani, J.; de Pedro, N.; Choquesillo-Lazarte, D.; Ruiz-Pérez, L. M.; González-Pacanowska, D.; Genilloud, O.; Vicente, F.; Reyes, F., Strasseriolides A–D, a family of antiplasmodial macrolides isolated from the fungus *Strasseria geniculata* CF-247251. *Org. Lett.* **2020**, *22*, 6709-6713; (b) Isak, D.; Schwartz, L. A.; Schulthoff, S.; Pérez-Moreno, G.; Bosch-Navarrete, C.; González-Pacanowska, D.; Fürstner, A., Collective and diverted total synthesis of the strasseriolides: A family of macrolides endowed with potent antiplasmodial and antitrypanosomal activity. *Angew. Chem. Int. Ed.* **2024**, *63*, e202408725.

(16) Thynne, E.; Mead, O. L.; Chooi, Y.-H.; McDonald, M. C.; Solomon, P. S., Acquisition and loss of secondary metabolites shaped the evolutionary path of three emerging phytopathogens of wheat. *Genome Biol. Evol.* **2019**, *11*, 890-905.

(17) Gilchrist, C. L. M.; Chooi, Y.-H., clinker & clustermap.js: Automatic generation of gene cluster comparison figures. *Bioinformatics* **2021**, *37*, 2473-2475.
